# Author Correction: Copper-catalyzed dehydrogenative *γ*-C(sp^3^)-H amination of saturated ketones for synthesis of polysubstituted anilines

**DOI:** 10.1038/s41467-021-23464-7

**Published:** 2021-05-10

**Authors:** Rong Hu, Fa-Jie Chen, Xiaofeng Zhang, Min Zhang, Weiping Su

**Affiliations:** 1 School of Physical Science and Technology, ShanghaiTech University, Shanghai, 201210 China; 2 State Key Laboratory of Structural Chemistry, Center for Excellence in Molecular Synthesis, Fujian Institute of Research on the Structure of Matter, Chinese Academy of Sciences, Fuzhou, 350002 China; 3 The University of the Chinese Academy of Sciences, Beijing, 100049 China

**Keywords:** Homogeneous catalysis, Catalytic mechanisms, Synthetic chemistry methodology

Correction to: *Nature Communications* 10.1038/s41467-019-11624-9, published online 15 August 2019.

The original version of this Article contained an error in the author affiliations.

The affiliation of Rong Hu with The University of the Chinese Academy of Sciences, Beijing 100049, China was inadvertently omitted.

This has now been corrected in both the PDF and HTML versions of the Article.

