# Supplementary material for: Copper-catalyzed dehydrogenative γ-C(sp3)-H amination of saturated ketones for synthesis of polysubstituted anilines
Source: Nat Commun. 2019 Aug 15;10:3681. doi: 10.1038/s41467-019-11624-9 (PMC6695438; doi:10.1038/s41467-019-11624-9)
Supplement: Supplementary file 1 — Supplementary Information [file 41467_2019_11624_MOESM1_ESM.pdf]

Supplementary Information

Cu-Catalyzed Dehydrogenative γ-C(sp^3)-H Amination of Saturated Ketones for Synthesis of Polysubstituted Anilines

Hu et al.

Supplementary Methods

General Information. All reactions were carried out under an atmosphere of nitrogen with dry solvents. Unless otherwise noted, commercial chemical reagents and solvents were purchased from sources （Sigma-Aldrich Co., J&K Chemicals, Acros Organics, Alfa Aesar and Adamas-beta®, Innochem, Aladdin, Sigma-Aldrich, TCI, Accela, and 3A Chemicals) and used directly without further purification. Toluene was distilled from metal Na and stored under nitrogen atmosphere. CH_3CN, 1,2-dichlorobenzene were distilled over CaH_2 and stored under nitrogen atmosphere. 4Å molecular sieve was dried at 150 °C overnight and stored in the nitrogen-filled glove-box. NMR spectra were recorded on a Bruker AVANCE 400 spectrometer (^{1}H NMR: 400 MHz, ^{13}C NMR: 100 MHz, ^{19}F NMR: 377 MHz). Chemical shifts were reported in parts per million (ppm) and calibrated using TMS (0 ppm for ^{1}H NMR) and residual undeuterated solvent $CDCl_3$ (7.26 ppm for ^{1}H NMR, 77.16 ppm for ^{13}C NMR). ESI-HRMS (High resolution mass spectra) spectra were performed by the Shanghai Institute of Organic Chemistry, Chinese Academic of Sciences (Instrument: Thermo Scientific Q Exactive HF Orbitrap-FTMS, Operated Mode: ESI Positive Ion Mode).

General Procedure A for the formation of products 4a - 4x, 5a - 5j, 8a, 8c and 8d: In a nitrogen-filled glovebox, a 25 mL Schlenk tube equipped with a stir bar was charged with $Cu(OAc)_2$ (7.26 mg, 0.04 mmol, 10 mol%), 2,2'-bipyridine (6.25 mg, 0.04 mmol, 10 mol%), N-substituted maleimide (0.6 mmol, 1.5 equiv.) and TEMPO (187.50 mg, 1.2 mmol, 3.0 equiv.), The tube was fitted with a rubber septum and moved out of the glove box. Then amine (0.4 mmol), ketone (0.8 mmol, 2.0 equiv.), p-Toluenesulfonic acid (6.88 mg, 0.04 mmol, 10 mol%, 12 wt.% solution in pure acetic acid), and toluene (1.5 mL) were added in turn to the Schlenk tube through the rubber septum using syringes, and the septum was replaced with a Teflon screwcap under nitrogen flow. The reaction mixture was allowed to stir for 48 h at 120 °C. After completion of the reaction, the reaction mixture was cooled to room temperature. Then the reaction mixture was diluted with ethyl acetate (10 mL), followed by filtration through a pad of silica gel with several washings. Then the filtrate was concentrated under reduced pressure, and purified by flash column chromatography on silica gel to provide the desired product.

General Procedure B for the formation of products 5k - 5z and 8b: In a nitrogen-filled glovebox, a 25 mL Schlenk tube equipped with a stir bar was charged with $Cu(OAc)_2$ (7.26 mg, 0.04 mmol, 10 mol%), 2,2'-bipyridine (6.25 mg, 0.04 mmol, 10 mol%), ketone (0.4 mmol), TEMPO (187.50 mg, 1.2 mmol, 3.0 equiv.), CsOAc (15.36 mg, 0.08 mmol, 0.2 equiv.). The tube was fitted with a rubber septum and moved out of the glove box. Then amine (0.4 mmol, 1.0 equiv.), and toluene (1.5 mL) were added in turn to the Schlenk tube through the rubber septum using syringes, and then the septum was replaced with a Teflon

screwcap under nitrogen flow. The reaction mixture was allowed to stir at 120 °C for 38 h. Then, N-substituted maleimide (75.08 mg, 0.6 mmol, 1.5 equiv.) was added under air at room temperature, followed by stirring at 120 °C for another 10 h. After cooling to room temperature, the reaction mixture was diluted with ethyl acetate (10 mL), followed by filtration through a pad of silica gel with several washings. Then, the filtrate was concentrated under reduced pressure and purified by flash column chromatography on silica gel to provide the desired product.

General Procedure C for tertiary amines with 1-phenylbutan-1-one (1a) reaction system: In a nitrogen-filled glovebox, a 25 mL Schlenk tube equipped with a stir bar was charged with $Cu(OAc)_2$ (7.26 mg, 0.04 mmol, 10 mol%), 2,2'-bipyridine (6.25 mg, 0.04 mmol, 10 mol%), TEMPO (187.50 mg, 1.2 mmol, 3 equiv) and N-substitutedmaleimide (75.08 mg, 0.6 mmol, 1.5 equiv). The tube was fitted with a rubber septum and moved out of the glove box. Then ketone (0.4 mmol), tertiary amine (0.8 mmol, 2.0 equiv.), p-Toluenesulfonic acid (6.88 mg, 0.04 mmol, 10 mol%, 12 wt.% solution in pure acetic acid) and toluene (1.5 mL) were added in turn to the Schlenk tube through the rubber septum using syringes, and then the septum was replaced with a Teflon screwcap under nitrogen flow. The reaction mixture was stirred at 120 °C for 48 h. Upon cooling to room temperature, the reaction mixture was diluted with 10 mL of ethyl acetate, followed by filtration through a pad of silica gel with several washings. The filtrate was concentrated under reduced pressure, and then purified by flash column chromatography on silica gel to provide the desired product.

General Procedure D for tertiary amines with 1,2-diphenylbutan-1-one (1h) reaction system: In a nitrogen-filled glovebox, a 25 mL Schlenk tube equipped with a stir bar was charged with $Cu(OAc)_2$ (7.26 mg, 0.04 mmol, 10 mol%), 2,2'-bipyridine (6.25 mg, 0.04 mmol, 10 mol%), ketone (0.4 mmol), TEMPO (187.50 mg, 1.2 mmol, 3.0 equiv.), CsOAc (15.36 mg, 0.08 mmol, 0.2 equiv.). The tube was fitted with a rubber septum and moved out of the glove box. Then tertiary amine (0.8 mmol, 2.0 equiv.), and toluene (1.5 mL) were added in turn to the Schlenk tube through the rubber septum using syringes, and then the septum was replaced with a Teflon screwcap under nitrogen flow. The reaction mixture was allowed to stir at 120 °C for 38 h. Then, N-substituted maleimide (75.08 mg, 0.6 mmol, 1.5 equiv.) was added under air at room temperature, followed by stirring at 120 °C for another 10 h. After cooling to room temperature, the reaction mixture was diluted with ethyl acetate (10 mL), followed by filtration through a pad of silica gel with several washings. Then, the filtrate was concentrated under reduced pressure and purified by flash column chromatography on silica gel to provide the desired product.

General Procedure E for Preparation of substrates 1i, 1j, 1k, 1l, 1m, 1w.

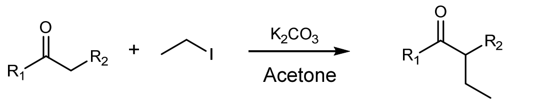

S1 **1i, 1j, 1k, 1l, 1m, 1w.**

The title product was synthesized according to modified literature procedures.[1] A 50 mL round-bottomed flask equipped with a stir bar was charged with **S1** (5.0 mmol), iodoethane (5.5 mmol), K_2CO_3 (7.5 mmol) and acetone （5.0 mL）. Then the mixture was heated to 60 °C for 5 hours. After the mixture was cooled to room temperature, the reaction mixture was diluted with ethyl acetate, followed by filtration through a pad of silica gel with several washings. The filtrate was concentrated under reduced pressure, and then purified by flash column chromatography on silica gel to afford product **1**.

Synthesis of Ethyl 2-benzoylbutanoate (1i)

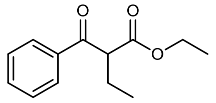

1i

Ethyl 2-benzoylbutanoate was prepared according to the **General Procedure E**. Carried out with ethyl 3-oxo-3-phenylpropanoate (5.0 mmol, 961.05 mg), iodoethane (5.5 mmol, 857.84 mg), K_2CO_3 (7.5 mmol, 1036.58 mg) and 5.0 mL of acetone.at 60 °C for 5 hours. After concentrated and purified by flash chromatography on silica gel (eluent = petroleum ether / ethyl acetate = 100:5), the product was obtained as colorless oil (473.24 mg, 43% yield).

^{1}H NMR (400 MHz, $CDCl_3$) δ 7.95 (d, *J* = 7.5 Hz, 2H), 7.52 (t, *J* = 7.3 Hz, 1H), 7.42 (t, *J* = 7.6 Hz, 2H), 4.19 (t, *J* = 7.1 Hz, 1H), 4.09 (q, *J* = 7.1 Hz, 2H), 2.05 - 1.93 (m, 2H), 1.11 (t, *J* = 7.1 Hz, 3H), 0.94 (t, *J* = 7.4 Hz, 3H).

^{13}C NMR (100 MHz, $CDCl_3$) δ 195.23, 169.93, 136.27, 133.42, 128.68, 128.48, 61.19, 55.72, 22.35, 13.95, 12.06. This compound is known.[1] The **^{1}H** and **^{13}C NMR** spectral data are in good agreement with the literature data.

Synthesis of 2-ethyl-1,3-diphenylpropane-1,3-dione (1j)

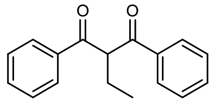

1j

2-Ethyl-1,3-diphenylpropane-1,3-dione was prepared according to the **General Procedure E**. Carried out with 1,3-diphenylpropane-1,3-dione (5.0 mmol, 1120.40 mg), iodoethane (5.5 mmol, 857.84 mg), K_2CO_3 (7.5 mmol, 1036.58 mg) and 5.0 mL of acetone.at 60 °C for 5 hours. After concentrated and purified by flash chromatography on silica gel (eluent = petroleum ether / ethyl acetate = 100:5), the product was obtained as yellow solid (781.58 mg, 62% yield).

^{1}H NMR (400 MHz, $CDCl_3$) δ 7.97 (d, *J* = 7.6 Hz, 4H), 7.56 (t, *J* = 7.4 Hz, 2H), 7.45 (t, *J* = 7.7 Hz, 4H), 5.13 (t, *J* = 6.6 Hz, 1H), 2.17 (p, *J* = 7.3 Hz, 2H), 1.05 (t, *J* = 7.5 Hz, 3H).

^{13}C NMR (100 MHz, $CDCl_3$) δ 196.34, 136.25, 133.60, 128.99, 128.67, 58.78, 23.06, 12.97.

This compound is known.[1] The **^{1}H** and **^{13}C NMR** spectral data are in good agreement with the literature data.

Synthesis of 2-ethyl-1-phenylbutane-1,3-dione (1k)

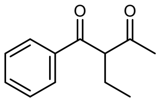

1k

2-Ethyl-1-phenylbutane-1,3-dione was prepared according to the **General Procedure E**. Carried out with 1-phenylbutane-1,3-dione (5.0 mmol, 810.35 mg), iodoethane (5.5 mmol, 857.84 mg), K_2CO_3 (7.5 mmol, 1036.58 mg) and 5.0 mL of acetone.at 60 °C for 5 hours. After concentrated and purified by flash chromatography on silica gel (eluent = petroleum ether / ethyl acetate = 100:10), the product was obtained as colorless oil (503.77 mg, 53% yield).

^{1}H NMR (400 MHz, $CDCl_3$) δ 7.89 (d, J = 7.6 Hz, 2H), 7.47 (t, J = 7.3 Hz, 1H), 7.36 (t, J = 7.7 Hz, 2H), 4.31 (t, J = 5.6 Hz, 1H), 2.03 (s, 3H), 1.99 - 1.81 (m, 2H), 0.84 (t, J = 7.4 Hz, 3H).

^{13}C NMR (100 MHz, $CDCl_3$) δ 204.15, 196.38, 136.37, 133.52, 128.69, 128.46, 64.45, 27.85, 22.23, 11.99.

This compound is known.[2] The **^{1}H** and **^{13}C NMR** spectral data are in good agreement with the literature data.

Synthesis of ethyl 2-(4-nitrobenzoyl)butanoate (1l)

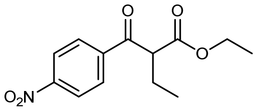

1l

Ethyl 2-(4-nitrobenzoyl)butanoate was prepared according to the **General Procedure E**. Carried out with ethyl 3-(4-nitrophenyl)-3-oxopropanoate (5.0 mmol, 1185.30 mg), iodoethane (5.5 mmol, 857.84 mg), K_2CO_3 (7.5 mmol, 1036.58 mg) and 5.0 mL of acetone.at 60 °C for 5 hours. After concentrated and purified by flash chromatography on silica gel (eluent = petroleum ether / ethyl acetate = 100:10), the product was obtained as yellow oil (755.54 mg, 57% yield).

^{1}H NMR (400 MHz, $CDCl_3$) δ 8.20 (d, J = 8.8 Hz, 2H), 8.06 (d, J = 8.8 Hz, 2H), 4.18 (t, J = 7.1 Hz, 1H), 4.04 (q, J = 7.1 Hz, 2H), 1.94 (p, J = 7.3 Hz, 2H), 1.06 (t, J = 7.2 Hz, 3H), 0.89 (t, J = 7.5 Hz, 3H).

^{13}C NMR (100 MHz, $CDCl_3$) δ 193.81, 169.12, 150.21, 140.72, 129.42, 123.73, 61.41, 56.01, 21.96, 13.79, 11.73.

HRMS (ESI) Calcd. for $C_{13}H_{16}NO_5$ ($[M+H]^+$): 266.1023, found: 266.1019.

Synthesis of ethyl 2-(4-(trifluoromethyl)benzoyl)butanoate (1m)

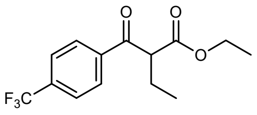

1m

Ethyl 2-(4-(trifluoromethyl)benzoyl)butanoate was prepared according to the **General Procedure E**. Carried out with ethyl 3-oxo-3-(4-(trifluoromethyl)phenyl)propanoate (5.0 mmol, 1300.35 mg), iodoethane (5.5 mmol, 857.84 mg), K_2CO_3 (7.5 mmol, 1036.58 mg) and 5.0 mL of acetone.at 60 °C for 5 hours. After concentrated and purified by flash chromatography on silica gel (eluent = petroleum ether / ethyl acetate = 100:10), the product was obtained as colorless oil (590.61 mg, 41% yield).

^{1}H NMR (400 MHz, $CDCl_3$) δ 8.08 (d, J = 8.2 Hz, 2H), 7.72 (d, J = 8.2 Hz, 2H), 4.19 (t, J = 7.1 Hz, 1H), 4.13 (q, J = 7.1 Hz, 2H), 2.04 (p, J = 7.2 Hz, 2H), 1.15 (t, J = 7.4 Hz, 3H), 0.98 (t, J = 7.4 Hz, 3H).

^{13}C NMR (100 MHz, $CDCl_3$) δ 194.44, 169.63, 139.15, 134.73 (q, $^2J_{C\text{-}F}$ = 32.56 Hz), 128.96, 125.89 (q, $^3J_{C\text{-}F}$ = 3.03 Hz), 123.61 (q, $^1J_{C\text{-}F}$ = 272.78 Hz), 61.64, 56.26, 22.31, 14.07, 12.11.

^{19}F NMR (377 MHz, $CDCl_3$) δ -63.15.

HRMS (ESI) Calcd. for $C_{14}H_{16}O_3F_3$ ($[M+H]^+$): 289.1046, found: 289.1039.

Synthesis of ethyl 3-(4-methoxyphenyl)pentan-2-one (1w)

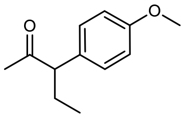

1w

3-(4-Methoxyphenyl)pentan-2-one was prepared according to the **General Procedure E**. Carried out with 1-(4-methoxyphenyl)propan-2-one (5.0 mmol, 820.40 mg), iodoethane (5.5 mmol, 857.84 mg), K_2CO_3 (7.5 mmol, 1036.58 mg) and 5.0 mL of acetone.at 60 °C for 5 hours. After concentrated and purified by flash chromatography on silica gel (eluent = petroleum ether / ethyl acetate = 100:10), the product was obtained as colorless oil (393.85 mg, 41% yield).

^{1}H NMR (400 MHz, $CDCl_3$) δ 7.02 (d, J = 8.6 Hz, 2H), 6.76 (d, J = 8.6 Hz, 2H), 3.65 (s, 3H), 3.37 (t, J = 7.5 Hz, 1H), 1.92 (s, 4H), 1.57 (dp, J = 14.9, 7.5 Hz, 1H), 0.71 (t, J = 7.4 Hz, 3H).

^{13}C NMR (100 MHz, $CDCl_3$) δ 208.36, 158.56, 130.69, 128.98, 113.99, 60.21, 54.80, 28.55, 24.69, 11.73.

This compound is known.[3] The **^{1}H** and **^{13}C NMR** spectral data are in good agreement with the literature data.

Synthesis of 4-butyrylbenzonitrile (1e)

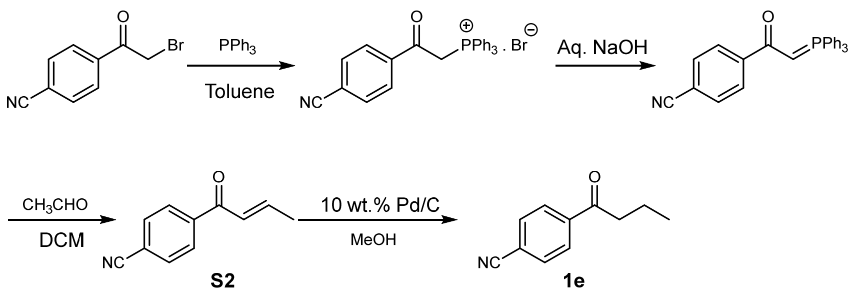

4-Butyrylbenzonitrile was prepared based on the literature procedures.[4] A 50 mL round-bottomed flask equipped with a stir bar was charged with 4-(2-bromoacetyl)benzonitrile (5.0 mmol, 1114.8 mg), triphenylphosphine (5.0 mmol, 1311.45 mg) and toluene (20 mL) under air. Then the mixture was allowed to stir at room temperature until the precipitate was formed. After filtered and washed with toluene, the residue was dissolved in water. To this solution, aqueous sodium hydroxide (1N) was added. Then the reaction mixture was extracted with DCM (2x20 mL). The combined organic layer was dried over anhydrous $MgSO_4$, followed by acetaldehyde (5.0 mmol, 220.25mg). The progress of the reaction was analyzed by TLC. Upon completion, the dichloromethane was evaporated under reduced pressure to afford a solid residue. Then hexane (50 mL) was added followed by the filtration of triphenylphosphine oxide. The filtrate was concentrated under reduced pressure to obtain crude (*E*)-4-methyl-1-phenylpent-2-en-1-one of **S2** for the next step. Then **S2** was treated with 10 wt.% Pd/C in methanol and refluxed under H_2 atmosphere for 4 h. Upon completion of the reaction, the reaction mixture was filtered through a pad of silica gel with several washings. Then the filtrate was concentrated under reduced pressure and purified by flash column chromatography on silica gel (eluent = petroleum ether / ethyl acetate = 100:10) to provide **1e** as white solid (389.43 mg, 45% overall yield).

^{1}H NMR (400 MHz, $CDCl_3$) δ 7.97 (d, *J* = 8.3 Hz, 2H), 7.69 (d, *J* = 8.3 Hz, 2H), 2.90 (t, *J* = 7.2 Hz, 2H), 1.68 (h, *J* = 7.3 Hz, 2H), 0.92 (t, *J* = 7.3 Hz, 3H).

^{13}C NMR (100 MHz, $CDCl_3$) δ 198.99, 140.00, 132.55, 128.49, 118.07, 116.18, 40.82, 17.47, 13.81.

This compound is known.[5] The **^{1}H** and **^{13}C NMR** spectral data are in good agreement with the literature data.

Synthesis of 1-phenylhexan-3-one (1s)

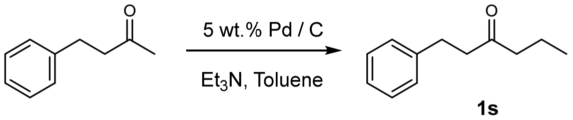

1s

1-Phenylhexan-3-one was prepared based on the literature procedures.[6] In a nitrogen-filled glovebox, a 50 mL pressure vessel equipped with a stir bar was charged with 5 wt.% Pd/C (0.212 g, 0.1 mmol). The vessel was fitted with a rubber septum and moved out of the glove box. Then 4-phenylbutan-2-one (0.5 mmol, 74.05 mg), triethylamine (1.5 mmol, 151.79 mg), and toluene (10 mL) were added in turn to the vessel through the rubber septum using syringes, then the septum was replaced with a Teflon screwcap under nitrogen flow. The reaction mixture was allowed to stir at 120 °C for 40 h. Upon the completion of the reaction, the reaction mixture was filtered through a short silica gel pad. The filtrate was concentrated under reduced pressure to remove the solvent and purified by flash column chromatography on silica gel (eluent = petroleum ether / ethyl acetate = 100:10) to provide **1s** as colorless oil (40.51 mg, 46%).

^{1}H NMR (400 MHz, $CDCl_3$) δ 7.24 (t, *J* = 7.5 Hz, 2H), 7.18 - 7.12 (m, 3H), 2.87 (t, *J* = 7.6 Hz, 2H), 2.65 (t, *J* = 7.6 Hz, 2H), 2.30 (t, *J* = 7.2 Hz, 2H), 1.57 (h, *J* = 7.2 Hz, 2H), 0.88 (t, *J* = 7.2 Hz, 3H).

^{13}**C NMR** (100 MHz, $CDCl_3$) δ 209.41, 140.93, 128.16, 128.03, 125.73, 44.47, 43.86, 29.45, 16.90, 13.44.
HRMS (ESI) Calcd. for $C_{12}H_{17}O$ ([M+H]$^+$): 177.1274, found: 177.1271.

Supplementary Table 1. Optimization of the reaction conditions[a]

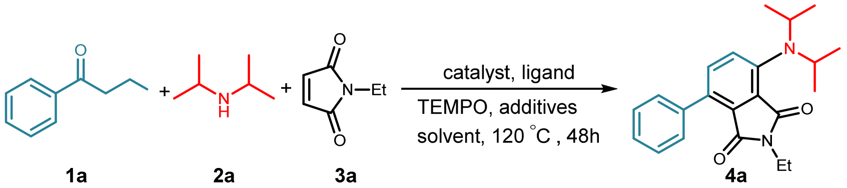

entry	catalyst	ligand	TEMPO	additives	solvent	yield (%)
1	$Cu(OAc)_2$	none	3.0 equiv.	-	$PhCH_3$	67
2	$Cu(OAc)_2$	bpy	3.0 equiv.	-	$PhCH_3$	80
3	$Cu(OAc)_2$	bpy	2.0 equiv.	-	$PhCH_3$	71
4	$Cu(OAc)_2$	bpy	3.0 equiv.	0.2 equiv. *o*-nitro-benzoic acid	$PhCH_3$	85
5	$Cu(OAc)_2$	bpy	3.0 equiv.	0.2 equiv. *o*-nitro-benzoic acid	*o*-dichlorobenzene	67
6	$Cu(OAc)_2$	bpy	3.0 equiv.	0.2 equiv. *o*-nitro-benzoic acid	CH_3CN	63
7	$Cu(OAc)_2$	bpy	3.0 equiv.	0.2 equiv. benzoic acid	$PhCH_3$	53
8	$Cu(OAc)_2$	bpy	3.0 equiv.	0.2 equiv. acetic acid	$PhCH_3$	56
**9**	**$Cu(OAc)_2$**	**bpy**	**3.0 equiv.**	**0.10 equiv. *p*-TsOH**[b]	**$PhCH_3$**	**96**
10	$Cu(OAc)_2$	bpy	3.0 equiv.	0.05 equiv. *p*-TsOH[b]	$PhCH_3$	87
11	$Cu(OAc)_2$	bpy	3.0 equiv.	0.2 equiv. *p*-TsOH[b]	$PhCH_3$	91
12	$Cu(OAc)_2$	bpy	3.0 equiv.	0.10 equiv. *p*-TsOH·H_2O	$PhCH_3$	83
13	-	-	3.0 equiv.	0.08 equiv. *p*-TsOH·H_2O	$PhCH_3$	30
14	$Cu(OAc)_2$	none	3.0 equiv.	0.10 equiv. *p*-TsOH[b]	$PhCH_3$	81
15	$Cu(OAc)_2$	1,10-Phen	3.0 equiv.	0.10 equiv. *p*-TsOH[b]	$PhCH_3$	83
16	$Cu(OAc)_2$	bpy	3.0 equiv.	200 mg 4Å MS	$PhCH_3$	51
17	$Cu(OTf)_2$	bpy	3.0 equiv.	-	$PhCH_3$	67
18	$Cu(OAc)_2$	bpy	3.0 equiv.	0.1 equiv. CsOAc	$PhCH_3$	81
19	$Cu(OAc)_2$	bpy	3.0 equiv.	0.2 equiv. CsOAc	$PhCH_3$	85
20	$Cu(OAc)_2$	bpy	3.0 equiv.	0.2 equiv. Cs_2CO_3	$PhCH_3$	61

21	$Cu(OAc)_2$	bpy	3.0 equiv.	0.2 equiv. Na_2CO_3	$PhCH_3$	53
22	$Cu(OAc)_2$	bpy	3.0 equiv.	0.2 equiv. NaOAc	$PhCH_3$	78
23	$Cu(OAc)_2$	bpy	3.0 equiv.	0.2 equiv. DBU	$PhCH_3$	58
24	$Cu(OAc)_2$	bpy	3.0 equiv.	0.2 equiv. DMAP	$PhCH_3$	69
25	$Cu(OAc)_2$	bpy	3.0 equiv.	0.2 equiv. DABCO	$PhCH_3$	51
26	$Cu(OAc)_2$	bpy	3.0 equiv.	0.2 equiv. CsF	$PhCH_3$	73
27	-	-	3.0 equiv.	0.1 equiv. *p*-TsOH[b]	$PhCH_3$	33
28	-	-	3.0 equiv.	0.2 equiv. *p*-TsOH[b]	$PhCH_3$	21
29	-	-	3.0 equiv.	-	$PhCH_3$	trace

[a]Reaction conditions: **1a** (0.8 mmol, 2.0 equiv.), **2a** (0.4 mmol), **3a** (0.6 mmol, 1.5 equiv.), catalyst (10 mol%), ligand (10 mol%), TEMPO, additives, solvent (1.5 mL), N_2, 120 °C for 48 h. Isolated yield. [b]12 wt.% *p*-TsOH solution in pure acetic acid.

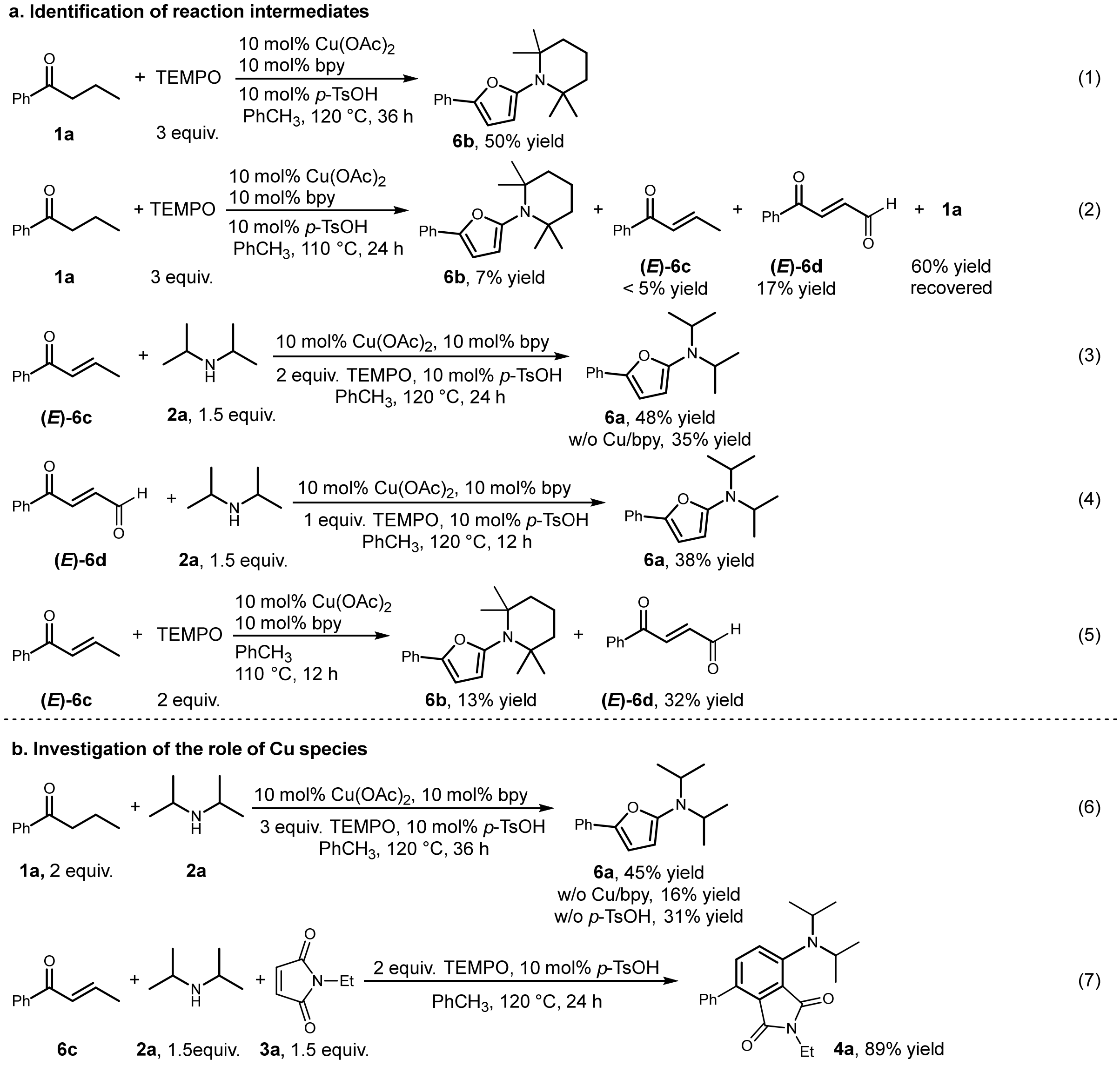

Supplementary Figure 1 Preliminary studies of mechanism[a]. [a] isolated yields are reported. **a** The control experiments show that the cascade sequence may proceed through initial ketone *α,β*-dehydrogenation desaturation that activates the adjacent *γ*-C(sp^3)-H bond. **b** The control experiments show that Cu catalyst is the main contributor to the ketone *α,β*-dehydrogenation desaturation step and *p*-TsOH catalyze the conversions of enone intermediates.

General procedure for the equation 1 in **Supplementary Figure 1**. In a nitrogen-filled glovebox, a 25 mL Schlenk tube equipped with a stir bar was charged with Cu(OAc)$_2$ (7.26 mg, 0.04 mmol, 10 mol%), 2,2'-bipyridine (6.25 mg, 0.04 mmol, 10 mol%), and TEMPO (187.50 mg, 1.2 mmol, 3.0 equiv.). The tube was fitted with a rubber septum and moved out of the glove box. Then 1-phenylbutan-1-one (59.24 mg, 0.4 mmol), p-Toluenesulfonic acid (6.88 mg, 0.04 mmol, 10 mol%, 12 wt.% solution in pure acetic acid), and toluene (1.5 mL) were added in turn to the Schlenk tube through the rubber septum using syringes, and the septum was replaced with a Teflon screwcap under nitrogen flow. The reaction mixture was allowed to stir for 36 h at 120 °C. After completion of the reaction, the reaction mixture was cooled to

room temperature. Then the reaction mixture was diluted with ethyl acetate (10 mL), followed by filtration through a pad of silica gel with several washings. Then the filtrate was concentrated under reduced pressure, and purified by flash column chromatography on silica gel to obtain **6b** as colorless solid in 50% yield.

General procedure for the equation 2 in **Supplementary Figure 1**. In a nitrogen-filled glovebox, a 25 mL Schlenk tube equipped with a stir bar was charged with $Cu(OAc)_2$ (7.26 mg, 0.04 mmol, 10 mol%), 2,2'-bipyridine (6.25 mg, 0.04 mmol, 10 mol%), and TEMPO (187.50 mg, 1.2 mmol, 3.0 equiv.). The tube was fitted with a rubber septum and moved out of the glove box. Then 1-phenylbutan-1-one (59.24 mg, 0.4 mmol), p-Toluenesulfonic acid (6.88 mg, 0.04 mmol, 10 mol%, 12 wt.% solution in pure acetic acid), and toluene (1.5 mL) were added in turn to the Schlenk tube through the rubber septum using syringes, and the septum was replaced with a Teflon screwcap under nitrogen flow. The reaction mixture was allowed to stir for 24 h at 110 °C. After completion of the reaction, the reaction mixture was cooled to room temperature. Then the reaction mixture was diluted with ethyl acetate (10 mL), followed by filtration through a pad of silica gel with several washings. Then the filtrate was concentrated under reduced pressure, and purified by flash column chromatography on silica gel to obtain **(E)-6b** as colorless solid in 7% isolated yield, **6c** as yellow oil in trace amount, **(E)-6d** as red solid in 17% isolated yield, and **1a** was recovered in 60% isolated yield, respectively.

General procedure for the equation 3 in **Supplementary Figure 1**. (E)-1-phenylbut-2-en-1-one (**6c**) was prepared according to literatures.[7] In a nitrogen-filled glovebox, a 25 mL Schlenk tube equipped with a stir bar was charged with $Cu(OAc)_2$ (7.26 mg, 0.04 mmol, 10 mol%), 2,2'-bipyridine (6.25 mg, 0.04 mmol, 10 mol%), and TEMPO (125 mg, 0.8 mmol, 2.0 equiv.). The tube was fitted with a rubber septum and moved out of the glove box. Then **6c** (58.43 mg, 0.4 mmol), diisopropylamine (60.67 mg, 0.6 mmol, 1.5 equiv.), p-Toluenesulfonic acid (6.88 mg, 0.04 mmol, 10 mol%, 12 wt.% solution in pure acetic acid), and toluene (1.5 mL) were added in turn to the Schlenk tube through the rubber septum using syringes, and the septum was replaced with a Teflon screwcap under nitrogen flow. The reaction mixture was allowed to stir for 24 h at 120 °C. After completion of the reaction, the reaction mixture was cooled to room temperature. Then the reaction mixture was diluted with ethyl acetate (10 mL), followed by filtration through a pad of silica gel with several washings. Then the filtrate was concentrated under reduced pressure, and purified by flash column chromatography on silica gel to obtain **6a** as colorless oil in 48% isolated yield. A control experiment without $Cu(OAc)_2$/bpy was conducted under the otherwise same circumstance. The desired product **6a** was isolated in 35% yield.

General procedure for the equation 4 in **Supplementary Figure 1**. In a nitrogen-filled glovebox, a 25 mL Schlenk tube equipped with a stir bar was charged with $Cu(OAc)_2$ (7.26 mg, 0.04 mmol, 10 mol%), 2,2'-

bipyridine (6.25 mg, 0.04 mmol, 10 mol%), (E)-4-oxo-4-phenylbut-2-enal (64.02mg, 0.4 mmol) and TEMPO (62.5 mg, 0.4 mmol, 1.0 equiv.). The tube was fitted with a rubber septum and moved out of the glove box. Then diisopropylamine (60.67 mg, 0.6 mmol, 1.5 equiv.), p-Toluenesulfonic acid (6.88 mg, 0.04 mmol, 10 mol%, 12 wt.% solution in pure acetic acid), and toluene (1.5 mL) were added in turn to the Schlenk tube through the rubber septum using syringes, and the septum was replaced with a Teflon screwcap under nitrogen flow. The reaction mixture was allowed to stir for 12 h at 120 °C. After completion of the reaction, the reaction mixture was cooled to room temperature. Then the reaction mixture was diluted with ethyl acetate (10 mL), followed by filtration through a pad of silica gel with several washings. Then the filtrate was concentrated under reduced pressure, and purified by flash column chromatography on silica gel to obtain **6a** in 38% yield.

General procedure for the equation 5 in **Supplementary Figure 1**. In a nitrogen-filled glovebox, a 25 mL Schlenk tube equipped with a stir bar was charged with $Cu(OAc)_2$ (7.26 mg, 0.04 mmol, 10 mol%), 2,2'-bipyridine (6.25 mg, 0.04 mmol, 10 mol%) and TEMPO (125 mg, 0.8 mmol, 2.0 equiv.). The tube was fitted with a rubber septum and moved out of the glove box. Then (E)-1-phenylbut-2-en-1-one (**6c**) (58.43 mg, 0.4 mmol) and toluene (1.5 mL) were added in turn to the Schlenk tube through the rubber septum using syringes, and the septum was replaced with a Teflon screwcap under nitrogen flow. The reaction mixture was allowed to stir for 12 h at 120 °C. After completion of the reaction, the reaction mixture was cooled to room temperature. Then the reaction mixture was diluted with ethyl acetate (10 mL), followed by filtration through a pad of silica gel with several washings. Then the filtrate was concentrated under reduced pressure, and purified by flash column chromatography on silica gel to obtain **6b** in 13% isolated yield and **(E)-6d** in 32% isolated yield.

General procedure for the equation 6 in **Supplementary Figure 1**. In a nitrogen-filled glovebox, a 25 mL Schlenk tube equipped with a stir bar was charged with $Cu(OAc)_2$ (7.26 mg, 0.04 mmol, 10 mol%), 2,2'-bipyridine (6.25 mg, 0.04 mmol, 10 mol%), and TEMPO (187.50 mg, 1.2 mmol, 3.0 equiv.). The tube was fitted with a rubber septum and moved out of the glove box. Then butyrophenone (118.4 mg, 0.8 mmol, 2.0 equiv.), diisopropylamine (40.48 mg, 0.4 mmol), p-Toluenesulfonic acid (6.88 mg, 0.04 mmol, 10 mol%, 12 wt.% solution in pure acetic acid), and toluene (1.5 mL) were added in turn to the Schlenk tube through the rubber septum using syringes, and the septum was replaced with a Teflon screwcap under nitrogen flow. The reaction mixture was allowed to stir for 36 h at 120 °C. After completion of the reaction, the reaction mixture was cooled to room temperature. Then the reaction mixture was diluted with ethyl acetate (10 mL), followed by filtration through a pad of silica gel with several washings. Then the filtrate was concentrated under reduced pressure, and purified by flash column chromatography on silica gel to obtain **6a** as colorless oil in 45% yield. Control experiments without $Cu(OAc)_2$/bpy and p-

Toluenesulfonic acid respectively were conducted under the otherwise same circumstance, and **6a** was isolated in 16% and 31%.

General procedure for the equation 7 in **Supplementary Figure 1**. In a nitrogen-filled glovebox, a 25 mL Schlenk tube equipped with a stir bar was charged with TEMPO (125.1 mg, 0.8 mmol, 2.0 equiv.) and N-ethyl maleimide (75.08 mg, 0.6 mmol, 1.5 equiv.). The tube was fitted with a rubber septum and moved out of the glove box. Then **6c** (58.43 mg, 0.4 mmol), diisopropylamine (60.67 mg, 0.6 mmol, 1.5 equiv.), p-Toluenesulfonic acid (6.88 mg, 0.04 mmol, 10 mol%, 12 wt.% solution in pure acetic acid), and toluene (1.5 mL) were added in turn to the Schlenk tube through the rubber septum using syringes, and the septum was replaced with a Teflon screwcap under nitrogen flow. The reaction mixture was allowed to stir for 24 h at 120 °C. After completion of the reaction, the reaction mixture was cooled to room temperature. Then the reaction mixture was diluted with ethyl acetate (10 mL), followed by filtration through a pad of silica gel with several washings. Then the filtrate was concentrated under reduced pressure, and purified by flash column chromatography on silica gel. **4a** was isolated in 89% yield as yellow solid.

Gram-scale Synthesis of 5o

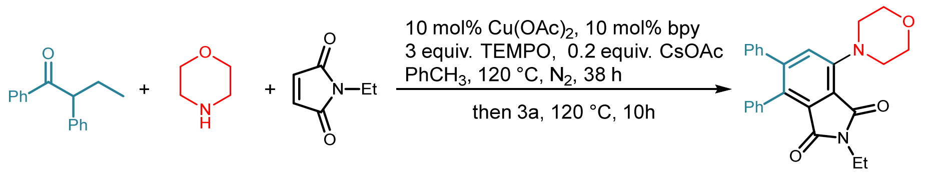

1 **2o** **3a** **5o**, 70%

In a nitrogen-filled glovebox, 1,2-diphenylbutan-1-one (1.60 g, 7.14 mmol, 1.0 equiv.), $Cu(OAc)_2$ (0.13 g, 0.71 mmol, 10 mol%), 2,2'-bipyridine (0.11 g, 0.71 mmol, 10 mol%), TEMPO (3.35 g, 21.42 mmol, 3.0 equiv.), CsOAc (0.27 g, 1.43 mmol, 0.2 equiv.) were placed in a 100 mL Schlenk tube equipped with a stir bar. The tube was fitted with a rubber septum and moved out of the glove box. Then morpholine (0.62 g, 7.14 mmol) and toluene (25 mL) were added in turn to the Schlenk tube through the rubber septum using syringes, and the septum was replaced with a Teflon screwcap under nitrogen flow at 120 ºC for 38 h. Upon cooling to room temperature, N-ethyl maleimide (1.34 g, 10.71 mmol, 1.5 equiv.) was added into the tube under the nitrogen atmosphere for another 10h at 120 ºC. After concentrated and purified by flash column chromatography on silica gel (eluent = petroleum ether / ethyl acetate = 100:15), the product **5o** was obtained as yellow solid (2.06 g, 70% yield).

4-(Diisopropylamino)-2-ethyl-7-phenylisoindoline-1,3-dione (4a)

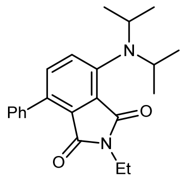

4a

The title product was prepared according to the **General Procedure A**: Carried out with butyrophenone (118.4 mg, 0.8 mmol, 2.0 equiv.), diisopropylamine (40.48 mg, 0.4 mmol), $Cu(OAc)_2$ (7.26 mg, 0.04 mmol, 10 mol%), 2,2'-bipyridine (6.25 mg, 0.04 mmol, 10 mol%), N-ethyl maleimide (75.08 mg, 0.6 mmol, 1.5 equiv.), TEMPO (187.50 mg, 1.2 mmol, 3.0 equiv.), p-Toluenesulfonic acid (6.88 mg, 0.04 mmol, 10 mol%, 12 wt.% solution in pure acetic acid) in toluene (1.5 mL) at 120 °C for 48 h. After concentrated and purified by flash column chromatography on silica gel (eluent = petroleum ether / ethyl acetate = 100:10), the product **4a** was obtained as yellow solid (134.4 mg, 96% yield).

^{1}H NMR (400 MHz, $CDCl_3$): δ 7.50 - 7.55 (m, 2H), 7.37 - 7.48 (m, 5H), 3.91 - 4.05 (m, *J* = 6.7 Hz, 2H), 3.60 - 3.72 (q, *J* = 7.2 Hz, 2H), 1.29 - 1.34 (d, *J* = 6.7 Hz, 12H), 1.20 - 1.26 (t, *J* = 7.2 Hz, 3H);

^{13}C NMR (100 MHz, $CDCl_3$) δ 167.54, 167.12, 147.07, 136.95, 135.54, 133.31, 130.69, 129.73, 129.44, 127.90, 127.79, 122.69, 50.87, 32.58, 22.61, 13.89.

HRMS (ESI) Calcd. for $C_{22}H_{27}N_2O_2$ ($[M+H]^+$): 351.2067, found:.351.2064.

4-(Diisopropylamino)-2-ethyl-7-(4-methoxyphenyl)isoindoline-1,3-dione (4b)

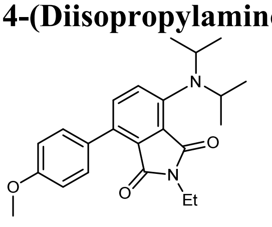

4b

The title product was prepared according to the **General Procedure A**: Carried out with 1-(4-methoxyphenyl)butan-1-one (142.48 mg, 0.8 mmol, 2.0 equiv.), diisopropylamine (40.48 mg, 0.4 mmol), $Cu(OAc)_2$ (7.26 mg, 0.04 mmol, 10 mol%), 2,2'-bipyridine (6.25 mg, 0.04 mmol, 10 mol%), N-ethyl maleimide (75.08 mg, 0.6 mmol, 1.5 equiv.), TEMPO (187.50 mg, 1.2 mmol, 3.0 equiv.), p-Toluenesulfonic acid (6.88 mg, 0.04 mmol, 10 mol%, 12 wt.% solution in pure acetic acid) in toluene (1.5 mL) at 120 °C for 48 h. After concentrated and purified by flash column chromatography on silica gel (eluent = petroleum ether / ethyl acetate = 100:10), the product **4b** was obtained as yellow solid (132.32 mg, 87% yield).

^{1}H NMR (400 MHz, $CDCl_3$) δ 7.45 (dd, *J* = 15.0, 8.5 Hz, 3H), 7.36 (d, *J* = 8.5 Hz, 1H), 6.97 (d, *J* = 8.3 Hz, 2H), 3.93 (p, *J* = 6.7 Hz, 2H), 3.84 (s, 3H), 3.64 (q, *J* = 7.1 Hz, 2H), 1.28 (d, *J* = 6.8 Hz, 13H), 1.21 (t, *J* = 7.2 Hz, 3H). **^{13}C NMR** (100 MHz, $CDCl_3$) δ 167.79, 167.18, 159.56, 146.78, 135.61, 133.42, 131.12, 130.79, 129.38, 129.21, 123.16, 113.35, 55.30, 50.88, 32.63, 22.65, 13.98.

HRMS (ESI) Calcd. for $C_{23}H_{29}N_2O_3$ ($[M+H]^+$): 381.2173, found: 381.2168.

4-(4-Bromophenyl)-7-(diisopropylamino)-2-ethylisoindoline-1,3-dione (4c)

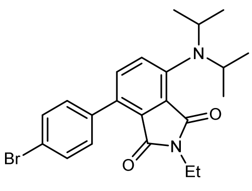

4c

The title product was prepared according to the **General Procedure A**: Carried out with 1-(4-bromophenyl)butan-1-one (180.81 mg, 0.8 mmol, 2.0 equiv.), diisopropylamine (40.48 mg, 0.4 mmol), $Cu(OAc)_2$ (7.26 mg, 0.04 mmol, 10 mol%), 2,2'-bipyridine (6.25 mg, 0.04 mmol, 10 mol%), N-ethyl maleimide (75.08 mg, 0.6 mmol, 1.5 equiv.), TEMPO (187.50 mg, 1.2 mmol, 3.0 equiv.), p-Toluenesulfonic acid (6.88 mg, 0.04 mmol, 10 mol%, 12 wt.% solution in pure acetic acid) in toluene (1.5 mL) at 120 ºC for 48 h. After concentrated and purified by flash column chromatography on silica gel (eluent = petroleum ether / ethyl acetate = 100:10), the product **4c** was obtained as yellow solid (150.69 mg, 88% yield).

^{1}H NMR (400 MHz, $CDCl_3$) δ 7.55 (t, J = 2.0 Hz, 1H), 7.53 (t, J = 2.0 Hz, 1H), 7.43 (d, J = 8.6 Hz, 1H), 7.38 (t, J = 2.0 Hz, 1H), 7.37 (t, J = 2.0 Hz, 1H), 7.32 (d, J = 8.6 Hz, 1H), 3.97 (m, J = 6.8 Hz, 2H), 3.63 (q, J = 7.2 Hz, 2H), 1.31 (d, J = 6.8 Hz, 12H), 1.20 (t, J = 7.2 Hz, 3H).

^{13}C NMR (100 MHz, $CDCl_3$) δ 167.56, 167.08, 147.44, 135.91, 135.25, 131.44, 131.16, 131.00, 130.14, 129.90, 122.27, 121.85, 51.01, 32.67, 22.67, 13.92.

HRMS (ESI) Calcd. for $C_{22}H_{26}N_2O_2Br$ ($[M+H]^+$): 429.1172, found: 429.1170.

4-(Diisopropylamino)-2-ethyl-7-(4-fluorophenyl)isoindoline-1,3-dione (4d)

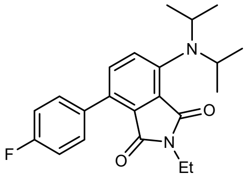

4d

The title product was prepared according to the **General Procedure A**: Carried out with 1-(4-fluorophenyl)butan-1-one (132.86 mg, 0.8 mmol, 2 equiv), diisopropylamine (40.48 mg, 0.4 mmol), $Cu(OAc)_2$ (7.26 mg, 0.04 mmol, 10 mol%), 2,2'-bipyridine (6.25 mg, 0.04 mmol, 10 mol%), N-ethyl maleimide (75.08 mg, 0.6 mmol, 1.5 equiv), TEMPO (187.50 mg, 1.2 mmol, 3 equiv), p-Toluenesulfonic acid (6.88 mg, 0.04 mmol, 10 mol%, 12 wt.% solution in pure acetic acid) in toluene (1.5 mL) at 120 ºC for 48 h. After concentrated and purified by flash column chromatography on silica gel (eluent = petroleum ether / ethyl acetate = 100:10), the product **4d** was obtained as yellow solid (139.91 mg, 95% yield).

^{1}H NMR (400 MHz, $CDCl_3$) δ 7.51 - 7.46 (m, 2H), 7.44 (d, J = 8.6 Hz, 1H), 7.33 (d, J = 8.6 Hz, 1H), 7.14 - 7.07 (m, 2H), 3.96 (hept, J = 6.8 Hz, 2H), 3.64 (q, J = 7.2 Hz, 2H), 1.30 (d, J = 6.8 Hz, 12H), 1.20 (t, J = 7.2 Hz, 3H). **^{13}C NMR** (100 MHz, $CDCl_3$) δ 167.65, 167.09, 162.67（d, $^1J_{C-F}$ = 248.46 Hz）,

135.46, 132.93 (d, $^4J_{C-F}$ = 3.3 Hz), 131.98, 131.23 (d, $^3J_{C-F}$ = 8.3 Hz), 130.48, 129.80, 122.28, 114.80 (d, $^2J_{C-F}$ = 21.21 Hz), 50.95, 32.63, 22.64, 13.91.

^{19}F NMR (377 MHz, $CDCl_3$) δ -114.19.

HRMS (ESI) Calcd. for $C_{22}H_{26}N_2O_2F$ ([M+H]$^+$): 369.1973, found: 369.1969.

4-(7-(Diisopropylamino)-2-ethyl-1,3-dioxoisoindolin-4-yl)benzonitrile (4e)

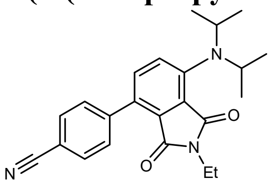

4e

The title product was prepared according to the **General Procedure A**: Carried out with 4-butyrylbenzonitrile (138.47 mg, 0.8 mmol, 2.0 equiv.), diisopropylamine (40.48 mg, 0.4 mmol), $Cu(OAc)_2$ (7.26 mg, 0.04 mmol, 10 mol%), 2,2'-bipyridine (6.25 mg, 0.04 mmol, 10 mol%), N-ethyl maleimide (75.08 mg, 0.6 mmol, 1.5 equiv.), TEMPO (187.50 mg, 1.2 mmol, 3.0 equiv.), p-Toluenesulfonic acid (6.88 mg, 0.04 mmol, 10 mol%, 12 wt.% solution in pure acetic acid) in toluene (1.5 mL) at 120 °C for 48 h. After concentrated and purified by flash column chromatography on silica gel (eluent = petroleum ether / ethyl acetate = 100:15), the product **4e** was obtained as yellow solid (87.04 mg, 58% yield).

^{1}H NMR (400 MHz, $CDCl_3$) δ 7.70 (d, *J* = 8.2 Hz, 2H), 7.63 - 7.56 (d, *J* = 8.2 Hz, 2H), 7.44 (d, *J* = 8.7 Hz, 1H), 7.31 (d, *J* = 8.7 Hz, 1H), 4.01 (p, *J* = 6.7 Hz, 2H), 3.63 (q, *J* = 7.2 Hz, 2H), 1.34 (d, *J* = 6.7 Hz, 13H), 1.20 (t, *J* = 7.2 Hz, 3H).

^{13}C NMR (100 MHz, $CDCl_3$) δ 167.51, 167.09, 148.07, 141.92, 135.01, 131.65, 130.52, 130.37, 129.73, 129.10, 120.51, 119.07, 111.45, 51.23, 32.80, 22.75, 13.95.

HRMS (ESI) Calcd. for $C_{23}H_{26}N_3O_2$ ([M+H]$^+$): 376.2020, found: 376.2017.

4-(Benzo[*d*][1,3]dioxol-5-yl)-7-(diisopropylamino)-2-ethylisoindoline-1,3-dione (4f)

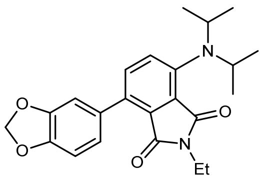

4f

The title product was prepared according to the **General Procedure A**: Carried out with 1-(benzo[*d*][1,3]dioxol-5-yl)butan-1-one (153.67 mg, 0.8 mmol, 2.0 equiv.), diisopropylamine (40.48 mg, 0.4 mmol), $Cu(OAc)_2$ (7.26 mg, 0.04 mmol, 10 mol%), 2,2'-bipyridine (6.25 mg, 0.04 mmol, 10 mol%), N-ethyl maleimide (75.08 mg, 0.6 mmol, 1.5 equiv.), TEMPO (187.50 mg, 1.2 mmol, 3.0 equiv.), p-Toluenesulfonic acid (6.88 mg, 0.04 mmol, 10 mol%, 12 wt.% solution in pure acetic acid) in toluene

(1.5 mL) at 120 °C for 48 h. After concentrated and purified by flash column chromatography on silica gel (eluent = petroleum ether / ethyl acetate = 100:10), the product **4f** was obtained as yellow solid (145.06 mg, 92% yield).

^{1}H NMR (400 MHz, $CDCl_3$) δ 7.40 (d, *J* = 8.6 Hz, 1H), 7.32 (d, *J* = 8.6 Hz, 1H), 6.99 (d, *J* = 1.6 Hz, 1H), 6.96 (dd, *J* = 8.0, 1.6 Hz, 1H), 6.85 (d, *J* = 8.0 Hz, 1H), 5.98 (s, 2H), 3.93 (m, 2H), 3.63 (q, *J* = 7.2 Hz, 2H), 1.27 (d, *J* = 6.8 Hz, 12H), 1.20 (t, *J* = 7.2 Hz, 3H).

^{13}C NMR (100 MHz, $CDCl_3$) δ 167.59, 167.07, 147.49, 147.17, 146.93, 135.50, 132.99, 130.77, 130.69, 129.56, 123.14, 122.67, 110.18, 107.85, 101.19, 50.87, 32.59, 22.60, 22.58, 13.90.

HRMS (ESI) Calcd. for $C_{23}H_{27}N_2O_4$ ($[M+H]^+$): 395.1965, found: 395.1961.

4-(2,4-Dichlorophenyl)-7-(diisopropylamino)-2-ethylisoindoline-1,3-dione (4g)

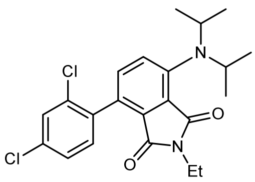

4g

The title product was prepared according to the **General Procedure A**: Carried out with 1-(2,4-dichlorophenyl)butan-1-one (172.81 mg, 0.8 mmol, 2.0 equiv.), diisopropylamine (40.48 mg, 0.4 mmol), $Cu(OAc)_2$ (7.26 mg, 0.04 mmol, 10 mol%), 2,2'-bipyridine (6.25 mg, 0.04 mmol, 10 mol%), N-ethyl maleimide (75.08 mg, 0.6 mmol, 1.5 equiv.), TEMPO (187.50 mg, 1.2 mmol, 3.0 equiv.), p-Toluenesulfonic acid (6.88 mg, 0.04 mmol, 10 mol%, 12 wt.% solution in pure acetic acid) in toluene (1.5 mL) at 120 °C for 48 h. After concentrated and purified by flash column chromatography on silica gel (eluent = petroleum ether / ethyl acetate = 100:10), the product **4g** was obtained as yellow solid (148.85 mg, 89% yield).

^{1}H NMR (400 MHz, $CDCl_3$) δ 7.48 (d, *J* = 2.0 Hz, 1H), 7.40 (d, *J* = 8.7 Hz, 1H), 7.30 (dd, *J* = 8.3, 2.0 Hz, 1H), 7.24 (dd, *J* = 8.5 Hz, 2H), 4.03 (p, *J* = 6.8 Hz, 2H), 3.61 (q, *J* = 7.2 Hz, 2H), 1.34 (d, *J* = 6.8 Hz, 12H), 1.19 (t, *J* = 7.2 Hz, 3H).

^{13}C NMR (100 MHz, $CDCl_3$) δ 167.38, 167.26, 147.87, 135.34, 135.02, 134.38, 134.34, 132.05, 131.62, 129.25, 128.42, 126.98, 126.84, 119.76, 51.06, 32.71, 22.83, 22.67, 13.98.

HRMS (ESI) Calcd. for $C_{22}H_{25}N_2O_2Cl_2$ ($[M+H]^+$): 419.1288, found: 419.1286.

7-(Diisopropylamino)-2-ethyl-4,5-diphenylisoindoline-1,3-dione (4h)

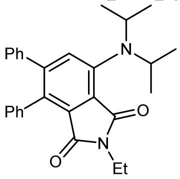

4h

The title product was prepared according to the **General Procedure A**: Carried out with 1,2-diphenylbutan-1-one (179.30 mg, 0.8 mmol, 2.0 equiv.), diisopropylamine (40.48 mg, 0.4 mmol), $Cu(OAc)_2$ (7.26 mg, 0.04 mmol, 10 mol%), 2,2'-bipyridine (6.25 mg, 0.04 mmol, 10 mol%), N-ethyl maleimide (75.08 mg, 0.6 mmol, 1.5 equiv.), TEMPO (187.50 mg, 1.2 mmol, 3.0 equiv.), p-Toluenesulfonic acid (6.88 mg, 0.04 mmol, 10 mol%, 12 wt.% solution in pure acetic acid) in toluene (1.5 mL) at 120 °C for 48 h. After concentrated and purified by flash column chromatography on silica gel (eluent = petroleum ether / ethyl acetate = 100:10), the product **4h** was obtained as yellow solid (136.39 mg, 80% yield).

^{1}H NMR (400 MHz, $CDCl_3$) δ 7.46 (s, 1H), 7.27 - 7.22 (m, 3H), 7.22 - 7.17 (m, 3H), 7.16 - 7.11 (m, 2H), 7.10 - 7.04 (m, 2H), 4.03 (hept, *J* = 6.8 Hz, 2H), 3.63 (q, *J* = 7.2 Hz, 2H), 1.35 (d, *J* = 6.8 Hz, 12H), 1.21 (t, *J* = 7.2 Hz, 3H).

^{13}C NMR (100 MHz, $CDCl_3$) δ 167.51, 167.08, 147.46, 146.89, 139.91, 135.66, 132.09, 131.77, 131.43, 130.61, 129.62, 127.92, 127.40, 127.24, 127.23, 121.20, 50.94, 32.66, 22.80, 13.94.

HRMS (ESI) Calcd. for $C_{28}H_{31}N_2O_2$ ($[M+H]^+$): 427.2380, found: 427.2379.

Ethyl 7-(diisopropylamino)-2-ethyl-1,3-dioxo-4-phenylisoindoline-5-carboxylate (4i)

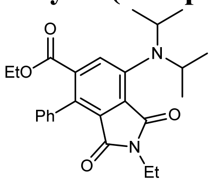

4i

The title product was prepared according to the **General Procedure A**: Carried out with ethyl 2-benzoylbutanoate (176.09 mg, 0.8 mmol, 2.0 equiv.), diisopropylamine (40.48 mg, 0.4 mmol), $Cu(OAc)_2$ (7.26 mg, 0.04 mmol, 10 mol%), 2,2'-bipyridine (6.25 mg, 0.04 mmol, 10 mol%), N-ethyl maleimide (75.08 mg, 0.6 mmol, 1.5 equiv.), TEMPO (187.50 mg, 1.2 mmol, 3.0 equiv.), p-Toluenesulfonic acid (6.88 mg, 0.04 mmol, 10 mol%, 12 wt.% solution in pure acetic acid) in toluene (1.5 mL) at 120 °C for 48 h. After concentrated and purified by flash column chromatography on silica gel (eluent = petroleum ether / ethyl acetate = 100:10), the product **4i** was obtained as yellow solid (152.01mg, 90% yield).

^{1}H NMR (400 MHz, $CDCl_3$) δ 7.80 (s, 1H), 7.43 - 7.34 (m, 3H), 7.27 (dd, *J* = 6.7, 2.9 Hz, 2H), 4.05 - 3.95 (m, 4H), 3.59 (q, *J* = 7.2 Hz, 2H), 1.34 (d, *J* = 6.8 Hz, 12H), 1.17 (t, *J* = 7.2 Hz, 3H), 0.88 (t, *J* = 7.2 Hz, 3H).

^{13}C NMR (100 MHz, $CDCl_3$) δ 167.47, 166.65, 146.71, 137.63, 136.01, 131.62, 131.01, 130.21, 128.99, 127.59, 127.49, 122.46, 61.35, 51.09, 32.72, 22.63, 13.78, 13.44.

HRMS (ESI) Calcd. for $C_{25}H_{31}N_2O_4$ ($[M+H]^+$): 423.2278, found: 423.2276.

5-Benzoyl-7-(diisopropylamino)-2-ethyl-4-phenylisoindoline-1,3-dione (4j)

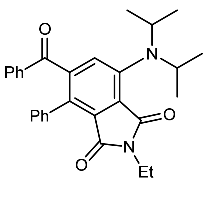

4j

The title product was prepared according to the **General Procedure A**: Carried out with 2-ethyl-1,3-diphenylpropane-1,3-dione (201.70 mg, 0.8 mmol, 2.0 equiv.), diisopropylamine (40.48 mg, 0.4 mmol), $Cu(OAc)_2$ (7.26 mg, 0.04 mmol, 10 mol%), 2,2'-bipyridine (6.25 mg, 0.04 mmol, 10 mol%), N-ethyl maleimide (75.08 mg, 0.6 mmol, 1.5 equiv.), TEMPO (187.50 mg, 1.2 mmol, 3.0 equiv.), p-Toluenesulfonic acid (6.88 mg, 0.04 mmol, 10 mol%, 12 wt.% solution in pure acetic acid) in toluene (1.5 mL) at 120 ºC for 48 h. After concentrated and purified by flash column chromatography on silica gel (eluent = petroleum ether / ethyl acetate = 100:10), the product **4j** was obtained as yellow solid (172.61 mg, 95% yield).

^{1}H NMR (400 MHz, $CDCl_3$) δ 7.66 - 7.60 (m, 2H), 7.49 - 7.41 (m, 2H), 7.31 (t, J = 7.6 Hz, 2H), 7.24 - 7.14 (m, 5H), 4.03 (p, J = 6.7 Hz, 2H), 3.65 (q, J = 7.1 Hz, 2H), 1.32 (d, J = 6.8 Hz, 12H), 1.22 (t, J = 7.2 Hz, 3H).

^{13}C NMR (100 MHz, $CDCl_3$) δ 196.94, 166.86, 166.73, 146.73, 145.56, 136.73, 134.42, 133.42, 131.32, 130.22, 129.92, 129.65, 128.38, 128.31, 127.87, 127.53, 122.00, 77.48, 77.16, 76.84, 51.15, 32.79, 22.68, 13.87.

HRMS (ESI) Calcd. for $C_{29}H_{31}N_2O_3$ ($[M+H]^+$): 455.2329, found: 455.2325.

5-Acetyl-7-(diisopropylamino)-2-ethyl-4-phenylisoindoline-1,3-dione (4k-1)

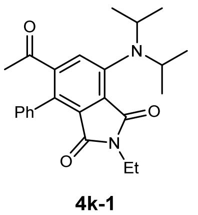

4k-1

The title product was prepared according to the **General Procedure A**: Carried out with 2-ethyl-1-phenylbutane-1,3-dione (152.08 mg, 0.8 mmol, 2.0 equiv.), diisopropylamine (40.48 mg, 0.4 mmol), $Cu(OAc)_2$ (7.26 mg, 0.04 mmol, 10 mol%), 2,2'-bipyridine (6.25 mg, 0.04 mmol, 10 mol%), N-ethyl maleimide (75.08 mg, 0.6 mmol, 1.5 equiv.), TEMPO (187.50 mg, 1.2 mmol, 3.0 equiv.), p-Toluenesulfonic acid (6.88 mg, 0.04 mmol, 10 mol%, 12 wt.% solution in pure acetic acid) in toluene (1.5 mL) at 120 ºC for 48 h. After concentrated and purified by flash column chromatography on silica gel (eluent = petroleum ether / ethyl acetate = 100:10), the product **4k-1** was obtained as yellow solid (40.07 mg, 30% yield).

^{1}H NMR (400 MHz, $CDCl_3$) δ 7.81 (dd, *J* = 8.0, 1.4 Hz, 2H), 7.65 - 7.59 (m, 1H), 7.48 (t, *J* = 8.0 Hz, 2H), 7.28 (s, 1H), 3.85 (m, 2H), 3.69 (q, *J* = 7.2 Hz, 2H), 2.51 (s, 3H), 1.25 (t, *J* = 7.2 Hz, 3H), 1.18 (d, *J* = 6.8 Hz, 12H).

^{13}C NMR (100 MHz, $CDCl_3$) δ 197.09, 168.49, 166.75, 145.47, 145.29, 136.66, 134.12, 131.63, 130.40, 130.06, 128.91, 128.51, 124.43, 50.77, 32.76, 22.53, 14.38, 14.03.

HRMS (ESI) Calcd. for $C_{24}H_{29}N_2O_3$ ($[M+H]^+$): 393.2173, found: 393.2168.

5-Benzoyl-7-(diisopropylamino)-2-ethyl-4-methylisoindoline-1,3-dione (4k-2)

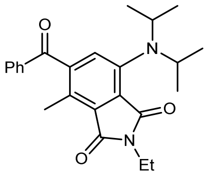

4k-2

The title product was prepared according to the **General Procedure A**: Carried out with 2-ethyl-1-phenylbutane-1,3-dione (152.08 mg, 0.8 mmol, 2.0 equiv.), diisopropylamine (40.48 mg, 0.4 mmol), $Cu(OAc)_2$ (7.26 mg, 0.04 mmol, 10 mol%), 2,2'-bipyridine (6.25 mg, 0.04 mmol, 10 mol%), N-ethyl maleimide (75.08 mg, 0.6 mmol, 1.5 equiv.), TEMPO (187.50 mg, 1.2 mmol, 3.0 equiv.), p-Toluenesulfonic acid (6.88 mg, 0.04 mmol, 10 mol%, 12 wt.% solution in pure acetic acid) in toluene (1.5 mL) at 120 ºC for 48 h. After concentrated and purified by flash column chromatography on silica gel (eluent = petroleum ether / ethyl acetate = 100:10), the product **4k-2** was obtained as yellow solid (73.74 mg, 47% yield).

^{1}H NMR (400 MHz, $CDCl_3$) δ 7.47 (s, 1H), 7.46 - 7.40 (m, 3H), 7.33 (dd, *J* = 6.5, 2.9 Hz, 2H), 4.01 (m, 2H), 3.61 (q, *J* = 7.2 Hz, 2H), 1.86 (s, 3H), 1.34 (d, *J* = 6.8 Hz, 12H), 1.18 (t, *J* = 7.2 Hz, 3H).

^{13}C NMR (100 MHz, $CDCl_3$) δ 203.72, 166.97, 166.71, 147.24, 147.04, 135.24, 131.40, 129.88, 129.17, 128.57, 128.23, 128.10, 122.00, 51.27, 32.87, 30.56, 22.77, 13.92.

HRMS (ESI) Calcd. for $C_{24}H_{29}N_2O_3$ ($[M+H]^+$): 393.2173, found: 393.2167.

Ethyl 7-(diisopropylamino)-2-ethyl-4-(4-nitrophenyl)-1,3-dioxoisoindoline-5-carboxylate (4l)

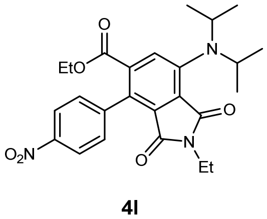

4l

The title product was prepared according to the **General Procedure A**: Carried out with ethyl 2-(4-nitrobenzoyl)butanoate (212.08 mg, 0.8 mmol, 2.0 equiv.), diisopropylamine (40.48 mg, 0.4 mmol), $Cu(OAc)_2$ (7.26 mg, 0.04 mmol, 10 mol%), 2,2'-bipyridine (6.25 mg, 0.04 mmol, 10 mol%), N-ethyl

maleimide (75.08 mg, 0.6 mmol, 1.5 equiv.), TEMPO (187.50 mg, 1.2 mmol, 3.0 equiv.), p-Toluenesulfonic acid (6.88 mg, 0.04 mmol, 10 mol%, 12 wt.% solution in pure acetic acid) in toluene (1.5 mL) at 120 °C for 48 h. After concentrated and purified by flash column chromatography on silica gel (eluent = petroleum ether / ethyl acetate = 100:10), the product **4l** was obtained as yellow solid (134.56 mg, 72% yield).

^{1}H NMR (400 MHz, $CDCl_3$) δ 8.30 - 8.18 (m, 2H), 7.94 (s, 1H), 7.47 - 7.39 (m, 2H), 4.07 (m, 4H), 3.57 (q, J = 7.1 Hz, 2H), 1.39 (d, J = 7.1 Hz, 12H), 1.16 (t, J = 7.1 Hz, 3H), 0.98 (t, J = 7.1 Hz, 3H).

^{13}C NMR (100 MHz, $CDCl_3$) δ 166.57, 166.45, 166.25, 147.38, 147.17, 144.00, 135.74, 132.27, 130.22, 129.43, 127.42, 122.75, 120.26, 61.66, 51.34, 32.90, 22.68, 13.75, 13.65.

HRMS (ESI) Calcd. for $C_{25}H_{30}N_3O_6$ ($[M+H]^+$): 468.2129, found: 468.2125.

Ethyl 7-(diisopropylamino)-2-ethyl-1,3-dioxo-4-(4-(trifluoromethyl)phenyl)isoindoline-5-carboxylate (4m)

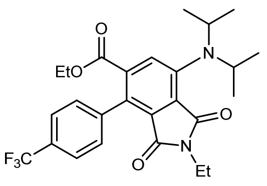

4m

The title product was prepared according to the **General Procedure A**: Carried out with ethyl 2-(4-(trifluoromethyl)benzoyl)butanoate (230.48 mg, 0.8 mmol, 2.0 equiv.), diisopropylamine (40.48 mg, 0.4 mmol), $Cu(OAc)_2$ (7.26 mg, 0.04 mmol, 10 mol%), 2,2'-bipyridine (6.25 mg, 0.04 mmol, 10 mol%), N-ethyl maleimide (75.08 mg, 0.6 mmol, 1.5 equiv.), TEMPO (187.50 mg, 1.2 mmol, 3.0 equiv.), p-Toluenesulfonic acid (6.88 mg, 0.04 mmol, 10 mol%, 12 wt.% solution in pure acetic acid) in toluene (1.5 mL) at 120 °C for 48 h. After concentrated and purified by flash column chromatography on silica gel (eluent = petroleum ether / ethyl acetate = 100:10), the product **4m** was obtained as yellow solid (127.45 mg, 65% yield).

^{1}H NMR (400 MHz,) δ 7.88 (s, 1H), 7.65 (d, J = 8.0 Hz, 2H), 7.39 (d, J = 8.0 Hz, 2H), 4.12 - 3.97 (m, 4H), 3.60 (q, J = 7.2 Hz, 2H), 1.38 (d, J = 6.8 Hz, 12H), 1.18 (t, J = 7.2 Hz, 3H), 0.89 (t, J = 7.2 Hz, 3H).

^{13}C NMR (100 MHz, $CDCl_3$) δ 167.00, 166.76, 166.62, 147.31, 140.41, 136.91, 132.13, 129.98, 129.78 (q, $^2J_{C\text{-}F}$ = 32.4 Hz), 129.65, 124.57 (q, $^3J_{C\text{-}F}$ = 3.91 Hz), 124.41 (q, $^1J_{C\text{-}F}$ = 271.5 Hz), 121.37, 61.66, 51.39, 32.98, 22.80, 13.91, 13.46.

^{19}F NMR (377 MHz, $CDCl_3$) δ -62.28.

HRMS (ESI) Calcd. for $C_{26}H_{30}N_2O_4F_3$ ($[M+H]^+$): 491.2152, found: 491.2148.

4-(Diisopropylamino)-2-ethyl-7-(furan-2-yl)isoindoline-1,3-dione (4n)

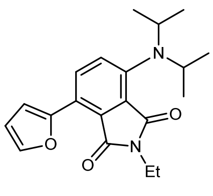

4n

The title product was prepared according to the **General Procedure A**: Carried out with 1-(furan-2-yl)butan-1-one (110.46 mg, 0.8 mmol, 2.0 equiv.), diisopropylamine (40.48 mg, 0.4 mmol), $Cu(OAc)_2$ (7.26 mg, 0.04 mmol, 10 mol%), 2,2'-bipyridine (6.25 mg, 0.04 mmol, 10 mol%), N-ethyl maleimide (75.08 mg, 0.6 mmol, 1.5 equiv.), TEMPO (187.50 mg, 1.2 mmol, 3.0 equiv.), p-Toluenesulfonic acid (6.88 mg, 0.04 mmol, 10 mol%, 12 wt.% solution in pure acetic acid) in toluene (1.5 mL) at 120 °C for 48 h. After concentrated and purified by flash column chromatography on silica gel (eluent = petroleum ether / ethyl acetate = 100:10), the product **4n** was obtained as yellow solid (111.58 mg, 82% yield).

^{1}H NMR (400 MHz, $CDCl_3$) δ 1.25 (dd, *J* = 10.7, 7.0 Hz, 15H), 3.70 (q, *J* = 7.2 Hz, 2H), 3.91 (hept, *J* = 6.7 Hz, 2H), 6.54 (dd, *J* = 3.6, 1.8 Hz, 1H), 7.44 (d, *J* = 8.9 Hz, 1H), 7.50 (d, *J* = 1.7 Hz, 1H), 7.73 (d, *J* = 3.5 Hz, 1H), 7.94 (d, *J* = 9.0 Hz, 1H).

^{13}C NMR (100 MHz, $CDCl_3$) δ 167.81, 167.08, 149.44, 147.04, 142.55, 131.83, 131.13, 127.40, 122.85, 122.15, 113.04, 112.29, 51.08, 32.86, 22.72, 14.10.

HRMS (ESI) Calcd. for $C_{20}H_{25}N_2O_3$ ($[M+H]^+$): 341.1860, found: 341.1852.

4-(Diisopropylamino)-2-ethyl-7-(thiophen-2-yl)isoindoline-1,3-dione （4o）

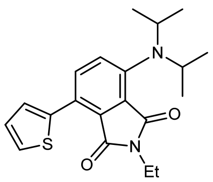

4o

The title compound was prepared according to the general procedure A: Carried out with **2-**butyrylthiophene (123.24 mg, 0.8 mmol, 2 equiv), diisopropylamine (40.48 mg, 0.4 mmol), $Cu(OAc)_2$ (7.26 mg, 0.04 mmol, 10 mol%), 2,2'-bipyridine (6.25 mg, 0.04 mmol, 10 mol%), N-ethyl maleimide (75.08 mg, 0.6 mmol, 1.5 equiv), TEMPO (187.50 mg, 1.2 mmol, 3 equiv), p-Toluenesulfonic acid (6.88 mg, 0.04 mmol, 10 mol%, 12% solution in pure acetic acid) in toluene (1.5 mL) at 120 °C for 48 h. After concentrated and purified by flash chromatography on silica gel (eluent = petroleum ether / ethyl acetate = 100:10), the product **4o** was obtained as yellow solid (131.07 mg, 92% yield).

^{1}H NMR (400 MHz, $CDCl_3$) δ 1.22 (t, *J* = 7.1 Hz, 3H), 1.28 (d, *J* = 6.9 Hz, 12H), 3.67 (q, *J* = 10.4, 7.7 Hz, 2H), 3.94 (p, *J* = 6.8 Hz, 2H), 7.11 (s, 1H), 7.39 (d, *J* = 11.4 Hz, 2H), 7.53 (d, *J* = 8.7 Hz, 1H), 7.61 (s, 1H).

^{13}C NMR (100 MHz, $CDCl_3$) δ 167.54, 167.12, 147.07, 136.95, 135.54, 133.31, 130.69, 129.73, 129.44, 127.89, 127.79, 122.69, 50.87, 32.58, 22.61, 13.89.

HRMS (ESI) Calcd. for $C_{20}H_{25}N_2O_2S$ ($[M+H]^+$): 357.1631, found: 357.1627.

4-(Tert-butyl)-7-(diisopropylamino)-2-ethylisoindoline-1,3-dione (4p)

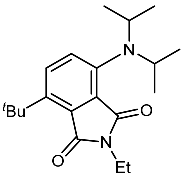

4p

The title product was prepared according to the **General Procedure A**: Carried out with 2,2-dimethylhexan-3-one (102.50 mg, 0.8 mmol, 2.0 equiv.), diisopropylamine (40.48 mg, 0.4 mmol), $Cu(OAc)_2$ (7.26 mg, 0.04 mmol, 10 mol%), 2,2'-bipyridine (6.25 mg, 0.04 mmol, 10 mol%), N-ethyl maleimide (75.08 mg, 0.6 mmol, 1.5 equiv.), TEMPO (187.50 mg, 1.2 mmol, 3.0 equiv.), p-Toluenesulfonic acid (6.88 mg, 0.04 mmol, 10 mol%, 12 wt.% solution in pure acetic acid) in toluene (1.5 mL) at 120 ºC for 48 h. After concentrated and purified by flash column chromatography on silica gel (eluent = petroleum ether / ethyl acetate = 100:10), the product **4p** was obtained as yellow oil (66.05 mg, 50% yield).

^{1}H NMR (400 MHz, $CDCl_3$) δ 7.48 (d, J = 8.8 Hz, 1H), 7.35 (d, J = 8.8 Hz, 1H), 3.74 (p, J = 6.6 Hz, 2H), 3.66 (q, J = 7.2 Hz, 2H), 1.48 (s, 9H), 1.22 (t, J = 7.2 Hz, 3H), 1.14 (d, J = 6.6 Hz, 12H).

^{13}C NMR (100 MHz, $CDCl_3$) δ 168.31, 167.14, 145.93, 145.31, 133.38, 131.69, 130.56, 127.53, 50.55, 35.11, 32.72, 30.12, 22.43, 13.98.

HRMS (ESI) Calcd. for $C_{20}H_{31}N_2O_2$ ($[M+H]^+$): 331.2380, found: 331.2373.

4-(Diisopropylamino)-2-ethyl-7-methylisoindoline-1,3-dione (4q)

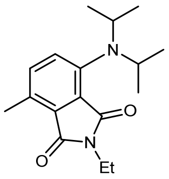

4q

The title product was prepared according to the **General Procedure A**: Carried out with pentan-2-one (68.86 mg, 0.8 mmol, 2.0 equiv.), diisopropylamine (40.48 mg, 0.4 mmol), $Cu(OAc)_2$ (7.26 mg, 0.04 mmol, 10 mol%), 2,2'-bipyridine (6.25 mg, 0.04 mmol, 10 mol%), N-ethyl maleimide (75.08 mg, 0.6 mmol, 1.5 equiv.), TEMPO (187.50 mg, 1.2 mmol, 3.0 equiv.), p-Toluenesulfonic acid (6.88 mg, 0.04 mmol, 10 mol%, 12 wt.% solution in pure acetic acid) in toluene (1.5 mL) at 120 ºC for 48 h. After concentrated and purified by flash column chromatography on silica gel (eluent = petroleum ether / ethyl acetate = 100:8), the product **4q** was obtained as yellow oil (66.86 mg, 58% yield).

^{1}H NMR (400 MHz, $CDCl_3$) δ 7.29 (d, J = 8.4 Hz, 1H), 7.22 (d, J = 8.4 Hz, 1H), 3.82 (p, J = 6.6 Hz, 2H), 3.66 (q, J = 7.2 Hz, 2H), 2.60 (s, 3H), 1.22 (t, J = 7.2 Hz, 3H), 1.16 (d, J = 6.6 Hz, 12H).

^{13}**C NMR** (100 MHz, $CDCl_3$) δ 168.98, 167.36, 145.71, 136.10, 132.54, 131.25, 130.38, 124.57, 50.43, 32.46, 22.41, 17.22, 14.04.

HRMS (ESI) Calcd. for $C_{17}H_{25}N_2O_2$ ($[M+H]^+$): 289.1911, found: 289.1904.

4-(Diisopropylamino)-2-ethyl-7-propylisoindoline-1,3-dione (4r)

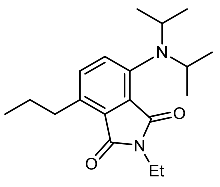

4r

The title product was prepared according to the **General Procedure A**: Carried out with heptan-4-one (91.28 mg, 0.8 mmol, 2.0 equiv.), diisopropylamine (40.48 mg, 0.4 mmol), $Cu(OAc)_2$ (7.26 mg, 0.04 mmol, 10 mol%), 2,2'-bipyridine (6.25 mg, 0.04 mmol, 10 mol%), N-ethyl maleimide (75.08 mg, 0.6 mmol, 1.5 equiv.), TEMPO (187.50 mg, 1.2 mmol, 3.0 equiv.), p-Toluenesulfonic acid (6.88 mg, 0.04 mmol, 10 mol%, 12 wt.% solution in pure acetic acid) in toluene (1.5 mL) at 120 ºC for 48 h. After concentrated and purified by flash column chromatography on silica gel (eluent = petroleum ether / ethyl acetate = 100:8), the product **4r** was obtained as yellow solid (50.59 mg, 40% yield).

^{1}**H NMR** (400 MHz, $CDCl_3$) δ 7.32 (d, *J* = 8.5 Hz, 1H), 7.25 (d, *J* = 8.5 Hz, 1H), 3.84 (p, *J* = 6.7 Hz, 2H), 3.67 (q, *J* = 7.2 Hz, 2H), 3.05 - 2.97 (m, 2H), 1.74 - 1.62 (m, 2H), 1.24 (t, *J* = 7.2 Hz, 3H), 1.19 (d, *J* = 6.7 Hz, 12H), 0.99 (t, *J* = 7.2 Hz, 3H).

^{13}**C NMR** (100 MHz, $CDCl_3$) δ 168.84, 167.43, 145.87, 136.28, 135.28, 132.41, 130.10, 124.51, 50.53, 32.83, 32.52, 24.16, 22.50, 14.17, 14.07.

HRMS (ESI) Calcd. for $C_{19}H_{29}N_2O_2$ ($[M+H]^+$): 317.2224, found: 317.2218.

(E)-4-(diisopropylamino)-2-ethyl-7-styrylisoindoline-1,3-dione （4s）

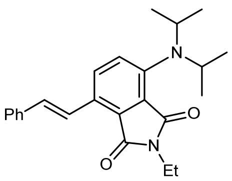

4s

The title product was prepared according to the **General Procedure A**: Carried out with 1-phenylhexan-3-one (140.90 mg, 0.8 mmol, 2.0 equiv.), diisopropylamine (40.48 mg, 0.4 mmol), $Cu(OAc)_2$ (7.26 mg, 0.04 mmol, 10 mol%), 2,2'-bipyridine (6.25 mg, 0.04 mmol, 10 mol%), N-ethyl maleimide (75.08 mg, 0.6 mmol, 1.5 equiv.), TEMPO (187.50 mg, 1.2 mmol, 3.0 equiv.), p-Toluenesulfonic acid (6.88 mg, 0.04 mmol, 10 mol%, 12 wt.% solution in pure acetic acid) in toluene (1.5 mL) at 120 ºC for 48 h. After concentrated and purified by flash column chromatography on silica gel (eluent = petroleum ether / ethyl acetate = 100:10), the product **4s** was obtained as yellow solid (63.20 mg, 42% yield).

^{1}H NMR (400 MHz, $CDCl_3$) δ 8.40 (d, *J* = 16.6 Hz, 1H), 7.79 (d, *J* = 9.0 Hz, 1H), 7.63 - 7.56 (m, 2H), 7.41 - 7.32 (m, 3H), 7.31 - 7.23 (m, 2H), 7.16 (d, *J* = 16.6 Hz, 1H), 3.98 (p, *J* = 6.7 Hz, 2H), 3.70 (q, *J* = 7.2 Hz, 2H), 1.30 (d, *J* = 6.7 Hz, 12H), 1.26 (t, *J* = 7.2 Hz, 3H).
^{13}C NMR (100 MHz, $CDCl_3$) δ 169.00, 167.37, 147.27, 137.27, 130.74, 129.98, 129.61, 129.02, 128.81, 128.49, 128.09, 127.02, 122.80, 120.45, 50.99, 32.66, 22.77, 14.10.
HRMS (ESI) Calcd. for $C_{24}H_{29}N_2O_2$ ($[M+H]^+$): 377.2224, found: 377.2219.

Ethyl 7-(diisopropylamino)-2-ethyl-1,3-dioxoisoindoline-4-carboxylate (4t)

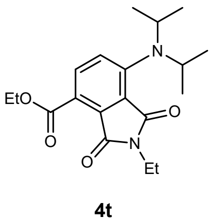

4t

The title product was prepared according to the **General Procedure A**: Carried out with ethyl 2-oxopentanoate (115.26 mg, 0.8 mmol, 2.0 equiv.), diisopropylamine (40.48 mg, 0.4 mmol), $Cu(OAc)_2$ (7.26 mg, 0.04 mmol, 10 mol%), 2,2'-bipyridine (6.25 mg, 0.04 mmol, 10 mol%), N-ethyl maleimide (75.08 mg, 0.6 mmol, 1.5 equiv.), TEMPO (187.50 mg, 1.2 mmol, 3.0 equiv.), p-Toluenesulfonic acid (6.88 mg, 0.04 mmol, 10 mol%, 12 wt.% solution in pure acetic acid) in toluene (1.5 mL) at 120 ºC for 48 h. After concentrated and purified by flash column chromatography on silica gel (eluent = petroleum ether / ethyl acetate = 100:10), the product **4t** was obtained as yellow oil (27.70 mg, 20% yield).
^{1}H NMR (400 MHz, $CDCl_3$) δ 7.59 (d, *J* = 8.8 Hz, 1H), 7.31 (d, *J* = 8.8 Hz, 1H), 4.42 (q, *J* = 7.1 Hz, 2H), 4.03 (p, *J* = 6.7 Hz, 2H), 3.66 (q, *J* = 7.2 Hz, 2H), 1.40 (t, *J* = 7.2 Hz, 3H), 1.33 (d, *J* = 6.7 Hz, 12H), 1.23 (t, *J* = 7.2 Hz, 3H). **^{13}C NMR** (100 MHz, $CDCl_3$) δ 167.12, 166.60, 166.05, 149.56, 133.44, 133.17, 126.48, 120.39, 118.65, 61.85, 51.39, 32.96, 22.64, 14.25, 14.00.
HRMS (ESI) Calcd. for $C_{19}H_{27}N_2O_4$ ($[M+H]^+$): 347.1965, found: 347.1960.

Ethyl 7-(diisopropylamino)-2-ethyl-4-methyl-1,3-dioxoisoindoline-5-carboxylate (4u)

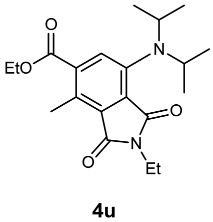

4u

The title product was prepared according to the **General Procedure A**: Carried out with ethyl 2-ethyl-3-oxobutanoate (126.47 mg, 0.8 mmol, 2.0 equiv.), diisopropylamine (40.48 mg, 0.4 mmol), $Cu(OAc)_2$ (7.26 mg, 0.04 mmol, 10 mol%), 2,2'-bipyridine (6.25 mg, 0.04 mmol, 10 mol%), N-ethyl maleimide (75.08 mg, 0.6 mmol, 1.5 equiv.), TEMPO (187.50 mg, 1.2 mmol, 3.0 equiv.), p-Toluenesulfonic acid (6.88 mg, 0.04 mmol, 10 mol%, 12 wt.% solution in pure acetic acid) in toluene (1.5 mL) at 120 ºC for

48 h. After concentrated and purified by flash column chromatography on silica gel (eluent = petroleum ether / ethyl acetate = 100:10), the product **4u** was obtained as yellow solid (100.86 mg, 70% yield).

^{1}H NMR (400 MHz, $CDCl_3$) δ 7.84 (s, 1H), 4.38 (q, *J* = 7.2 Hz, 2H), 3.85 (p, *J* = 6.6 Hz, 2H), 3.67 (q, *J* = 7.2 Hz, 2H), 2.81 (s, 3H), 1.40 (t, *J* = 7.2 Hz, 3H), 1.25 - 1.17 (m, 15H).

^{13}C NMR (100 MHz, $CDCl_3$) δ 168.52, 167.05, 166.52, 145.12, 136.86, 133.51, 131.66, 131.47, 125.83, 61.63, 50.73, 32.78, 22.54, 14.45, 14.35, 13.99.

HRMS (ESI) Calcd. for $C_{20}H_{29}N_2O_4$ ($[M+H]^+$): 361.2122, found: 361.2117.

5-Acetyl-7-(diisopropylamino)-2-ethyl-4-methylisoindoline-1,3-dione (4v)

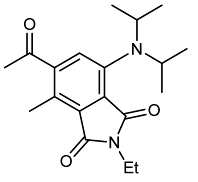

4v

The title product was prepared according to the **General Procedure A**: Carried out with 3-ethylpentane-2,4-dione (102.46 mg, 0.8 mmol, 2.0 equiv.), diisopropylamine (40.48 mg, 0.4 mmol), $Cu(OAc)_2$ (7.26 mg, 0.04 mmol, 10 mol%), 2,2'-bipyridine (6.25 mg, 0.04 mmol, 10 mol%), N-ethyl maleimide (75.08 mg, 0.6 mmol, 1.5 equiv.), TEMPO (187.50 mg, 1.2 mmol, 3.0 equiv.), p-Toluenesulfonic acid (6.88 mg, 0.04 mmol, 10 mol%, 12 wt.% solution in pure acetic acid) in toluene (1.5 mL) at 120 °C for 48 h. After concentrated and purified by flash column chromatography on silica gel (eluent = petroleum ether / ethyl acetate = 100:10), the product **4v** was obtained as yellow solid (59.43 mg, 45% yield).

^{1}H NMR (400 MHz, $CDCl_3$) δ 7.49 (s, 1H), 3.86 (p, *J* = 6.7 Hz, 2H), 3.66 (q, *J* = 6.8 Hz, 2H), 2.69 (s, 3H), 2.55 (s, 3H), 1.35 - 1.07 (m, 15H).

^{13}C NMR (100 MHz, $CDCl_3$) δ 202.11, 168.45, 166.51, 145.36, 145.23, 131.92, 131.07, 129.24, 125.45, 50.73, 32.81, 30.58, 22.63, 14.32, 14.00.

HRMS (ESI) Calcd. for $C_{19}H_{27}N_2O_3$ ($[M+H]^+$): 331.2016, found: 331.2011.

7-(Diisopropylamino)-2-ethyl-5-(4-methoxyphenyl)-4-methylisoindoline-1,3-dione (4w)

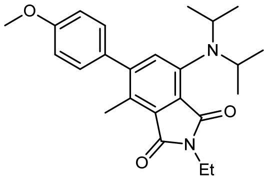

4w

The title product was prepared according to the **General Procedure A**: Carried out with 3-(4-methoxyphenyl)pentan-2-one (153.70 mg, 0.8 mmol, 2.0 equiv.), diisopropylamine (40.48 mg, 0.4 mmol), $Cu(OAc)_2$ (7.26 mg, 0.04 mmol, 10 mol%), 2,2'-bipyridine (6.25 mg, 0.04 mmol, 10 mol%), N-ethyl maleimide (75.08 mg, 0.6 mmol, 1.5 equiv.), TEMPO (187.50 mg, 1.2 mmol, 3.0 equiv.), p-Toluenesulfonic acid (6.88 mg, 0.04 mmol, 10 mol%, 12 wt.% solution in pure acetic acid) in toluene

(1.5 mL) at 120 ºC for 48 h. After concentrated and purified by flash column chromatography on silica gel (eluent = petroleum ether / ethyl acetate = 100:10), the product **4w** was obtained as yellow solid (66.23 mg, 42% yield).

^{1}H NMR (400 MHz,) δ 7.27 (s, 1H), 7.24 (d, *J* = 8.3 Hz, 2H), 6.98 (d, *J* = 8.3 Hz, 2H), 3.92 - 3.81 (m, 5H), 3.69 (q, *J* = 7.1 Hz, 2H), 2.54 (s, 3H), 1.24 (t, *J* = 7.1 Hz, 3H), 1.19 (d, *J* = 7.1 Hz, 12H).

^{13}C NMR (100 MHz, $CDCl_3$) δ 169.28, 167.17, 159.29, 148.50, 145.21, 133.83, 132.40, 131.22, 130.38, 129.71, 123.38, 113.87, 55.45, 50.53, 32.60, 22.55, 15.08, 14.10.

HRMS (ESI) Calcd. for $C_{24}H_{31}N_2O_3$ ($[M+H]^+$): 395.2329, found: 395.2322.

4-(Diisopropylamino)-2-ethyl-6-phenylisoindoline-1,3-dione (4x)

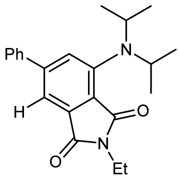

4x

The title product was prepared according to the **General Procedure A**: Carried out with 2-phenylbutanal (118.47 mg, 0.8 mmol, 2.0 equiv.), diisopropylamine (40.48 mg, 0.4 mmol), $Cu(OAc)_2$ (7.26 mg, 0.04 mmol, 10 mol%), 2,2'-bipyridine (6.25 mg, 0.04 mmol, 10 mol%), N-ethyl maleimide (75.08 mg, 0.6 mmol, 1.5 equiv.), TEMPO (187.50 mg, 1.2 mmol, 3.0 equiv.), p-Toluenesulfonic acid (6.88 mg, 0.04 mmol, 10 mol%, 12 wt.% solution in pure acetic acid) in toluene (1.5 mL) at 120 ºC for 48 h. After concentrated and purified by flash column chromatography on silica gel (eluent = petroleum ether / ethyl acetate = 100:10), the product **4x** was obtained as yellow solid (63.04 mg, 45% yield).

^{1}H NMR (400 MHz, $CDCl_3$) δ 7.61 (d, *J* = 7.4 Hz, 2H), 7.57 (d, *J* = 2.9 Hz, 2H), 7.47 (t, *J* = 6.1 Hz, 2H), 7.41 (t, *J* = 7.4 Hz, 1H), 4.07 (hept, *J* = 6.6 Hz, 2H), 3.71 (q, *J* = 7.2 Hz, 2H), 1.34 (d, *J* = 6.6 Hz, 12H), 1.26 (t, *J* = 7.2 Hz, 3H), 1.18 (d, *J* = 6.6 Hz, 2H).

^{13}C NMR (100 MHz, $CDCl_3$) δ 168.32, 167.73, 148.05, 146.40, 139.72, 136.07, 129.15, 128.63, 127.48, 127.19, 118.58, 113.81, 50.80, 32.81, 22.74, 14.11.

HRMS (ESI) Calcd. for $C_{22}H_{27}N_2O_2$ ($[M+H]^+$): 351.2067, found: 351.2061.

2-Ethyl-4-(isopropyl(phenyl)amino)-7-phenylisoindoline-1,3-dione (5a)

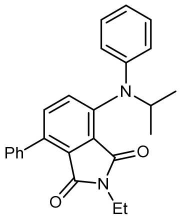

5a

The title product was prepared according to the **General Procedure A**: Carried out with 1-phenylbutan-1-one (118.47 mg, 0.8 mmol, 2.0 equiv.), N-isopropylaniline (54.04 mg, 0.4 mmol), $Cu(OAc)_2$ (7.26 mg,

0.04 mmol, 10 mol%), 2,2'-bipyridine (6.25 mg, 0.04 mmol, 10 mol%), N-ethyl maleimide (75.08 mg, 0.6 mmol, 1.5 equiv.), TEMPO (187.50 mg, 1.2 mmol, 3.0 equiv.), p-Toluenesulfonic acid (6.88 mg, 0.04 mmol, 10 mol%, 12 wt.% solution in pure acetic acid) in toluene (1.5 mL) at 120 °C for 48 h. After concentrated and purified by flash column chromatography on silica gel (eluent = petroleum ether / ethyl acetate = 100:10), the product **5a** was obtained as yellow solid (153.67 mg, 70% yield).

^{1}H NMR (400 MHz, $CDCl_3$) δ 7.55 (dd, *J* = 7.8, 1.6 Hz, 2H), 7.51 - 7.42 (m, 4H), 7.30 (d, *J* = 8.5 Hz, 1H), 7.28 - 7.26 (m, 1H), 7.26 - 7.24 (m, 1H), 6.95 (t, *J* = 7.5 Hz, 1H), 6.91 (d, *J* = 7.8 Hz, 2H), 4.59 (hept, *J* = 6.7 Hz, 1H), 3.62 (q, *J* = 6.9 Hz, 2H), 1.33 (d, *J* = 6.7 Hz, 6H), 1.18 (t, *J* = 6.9 Hz, 3H).

^{13}C NMR (100 MHz, $CDCl_3$) δ 167.48, 166.27, 146.62, 144.14, 136.91, 136.57, 136.28, 134.29, 129.80, 129.57, 129.09, 128.46, 128.07, 125.44, 121.28, 121.22, 52.22, 32.86, 21.65, 13.92.

HRMS (ESI) Calcd. for $C_{25}H_{25}N_2O_2$ ($[M+H]^+$): 385.1911, found: 385.1910.

4-(Cyclohexyl(phenyl)amino)-2-ethyl-7-phenylisoindoline-1,3-dione (5b)

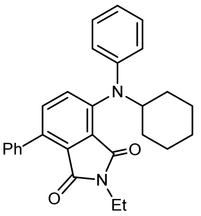

5b

The title product was prepared according to the **General Procedure A**: Carried out with 1-phenylbutan-1-one (118.47 mg, 0.8 mmol, 2.0 equiv.), N-cyclohexylaniline (70.06 mg, 0.4 mmol), $Cu(OAc)_2$ (7.26 mg, 0.04 mmol, 10 mol%), 2,2'-bipyridine (6.25 mg, 0.04 mmol, 10 mol%), N-ethyl maleimide (75.08 mg, 0.6 mmol, 1.5 equiv.), TEMPO (187.50 mg, 1.2 mmol, 3.0 equiv.), p-Toluenesulfonic acid (6.88 mg, 0.04 mmol, 10 mol%, 12 wt.% solution in pure acetic acid) in toluene (1.5 mL) at 120 °C for 48 h. After concentrated and purified by flash column chromatography on silica gel (eluent = petroleum ether / ethyl acetate = 100:10), the product **5b** was obtained as yellow solid (113.69 mg, 67% yield).

^{1}H NMR (400 MHz, $CDCl_3$) δ 7.58 (d, *J* = 7.3 Hz, 2H), 7.56 - 7.42 (m, 4H), 7.34 (d, *J* = 8.3 Hz, 1H), 7.30 - 7.22 (m, 3H), 6.93 (t, *J* = 7.2 Hz, 1H), 6.86 (d, *J* = 8.0 Hz, 2H), 4.19 - 4.08 (m, 1H), 3.63 (q, *J* = 7.2 Hz, 2H), 2.15 (d, *J* = 11.6 Hz, 2H), 1.84 (d, *J* = 12.7 Hz, 2H), 1.69 (d, *J* = 13.0 Hz, 1H), 1.52 - 1.25 (m, 5H), 1.19 (t, *J* = 7.2 Hz, 3H). **^{13}C NMR** (100 MHz, $CDCl_3$) δ 167.34, 166.01, 147.00, 143.25, 136.82, 136.66, 136.44, 135.02, 129.67, 129.48, 128.97, 128.38, 127.97, 126.37, 120.53, 119.86, 59.93, 32.76, 32.28, 26.24, 25.79, 13.80.

HRMS (ESI) Calcd. for $C_{28}H_{29}N_2O_2$ ($[M+H]^+$): 425.2224, found: 425.2225.

4-(Butyl(phenyl)amino)-2-ethyl-7-phenylisoindoline-1,3-dione (5c)

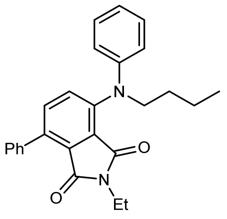

5c

The title product was prepared according to the **General Procedure A**: Carried out with 1-phenylbutan-1-one (118.47 mg, 0.8 mmol, 2.0 equiv.), N-butylaniline (59.35 mg, 0.4 mmol), $Cu(OAc)_2$ (7.26 mg, 0.04 mmol, 10 mol%), 2,2'-bipyridine (6.25 mg, 0.04 mmol, 10 mol%), N-ethyl maleimide (75.08 mg, 0.6 mmol, 1.5 equiv.), TEMPO (187.50 mg, 1.2 mmol, 3.0 equiv.), p-Toluenesulfonic acid (6.88 mg, 0.04 mmol, 10 mol%, 12 wt.% solution in pure acetic acid) in toluene (1.5 mL) at 120 °C for 48 h. After concentrated and purified by flash column chromatography on silica gel (eluent = petroleum ether / ethyl acetate = 100:10), the product **5c** was obtained as yellow solid (82.83 mg, 52% yield).

^{1}H NMR (400 MHz, $CDCl_3$) δ 7.56 (d, *J* = 7.2 Hz, 2H), 7.52 - 7.40 (m, 5H), 7.27 (t, *J* = 7.8 Hz, 2H), 7.04 - 6.88 (m, 3H), 3.92 (t, J = 7.8 Hz, 2H), 3.64 (q, *J* = 7.2 Hz, 2H), 1.73 (p, *J* = 7.8 Hz, 2H), 1.47 - 1.34 (m, 2H), 1.20 (t, *J* = 7.2 Hz, 3H), 0.96 (t, *J* = 7.8 Hz, 3H).

^{13}C NMR (100 MHz, $CDCl_3$) δ 167.44, 166.04, 147.98, 145.07, 137.01, 136.54, 135.88, 132.30, 129.82, 129.51, 129.23, 128.39, 128.04, 123.68, 120.83, 118.49, 53.16, 32.81, 30.04, 20.35, 14.06, 13.89.

HRMS (ESI) Calcd. for $C_{26}H_{27}N_2O_2$ ($[M+H]^+$): 399.2067, found: 399.2064.

2-Ethyl-4-(ethyl(phenyl)amino)-7-phenylisoindoline-1,3-dione (5d)

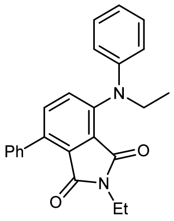

5d

The title product was prepared according to the **General Procedure A**: Carried out with 1-phenylbutan-1-one (118.47 mg, 0.8 mmol, 2.0 equiv.), N-ethylaniline (48.44 mg, 0.4 mmol), $Cu(OAc)_2$ (7.26 mg, 0.04 mmol, 10 mol%), 2,2'-bipyridine (6.25 mg, 0.04 mmol, 10 mol%), N-ethyl maleimide (75.08 mg, 0.6 mmol, 1.5 equiv.), TEMPO (187.50 mg, 1.2 mmol, 3.0 equiv.), p-Toluenesulfonic acid (6.88 mg, 0.04 mmol, 10 mol%, 12 wt.% solution in pure acetic acid) in toluene (1.5 mL) at 120 °C for 48 h. After concentrated and purified by flash column chromatography on silica gel (eluent = petroleum ether / ethyl acetate = 100:10), the product **5d** was obtained as yellow solid (59.23 mg, 40% yield).

^{1}H NMR (400 MHz, $CDCl_3$) δ 7.54 (d, *J* = 7.2 Hz, 2H), 7.51 - 7.36 (m, 5H), 7.33 - 7.23 (m, 3H), 7.03 - 6.91 (m, 3H), 4.00 (q, *J* = 7.2 Hz, 2H), 3.64 (q, *J* = 7.2 Hz, 2H), 1.31 (t, *J* = 7.2 Hz, 3H), 1.20 (t, *J* = 7.2 Hz, 3H).

^{13}C NMR (100 MHz, $CDCl_3$) δ 167.50, 166.28, 147.27, 145.34, 137.00, 136.60, 135.78, 132.11, 129.82, 129.55, 129.34, 128.43, 128.09, 123.38, 121.23, 119.17, 47.81, 32.87, 13.96, 13.28.

HRMS (ESI) Calcd. for $C_{24}H_{23}N_2O_2$ ($[M+H]^+$): 371.1754, found: 371.1747.

2-Ethyl-4-(ethyl(o-tolyl)amino)-7-phenylisoindoline-1,3-dione (5e)

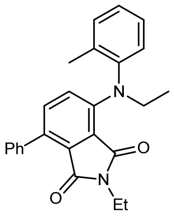

5e

The title product was prepared according to the **General Procedure A**: Carried out with 1-phenylbutan-1-one (118.47 mg, 0.8 mmol, 2.0 equiv.), N-ethyl-2-methylaniline (54.04 mg, 0.4 mmol), $Cu(OAc)_2$ (7.26 mg, 0.04 mmol, 10 mol%), 2,2'-bipyridine (6.25 mg, 0.04 mmol, 10 mol%), N-ethyl maleimide (75.08 mg, 0.6 mmol, 1.5 equiv.), TEMPO (187.50 mg, 1.2 mmol, 3.0 equiv.), p-Toluenesulfonic acid (6.88 mg, 0.04 mmol, 10 mol%, 12 wt.% solution in pure acetic acid) in toluene (1.5 mL) at 120 ºC for 48 h. After concentrated and purified by flash column chromatography on silica gel (eluent = petroleum ether / ethyl acetate = 100:10), the product **5e** was obtained as yellow solid (92.20 mg, 60% yield).

^{1}H NMR (400 MHz, $CDCl_3$) δ 7.49 (d, J = 7.5 Hz, 2H), 7.46 - 7.36 (m, 3H), 7.33 - 7.22 (m, 4H), 7.19 (t, J = 7.5 Hz, 1H), 6.75 (d, J = 8.7 Hz, 1H), 4.02 (q, J = 6.8 Hz, 2H), 3.67 (q, J = 7.1 Hz, 2H), 2.14 (s, 3H), 1.31 (t, J = 6.8 Hz, 3H), 1.23 (t, J = 7.1 Hz, 3H).

^{13}C NMR (100 MHz, $CDCl_3$) δ 167.71, 166.96, 147.27, 144.63, 136.97, 136.65, 136.12, 132.28, 131.85, 129.89, 129.50, 127.98, 127.92, 127.15, 126.17, 126.05, 125.96, 117.31, 50.15, 32.75, 18.53, 14.01, 13.88.

HRMS (ESI) Calcd. for $C_{25}H_{25}N_2O_2$ ($[M+H]^+$): 385.1911, found: 385.1908.

2-Ethyl-4-(methyl(o-tolyl)amino)-7-phenylisoindoline-1,3-dione (5f)

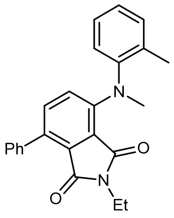

5f

The title product was prepared according to the **General Procedure A**: Carried out with 1-phenylbutan-1-one (118.47 mg, 0.8 mmol, 2.0 equiv.), N,2-dimethylaniline (48.44 mg, 0.4 mmol), $Cu(OAc)_2$ (7.26 mg, 0.04 mmol, 10 mol%), 2,2'-bipyridine (6.25 mg, 0.04 mmol, 10 mol%), N-ethyl maleimide (75.08 mg, 0.6 mmol, 1.5 equiv.), TEMPO (187.50 mg, 1.2 mmol, 3.0 equiv.), p-Toluenesulfonic acid (6.88 mg, 0.04 mmol, 10 mol%, 12 wt.% solution in pure acetic acid) in toluene (1.5 mL) at 120 ºC for 48 h. After concentrated and purified by flash column chromatography on silica gel (eluent = petroleum ether / ethyl acetate = 100:10), the product **5f** was obtained as yellow solid (66.63 mg, 45% yield).

^{1}H NMR (400 MHz, $CDCl_3$) δ 7.52 - 7.48 (m, 2H), 7.46 - 7.37 (m, 3H), 7.32 - 7.24 (m, 3H), 7.23 - 7.16 (m, 2H), 6.82 (d, *J* = 8.7 Hz, 1H), 3.65 (q, *J* = 7.2 Hz, 2H), 3.57 (s, 3H), 2.19 (s, 3H), 1.21 (t, *J* = 7.2 Hz, 3H).

^{13}C NMR (100 MHz, $CDCl_3$) δ 167.74, 166.70, 147.64, 147.07, 136.96, 136.62, 135.23, 132.30, 131.82, 129.91, 129.52, 128.01, 127.95, 127.41, 126.19, 125.60, 125.24, 116.62, 43.80, 32.77, 18.41, 13.98.

HRMS (ESI) Calcd. for $C_{24}H_{23}N_2O_2$ ($[M+H]^+$): 371.1754, found: 371.1750.

2-Ethyl-4-(ethyl(naphthalen-1-yl)amino)-7-phenylisoindoline-1,3-dione (5g)

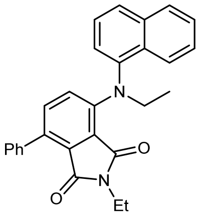

5g

The title product was prepared according to the **General Procedure A**: Carried out with 1-phenylbutan-1-one (118.47 mg, 0.8 mmol, 2.0 equiv.), N-ethylnaphthalen-1-amine (68.44 mg, 0.4 mmol), $Cu(OAc)_2$ (7.26 mg, 0.04 mmol, 10 mol%), 2,2'-bipyridine (6.25 mg, 0.04 mmol, 10 mol%), N-ethyl maleimide (75.08 mg, 0.6 mmol, 1.5 equiv.), TEMPO (187.50 mg, 1.2 mmol, 3.0 equiv.), p-Toluenesulfonic acid (6.88 mg, 0.04 mmol, 10 mol%, 12 wt.% solution in pure acetic acid) in toluene (1.5 mL) at 120 ºC for 48 h. After concentrated and purified by flash column chromatography on silica gel (eluent = petroleum ether / ethyl acetate = 100:10), the product **5g** was obtained as yellow solid (82.36 mg, 49% yield).

^{1}H NMR (400 MHz, $CDCl_3$) δ 7.95 (d, *J* = 8.5 Hz, 1H), 7.90 （d, *J* = 8.5 Hz, 1H） 7.81 (d, *J* = 8.7 Hz, 1H), 7.56 (t, *J* = 7.8 Hz, 1H), 7.53 - 7.34 (m, 8H), 7.14 (d, *J* = 8.7 Hz, 1H), 6.67 (d, *J* = 8.7 Hz, 1H), 4.21 (q, *J* = 7.0 Hz, 2H), 3.72 (q, *J* = 7.2 Hz, 2H), 1.35 (t, *J* = 7.0 Hz, 3H), 1.28 (t, *J* = 7.2 Hz, 3H).

^{13}C NMR (100 MHz, $CDCl_3$) δ 167.85, 167.28, 148.19, 142.44, 137.00, 136.75, 135.36, 132.64, 131.23, 129.91, 129.58, 128.59, 128.11, 128.01, 126.80, 126.77, 126.54, 126.01, 124.08, 123.47, 117.43, 51.17, 32.94, 14.19, 14.17.

HRMS (ESI) Calcd. for $C_{28}H_{25}N_2O_2$ ($[M+H]^+$): 421.1911, found: 421.1913.

4-(Dicyclohexylamino)-2-ethyl-7-phenylisoindoline-1,3-dione (5h)

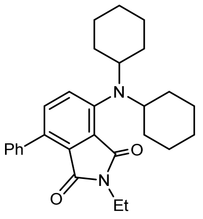

5h

The title product was prepared according to the **General Procedure A**: Carried out with 1-phenylbutan-1-one (118.47 mg, 0.8 mmol, 2.0 equiv.), dicyclohexylamine (72.47 mg, 0.4 mmol), $Cu(OAc)_2$ (7.26 mg,

0.04 mmol, 10 mol%), 2,2'-bipyridine (6.25 mg, 0.04 mmol, 10 mol%), N-ethyl maleimide (75.08 mg, 0.6 mmol, 1.5 equiv.), TEMPO (187.50 mg, 1.2 mmol, 3.0 equiv.), p-Toluenesulfonic acid (6.88 mg, 0.04 mmol, 10 mol%, 12 wt.% solution in pure acetic acid) in toluene (1.5 mL) at 120 °C for 48 h. After concentrated and purified by flash column chromatography on silica gel (eluent = petroleum ether / ethyl acetate = 100:10), the product **5h** was obtained as yellow solid (163.50 mg, 95% yield).

^{1}H NMR (400 MHz, $CDCl_3$) δ 7.57 - 7.48 (m, 3H), 7.48 - 7.37 (m, 4H), 3.66 (q, J = 7.2 Hz, 2H), 3.51 - 3.38 (m, 2H), 2.03 - 1.95 (m, 4H), 1.84 - 1.72 (m, 4H), 1.63 - 1.58 (m, 2H), 1.42 - 1.26 (m, 9H), 1.22 (t, J = 7.2 Hz, 3H), 1.18 - 1.05 (m, 2H).

^{13}C NMR (100 MHz, $CDCl_3$) δ 167.54, 166.87, 147.12, 136.87, 135.44, 135.09, 134.61, 129.47, 129.12, 128.06, 127.83, 126.63, 59.53, 32.94, 32.62, 26.20, 26.10, 13.88.

HRMS (ESI) Calcd. for $C_{28}H_{35}N_2O_2$ ($[M+H]^+$): 431.2693, found: 431.2687.

4-(Di-sec-butylamino)-2-ethyl-7-phenylisoindoline-1,3-dione (5i)

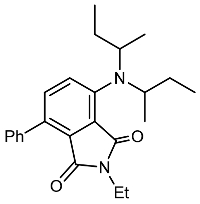

5i

The title product was prepared according to the **General Procedure A**: Carried out with 1-phenylbutan-1-one (118.47 mg, 0.8 mmol, 2.0 equiv.), di-sec-butylamine (51.66 mg, 0.4 mmol), $Cu(OAc)_2$ (7.26 mg, 0.04 mmol, 10 mol%), 2,2'-bipyridine (6.25 mg, 0.04 mmol, 10 mol%), N-ethyl maleimide (75.08 mg, 0.6 mmol, 1.5 equiv.), TEMPO (187.50 mg, 1.2 mmol, 3.0 equiv.), p-Toluenesulfonic acid (6.88 mg, 0.04 mmol, 10 mol%, 12 wt.% solution in pure acetic acid) in toluene (1.5 mL) at 120 °C for 48 h. After concentrated and purified by flash column chromatography on silica gel (eluent = petroleum ether / ethyl acetate = 100:10), the product **5i** was obtained as yellow solid (136.16 mg, 90% yield).

^{1}H NMR (400 MHz, $CDCl_3$) δ 7.47 (d, J = 7.5 Hz, 2H), 7.44 - 7.31 (m, 4H), 7.27 (d, J = 8.5 Hz, 1H), 3.69 - 3.54 (m, 4H), 1.92 - 1.78 (m, 1H), 1.72 - 1.64 (m, 1H), 1.61 - 1.48 (m, 2H), 1.45 (d, J = 6.8 Hz, 3H), 1.32 (d, J = 6.9 Hz, 3H), 1.17 (t, J = 7.4 Hz, 3H), 0.90 (t, J = 7.5 Hz, 3H), 0.81 (t, J = 7.5 Hz, 3H).

^{13}C NMR (100 MHz, $CDCl_3$) δ 167.63, 167.22, 167.14, 148.03, 147.73, 137.09, 135.55, 131.76, 131.44, 130.22, 130.18, 129.47, 128.52, 127.82, 127.76, 119.57, 118.75, 57.95, 57.59, 32.56, 30.01, 29.62, 21.40, 20.60, 13.93, 12.29, 12.07.

HRMS (ESI) Calcd. for $C_{24}H_{31}N_2O_2$ ($[M+H]^+$): 379.2380, found: 379.2373.

4-(Cyclohexyl(isopropyl)amino)-2-ethyl-7-phenylisoindoline-1,3-dione （5j）

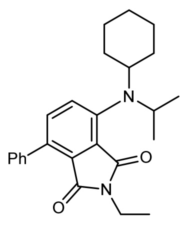

5j

The title product was prepared according to the **General Procedure A**: Carried out with 1-phenylbutan-1-one (118.47 mg, 0.8 mmol, 2.0 equiv.), N-isopropylcyclohexanamine (56.46 mg, 0.4 mmol), $Cu(OAc)_2$ (7.26 mg, 0.04 mmol, 10 mol%), 2,2'-bipyridine (6.25 mg, 0.04 mmol, 10 mol%), N-ethyl maleimide (75.08 mg, 0.6 mmol, 1.5 equiv.), TEMPO (187.50 mg, 1.2 mmol, 3.0 equiv.), p-Toluenesulfonic acid (6.88 mg, 0.04 mmol, 10 mol%, 12 wt.% solution in pure acetic acid) in toluene (1.5 mL) at 120 ºC for 48 h. After concentrated and purified by flash column chromatography on silica gel (eluent = petroleum ether / ethyl acetate = 100:10), the product **5j** was obtained as yellow solid (149.85 mg, 96% yield).

^{1}H NMR (400 MHz, $CDCl_3$) δ 7.56 - 7.50 (m, 2H), 7.50 - 7.37 (m, 5H), 3.93 (hept, *J* = 6.6 Hz, 1H), 3.66 (q, *J* = 7.2 Hz, 2H), 3.54 - 3.42 (m, 1H), 2.08 - 1.96 (m, 2H), 1.85 - 1.74 (m, 2H), 1.62 (dt, *J* = 13.0, 3.4 Hz, 1H), 1.45 (qd, *J* = 11.9, 3.0 Hz, 2H), 1.39 - 1.30 (m, 2H), 1.27 (d, *J* = 6.7 Hz, 6H), 1.22 (t, *J* = 7.2 Hz, 3H), 1.18 - 1.06 (m, 1H).

^{13}C NMR (100 MHz, $CDCl_3$) δ 167.58, 167.02, 147.11, 136.94, 135.50, 134.01, 132.31, 129.48, 127.99, 127.84, 124.25, 60.88, 49.90, 33.05, 32.62, 26.33, 26.08, 22.64, 13.92.

HRMS (ESI) Calcd. for $C_{25}H_{31}N_2O_2$ ($[M+H]^+$): 391.2380, found: 391.2372.

(R)-2-Ethyl-7-(methyl(1-phenylethyl)amino)-4,5-diphenylisoindoline-1,3-dione (5k)

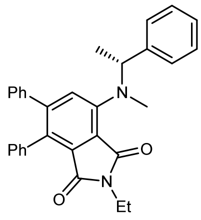

5k

The title product was prepared according to the **General Procedure B:** Carried out with 1,2-diphenylbutan-1-one (89.65 mg, 0.4 mmol, 1.0 equiv.), (R)-N-methyl-1-phenylethan-1-amine (54.04 mg, 0.4 mmol), $Cu(OAc)_2$ (7.26 mg, 0.04 mmol, 10 mol%), 2,2'-bipyridine (6.25 mg, 0.04 mmol, 10 mol%), N-ethyl maleimide (75.08 mg, 0.6 mmol, 1.5 equiv.), TEMPO (187.50 mg, 1.2 mmol, 3.0 equiv.), CsOAc (15.36 mg, 0.08 mmol, 0.2 equiv.) in toluene (1.5 mL) at 120 ºC for 48 h. After concentrated and purified by flash column chromatography on silica gel (eluent = petroleum ether / ethyl acetate = 100:10), the product **5k** was obtained as yellow solid (154.63 mg, 84% yield).

^{1}H NMR (400 MHz, $CDCl_3$) δ 7.41 (d, *J* = 7.5 Hz, 2H), 7.39 - 7.32 (m, 3H), 7.29 (d, *J* = 7.2 Hz, 2H), 7.21 - 7.15 (m, 5H), 7.09 (s, 1H), 7.07 - 7.02 (m, 3H), 5.48 (q, *J* = 6.8 Hz, 1H), 3.67 (q, *J* = 7.2 Hz, 2H), 2.74 (s, 3H), 1.73 (d, *J* = 6.8 Hz, 3H), 1.22 (t, *J* = 7.2 Hz, 3H).

^{13}C NMR (100 MHz, $CDCl_3$) δ 167.73, 167.51, 149.22, 149.03, 141.20, 139.92, 135.66, 134.23, 130.73, 129.71, 128.66, 128.41, 127.92, 127.49, 127.41, 127.31, 127.28, 116.43, 61.54, 33.56, 32.84, 16.43, 14.04.
HRMS (ESI) Calcd. for $C_{31}H_{29}N_2O_2$ ([M+H]$^+$): 461.2224, found: 461.2215.

7-(Diisobutylamino)-2-ethyl-4,5-diphenylisoindoline-1,3-dione (5l)

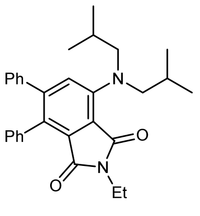

5l

The title product was prepared according to the **General Procedure B:** Carried out with 1,2-diphenylbutan-1-one (89.65 mg, 0.4 mmol, 1.0 equiv.), diisobutylamine (51.66 mg, 0.4 mmol), $Cu(OAc)_2$ (7.26 mg, 0.04 mmol, 10 mol%), 2,2'-bipyridine (6.25 mg, 0.04 mmol, 10 mol%), N-ethyl maleimide (75.08 mg, 0.6 mmol, 1.5 equiv.), TEMPO (187.50 mg, 1.2 mmol, 3.0 equiv.), CsOAc (15.36 mg, 0.08 mmol, 0.2 equiv.) in toluene (1.5 mL) at 120 ºC for 48 h. After concentrated and purified by flash column chromatography on silica gel (eluent = petroleum ether / ethyl acetate = 100:10), the product **5l** was obtained as yellow solid (118.11 mg, 65% yield).
^{1}H NMR (400 MHz, $CDCl_3$) δ 7.40 - 7.28 (m, 7H), 7.28 - 7.15 (m, 4H), 3.77 (q, J = 7.2 Hz, 2H), 3.50 (d, J = 7.2 Hz, 4H), 2.26 - 2.06 (m, 2H), 1.34 (t, J = 7.2 Hz, 3H), 1.03 (d, J = 7.5 Hz, 12H).
^{13}C NMR (100 MHz, $CDCl_3$) δ 166.35, 165.35, 147.11, 147.08, 138.69, 134.37, 130.13, 129.39, 128.31, 128.29, 126.54, 126.02, 125.91, 125.79, 124.96, 114.23, 59.33, 31.36, 25.61, 19.07, 12.62.
HRMS (ESI) Calcd. for $C_{30}H_{35}N_2O_2$ ([M+H]$^+$): 455.2693, found: 455.2686.

Di-tert-butyl 3,3'-((2-ethyl-1,3-dioxo-6,7-diphenylisoindolin-4-yl)azanediyl)dipropionate (5m)

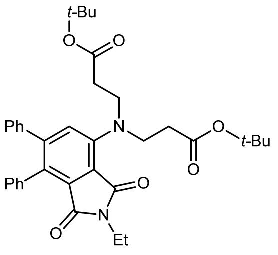

5m

The title product was prepared according to the **General Procedure B:** Carried out with 1,2-diphenylbutan-1-one (89.65 mg, 0.4 mmol, 1.0 equiv.), di-tert-butyl 3,3'-azanediyldipropionate (109.28 mg, 0.4 mmol), $Cu(OAc)_2$ (7.26 mg, 0.04 mmol, 10 mol%), 2,2'-bipyridine (6.25 mg, 0.04 mmol, 10 mol%), N-ethyl maleimide (75.08 mg, 0.6 mmol, 1.5 equiv.), TEMPO (187.50 mg, 1.2 mmol, 3.0 equiv.), CsOAc (15.36 mg, 0.08 mmol, 0.2 equiv.) in toluene (1.5 mL) at 120 ºC for 48 h. After concentrated and purified by flash column chromatography on silica gel (eluent = petroleum ether / ethyl acetate = 100:10), the product **5m** was obtained as yellow solid (95.73 mg, 40% yield).

^{1}H NMR (400 MHz, $CDCl_3$) δ 7.25 - 7.14 (m, 7H), 7.12 - 7.02 (m, 4H), 3.79 (t, J = 7.2 Hz, 4H), 3.62 (q, J = 7.2 Hz, 2H), 2.58 (t, J = 7.2 Hz, 4H), 1.39 (s, 18H), 1.18 (t, J = 7.2 Hz, 3H).
^{13}C NMR (100 MHz, $CDCl_3$) δ 171.24, 167.48, 166.69, 148.78, 146.73, 139.63, 135.45, 131.59, 131.47, 130.57, 129.61, 127.94, 127.48, 127.41, 127.34, 126.57, 118.15, 80.85, 48.58, 34.34, 32.77, 28.08, 13.93.
HRMS (ESI) Calcd. for $C_{36}H_{43}N_2O_6$ ($[M+H]^+$): 599.3116, found: 599.3108.

7-(Bis(2-ethoxyethyl)amino)-2-ethyl-4,5-diphenylisoindoline-1,3-dione （5n）

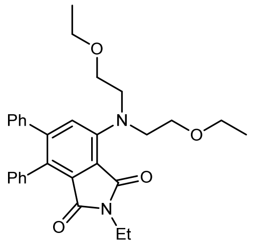

5n

The title product was prepared according to the **General Procedure B:** Carried out with 1,2-diphenylbutan-1-one (89.65 mg, 0.4 mmol, 1.0 equiv.), bis(2-ethoxyethyl)amine (64.46 mg, 0.4 mmol), $Cu(OAc)_2$ (7.26 mg, 0.04 mmol, 10 mol%), 2,2'-bipyridine (6.25 mg, 0.04 mmol, 10 mol%), N-ethyl maleimide (75.08 mg, 0.6 mmol, 1.5 equiv.), TEMPO (187.50 mg, 1.2 mmol, 3.0 equiv.), CsOAc (15.36 mg, 0.08 mmol, 0.2 equiv.) in toluene (1.5 mL) at 120 ºC for 48 h. After concentrated and purified by flash column chromatography on silica gel (eluent = petroleum ether / ethyl acetate = 100:10), the product **5n** was obtained as yellow solid (83.64 mg, 43% yield).
^{1}H NMR (400 MHz, $CDCl_3$) δ 7.33 (s, 1H), 7.25 - 7.21 (m, 3H), 7.20 - 7.18 (m, 3H), 7.09 (dd, J = 7.8, 3.2 Hz, 2H), 7.05 (dd, J = 7.8, 3.2 Hz, 2H), 3.80 (t, J = 5.7 Hz, 4H), 3.72 (t, J = 5.7 Hz, 4H), 3.62 (q, J = 7.1 Hz, 2H), 3.49 (q, J = 7.0 Hz, 4H), 1.21 - 1.14 (m, 9H).
^{13}C NMR (100 MHz, $CDCl_3$) δ 167.62, 167.26, 148.72, 148.08, 139.91, 135.71, 131.39, 130.65, 129.63, 127.89, 127.47, 127.32, 127.26, 126.12, 116.09, 69.15, 66.62, 53.02, 32.77, 15.33, 13.97.
HRMS (ESI) Calcd. for $C_{30}H_{35}N_2O_4$ ($[M+H]^+$): 487.2591, found: 487.2584.

2-Ethyl-7-morpholino-4,5-diphenylisoindoline-1,3-dione （5o）

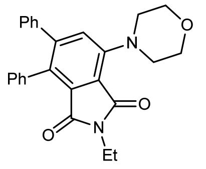

5o

The title product was prepared according to the **General Procedure B:** Carried out with 1,2-diphenylbutan-1-one (89.65 mg, 0.4 mmol, 1.0 equiv.), morpholine (34.83 mg, 0.4 mmol), $Cu(OAc)_2$ (7.26 mg, 0.04 mmol, 10 mol%), 2,2'-bipyridine (6.25 mg, 0.04 mmol, 10 mol%), N-ethyl maleimide (75.08 mg, 0.6 mmol, 1.5 equiv.), TEMPO (187.50 mg, 1.2 mmol, 3.0 equiv.), CsOAc (15.36 mg, 0.08

mmol, 0.2 equiv.) in toluene (1.5 mL) at 120 °C for 48 h. After concentrated and purified by flash column chromatography on silica gel (eluent = petroleum ether / ethyl acetate = 100:15), the product **5o** was obtained as yellow solid (120.36 mg, 73% yield).

^{1}H NMR (400 MHz, $CDCl_3$) δ 7.27 - 7.18 (m, 6H), 7.17 (s, 1H), 7.11 - 7.05 (m, 4H), 3.98 (t, *J* = 7.1 Hz, 4H), 3.64 (q, *J* = 7.2 Hz, 2H), 3.39 (t, *J* = 7.1 Hz, 4H), 1.20 (t, *J* = 7.2 Hz, 3H).

^{13}C NMR (100 MHz, $CDCl_3$) δ 167.52, 167.30, 149.65, 148.97, 139.68, 135.26, 132.17, 131.36, 130.58, 129.62, 128.02, 127.56, 127.49, 124.49, 118.26, 67.08, 51.80, 32.87, 13.97.

HRMS (ESI) Calcd. for $C_{26}H_{25}N_2O_3$ ($[M+H]^+$): 413.1860, found: 413.1854.

7-(3,5-dimethylmorpholino)-2-ethyl-4,5-diphenylisoindoline-1,3-dione (5p)

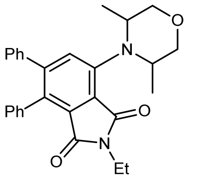

5p

The title product was prepared according to the **General Procedure B:** Carried out with 1,2-diphenylbutan-1-one (89.65 mg, 0.4 mmol, 1.0 equiv.), 3,5-dimethylmorpholine (46.04 mg, 0.4 mmol), $Cu(OAc)_2$ (7.26 mg, 0.04 mmol, 10 mol%), 2,2'-bipyridine (6.25 mg, 0.04 mmol, 10 mol%), N-ethyl maleimide (75.08 mg, 0.6 mmol, 1.5 equiv.), TEMPO (187.50 mg, 1.2 mmol, 3.0 equiv.), CsOAc (15.36 mg, 0.08 mmol, 0.2 equiv.) in toluene (1.5 mL) at 120 °C for 48 h. After concentrated and purified by flash column chromatography on silica gel (eluent = petroleum ether / ethyl acetate = 100:10), the product **5p** was obtained as yellow solid (133.82 mg, 76% yield).

^{1}H NMR (400 MHz, $CDCl_3$) δ 7.26 - 7.19 (m, 6H), 7.15 (s, 1H), 7.12 - 7.07 (m, 4H), 4.05 - 3.98 (m, 2H), 3.71 - 3.62 (m, 4H), 2.66 (t, *J* = 10.8 Hz, 2H), 1.29 (d, *J* = 6.3 Hz, 7H), 1.21 (t, *J* = 7.1 Hz, 3H).

^{13}C NMR (100 MHz, $CDCl_3$) δ 167.48, 167.21, 149.53, 148.54, 139.67, 135.25, 131.82, 131.27, 130.52, 129.56, 127.94, 127.46, 127.38, 124.56, 117.93, 71.86, 56.96, 32.79, 18.97, 13.92.

HRMS (ESI) Calcd. for $C_{28}H_{29}N_2O_3$ ($[M+H]^+$): 441.2173, found: 441.2166.

2-Ethyl-4,5-diphenyl-7-thiomorpholinoisoindoline-1,3-dione (5q)

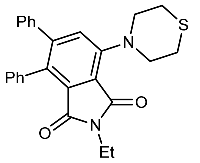

5q

The title product was prepared according to the **General Procedure B:** Carried out with 1,2-diphenylbutan-1-one (89.65 mg, 0.4 mmol, 1.0 equiv.), thiomorpholine (41.22 mg, 0.4 mmol), $Cu(OAc)_2$ (7.26 mg, 0.04 mmol, 10 mol%), 2,2'-bipyridine (6.25 mg, 0.04 mmol, 10 mol%), N-ethyl maleimide (75.08 mg, 0.6 mmol, 1.5 equiv.), TEMPO (187.50 mg, 1.2 mmol, 3.0 equiv.), CsOAc (15.36 mg, 0.08

mmol, 0.2 equiv.) in toluene (1.5 mL) at 120 °C for 48 h. After concentrated and purified by flash column chromatography on silica gel (eluent = petroleum ether / ethyl acetate = 100:10), the product **5q** was obtained as red solid (85.63 mg, 50% yield).

^{1}H NMR (400 MHz, $CDCl_3$) δ 7.28 - 7.18 (m, 6H), 7.17 (s, 1H), 7.11 - 7.05 (m, 4H), 3.70 - 3.56 (m, 6H), 2.94 (t, *J* = 7.2 Hz, 4H), 1.20 (t, *J* = 7.1 Hz, 3H).

^{13}C NMR (100 MHz, $CDCl_3$) δ 167.51, 167.17, 149.75, 149.53, 139.69, 135.27, 132.12, 131.30, 130.57, 129.62, 128.02, 127.54, 127.48, 125.37, 118.56, 54.35, 32.84, 28.06, 13.99.

HRMS (ESI) Calcd. for $C_{26}H_{25}N_2O_2S$ ($[M+H]^+$): 429.1631, found: 429.1626.

2-Ethyl-4,5-diphenyl-7-(1,4-dioxa-8-azaspiro[4.5]decan-8-yl)isoindoline-1,3-dione (5r)

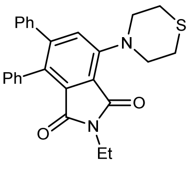

5q

The title product was prepared according to the **General Procedure B:** Carried out with 1,2-diphenylbutan-1-one (89.65 mg, 0.4 mmol, 1.0 equiv.), 1,4-dioxa-8-azaspiro[4.5]decane (57.24 mg, 0.4 mmol), $Cu(OAc)_2$ (7.26 mg, 0.04 mmol, 10 mol%), 2,2'-bipyridine (6.25 mg, 0.04 mmol, 10 mol%), N-ethyl maleimide (75.08 mg, 0.6 mmol, 1.5 equiv.), TEMPO (187.50 mg, 1.2 mmol, 3.0 equiv.), CsOAc (15.36 mg, 0.08 mmol, 0.2 equiv.) in toluene (1.5 mL) at 120 °C for 48 h. After concentrated and purified by flash column chromatography on silica gel (eluent = petroleum ether / ethyl acetate = 100:10), the product **5r** was obtained as yellow solid (116.11 mg, 62% yield).

^{1}H NMR (400 MHz, $CDCl_3$) δ 7.25 - 7.21 (m, 3H), 7.19 - 7.16 (m, 4H), 7.13 - 7.08 (m, 2H), 7.08 - 7.03 (m, 2H), 4.02 (s, 4H), 3.64 (q, *J* = 7.2 Hz, 2H), 3.49 (t, *J* = 5.6 Hz, 4H), 2.01 (t, *J* = 5.6 Hz, 4H), 1.21 (t, *J* = 7.2 Hz, 4H).

^{13}C NMR (100 MHz, $CDCl_3$) δ 167.46, 167.10, 149.21, 148.88, 139.68, 135.30, 131.44, 131.11, 130.48, 129.48, 127.81, 127.34, 127.31, 127.29, 127.21, 125.08, 117.89, 106.81, 64.33, 49.85, 35.06, 32.64, 13.86.

HRMS (ESI) Calcd. for $C_{29}H_{29}N_2O_4$ ($[M+H]^+$): 469.2122, found: 469.2115.

2-Ethyl-4,5-diphenyl-7-(piperidin-1-yl)isoindoline-1,3-dione (5s)

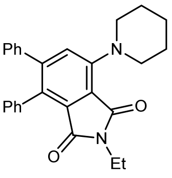

5s

The title product was prepared according to the **General Procedure B:** Carried out with 1,2-diphenylbutan-1-one (89.65 mg, 0.4 mmol, 1.0 equiv.), piperidine (34.04 mg, 0.4 mmol), $Cu(OAc)_2$ (7.26 mg, 0.04 mmol, 10 mol%), 2,2'-bipyridine (6.25 mg, 0.04 mmol, 10 mol%), N-ethyl maleimide (75.08

mg, 0.6 mmol, 1.5 equiv.), TEMPO (187.50 mg, 1.2 mmol, 3.0 equiv.), CsOAc (15.36 mg, 0.08 mmol, 0.2 equiv.) in toluene (1.5 mL) at 120 ºC for 48 h. After concentrated and purified by flash column chromatography on silica gel (eluent = petroleum ether / ethyl acetate = 100:10), the product **5s** was obtained as yellow solid (73.84 mg, 45% yield).

^{1}H NMR (400 MHz, $CDCl_3$) δ 7.25 - 7.21 (m, 3H), 7.21 - 7.16 (m, 4H), 7.12 - 7.08 (m, 2H), 7.08 - 7.04 (m, 2H), 3.64 (q, J = 7.2 Hz, 2H), 3.35 - 3.32 (t, J = 7.2 Hz, 4H), 1.89 - 1.83 (m, 4H), 1.69 - 1.63 (m, 2H), 1.20 (t, J = 7.2 Hz, 3H).

^{13}C NMR (100 MHz, $CDCl_3$) δ 167.74, 167.35, 149.98, 149.26, 139.98, 135.56, 131.29, 131.18, 130.67, 129.67, 127.94, 127.49, 127.38, 127.32, 125.07, 117.80, 53.08, 32.77, 26.15, 24.19, 14.01.

HRMS (ESI) Calcd. for $C_{27}H_{27}N_2O_2$ ($[M+H]^+$): 411.2067, found: 411.2061.

2-Ethyl-7-(2-methylpiperidin-1-yl)-4,5-diphenylisoindoline-1,3-dione (5t)

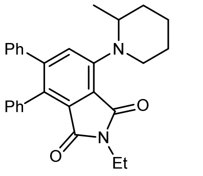

5t

The title product was prepared according to the **General Procedure B:** Carried out with 1,2-diphenylbutan-1-one (89.65 mg, 0.4 mmol, 1.0 equiv.), 2-methylpiperidine (39.64 mg, 0.4 mmol), $Cu(OAc)_2$ (7.26 mg, 0.04 mmol, 10 mol%), 2,2'-bipyridine (6.25 mg, 0.04 mmol, 10 mol%), N-ethyl maleimide (75.08 mg, 0.6 mmol, 1.5 equiv.), TEMPO (187.50 mg, 1.2 mmol, 3.0 equiv.), CsOAc (15.36 mg, 0.08 mmol, 0.2 equiv.) in toluene (1.5 mL) at 120 ºC for 48 h. After concentrated and purified by flash column chromatography on silica gel (eluent = petroleum ether / ethyl acetate = 100:10), the product **5t** was obtained as yellow solid (101.81 mg, 60% yield).

^{1}H NMR (400 MHz, $CDCl_3$) δ 7.36 - 7.15 (m, 8H), 7.13 - 6.96 (m, 3H), 4.32 - 4.29 (m, 1H), 3.66 (q, J = 7.3 Hz, 2H), 3.45 - 3.26 (m, 2H), 2.15 (m, 1H), 1.90 - 1.56 (m, 5H), 1.27 - 1.15 (m, 6H).

^{13}C NMR (100 MHz, $CDCl_3$) δ 167.75, 167.31, 149.40, 148.93, 140.02, 135.66, 131.40, 130.96, 129.72, 127.95, 127.48, 127.36, 127.30, 126.59, 117.90, 54.26, 45.80, 32.76, 30.98, 26.22, 18.98, 14.53, 14.04.

HRMS (ESI) Calcd. for $C_{28}H_{29}N_2O_2$($[M+H]^+$): 425.2224, found: 425.2217.

2-Ethyl-4,5-diphenyl-7-(2-phenylpiperidin-1-yl)isoindoline-1,3-dione (5u)

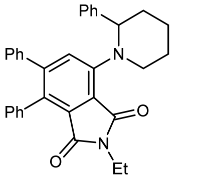

5u

The title product was prepared according to the **General Procedure B:** Carried out with 1,2-diphenylbutan-1-one (89.65 mg, 0.4 mmol, 1.0 equiv.), 2-phenylpiperidine (64.45 mg, 0.4 mmol),

$Cu(OAc)_2$ (7.26 mg, 0.04 mmol, 10 mol%), 2,2'-bipyridine (6.25 mg, 0.04 mmol, 10 mol%), N-ethyl maleimide (75.08 mg, 0.6 mmol, 1.5 equiv.), TEMPO (187.50 mg, 1.2 mmol, 3.0 equiv.), CsOAc (15.36 mg, 0.08 mmol, 0.2 equiv.) in toluene (1.5 mL) at 120 °C for 48 h. After concentrated and purified by flash column chromatography on silica gel (eluent = petroleum ether / ethyl acetate = 100:10), the product **5u** was obtained as yellow solid (85.58 mg, 44% yield).

^{1}H NMR (400 MHz, $CDCl_3$) δ 7.43 - 7.30 (m, 6H), 7.28 - 7.20 (m, 2H), 7.18 - 7.03 (m, 5H), 6.69 - 6.62 (m, 3H), 4.45 (dd, *J* = 9.1, 3.4 Hz, 1H), 3.94 (dt, *J* = 12.0, 4.3 Hz, 1H), 3.69 (q, *J* = 7.5 Hz, 2H), 3.06 (ddd, *J* = 12.3, 10.3, 2.6 Hz, 1H), 2.20 - 2.04 (m, 2H), 1.99 - 1.86 (m, 3H), 1.67 - 1.54 (m, 1H), 1.27 (t, *J* = 7.5 Hz, 3H).

^{13}C NMR (100 MHz, $CDCl_3$) δ 167.63, 166.98, 149.42, 147.70, 143.98, 139.65, 135.26, 131.86, 131.31, 130.91, 130.07, 129.79, 129.58, 128.69, 127.72, 127.50, 127.31, 127.16, 126.77, 120.63, 63.35, 55.61, 36.07, 32.74, 26.26, 23.93, 14.02.

HRMS (ESI) Calcd. for $C_{33}H_{31}N_2O_2$ ($[M+H]^+$): 487.2380, found: 487.2371.

2-Ethyl-4,5-diphenyl-7-(4-phenylpiperidin-1-yl)isoindoline-1,3-dione (5v)

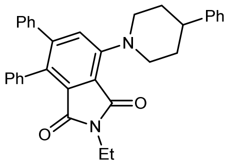

5v

The title product was prepared according to the **General Procedure B:** Carried out with 1,2-diphenylbutan-1-one (89.65 mg, 0.4 mmol, 1.0 equiv.), 4-phenylpiperidine (64.45 mg, 0.4 mmol), $Cu(OAc)_2$ (7.26 mg, 0.04 mmol, 10 mol%), 2,2'-bipyridine (6.25 mg, 0.04 mmol, 10 mol%), N-ethyl maleimide (75.08 mg, 0.6 mmol, 1.5 equiv.), TEMPO (187.50 mg, 1.2 mmol, 3.0 equiv.), CsOAc (15.36 mg, 0.08 mmol, 0.2 equiv.) in toluene (1.5 mL) at 120 °C for 48 h. After concentrated and purified by flash column chromatography on silica gel (eluent = petroleum ether / ethyl acetate = 100:10), the product **5v** was obtained as yellow solid (77.80 mg, 40% yield).

^{1}H NMR (400 MHz, $CDCl_3$) δ 7.41 - 7.38 (m, 4H), 7.34 - 7.22 (m, 8H), 7.19- 7.14 (dt, 4H), 4.02 (d, *J* = 11.6 Hz, 2H), 3.72 (q, *J* = 7.1 Hz, 2H), 3.10 (t, *J* = 11.7 Hz, 2H), 2.78 (tt, *J* = 12.2, 3.8 Hz, 1H), 2.21 (qd, *J* = 12.4, 3.7 Hz, 2H), 2.13 - 2.02 (m, 2H), 1.28 (t, *J* = 7.1 Hz, 3H).

^{13}C NMR (100 MHz, $CDCl_3$) δ 167.57, 167.24, 149.46, 149.27, 145.82, 139.82, 135.40, 131.37, 131.24, 130.59, 129.59, 128.52, 127.89, 127.43, 127.36, 127.29, 126.97, 126.36, 125.06, 117.93, 52.70, 42.40, 33.54, 32.73, 13.95.

HRMS (ESI) Calcd. for $C_{33}H_{31}N_2O_2$ ($[M+H]^+$): 487.2380, found: 487.2375.

7-(4-Chloropiperidin-1-yl)-2-ethyl-4,5-diphenylisoindoline-1,3-dione (5w)

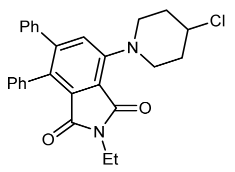

5w

The title product was prepared according to the **General Procedure B:** Carried out with 1,2-diphenylbutan-1-one (89.65 mg, 0.4 mmol, 1.0 equiv.), 4-chloropiperidine (47.62 mg, 0.4 mmol), $Cu(OAc)_2$ (7.26 mg, 0.04 mmol, 10 mol%), 2,2'-bipyridine (6.25 mg, 0.04 mmol, 10 mol%), N-ethyl maleimide (75.08 mg, 0.6 mmol, 1.5 equiv.), TEMPO (187.50 mg, 1.2 mmol, 3.0 equiv.), CsOAc (15.36 mg, 0.08 mmol, 0.2 equiv.) in toluene (1.5 mL) at 120 °C for 48 h. After concentrated and purified by flash column chromatography on silica gel (eluent = petroleum ether / ethyl acetate = 100:10), the product **5w** was obtained as yellow solid (87.06 mg, 49% yield).

^{1}H NMR (400 MHz, $CDCl_3$) δ 7.25 - 7.22 (m, 3H), 7.22 - 7.16 (m, 4H), 7.10 (dd, J = 6.5, 2.9 Hz, 2H), 7.05 (dd, J = 6.5, 2.9 Hz, 2H), 4.34 (tt, J = 7.3, 3.7 Hz, 1H), 3.70 - 3.57 (m, 4H), 3.31 (ddd, J = 11.5, 7.3, 3.7 Hz, 2H), 2.38 (ddt, J = 14.4, 7.4, 3.7 Hz, 2H), 2.17 (dtd, J = 13.9, 7.3, 3.7 Hz, 2H), 1.21 (t, J = 7.3 Hz, 3H).

^{13}C NMR (100 MHz, $CDCl_3$) δ 167.58, 167.30, 149.50, 149.04, 139.75, 135.34, 131.90, 131.28, 130.61, 129.63, 128.01, 127.54, 127.52, 127.46, 125.12, 118.24, 56.90, 49.06, 35.26, 32.84, 14.01.

HRMS (ESI) Calcd. for $C_{27}H_{26}N_2O_2Cl$ ($[M+H]^+$): 445.1677, found: 445.1671.

2-Ethyl-7-(4-methylpiperidin-1-yl)-4,5-diphenylisoindoline-1,3-dione (5x)

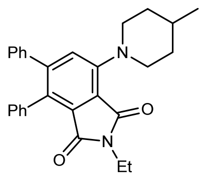

5x

The title product was prepared according to the **General Procedure B:** Carried out with 1,2-diphenylbutan-1-one (89.65 mg, 0.4 mmol, 1.0 equiv.), 4-methylpiperidine (39.64 mg, 0.4 mmol), $Cu(OAc)_2$ (7.26 mg, 0.04 mmol, 10 mol%), 2,2'-bipyridine (6.25 mg, 0.04 mmol, 10 mol%), N-ethyl maleimide (75.08 mg, 0.6 mmol, 1.5 equiv.), TEMPO (187.50 mg, 1.2 mmol, 3.0 equiv.), CsOAc (15.36 mg, 0.08 mmol, 0.2 equiv.) in toluene (1.5 mL) at 120 °C for 48 h. After concentrated and purified by flash column chromatography on silica gel (eluent = petroleum ether / ethyl acetate = 100:10), the product **5x** was obtained as yellow solid (72.97 mg, 43% yield).

^{1}H NMR (400 MHz, $CDCl_3$) δ 7.26 - 7.22 (m, 3H), 7.22 - 7.17 (m, 4H), 7.11 (dd, J = 6.6, 3.0 Hz, 2H), 7.08 (dd, J = 6.6, 3.0 Hz, 2H), 3.81 (d, J = 11.1 Hz, 2H), 3.65 (q, J = 7.2 Hz, 2H), 2.92 (t, J = 11.1 Hz, 2H), 1.83 (d, J = 9.1 Hz, 2H), 1.60 (d, J = 7.3 Hz, 3H), 1.21 (t, J = 7.1 Hz, 3H), 1.05 (d, J = 4.6 Hz, 3H).

^{13}C NMR (100 MHz, $CDCl_3$) δ 167.68, 167.29, 149.70, 149.21, 139.93, 135.52, 131.25, 131.12, 130.63, 129.62, 127.89, 127.44, 127.34, 127.27, 125.10, 117.71, 52.37, 34.39, 32.73, 30.62, 21.96, 13.97.

HRMS (ESI) Calcd. for $C_{28}H_{29}N_2O_2$ ($[M+H]^+$): 425.2224, found: 425.2216.

Ethyl 1-(2-ethyl-1,3-dioxo-6,7-diphenylisoindolin-4-yl)piperidine-3-carboxylate （5y）

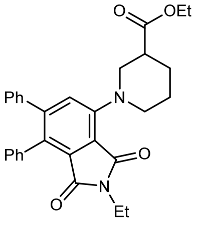

5y

The title product was prepared according to the **General Procedure B:** Carried out with 1,2-diphenylbutan-1-one (89.65 mg, 0.4 mmol, 1.0 equiv.), ethyl piperidine-3-carboxylate (62.84 mg, 0.4 mmol), $Cu(OAc)_2$ (7.26 mg, 0.04 mmol, 10 mol%), 2,2'-bipyridine (6.25 mg, 0.04 mmol, 10 mol%), N-ethyl maleimide (75.08 mg, 0.6 mmol, 1.5 equiv.), TEMPO (187.50 mg, 1.2 mmol, 3.0 equiv.), CsOAc (15.36 mg, 0.08 mmol, 0.2 equiv.) in toluene (1.5 mL) at 120 ºC for 48 h. After concentrated and purified by flash column chromatography on silica gel (eluent = petroleum ether / ethyl acetate = 100:10), the product **5y** was obtained as yellow solid (86.80 mg, 45% yield).

^{1}H NMR (400 MHz, $CDCl_3$) δ 7.25 - 7.16 (m, 7H), 7.14 - 7.03 (m, 4H), 4.17 (q, J = 6.8 Hz, 2H), 3.97 (d, J = 11.0 Hz, 1H), 3.74 (d, J = 11.6 Hz, 1H), 3.64 (q, J = 7.1 Hz, 2H), 3.04 (t, J = 11.1 Hz, 1H), 2.92 (tt, J = 11.6, 3.8 Hz, 2H), 2.23 (d, J = 12.8 Hz, 1H), 1.94 (tq, J = 8.0, 3.8 Hz, 2H), 1.65 - 1.56 (m, 1H), 1.28 (t, J = 7.1 Hz, 3H), 1.21 (t, J = 7.1 Hz, 3H).

^{13}C NMR (100 MHz, $CDCl_3$) δ 173.90, 167.58, 167.13, 149.35, 149.26, 139.74, 135.37, 131.68, 131.22, 130.57, 129.62, 127.93, 127.47, 127.42, 127.35, 125.21, 118.24, 60.61, 53.81, 52.31, 41.92, 32.76, 27.18, 24.97, 14.28, 13.96.

HRMS (ESI) Calcd. for $C_{30}H_{31}N_2O_4$ ($[M+H]^+$): 483.2278, found: 483.2274.

7-((3S,4R)-3-((benzo[d][1,3]dioxol-5-yloxy)methyl)-4-(4-fluorophenyl)piperidin-1-yl)-2-ethyl-4,5-diphenylisoindoline-1,3-dione (5z)

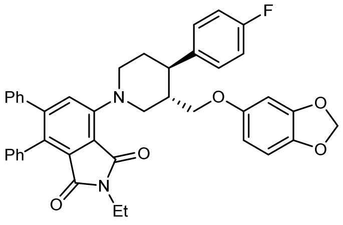

5z

The title product was prepared according to the **General Procedure B:** Carried out with 1,2-diphenylbutan-1-one (89.65 mg, 0.4 mmol, 1.0 equiv.), (3S,4R)-3-((benzo[d][1,3]dioxol-5-yloxy)methyl)-4-(4-fluorophenyl)piperidine (131.66 mg, 0.4 mmol), $Cu(OAc)_2$ (7.26 mg, 0.04 mmol, 10 mol%), 2,2'-bipyridine (6.25 mg, 0.04 mmol, 10 mol%), N-ethyl maleimide (75.08 mg, 0.6 mmol, 1.5 equiv.), TEMPO (187.50 mg, 1.2 mmol, 3.0 equiv.), CsOAc (15.36 mg, 0.08 mmol, 0.2 equiv.) in toluene

(1.5 mL) at 120 ºC for 48 h. After concentrated and purified by flash column chromatography on silica gel (eluent = petroleum ether / ethyl acetate = 100:10), the product **5z** was obtained as yellow solid (138.70 mg, 53% yield).

^{1}H NMR (400 MHz, $CDCl_3$) δ 7.34 - 7.18 (m, 9H), 7.13 - 7.08 (m, 4H), 7.01 (t, *J* = 8.7 Hz, 2H), 6.64 (d, *J* = 8.5 Hz, 1H), 6.38 (d, *J* = 2.5 Hz, 1H), 6.17 (dd, *J* = 8.5, 2.5 Hz, 1H), 5.88 (s, 2H), 4.17 - 4.05 (m, 1H), 4.01 - 3.93 (m, 1H), 3.78 - 3.62 (m, 3H), 3.58 (dd, *J* = 9.5, 6.2 Hz, 1H), 3.09 - 3.02 m, 2H), 2.78 (td, *J* = 11.9, 3.9 Hz, 1H), 2.54 - 2.49 (m, 1H), 2.24 (qd, *J* = 12.6, 4.1 Hz, 1H), 2.07 - 1.99 (m, 1H), 1.23 (t, *J* = 7.2 Hz, 3H).

^{13}C NMR (100 MHz, $CDCl_3$) δ 167.63, 167.33, 161.71 (d, $^1J_{C\text{-}F}$ = 244.65 Hz), 154.39, 149.46, 149.34, 148.23, 141.68, 139.82, 139.27 (d, $^4J_{C\text{-}F}$ = 3.14 Hz), 135.42, 131.74, 131.30, 130.64, 129.67, 129.08 (d, $^3J_{C\text{-}F}$ = 7.82 Hz), 127.99, 127.53, 127.48, 127.41, 125.16, 118.25, 115.56 (d, $^2J_{C\text{-}F}$ = 20.93 Hz), 107.93, 105.51, 101.19, 98.02, 69.05, 55.62, 52.80, 43.66, 42.55, 34.36, 32.84, 14.02.

^{19}F NMR (377 MHz, $CDCl_3$) δ -116.10.

HRMS (ESI) Calcd. for $C_{41}H_{36}N_2O_5F$ ($[M+H]^+$): 655.2603, found: 655.2596.

4-(Dibenzylamino)-2-ethyl-7-phenylisoindoline-1,3-dione (5aa)

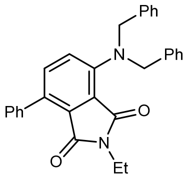

5aa

The title product was prepared according to the **General Procedure C**: Carried out with 1-phenylbutan-1-one (59.24 mg, 0.4 mmol), ethyl dibenzylglycinate (226.53 mg, 0.8 mmol, 2.0 equiv.), $Cu(OAc)_2$ (7.26 mg, 0.04 mmol, 10 mol%), 2,2'-bipyridine (6.25 mg, 0.04 mmol, 10 mol%), N-ethyl maleimide (75.08 mg, 0.6 mmol, 1.5 equiv.), TEMPO (187.50 mg, 1.2 mmol, 3.0 equiv.), p-Toluenesulfonic acid (6.88 mg, 0.04 mmol, 10 mol%, 12 wt.% solution in pure acetic acid) in toluene (1.5 mL) at 120 ºC for 48 h. After concentrated and purified by flash column chromatography on silica gel (eluent = petroleum ether / ethyl acetate = 100:10), the product **5aa** was obtained as yellow solid (64.25 mg, 36% yield).

^{1}H NMR (400 MHz, $CDCl_3$) δ 7.50 (d, *J* = 7.3 Hz, 2H), 7.46 - 7.23 (m, 14H), 7.16 (d, *J* = 8.6 Hz, 1H), 4.60 (s, 4H), 3.70 (q, *J* = 7.2 Hz, 2H), 1.25 (t, *J* = 7.2 Hz, 3H).

^{13}C NMR (100 MHz, $CDCl_3$) δ 167.80, 167.40, 148.45, 137.83, 136.97, 136.87, 132.72, 129.96, 129.58, 128.66, 128.13, 128.11, 127.98, 127.43, 125.32, 118.74, 56.73, 32.88, 14.07.

HRMS (ESI) Calcd. for $C_{30}H_{27}N_2O_2$ ($[M+H]^+$): 447.2067, found: 447.2061.

2-Ethyl-4-(mesityl(methyl)amino)-7-phenylisoindoline-1,3-dione (5ab)

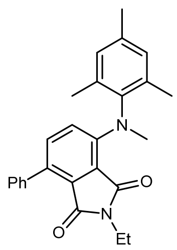

5ab

The title product was prepared according to the **General Procedure C**: Carried out with 1-phenylbutan-1-one (59.24 mg, 0.4 mmol), N,N,2,4,6-pentamethylaniline (130.51 mg, 0.8 mmol, 2.0 equiv.), $Cu(OAc)_2$ (7.26 mg, 0.04 mmol, 10 mol%), 2,2'-bipyridine (6.25 mg, 0.04 mmol, 10 mol%), N-ethyl maleimide (75.08 mg, 0.6 mmol, 1.5 equiv.), TEMPO (187.50 mg, 1.2 mmol, 3.0 equiv.), p-Toluenesulfonic acid (6.88 mg, 0.04 mmol, 10 mol%, 12 wt.% solution in pure acetic acid) in toluene (1.5 mL) at 120 ºC for 48 h. After concentrated and purified by flash column chromatography on silica gel (eluent = petroleum ether / ethyl acetate = 100:10), the product **5ab** was obtained as yellow solid (74.86 mg, 47% yield).

^{1}H NMR (400 MHz, $CDCl_3$) δ 7.53 - 7.48 (m, 2H), 7.47 - 7.35 (m, 3H), 7.16 (d, J = 8.8 Hz, 1H), 7.04 (s, 2H), 6.51 (d, J = 8.8 Hz, 1H), 3.73 (q, J = 7.1 Hz, 2H), 3.60 (s, 3H), 2.37 (s, 3H), 2.20 (s, 6H), 1.29 (t, J = 7.1 Hz, 3H).

^{13}C NMR (100 MHz, $CDCl_3$) δ 167.81, 167.42, 146.90, 140.98, 137.41, 137.21, 136.43, 136.37, 130.38, 130.01, 129.49, 129.46, 127.83, 127.73, 122.97, 111.95, 43.76, 32.73, 21.08, 17.78, 13.99.

HRMS (ESI) Calcd. for $C_{26}H_{27}N_2O_2$ ($[M+H]^+$): 399.2067, found: 399.2061.

4-(Diisopropylamino)-2,7-diphenylisoindoline-1,3-dione (8a)

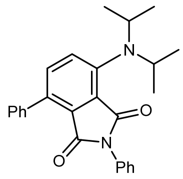

8a

The title product was prepared according to the **General Procedure A**: Carried out with 1-phenylbutan-1-one (118.47 mg, 0.8 mmol, 2.0 equiv.), diisopropylamine (40.48 mg, 0.4 mmol), $Cu(OAc)_2$ (7.26 mg, 0.04 mmol, 10 mol%), 2,2'-bipyridine (6.25 mg, 0.04 mmol, 10 mol%), N-phenyl maleimide (103.83 mg, 0.6 mmol, 1.5 equiv.), TEMPO (187.50 mg, 1.2 mmol, 3.0 equiv.), p-Toluenesulfonic acid (6.88 mg, 0.04 mmol, 10 mol%, 12 wt.% solution in pure acetic acid) in toluene (1.5 mL) at 120 ºC for 48 h. After concentrated and purified by flash column chromatography on silica gel (eluent = petroleum ether / ethyl acetate = 100:10), the product **8a** was obtained as yellow solid (116.27 mg, 73% yield).

^{1}H NMR (400 MHz, $CDCl_3$) δ 7.58 (d, J = 1.8 Hz, 1H), 7.57 - 7.55 (m, 1H), 7.53 (s, 1H), 7.49 (s, 1H), 7.48 - 7.39 (m, 7H), 7.38 - 7.32 (m, 1H), 4.04 (hept, J = 6.8 Hz, 2H), 1.37 (d, J = 6.8 Hz, 12H).

^{13}C NMR (100 MHz, $CDCl_3$) δ 166.74, 166.28, 147.82, 136.94, 136.18, 133.57, 132.09, 130.64, 129.57, 129.40, 128.89, 128.02, 127.90, 127.75, 127.10, 121.35, 51.15, 22.79.

HRMS (ESI) Calcd. for $C_{26}H_{27}N_2O_2$ ($[M+H]^+$): 399.2067, found: 399.2060.

2-(Tert-butyl)-7-morpholino-4,5-diphenylisoindoline-1,3-dione (8b)

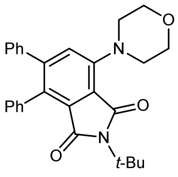

8b

The title product was prepared according to the **General Procedure B:** Carried out with 1,2-diphenylbutan-1-one (89.65 mg, 0.4 mmol, 1.0 equiv.), morpholine (34.83 mg, 0.4 mmol), $Cu(OAc)_2$ (7.26 mg, 0.04 mmol, 10 mol%), 2,2'-bipyridine (6.25 mg, 0.04 mmol, 10 mol%), N-tert-butyl maleimide (75.08 mg, 0.6 mmol, 1.5 equiv.), TEMPO (187.50 mg, 1.2 mmol, 3.0 equiv.), CsOAc (15.36 mg, 0.08 mmol, 0.2 equiv.) in toluene (1.5 mL) at 120 ºC for 48 h. After concentrated and purified by flash column chromatography on silica gel (eluent = petroleum ether / ethyl acetate = 100:15), the product **8b** was obtained as yellow solid (89.80 mg, 51% yield).

^{1}H NMR (400 MHz, $CDCl_3$) δ 7.26 - 7.16 (m, 6H), 7.15 (s, 1H), 7.06 (ddd, *J* = 18.5, 6.6, 2.9 Hz, 4H), 4.06 - 3.91 (m, 4H), 3.42 - 3.31 (m, 4H), 1.64 (s, 10H).

^{13}C NMR (100 MHz, $CDCl_3$) δ 168.61, 149.48, 148.64, 139.95, 135.72, 131.60, 131.26, 130.57, 129.63, 127.95, 127.52, 127.39, 127.33, 124.24, 118.68, 67.10, 57.75, 51.91, 29.36.

HRMS (ESI) Calcd. for $C_{28}H_{29}N_2O_3$ ($[M+H]^+$): 441.2173, found: 441.2167.

4-(Diisopropylamino)-2,7-dimethylisoindoline-1,3-dione (8c)

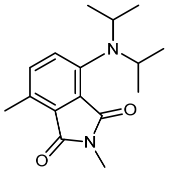

8c

The title product was prepared according to the **General Procedure A**: Carried out with pentan-2-one (68.86 mg, 0.8 mmol, 2.0 equiv.), diisopropylamine (40.48 mg, 0.4 mmol), $Cu(OAc)_2$ (7.26 mg, 0.04 mmol, 10 mol%), 2,2'-bipyridine (6.25 mg, 0.04 mmol, 10 mol%), N-methyl maleimide (66.62 mg, 0.6 mmol, 1.5 equiv.), TEMPO (187.50 mg, 1.2 mmol, 3.0 equiv.), p-Toluenesulfonic acid (6.88 mg, 0.04 mmol, 10 mol%, 12 wt.% solution in pure acetic acid) in toluene (1.5 mL) at 120 ºC for 48 h. After concentrated and purified by flash column chromatography on silica gel (eluent = petroleum ether / ethyl acetate = 100:10), the product **8c** was obtained as yellow solid (62.51 mg, 57% yield).

^{1}H NMR (400 MHz, $CDCl_3$) δ 7.22 (d, *J* = 8.4 Hz, 1H), 7.22 (d, *J* = 8.5 Hz, 1H), 3.80 (p, *J* = 6.6 Hz, 2H), 3.08 (s, 3H), 2.59 (s, 3H), 1.15 (d, *J* = 6.6 Hz, 12H).

^{13}C NMR (100 MHz, $CDCl_3$) δ 169.19, 167.70, 145.68, 136.15, 132.74, 131.40, 130.36, 124.77, 50.41, 23.59, 22.37, 17.24.

HRMS (ESI) Calcd. for $C_{16}H_{23}N_2O_2$ ($[M+H]^+$): 275.1754, found: 275.1749.

2-Benzyl-4-(diisopropylamino)-7-phenylisoindoline-1,3-dione (8d)

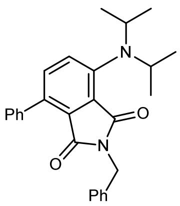

8d

The title product was prepared according to the **General Procedure A**: Carried out with 1-phenylbutan-1-one (118.47 mg, 0.8 mmol, 2.0 equiv.), diisopropylamine (40.48 mg, 0.4 mmol), $Cu(OAc)_2$ (7.26 mg, 0.04 mmol, 10 mol%), 2,2'-bipyridine (6.25 mg, 0.04 mmol, 10 mol%), 1-benzyl-1H-pyrrole-2,5-dione (112.24 mg, 0.6 mmol, 1.5 equiv.), TEMPO (187.50 mg, 1.2 mmol, 3.0 equiv.), p-Toluenesulfonic acid (6.88 mg, 0.04 mmol, 10 mol%, 12 wt.% solution in pure acetic acid) in toluene (1.5 mL) at 120 ºC for 48 h. After concentrated and purified by flash column chromatography on silica gel (eluent = petroleum ether / ethyl acetate = 100:10), the product **8d** was obtained as yellow solid (150.05 mg, 91% yield).

^{1}H NMR (400 MHz, $CDCl_3$) δ 7.59 - 7.54 (m, 2H), 7.53 - 7.40 (m, 7H), 7.36 - 7.30 (m, 2H), 7.30 - 7.26 (m, 1H), 4.82 (s, 2H), 4.01 (hept, J = 6.8 Hz, 2H), 1.34 (d, J = 6.8 Hz, 12H).

^{13}C NMR (100 MHz, $CDCl_3$) δ 167.39, 166.91, 147.23, 136.95, 136.92, 135.76, 133.58, 130.92, 129.57, 129.48, 128.72, 128.53, 127.99, 127.88, 127.52, 122.73, 50.88, 41.30, 22.60.

HRMS (ESI) Calcd. for $C_{26}H_{27}N_2O_2$ ($[M+H]^+$): 413.2224, found: 413.2226.

N,N-Diisopropyl-5-phenylfuran-2-amine (6a)

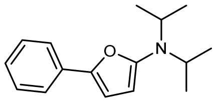

6a

The title compound was obtained according to **Experimental Procedures for Mechanistic Studies**.

^{1}H NMR (400 MHz, $CDCl_3$) δ 7.55 (d, J = 7.7 Hz, 2H), 7.33 (t, J = 7.7 Hz, 2H), 7.13 (t, J = 7.3 Hz, 1H), 6.58 (d, J = 3.2 Hz, 1H), 5.32 (d, J = 3.2 Hz, 1H), 3.61 (hept, J = 6.6 Hz, 2H), 1.26 (d, J = 6.6 Hz, 12H).

^{13}C NMR (100 MHz, $CDCl_3$) δ 157.34, 145.37, 131.78, 128.67, 125.42, 122.21, 106.69, 90.93, 48.74, 21.51.

HRMS (ESI) Calcd. for $C_{16}H_{22}NO$ ($[M+H]^+$): 244.1696, found: 244.1691.

2,2,6,6-Tetramethyl-1-(5-phenylfuran-2-yl)piperidine (6b)

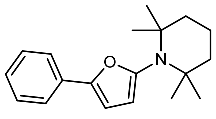

6b

The title compound was obtained according to **Experimental Procedures for Mechanistic Studies**..

^{1}H NMR (400 MHz, $CDCl_3$) δ 7.72 - 7.61 (m, 2H), 7.44 - 7.34 (m, 2H), 7.30 - 7.17 (m, 1H), 6.60 (s, 1H), 5.95 (s, 1H), 1.74 (s, 2H), 1.64 - 1.55 (m, 4H), 1.17 (d, J = 6.6 Hz, 12H).

^{13}C NMR (100 MHz, $CDCl_3$) δ 154.67, 148.88, 131.74, 128.70, 126.67, 123.39, 108.37, 105.13, 54.76, 40.55, 29.44, 18.02.

HRMS (ESI) Calcd. for $C_{19}H_{26}NO$ ($[M+H]^+$): 284.2009, found: 284.2004.

(E)-1-Phenylbut-2-en-1-one (6c)

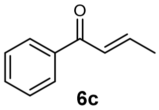

6c

The title compound was obtained according to **Experimental Procedures for Mechanistic Studies**.

^{1}H NMR (400 MHz, $CDCl_3$) δ 7.92 (d, J = 7.6 Hz, 2H), 7.55 (t, J = 7.2 Hz, 1H), 7.46 (t, J = 7.2 Hz, 2H), 7.07 (dq, J = 13.4, 6.5 Hz, 1H), 6.91 (d, J = 15.3 Hz, 1H), 2.00 (d, J = 6.6 Hz, 3H).

^{13}C NMR (100 MHz, $CDCl_3$) δ 190.90, 145.24, 137.97, 132.71, 128.61, 127.60, 18.74.

This compound is known.[7] The **^{1}H** and **^{13}C NMR** spectral data are in good agreement with the literature data.

(E)-4-oxo-4-phenylbut-2-enal (6d)

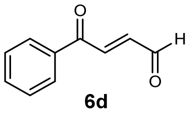

6d

The title compound was obtained according to **Experimental Procedures for Mechanistic Studies**.

^{1}H NMR (400 MHz, $CDCl_3$) δ 9.90 (d, J = 7.4 Hz, 1H), 7.99 (d, J = 7.8 Hz, 2H), 7.72 (d, J = 15.8 Hz, 1H), 7.66 (t, J = 7.4 Hz, 1H), 7.54 (t, J = 7.7 Hz, 2H), 7.00 (dd, J = 15.8, 7.4 Hz, 1H).

^{13}C NMR (100 MHz, $CDCl_3$) δ 192.95, 189.88, 142.21, 139.26, 136.34, 134.29, 129.14, 128.98.

This compound is known.[8] The **^{1}H** and **^{13}C NMR** spectral data are in good agreement with the literature data.

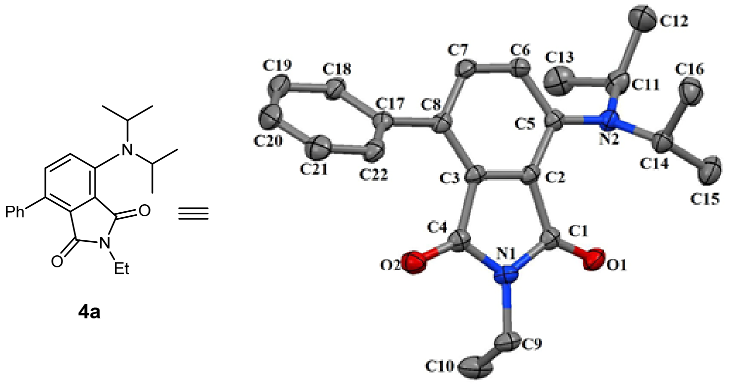

Supplementary Figure 2. X-ray structure of compound **4a**. Hydrogen atoms have been omitted for clarity. The thermal ellipsoid was drawn at the 50% probability level. (**CCDC 1905935)**

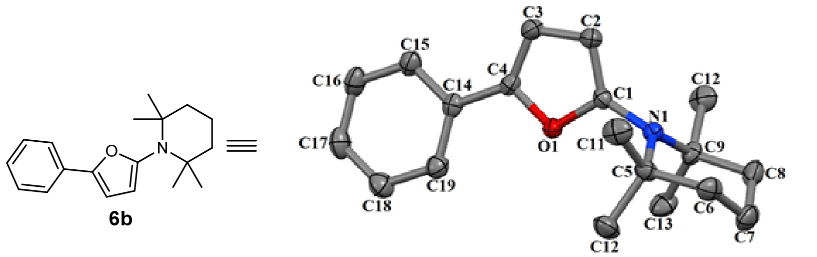

Supplementary Figure 3. X-ray structure of compound **6b**. The thermal ellipsoid was drawn at the 50% probability level. (**CCDC 1905936)**

Supplementary Table 2. Crystal data and structure refinement for compound 4a.

Identification code	4a
Empirical formula	C22 H26 N2 O2
Formula weight	350.45
Temperature	273(2) K
Wavelength	0.71073 Å
Crystal system	Monoclinic
Space group	Pn
Unit cell dimensions	a = 17.402(4) Å, α = 90°<br>b = 11.776(3) Å, β = 100.726(14)°<br>c = 19.132(5) Å, γ = 90°
Volume	3852.1(15) $Å^3$
Z	8
Density (calculated)	1.209 Mg/m^3
Absorption coefficient	0.078 mm^{-1}
F(000)	1504
Crystal size	0.24 x 0.22 x 0.22 mm^3
Theta range for data collection	2.17 to 27.45°
Index ranges	-22<=h<=22, -15<=k<=15, -24<=l<=24
Reflections collected	68238
Independent reflections	16936 [R(int) = 0.0757]
Completeness to theta = 27.45°	99.7 %
Absorption correction	Semi-empirical from equivalents
Max. and min. transmission	0.9831 and 0.9816
Refinement method	Full-matrix least-squares on F^2
Data / restraints / parameters	16936 / 2 / 937
Goodness-of-fit on F^2	1.109
Final R indices [I>2sigma(I)]	R1 = 0.0574, wR2 = 0.1340
R indices (all data)	R1 = 0.0763, wR2 = 0.1485
Absolute structure parameter	1.3(9)
Largest diff. peak and hole	0.410 and -0.458 e.$Å^{-3}$

Supplementary Table 3. Crystal data and structure refinement for compound 6b.

Identification code	6b
Empirical formula	C19 H25 N O
Formula weight	283.40
Temperature	160(2) K
Wavelength	0.71073 Å
Crystal system	Monoclinic
Space group	$P2_1/c$
Unit cell dimensions	a = 10.2135(6) Å, α= 90°<br>b = 16.6286(8) Å, β= 103.744(2)°<br>c = 9.9041(5) Å, γ = 90°
Volume	1633.91(15) $Å^3$
Z	4
Density (calculated)	1.152 Mg/m^3
Absorption coefficient	0.070 mm^{-1}
F(000)	616
Crystal size	0.26 x 0.24 x 0.22 mm^3
Theta range for data collection	2.852 to 27.503°
Index ranges	-13<=h<=13, -21<=k<=21, -12<=l<=12
Reflections collected	33829
Independent reflections	3721 [R(int) = 0.0860]
Completeness to theta = 27.45°	99.5 %
Absorption correction	Semi-empirical from equivalents
Refinement method	Full-matrix least-squares on F^2
Data / restraints / parameters	3721 / 0 / 190
Goodness-of-fit on F^2	1.051
Final R indices [I>2sigma(I)]	R1 = 0.0451, wR2 = 0.1084
R indices (all data)	R1 = 0.0628, wR2 = 0.1137
Extinction coefficient	n/a
Largest diff. peak and hole	0.244 and -0.181 e.$Å^{-3}$

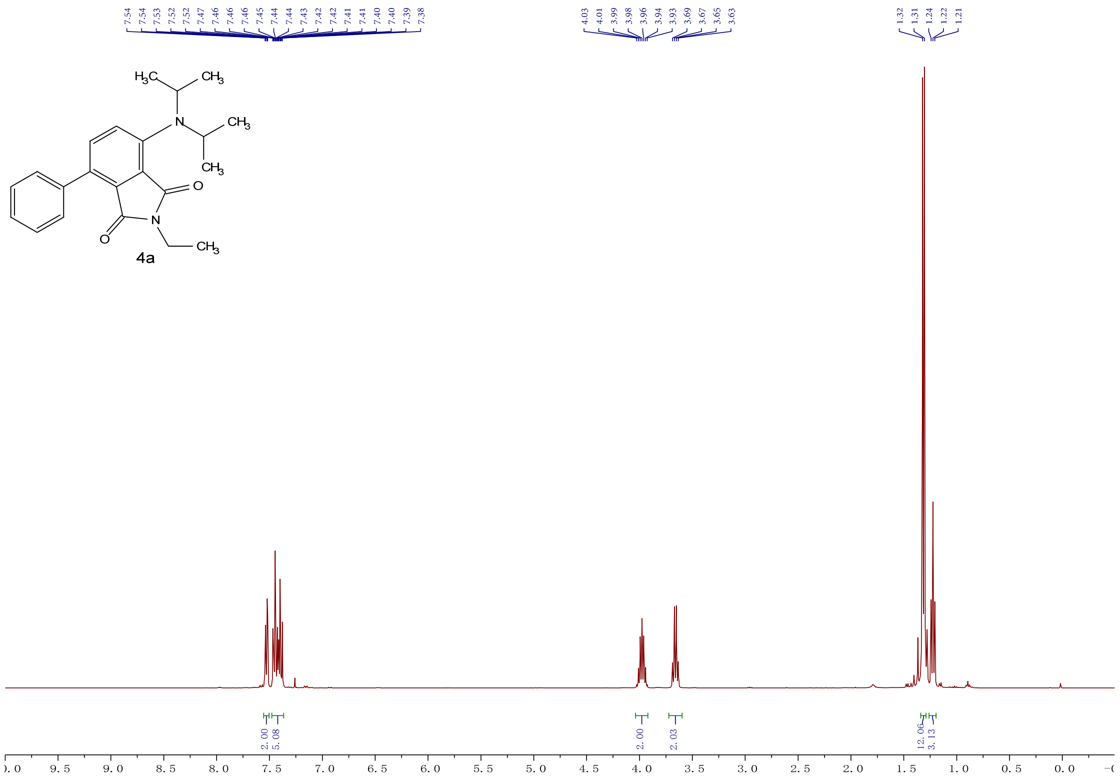

Supplementary Figure 4. ^{1}H NMR spectrum of 4a

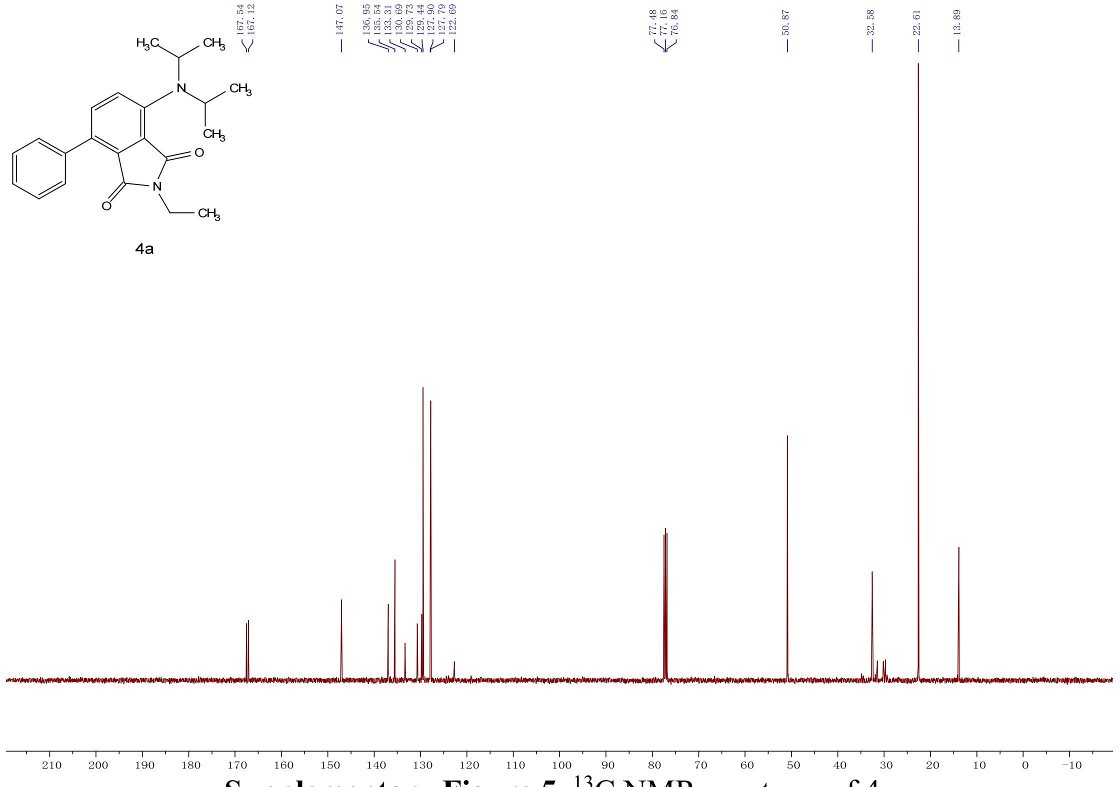

Supplementary Figure 5. ^{13}C NMR spectrum of 4a

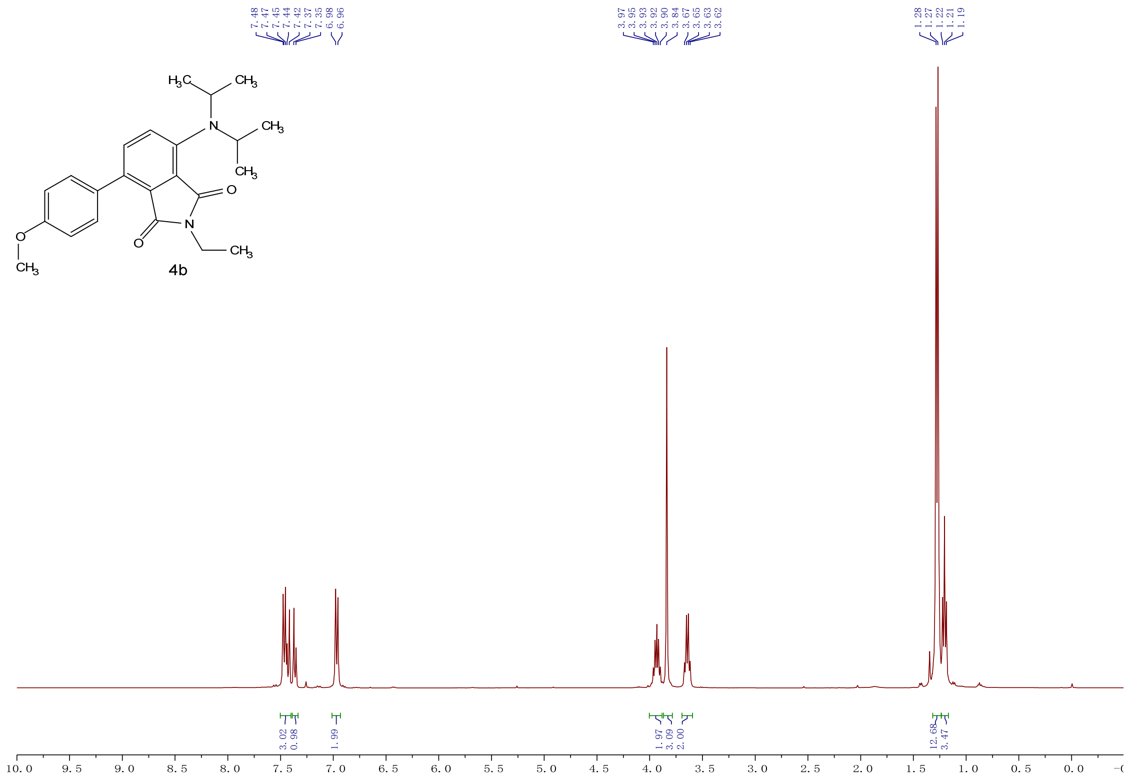

Supplementary Figure 6. ^{1}H NMR spectrum of 4b

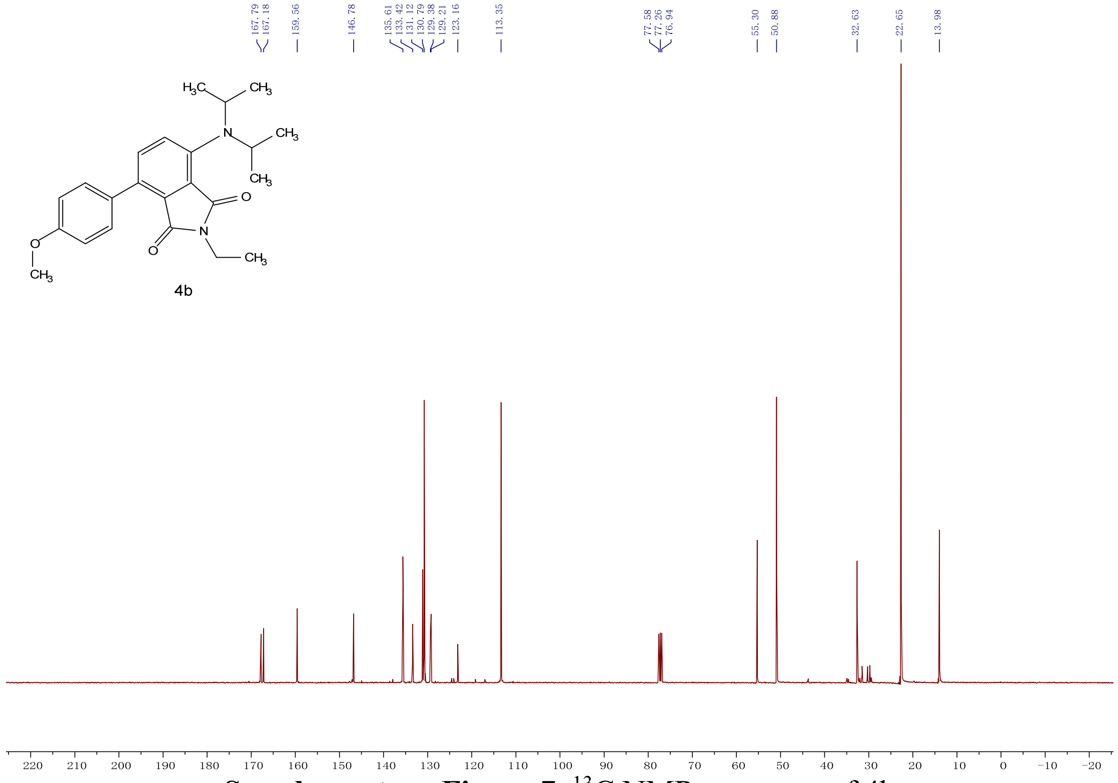

Supplementary Figure 7. ^{13}C NMR spectrum of 4b

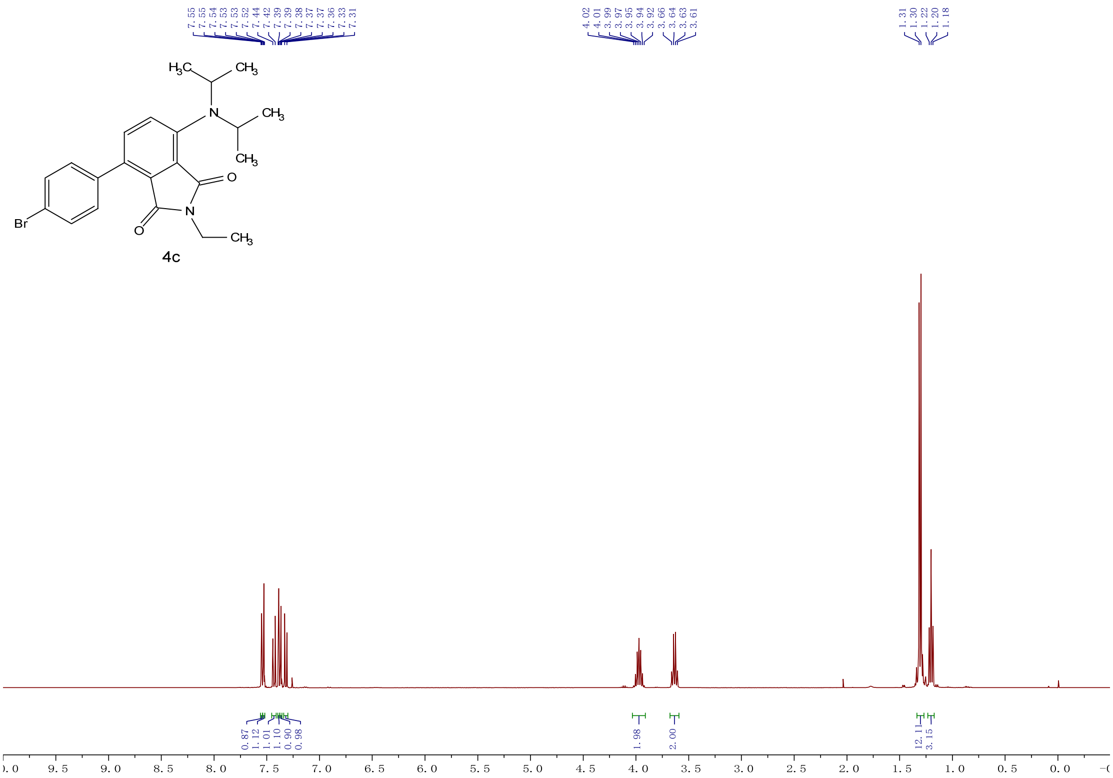

Supplementary Figure 8. ^{1}H NMR spectrum of 4c

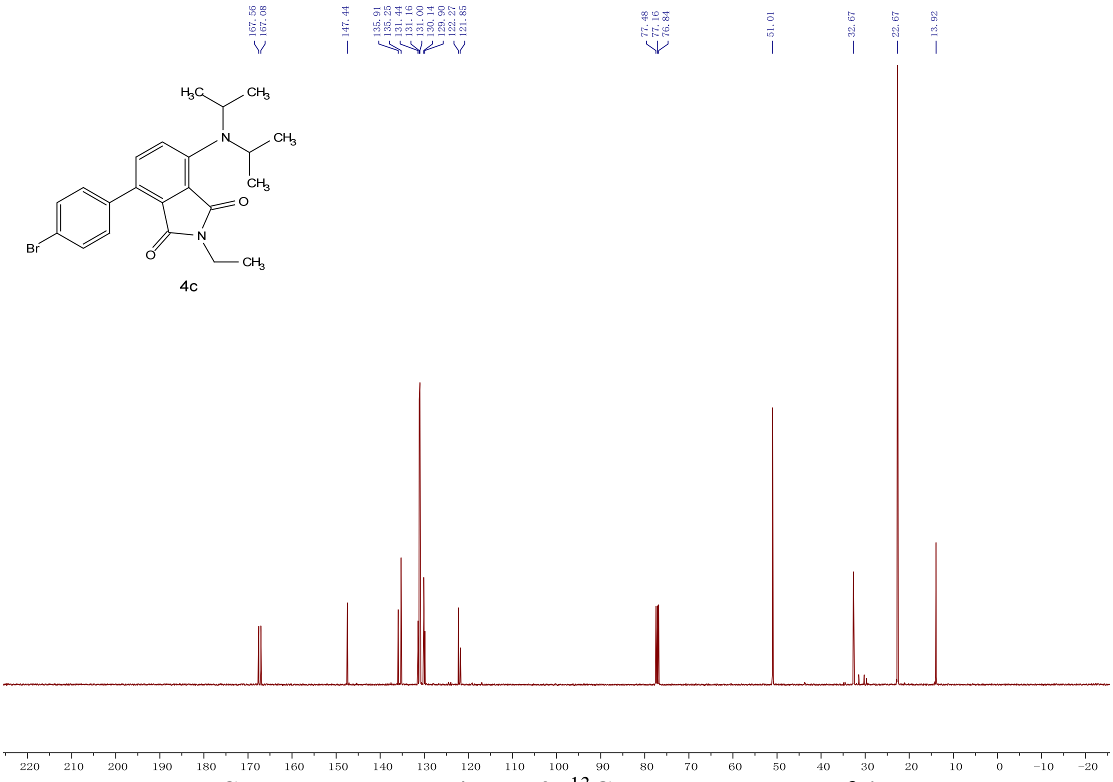

Supplementary Figure 9. ^{13}C NMR spectrum of 4c

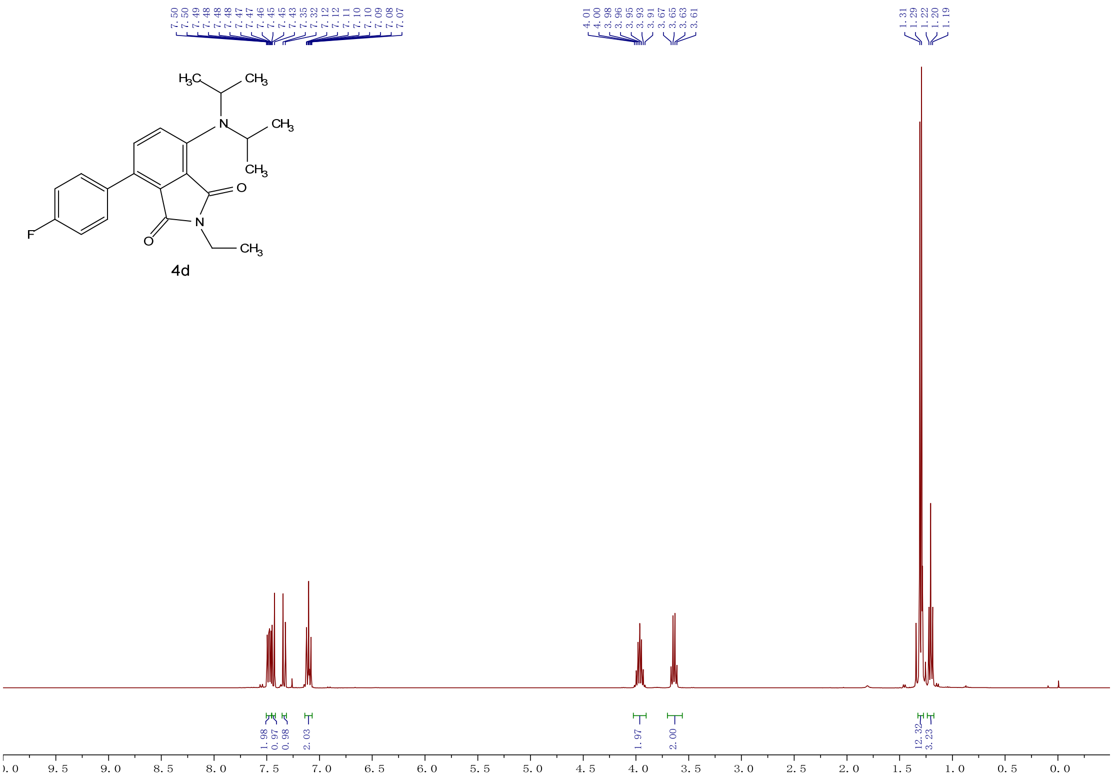

Supplementary Figure 10. ^{1}H NMR spectrum of 4d

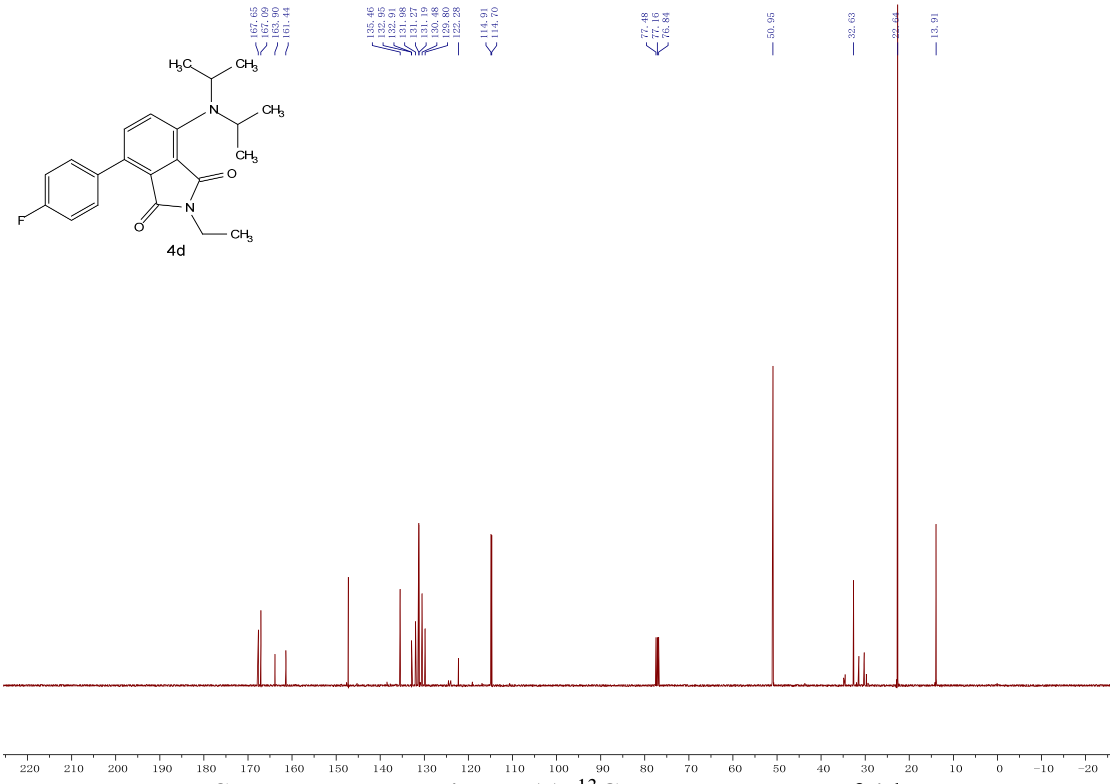

Supplementary Figure 11. ^{13}C NMR spectrum of 4d

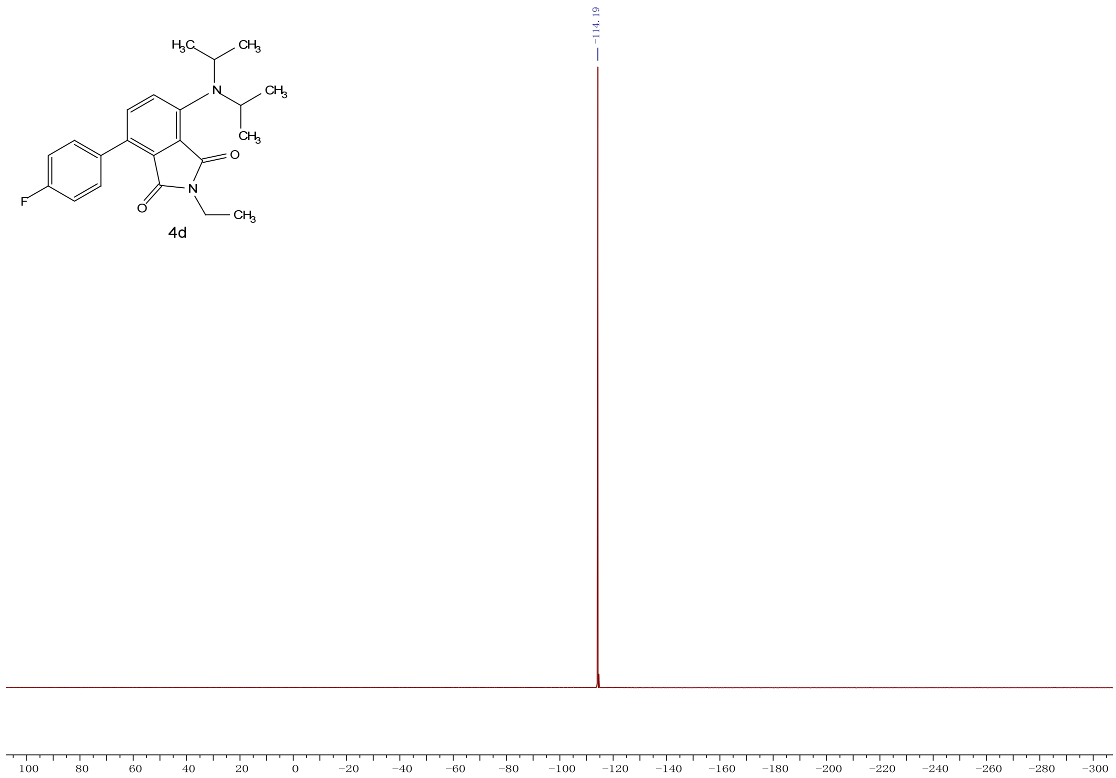

Supplementary Figure 12. ^{19}F NMR spectrum of 4d

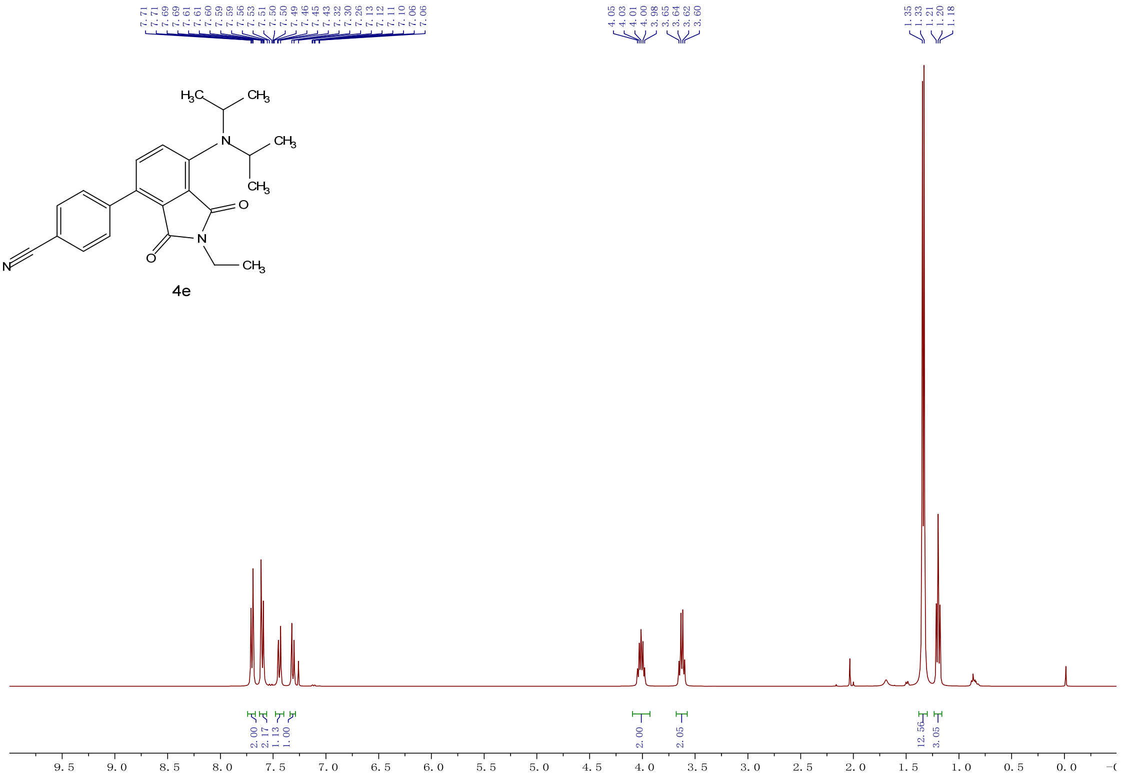

Supplementary Figure 13. ^{1}H NMR spectrum of 4e

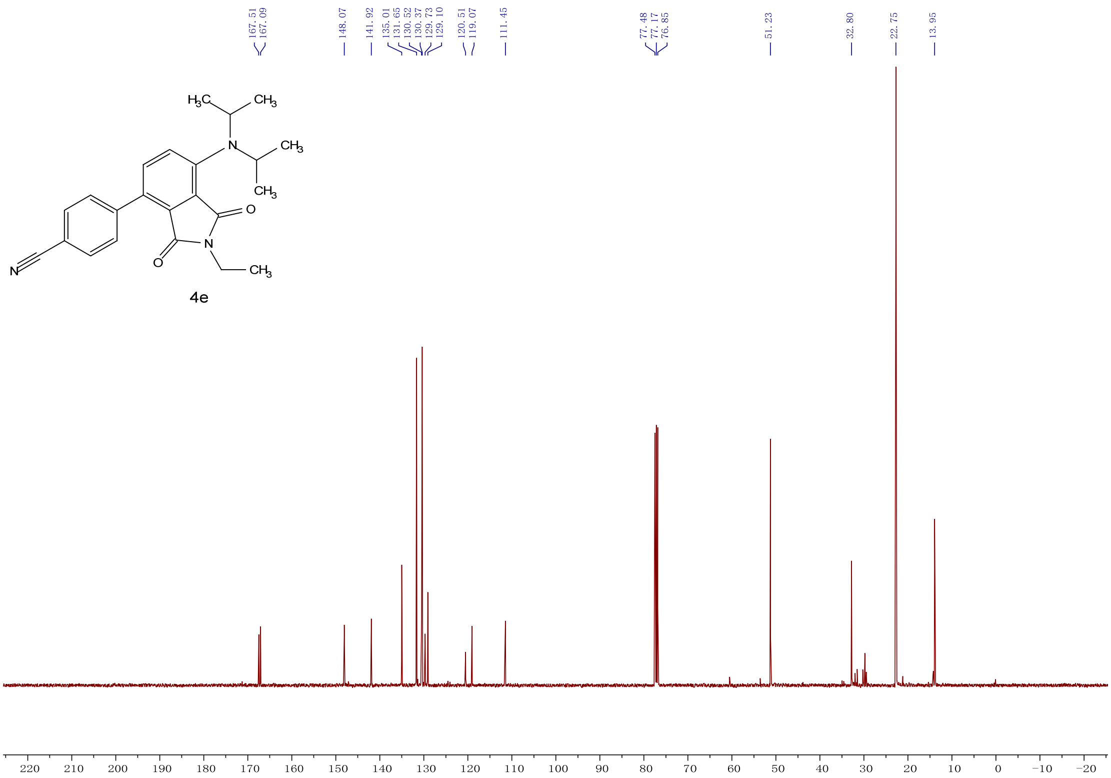

Supplementary Figure 14. ^{13}C NMR spectrum of 4e

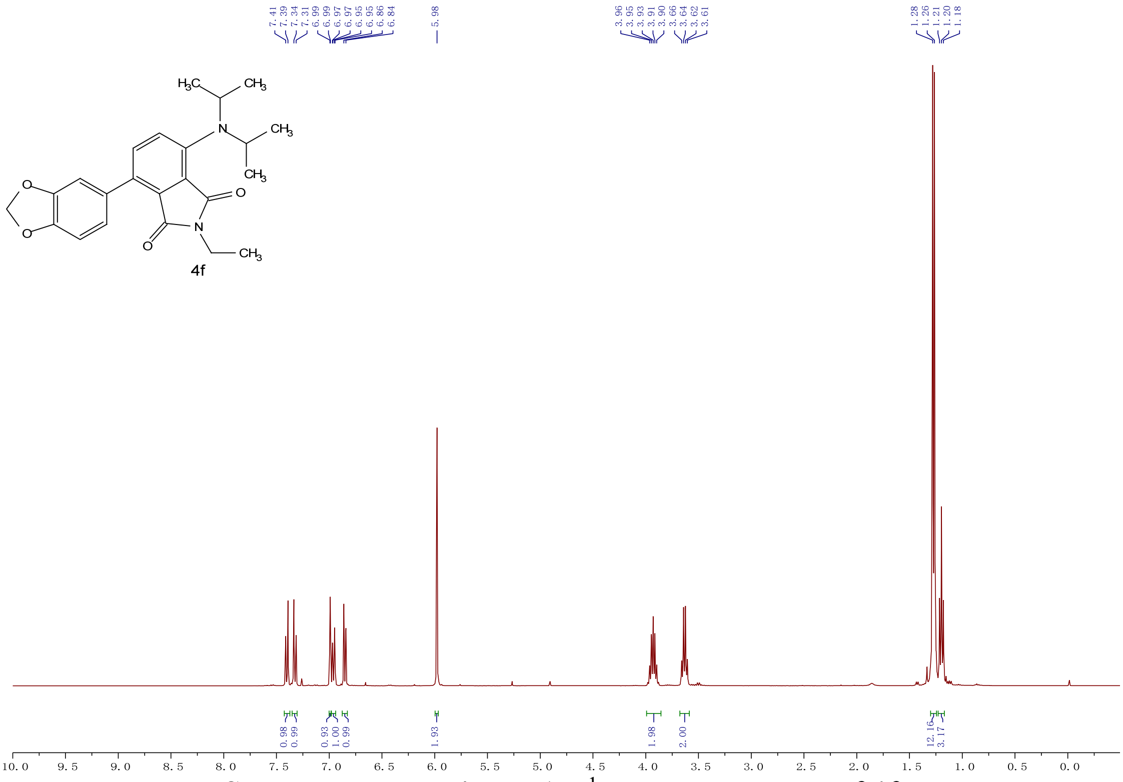

Supplementary Figure 15. ^{1}H NMR spectrum of 4f

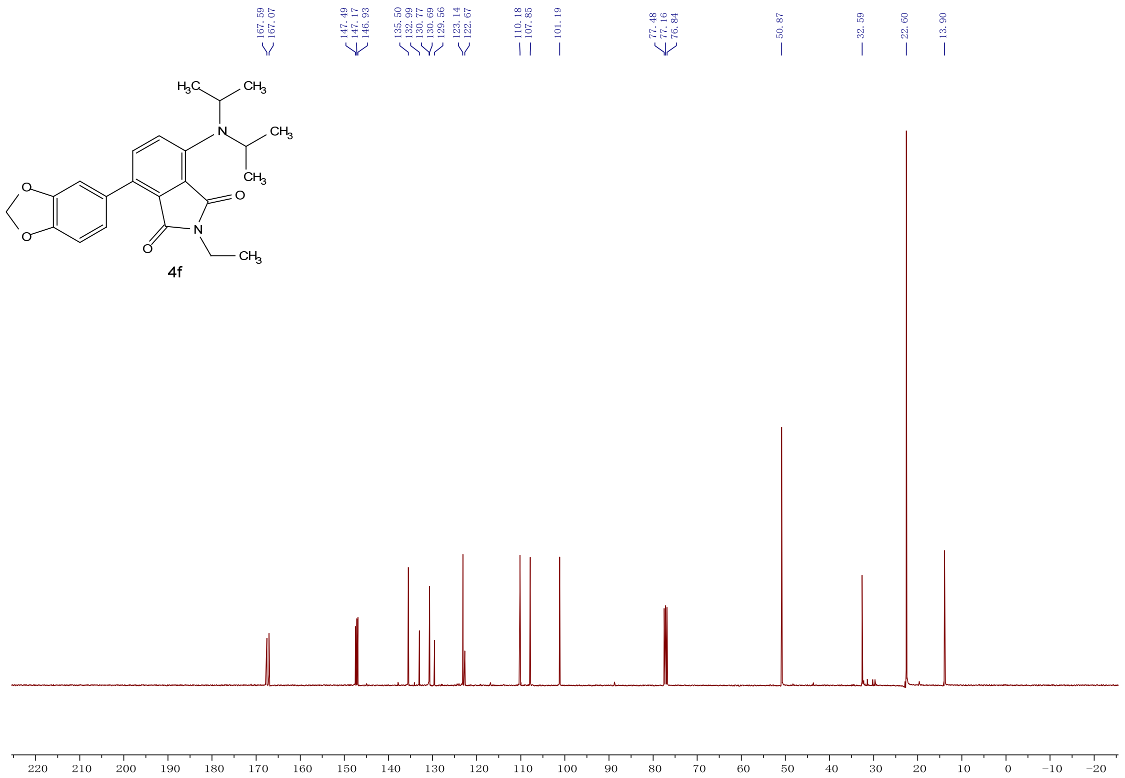

Supplementary Figure 16. ^{13}C NMR spectrum of 4f

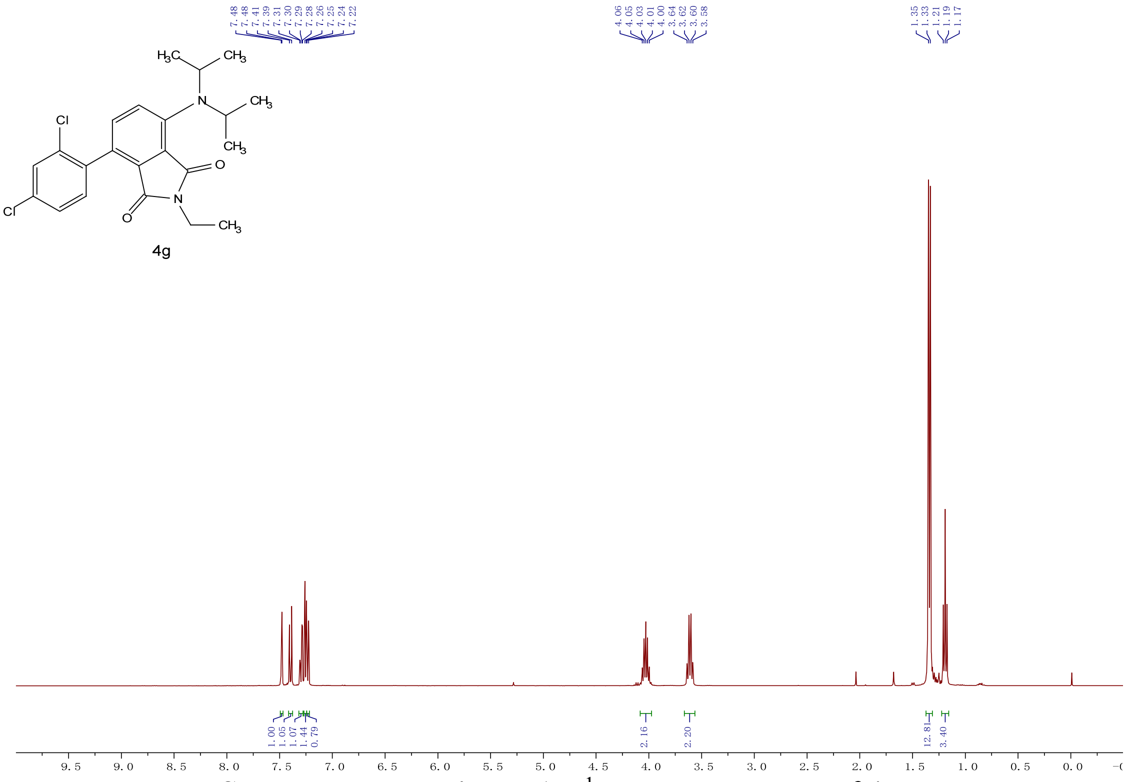

Supplementary Figure 17. ^{1}H NMR spectrum of 4g

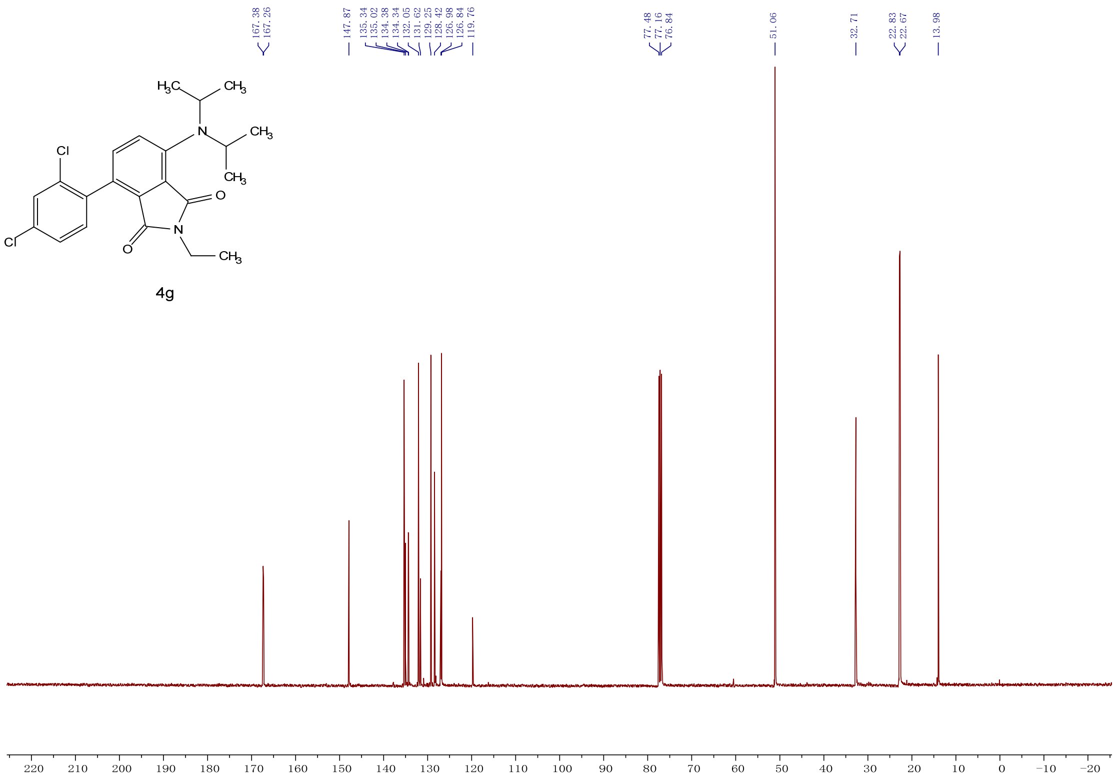

Supplementary Figure 18. ^{13}C NMR spectrum of 4g

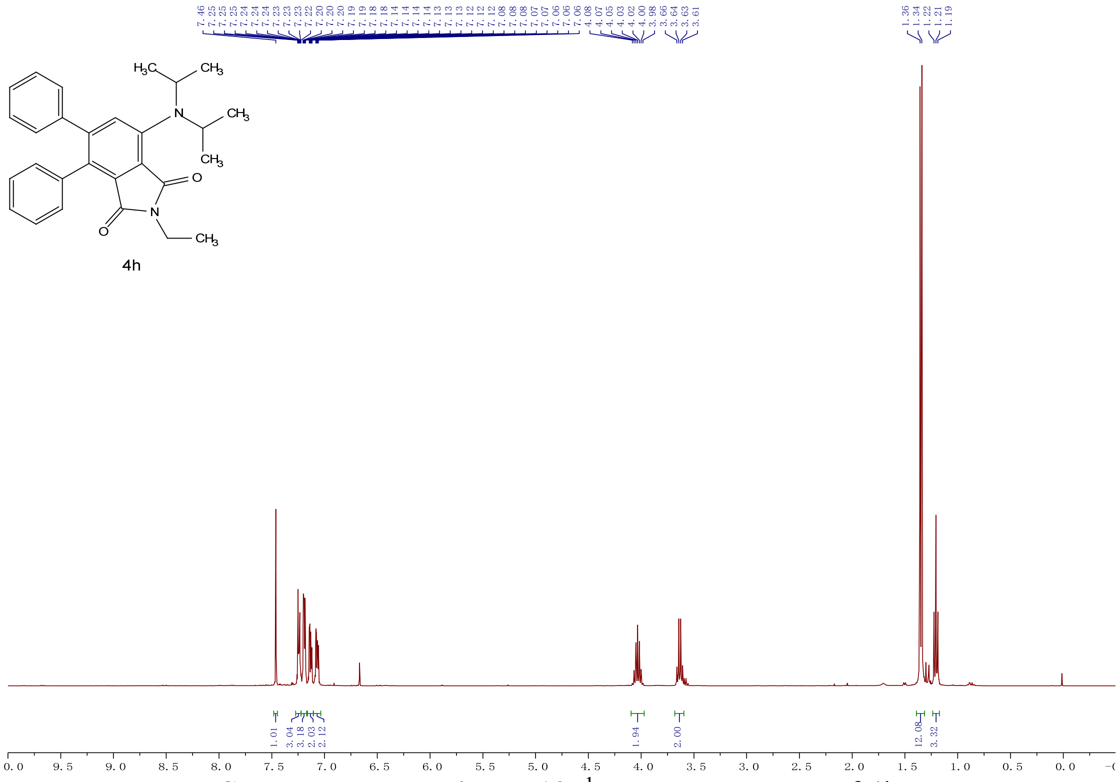

Supplementary Figure 19. ^{1}H NMR spectrum of 4h

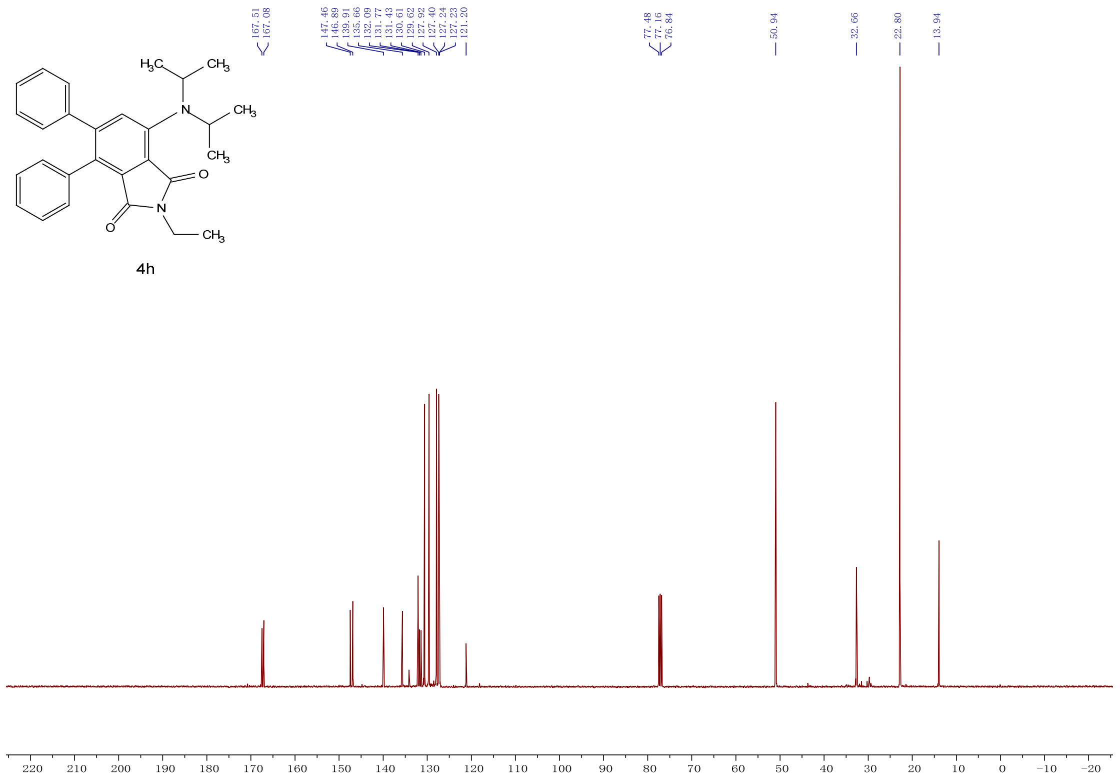

Supplementary Figure 20. ^{13}C NMR spectrum of 4h

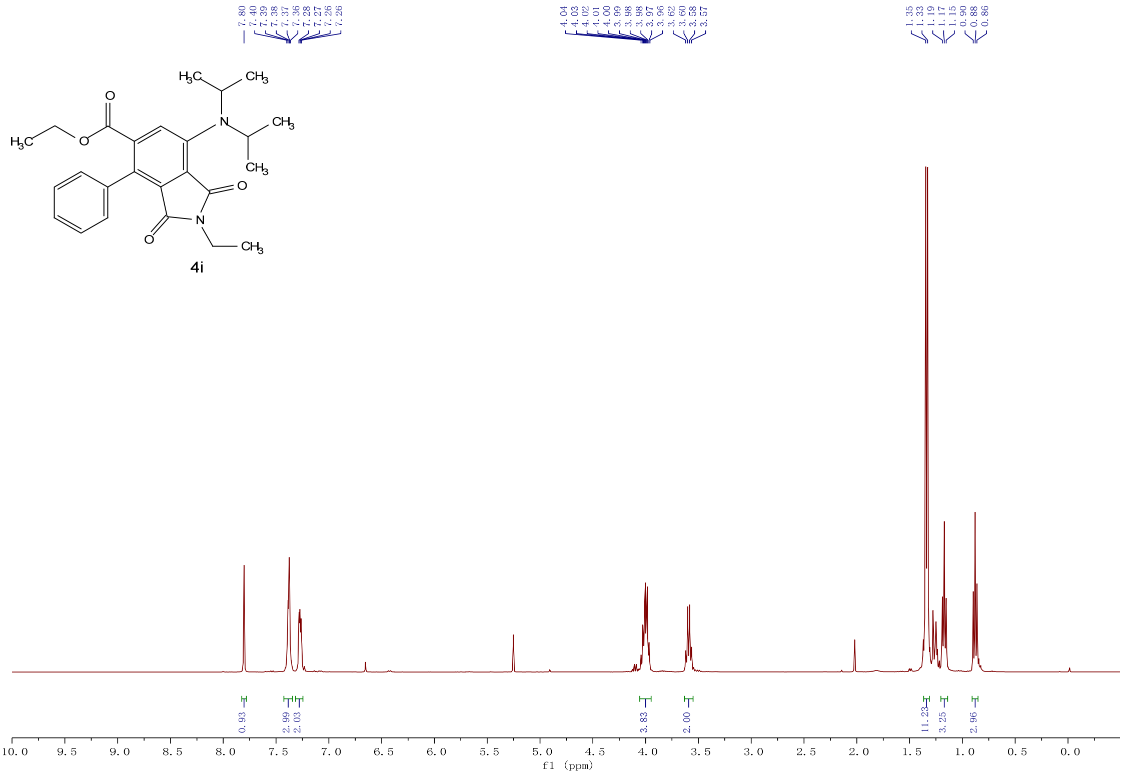

Supplementary Figure 21. ^{1}H NMR spectrum of 4i

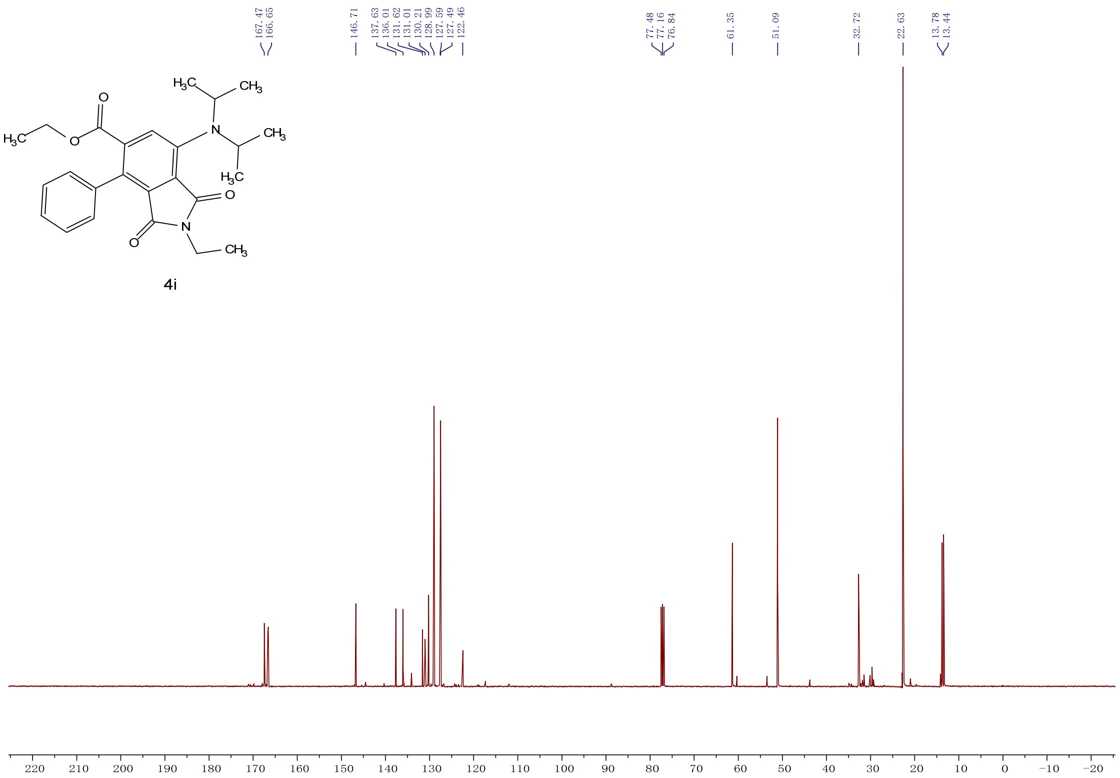

Supplementary Figure 22. ^{13}C NMR spectrum of 4i

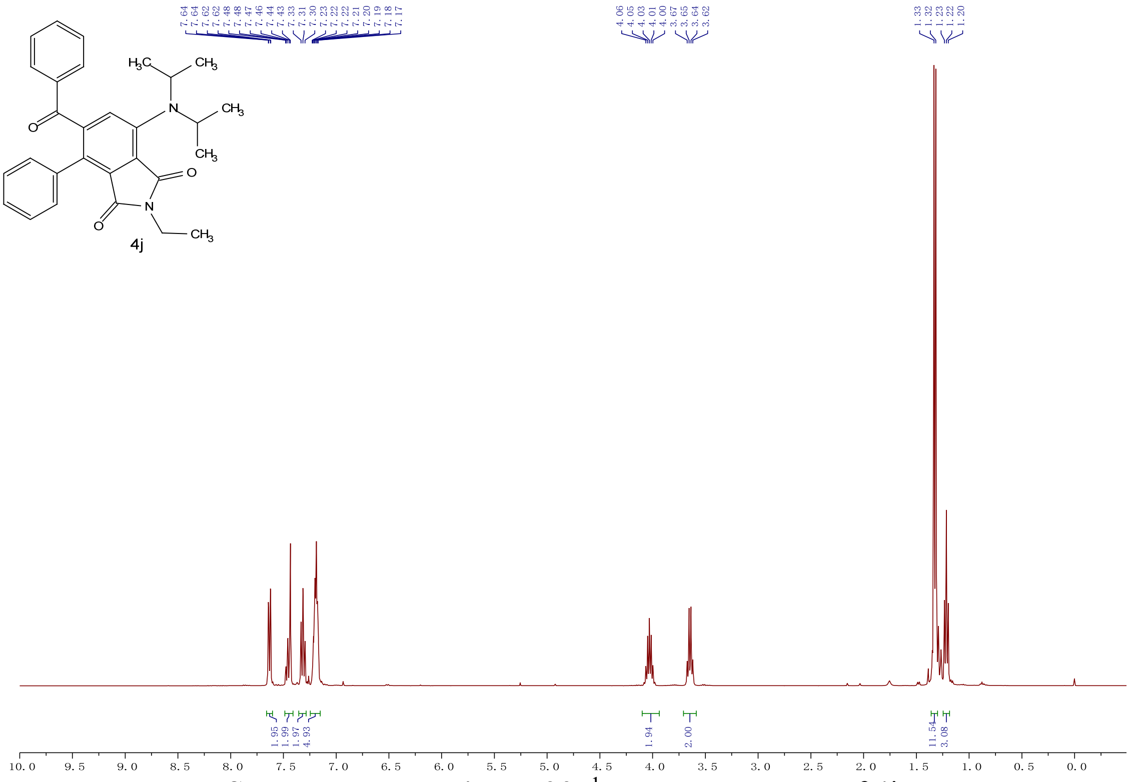

Supplementary Figure 23. ^{1}H NMR spectrum of 4j

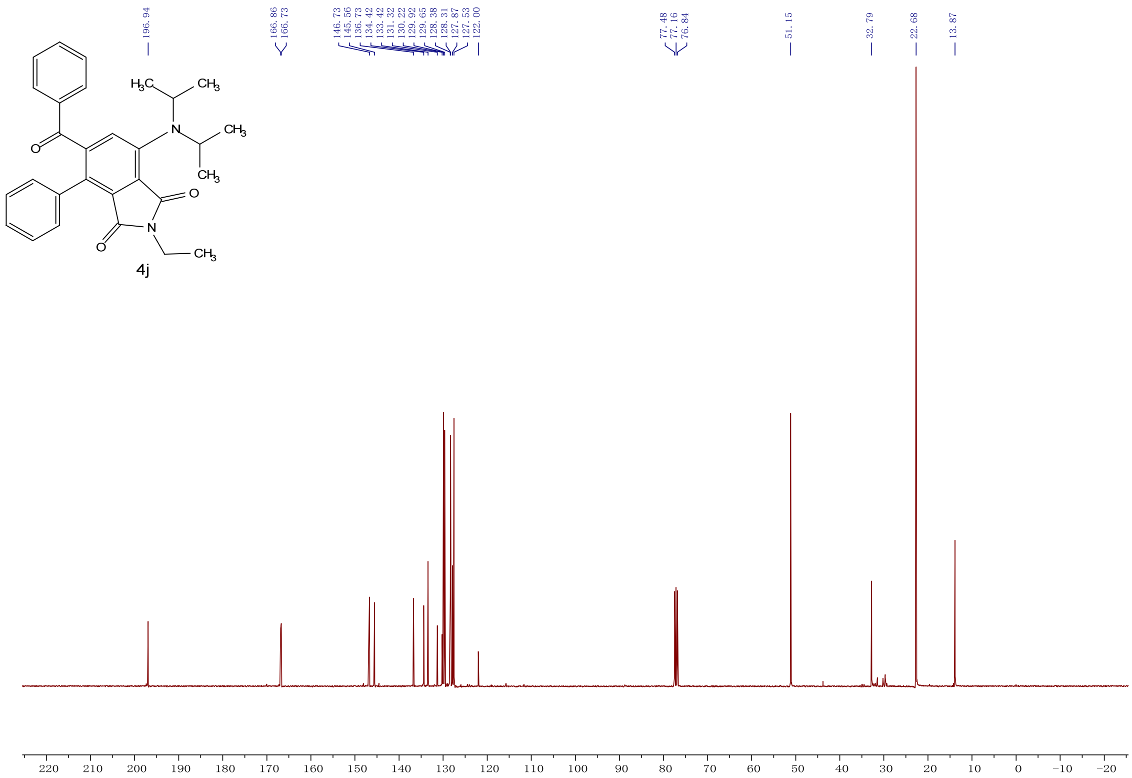

Supplementary Figure 24. ^{13}C NMR spectrum of 4j

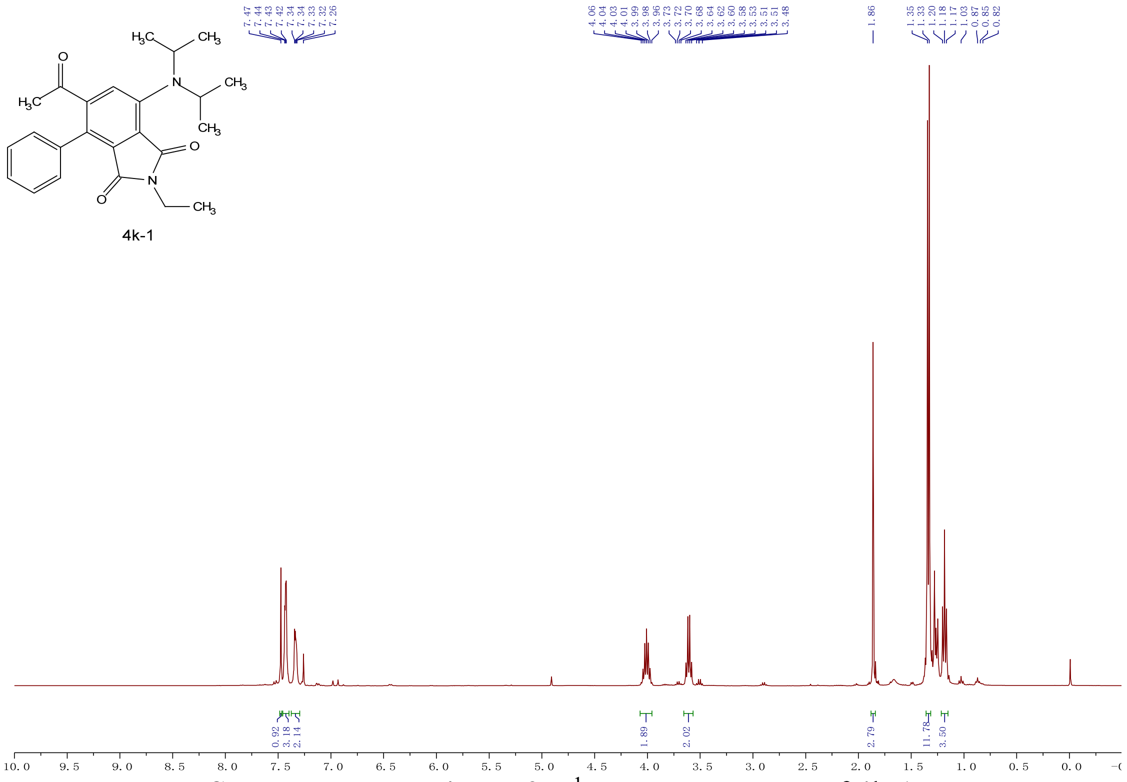

Supplementary Figure 25. ^{1}H NMR spectrum of 4k-1

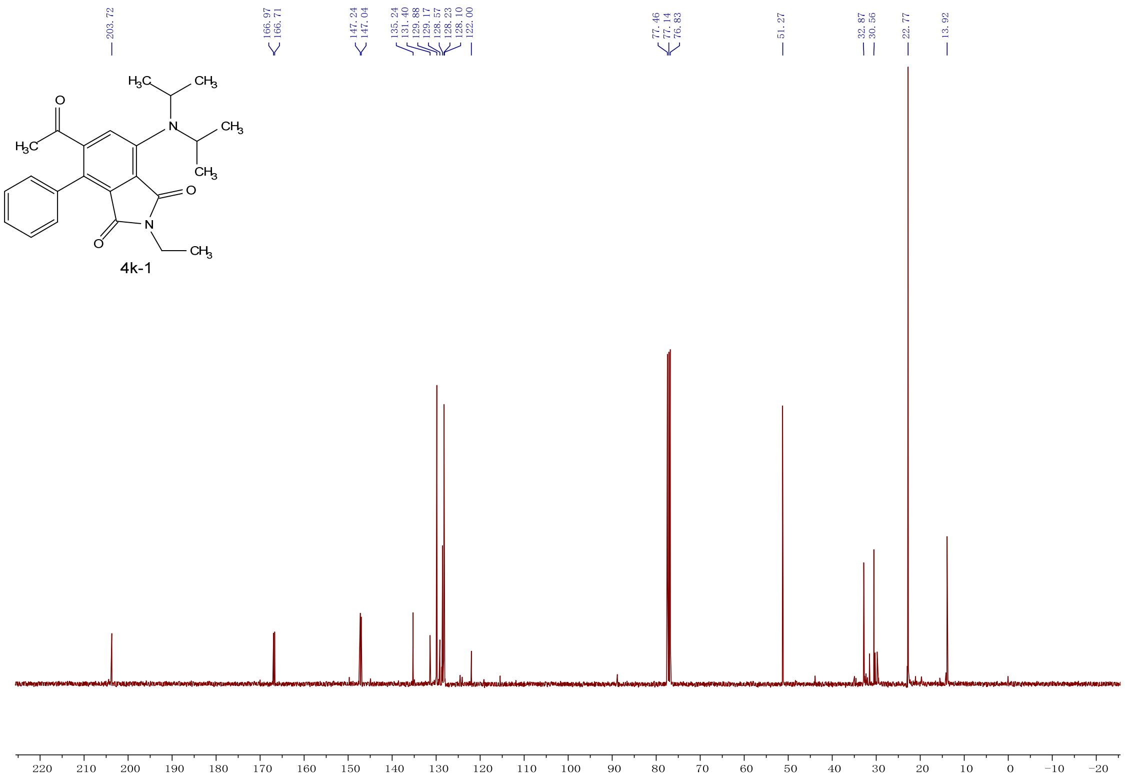

Supplementary Figure 26. ^{13}C NMR spectrum of 4k-1

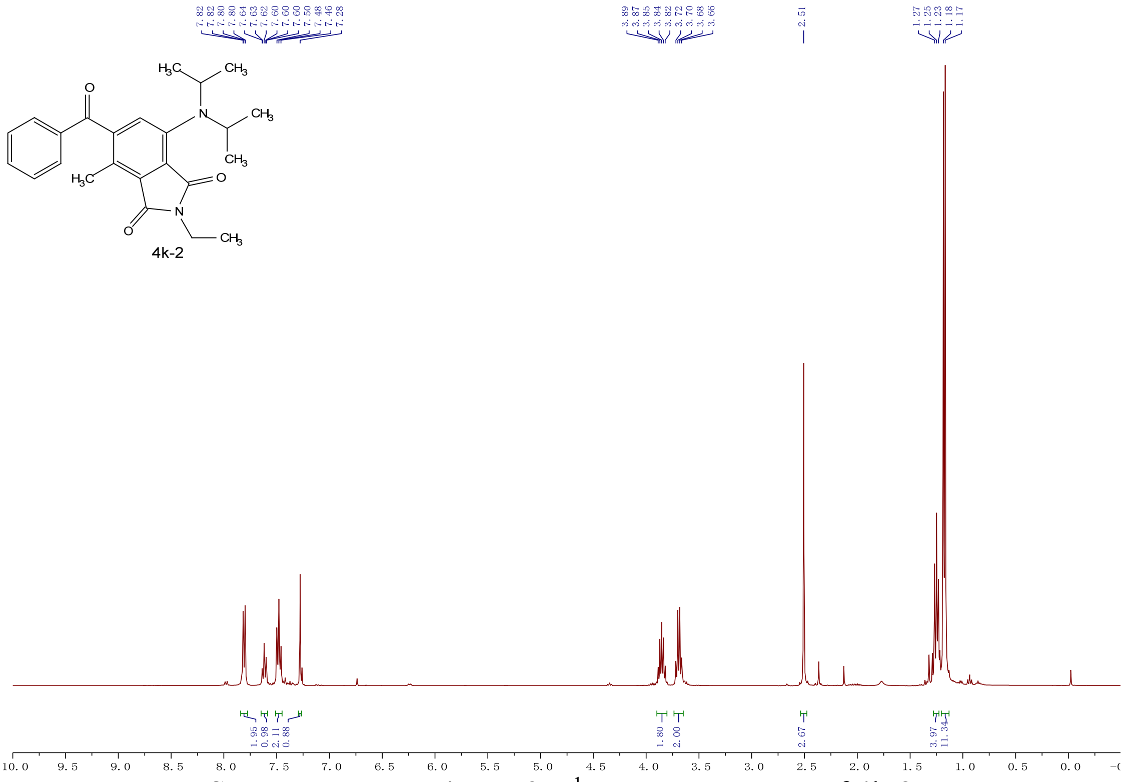

Supplementary Figure 27. ^{1}H NMR spectrum of 4k-2

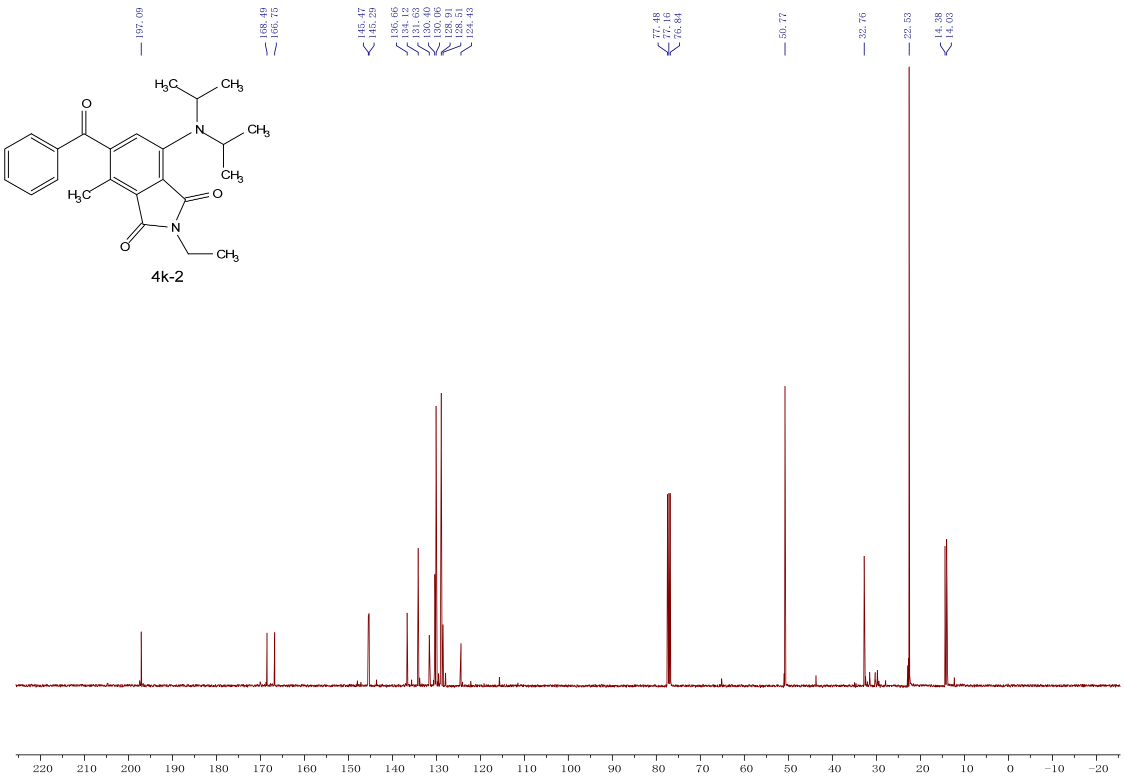

Supplementary Figure 28. ^{13}C NMR spectrum of 4k-2

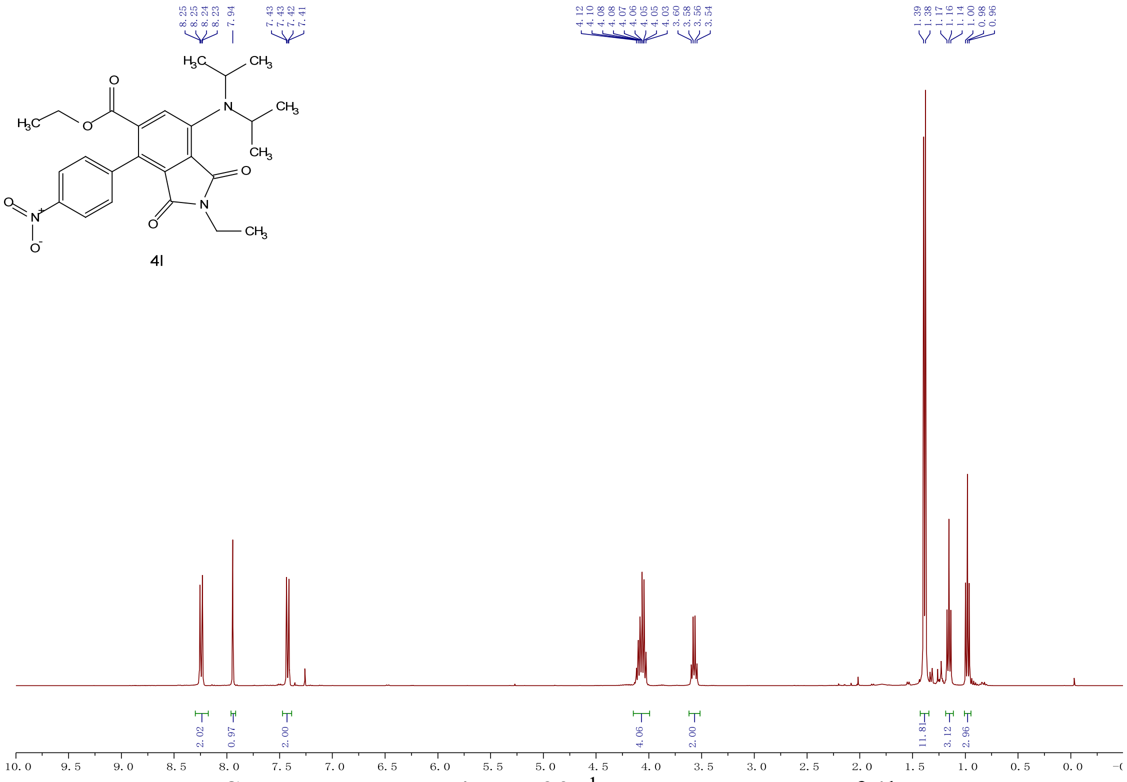

Supplementary Figure 29. ^{1}H NMR spectrum of 4l

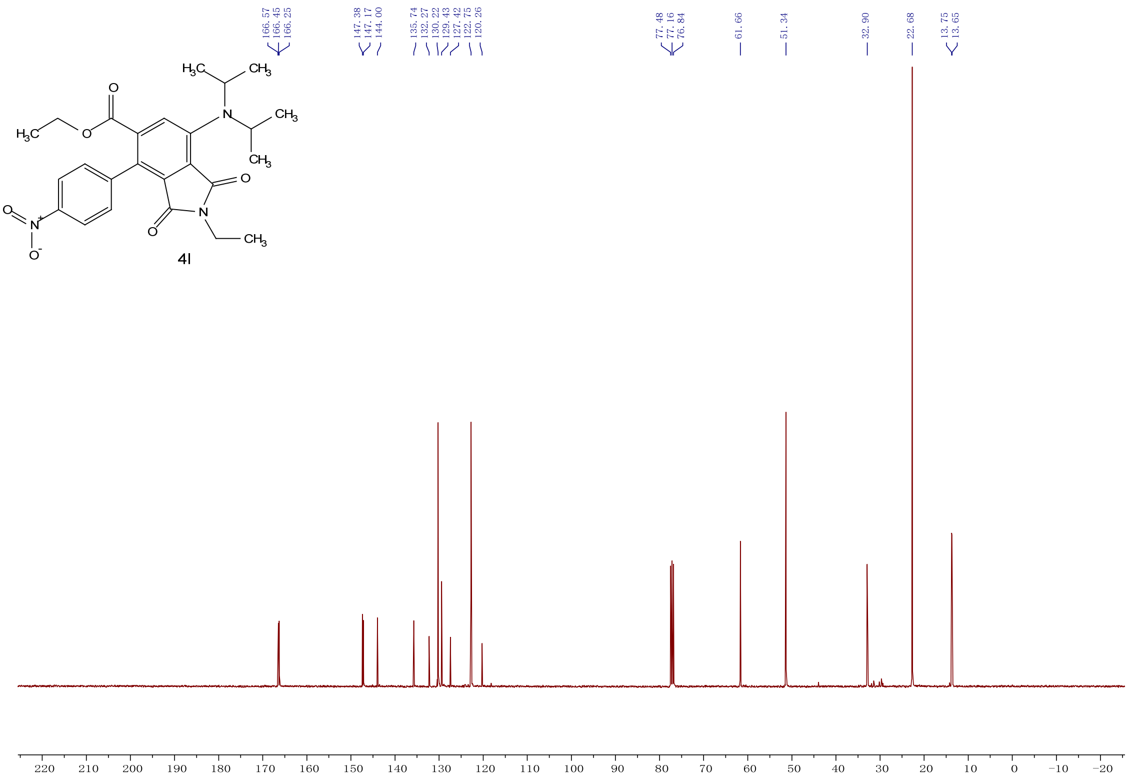

Supplementary Figure 30. ^{13}C NMR spectrum of 4l

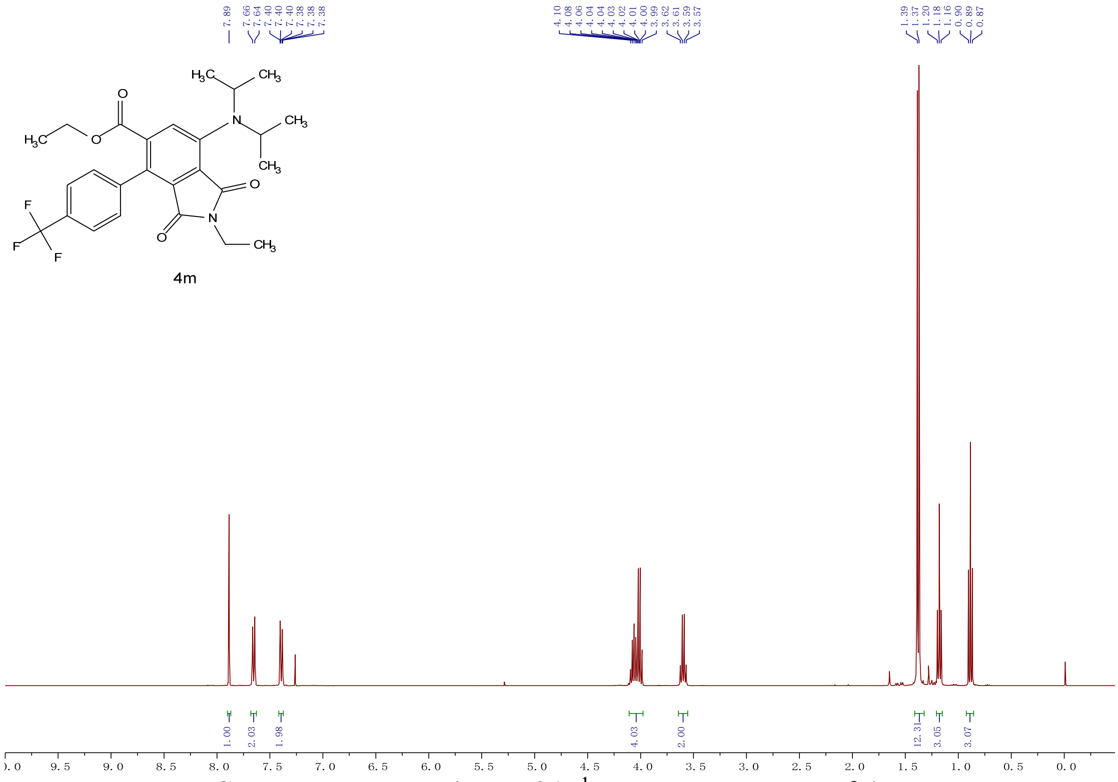

Supplementary Figure 31. ^{1}H NMR spectrum of 4m

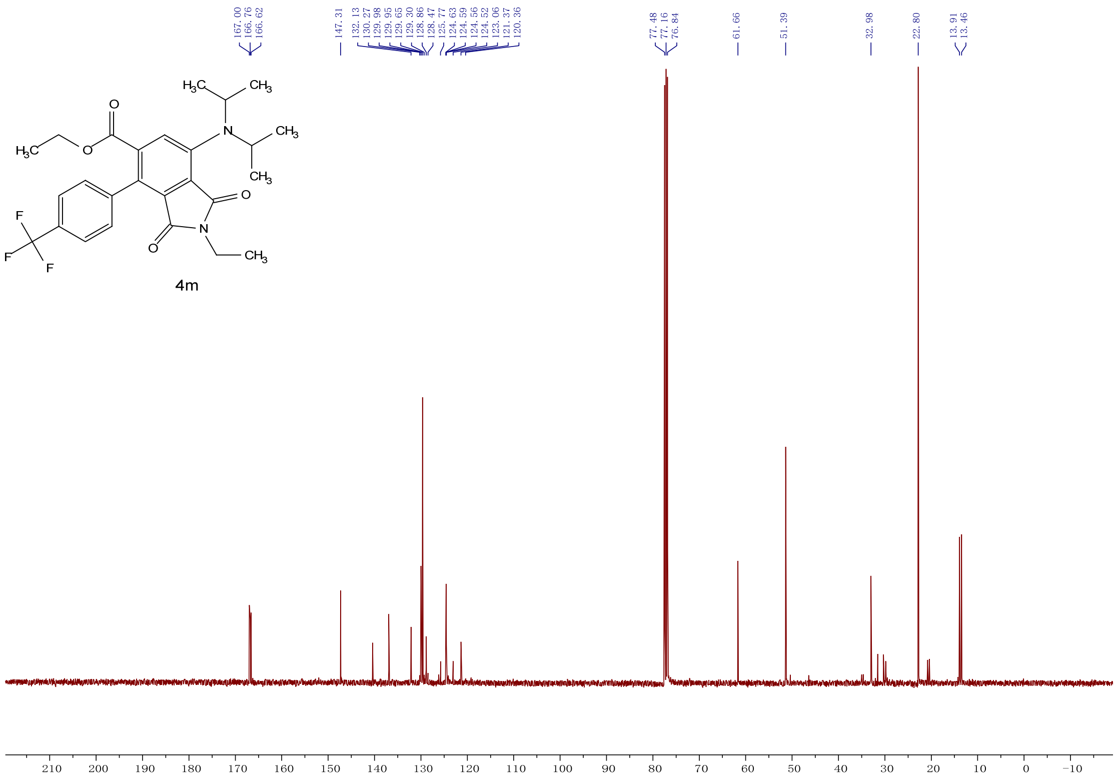

Supplementary Figure 32. ^{13}C NMR spectrum of 4m

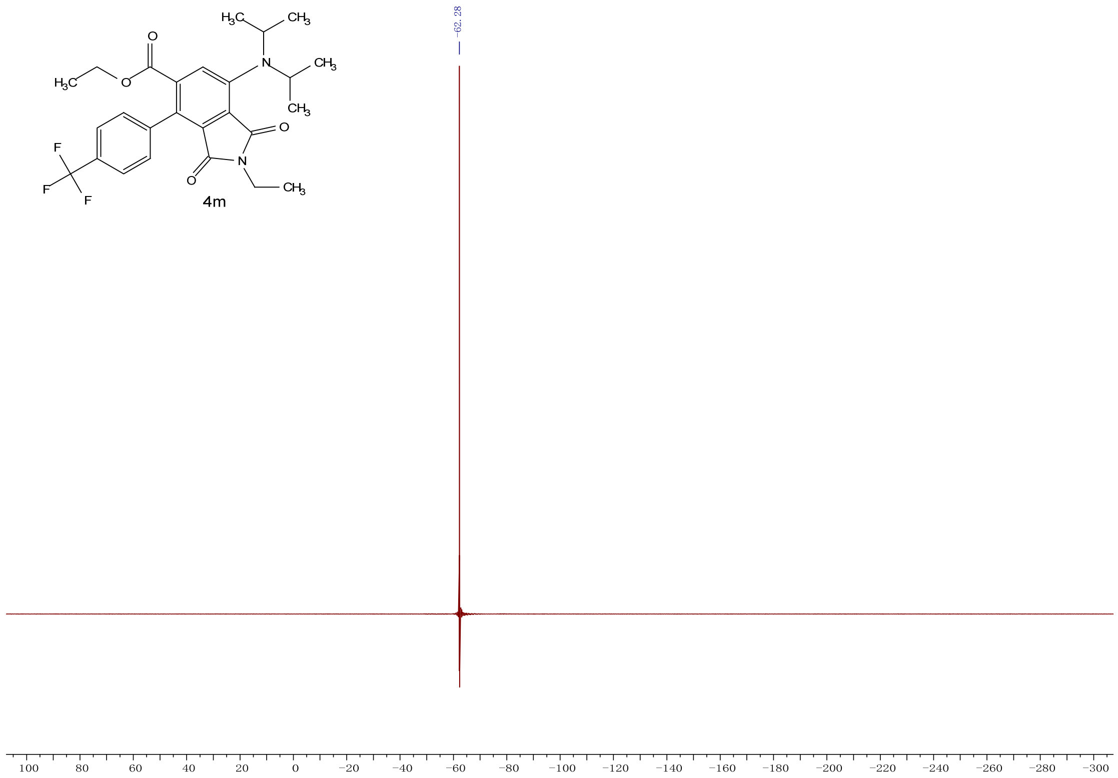

Supplementary Figure 33. ^{19}F NMR spectrum of 4m

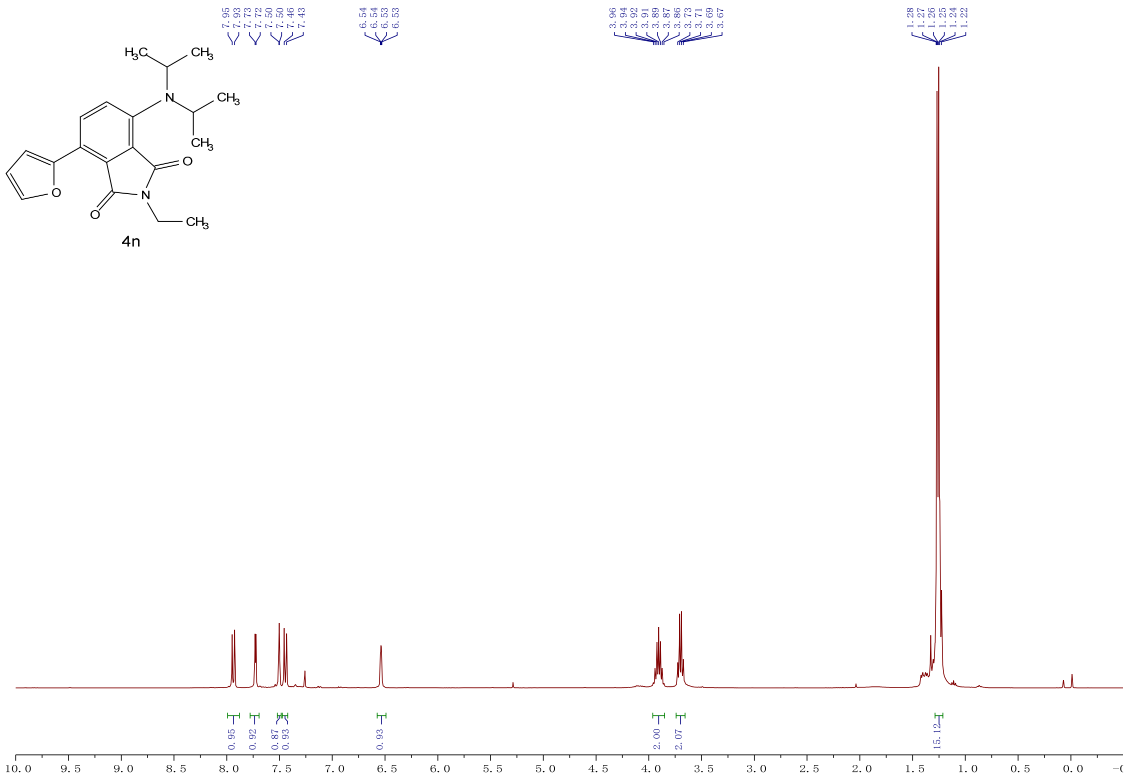

Supplementary Figure 34. ^{1}H NMR spectrum of 4n

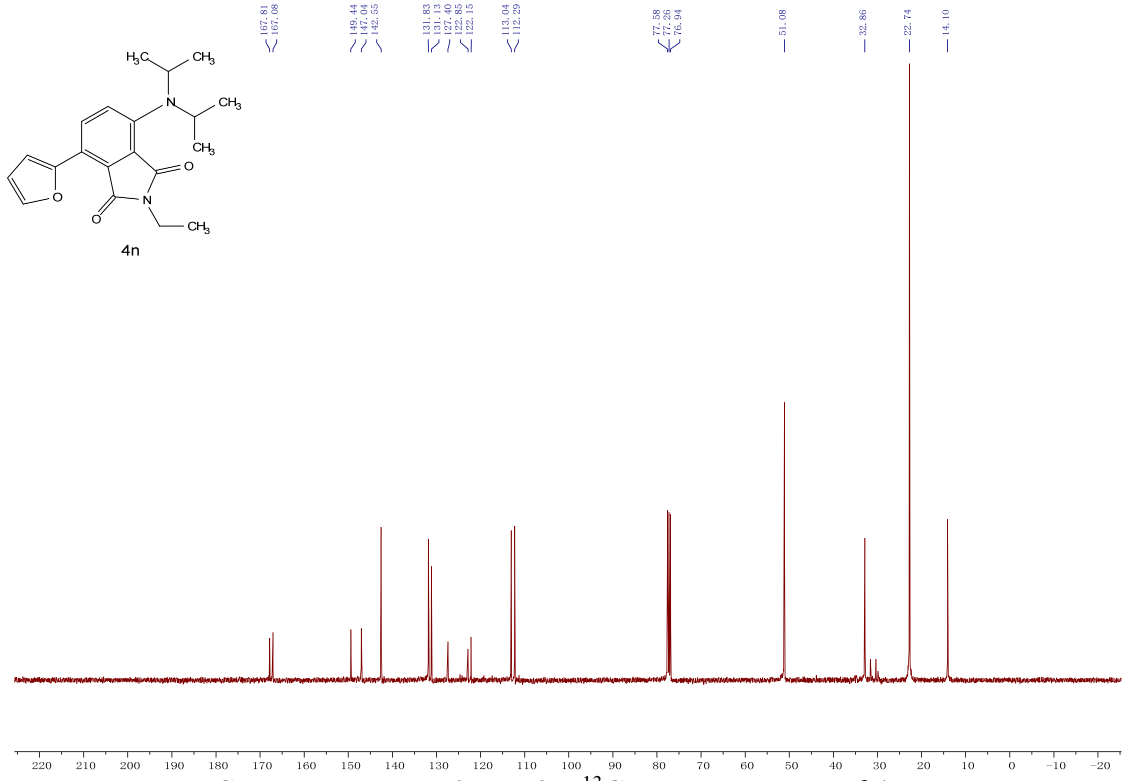

Supplementary Figure 35. ^{13}C NMR spectrum of 4n

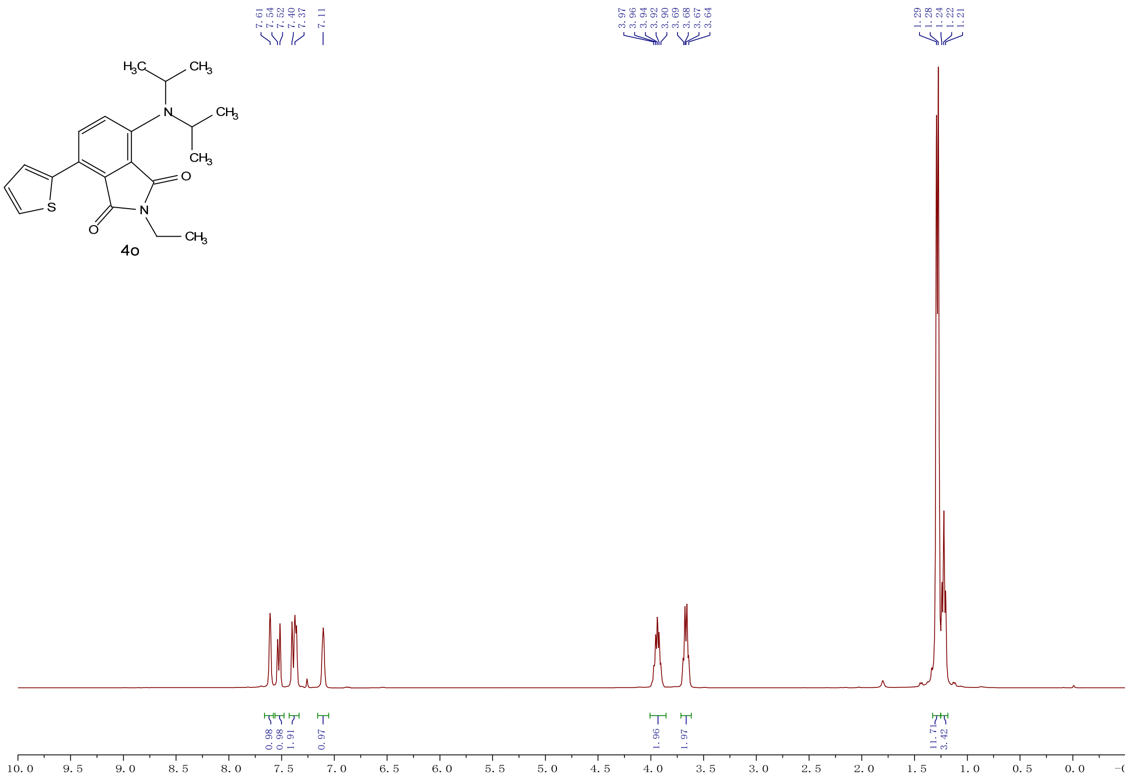

Supplementary Figure 36. ^{1}H NMR spectrum of 4o

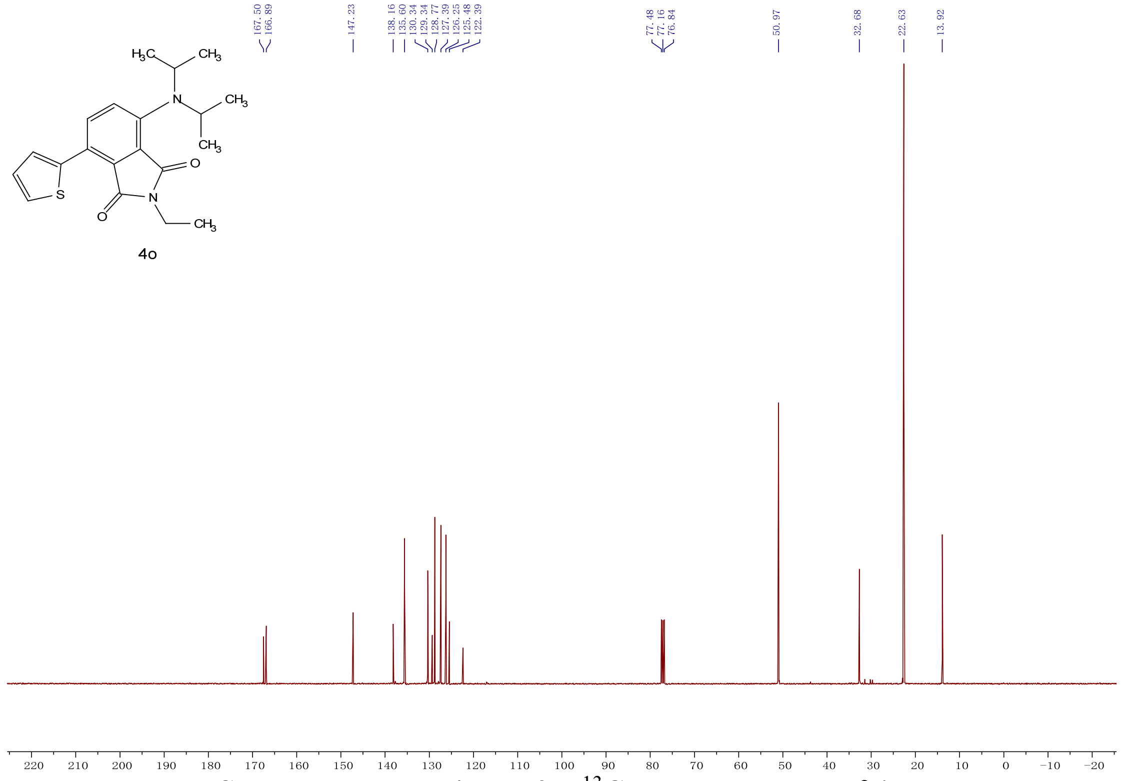

Supplementary Figure 37. ^{13}C NMR spectrum of 4o

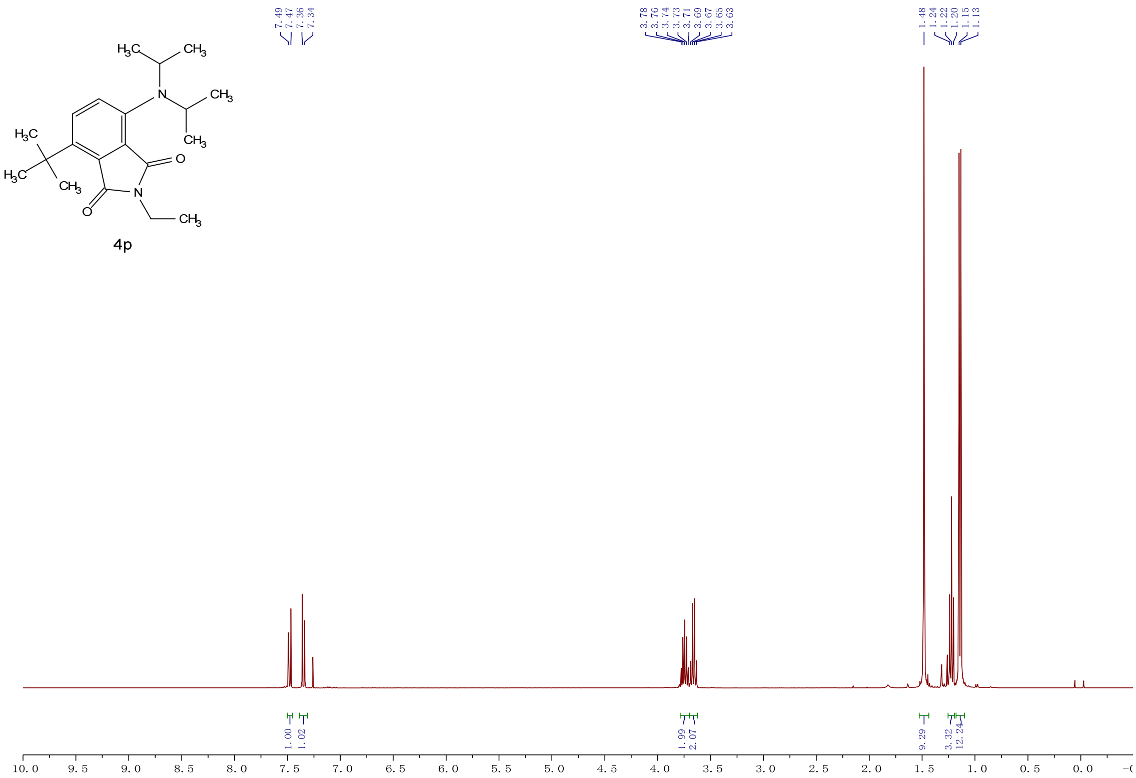

Supplementary Figure 38. ^{1}H NMR spectrum of 4p

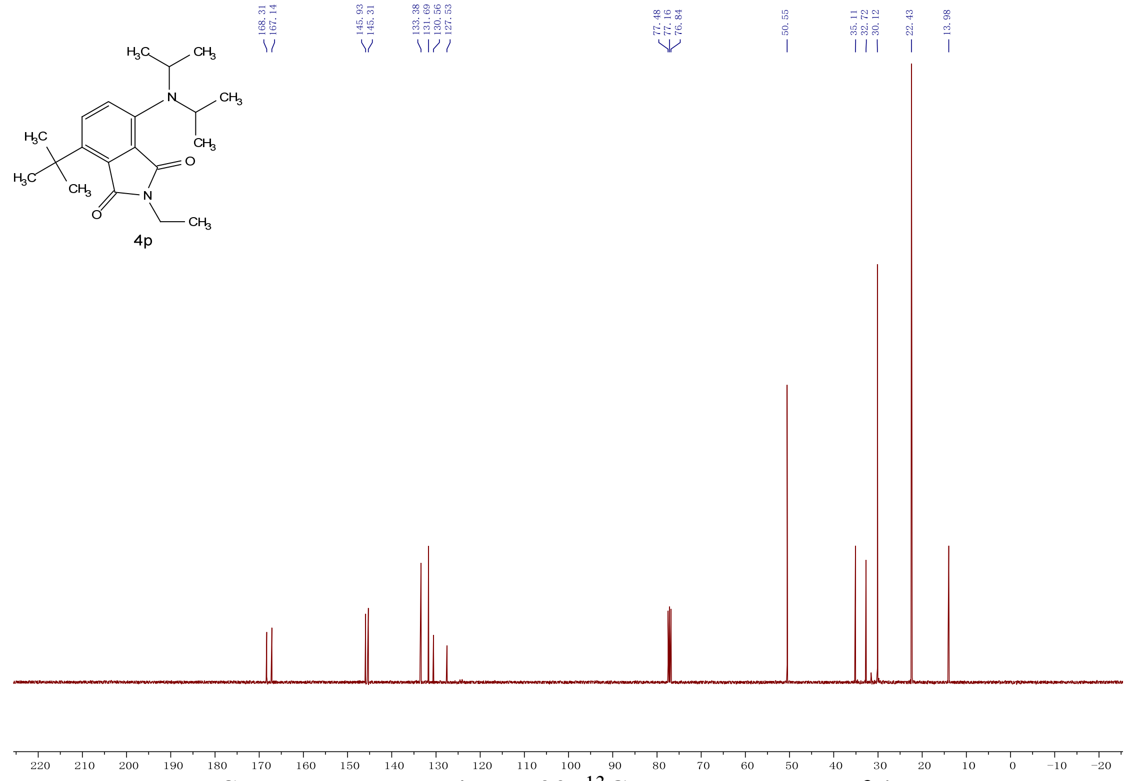

Supplementary Figure 39. ^{13}C NMR spectrum of 4p

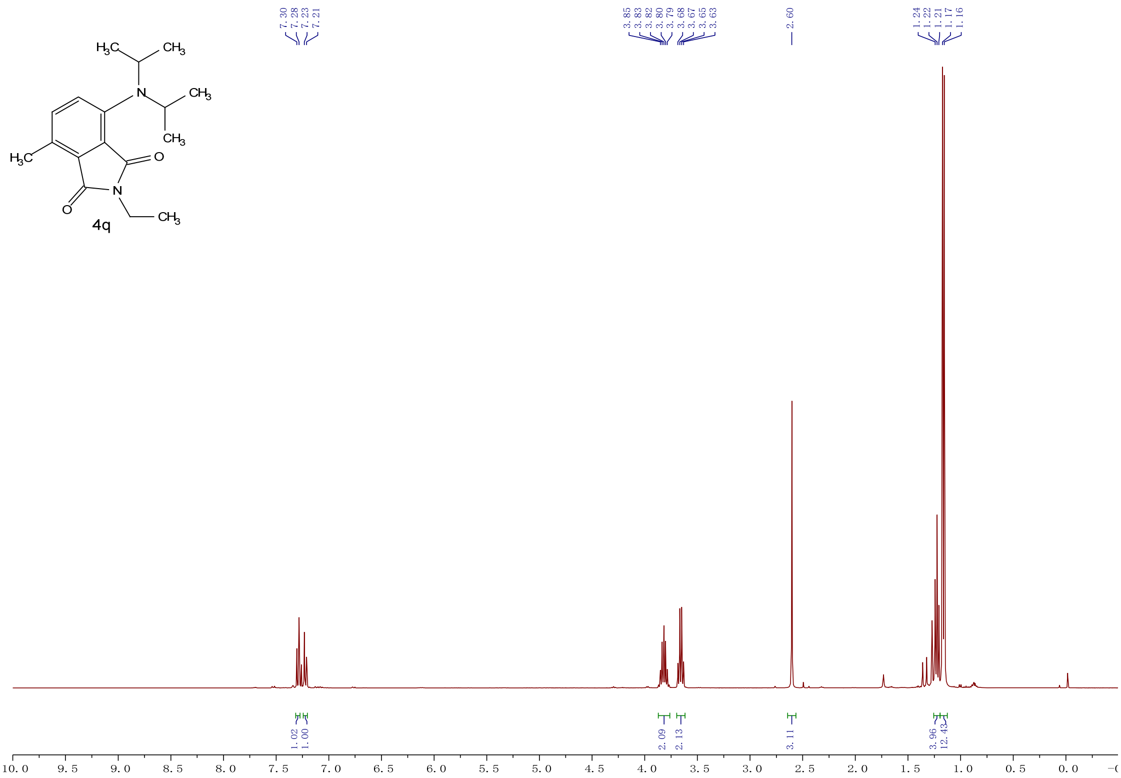

Supplementary Figure 40. ^{1}H NMR spectrum of 4q

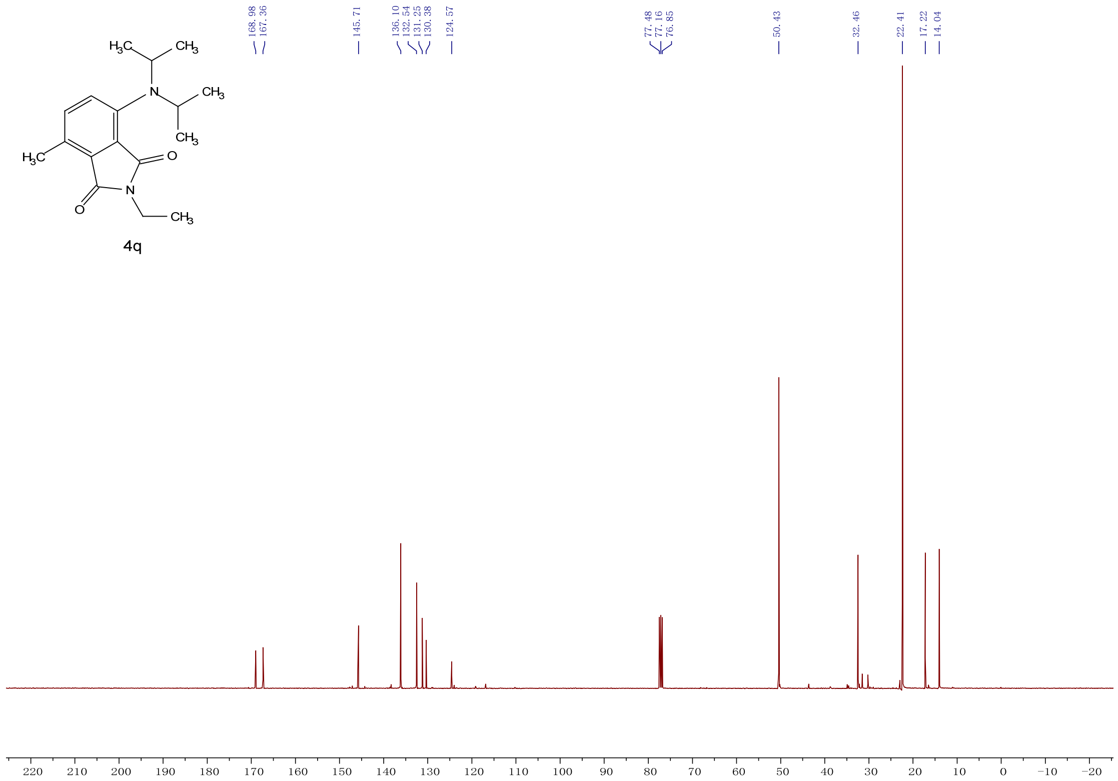

Supplementary Figure 41. ^{13}C NMR spectrum of 4q

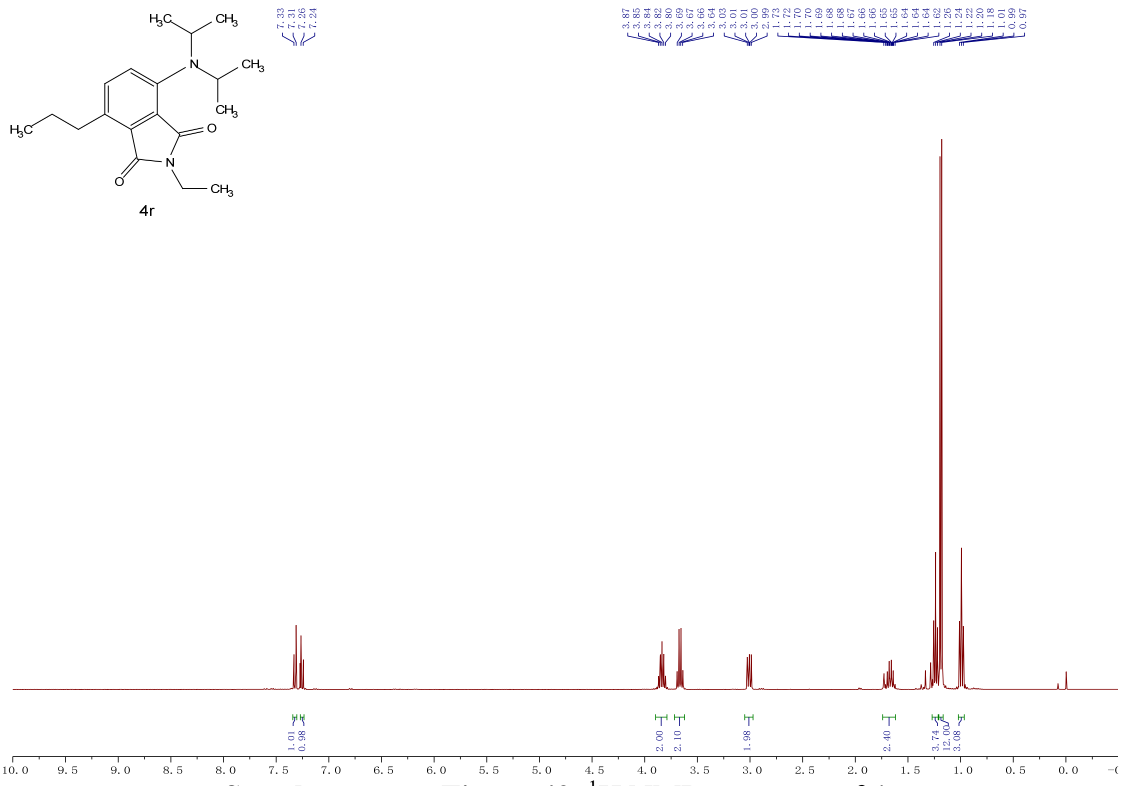

Supplementary Figure 42. ^{1}H NMR spectrum of 4r

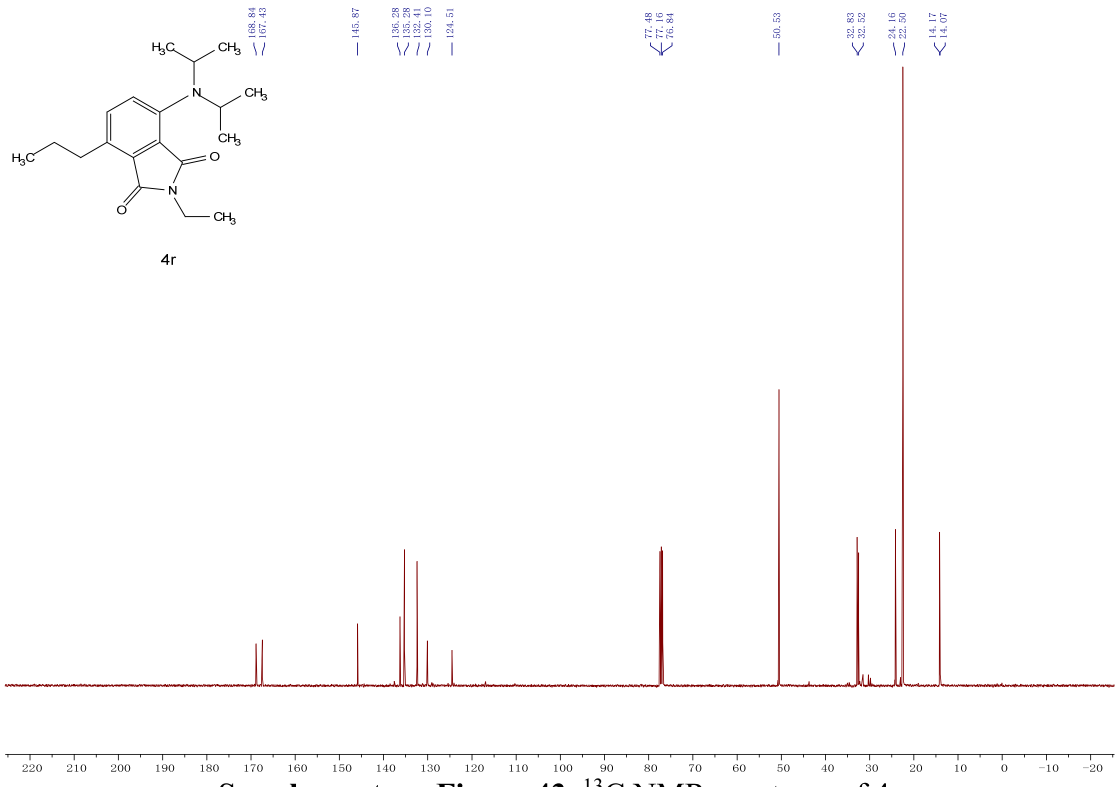

Supplementary Figure 43. ^{13}C NMR spectrum of 4r

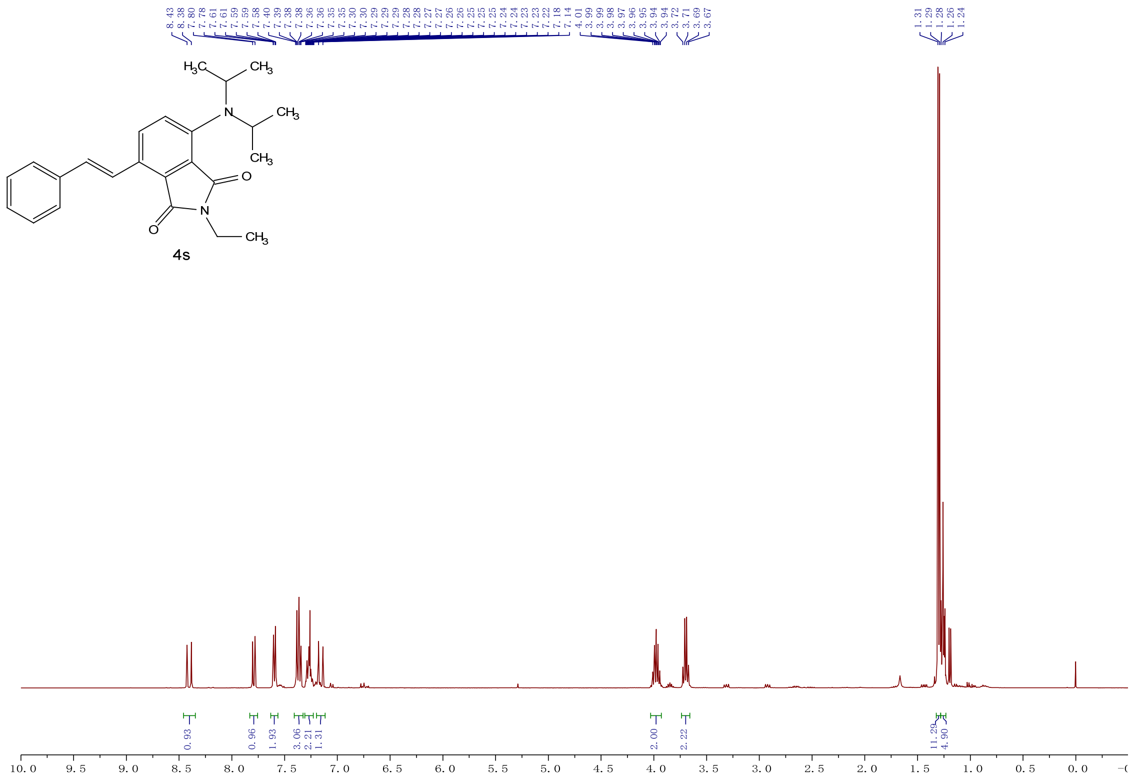

Supplementary Figure 44. ^{1}H NMR spectrum of 4s

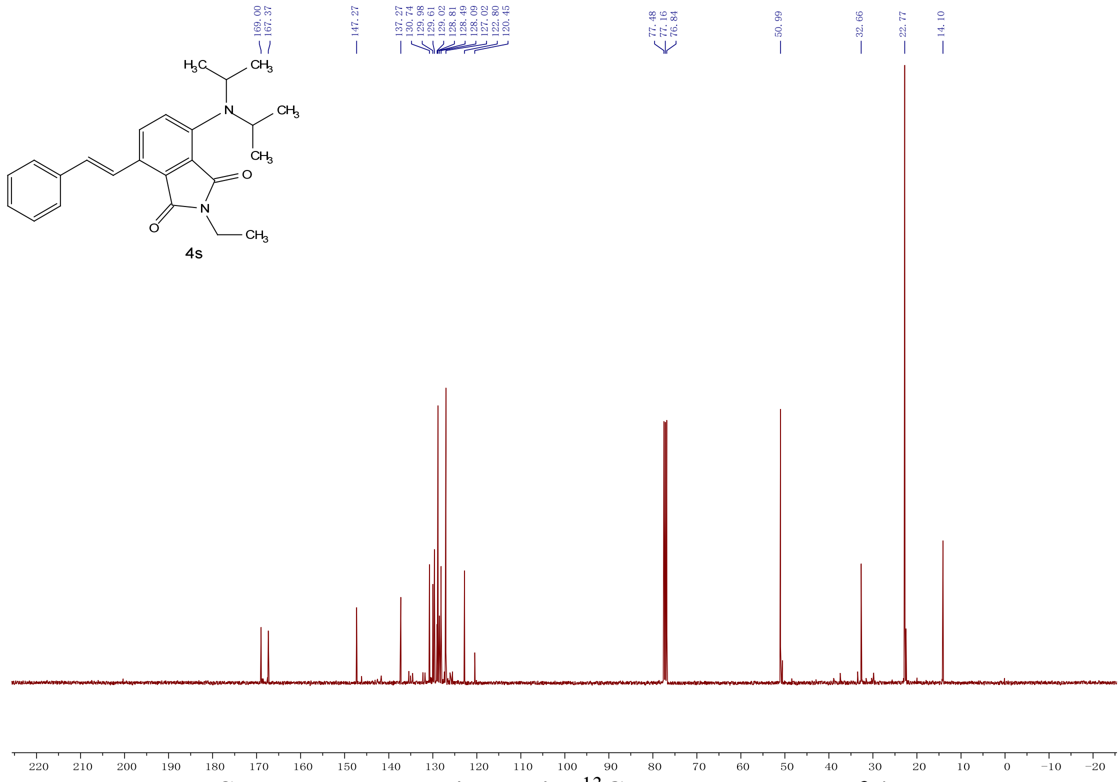

Supplementary Figure 45. ^{13}C NMR spectrum of 4s

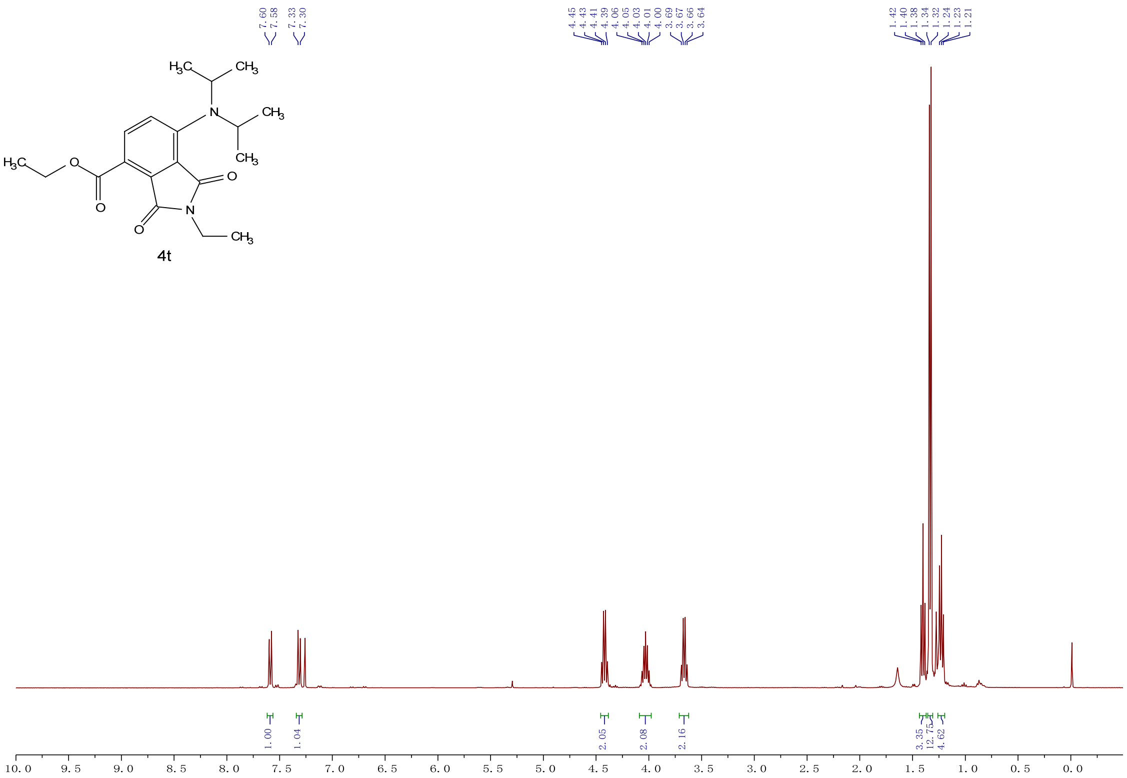

Supplementary Figure 46. ^{1}H NMR spectrum of 4t

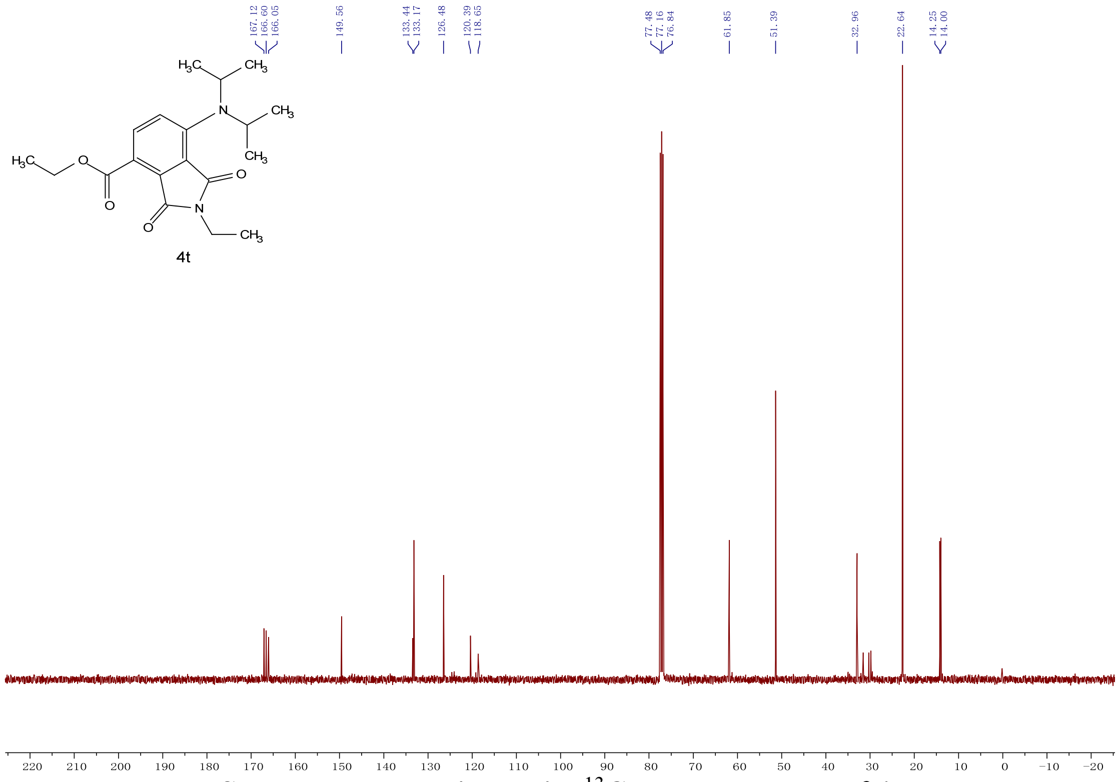

Supplementary Figure 47. ^{13}C NMR spectrum of 4t

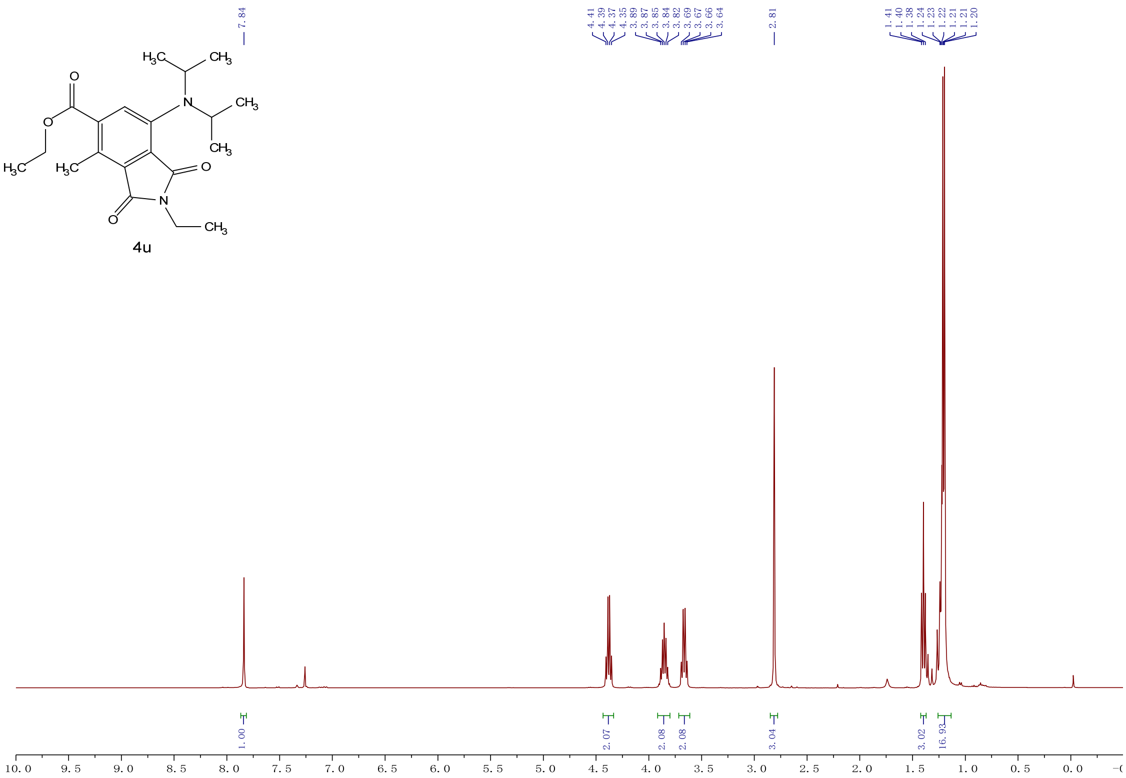

Supplementary Figure 48. ^{1}H NMR spectrum of 4u

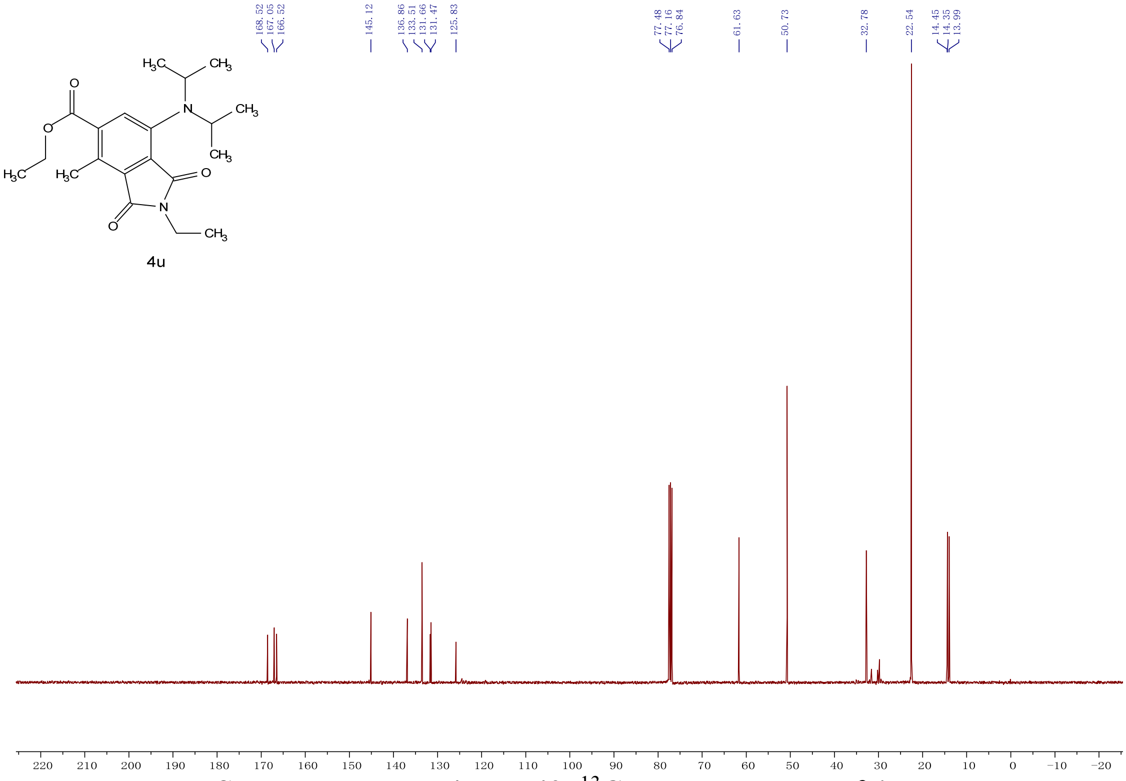

Supplementary Figure 49. ^{13}C NMR spectrum of 4u

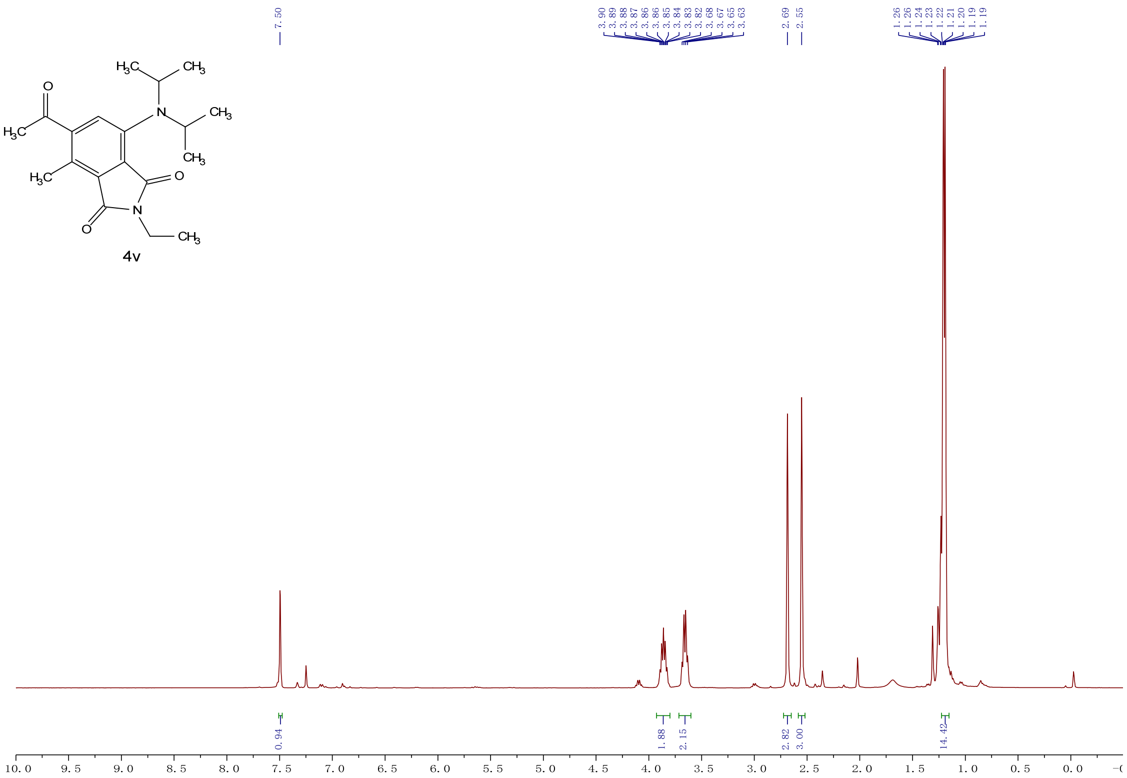

Supplementary Figure 50. ^{1}H NMR spectrum of 4v

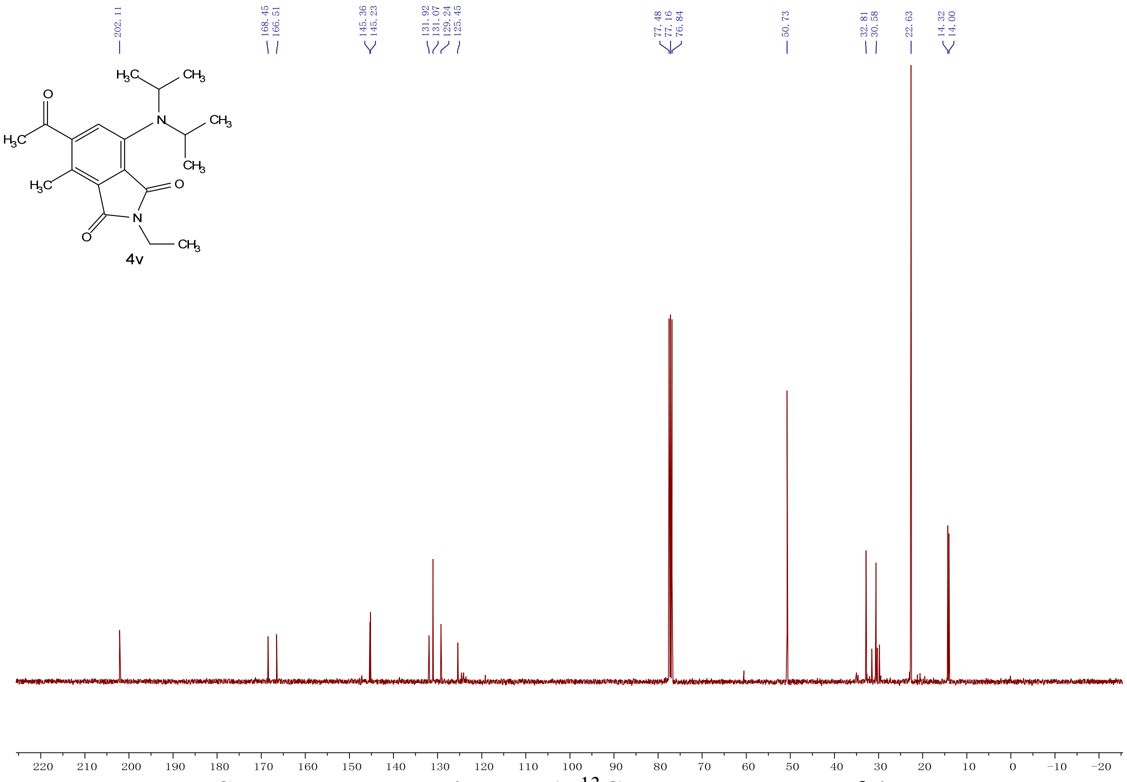

Supplementary Figure 51. ^{13}C NMR spectrum of 4v

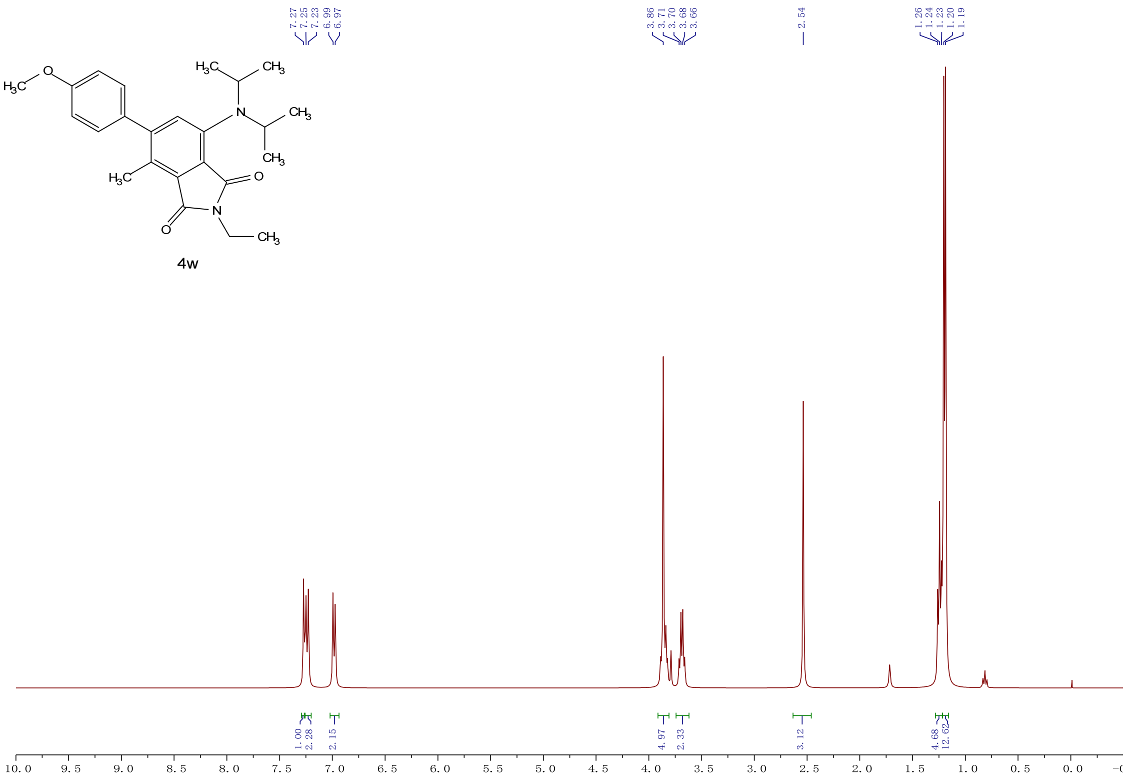

Supplementary Figure 52. ^{1}H NMR spectrum of 4w

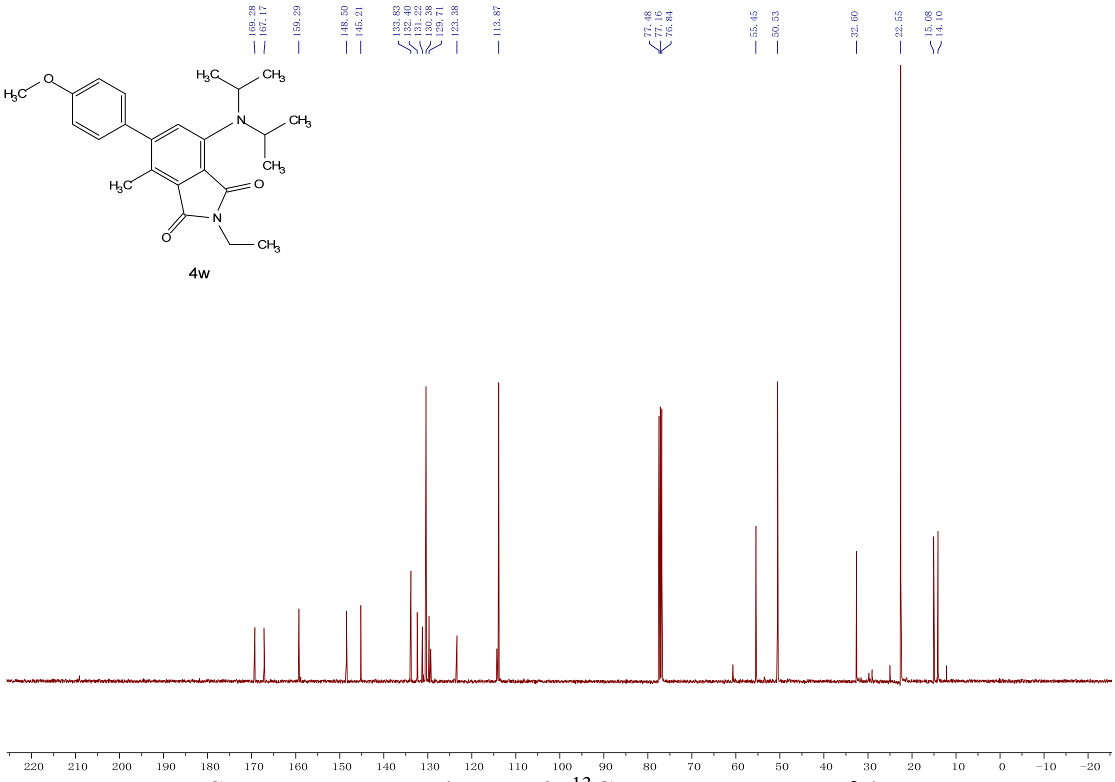

Supplementary Figure 53. ^{13}C NMR spectrum of 4w

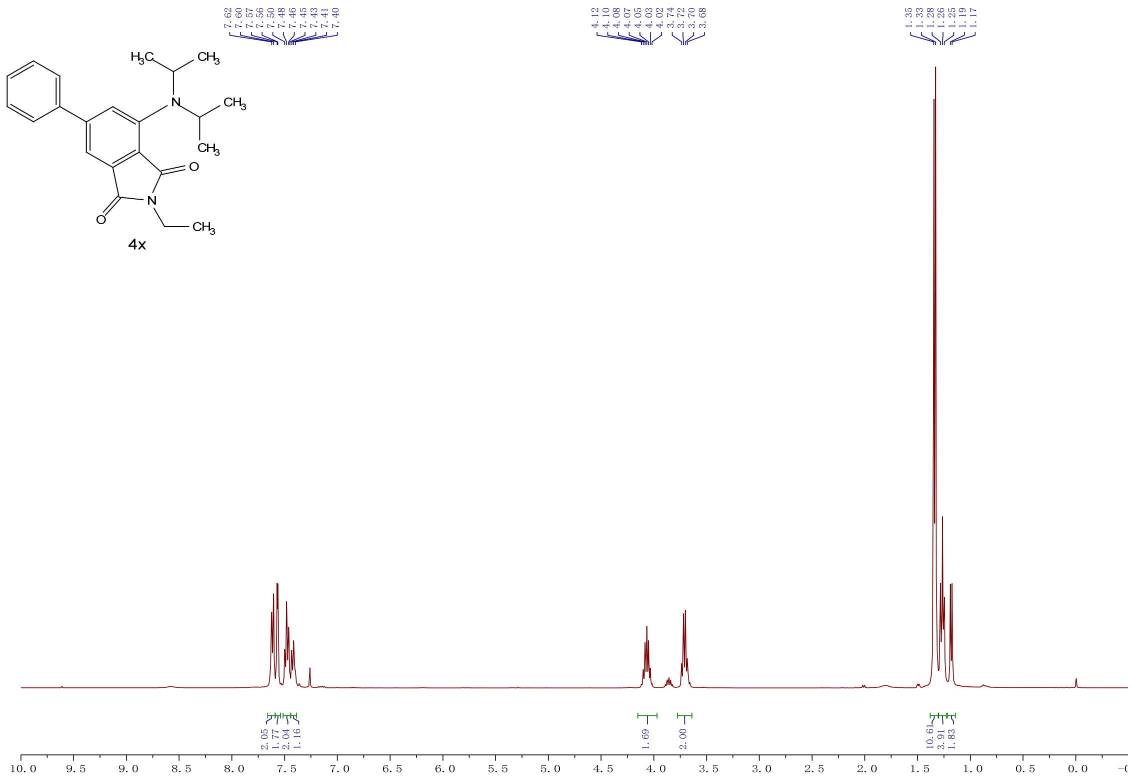

Supplementary Figure 54. ^{1}H NMR spectrum of 4x

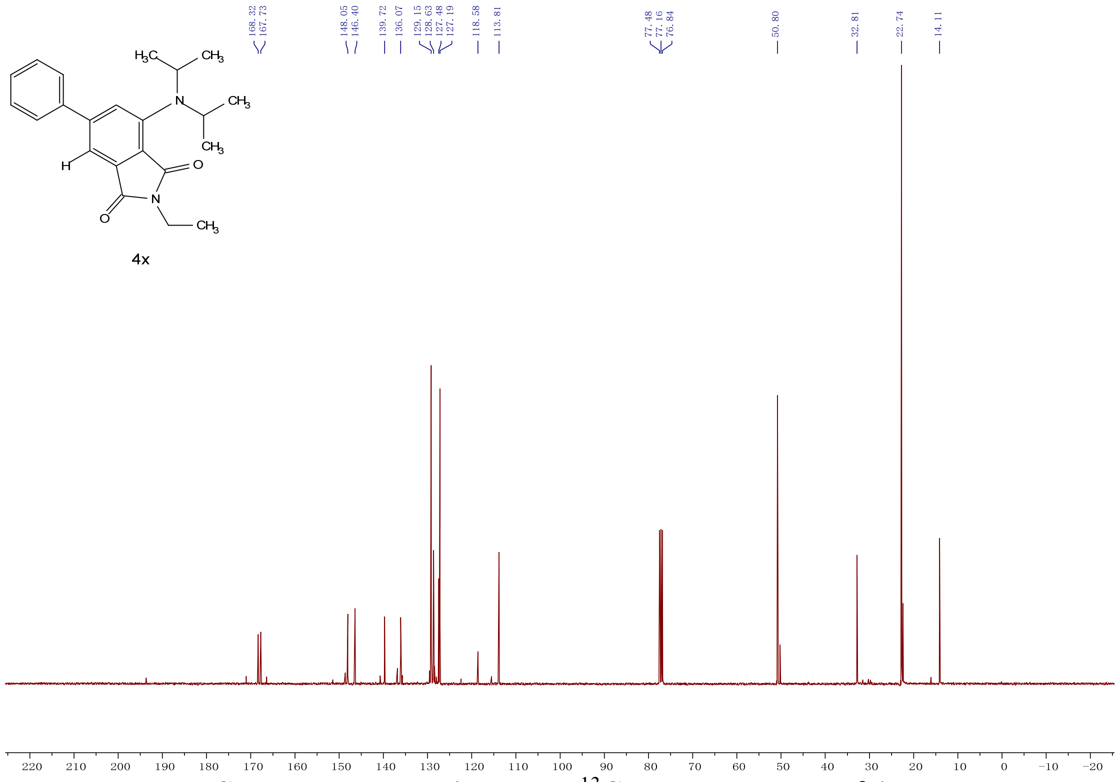

Supplementary Figure 55. ^{13}C NMR spectrum of 4x

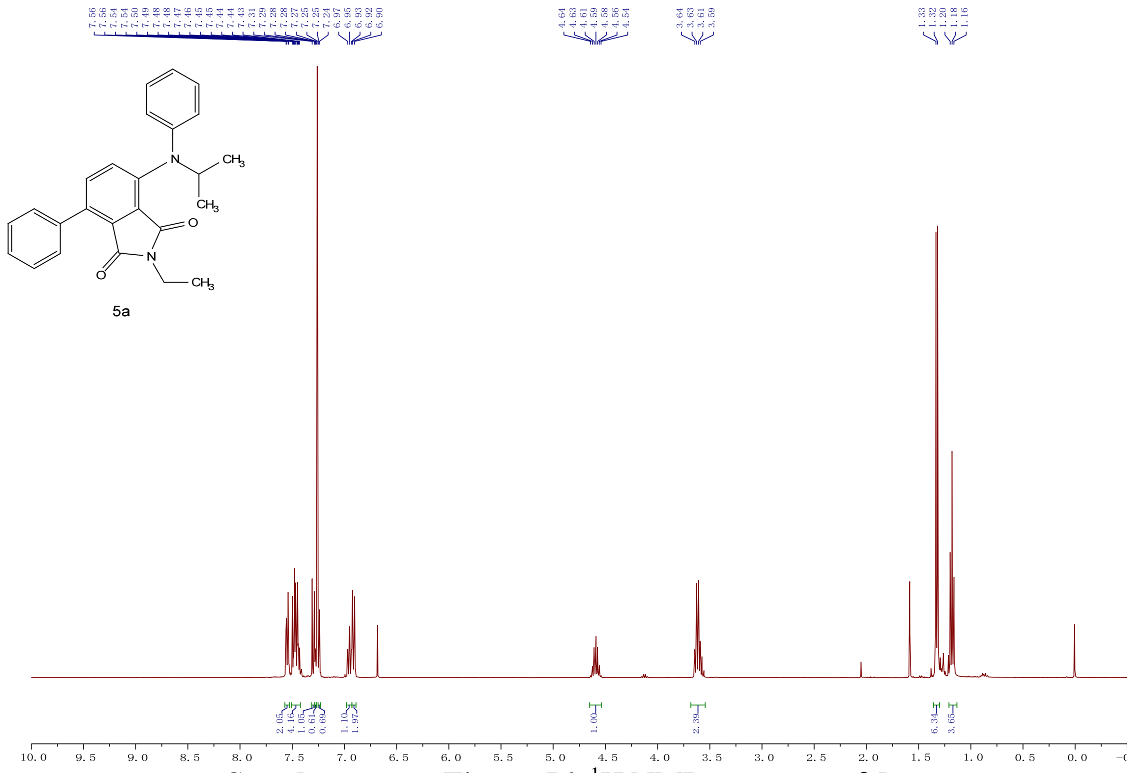

Supplementary Figure 56. ^{1}H NMR spectrum of 5a

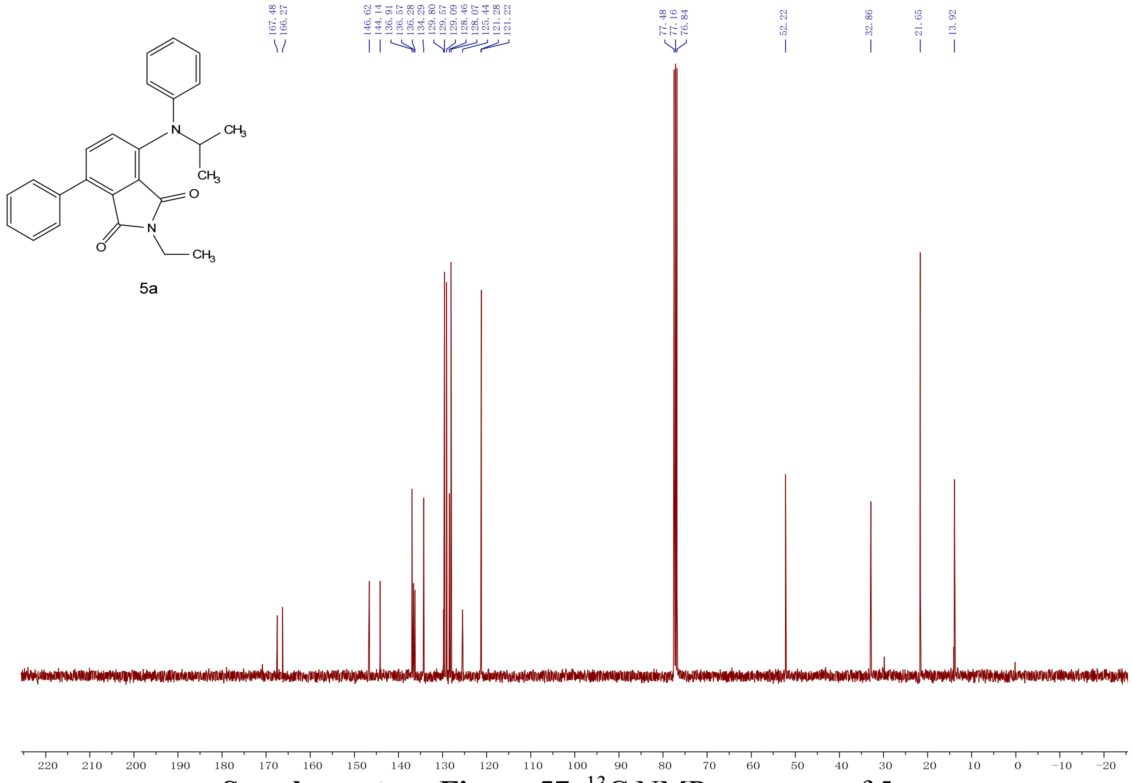

Supplementary Figure 57. ^{13}C NMR spectrum of 5a

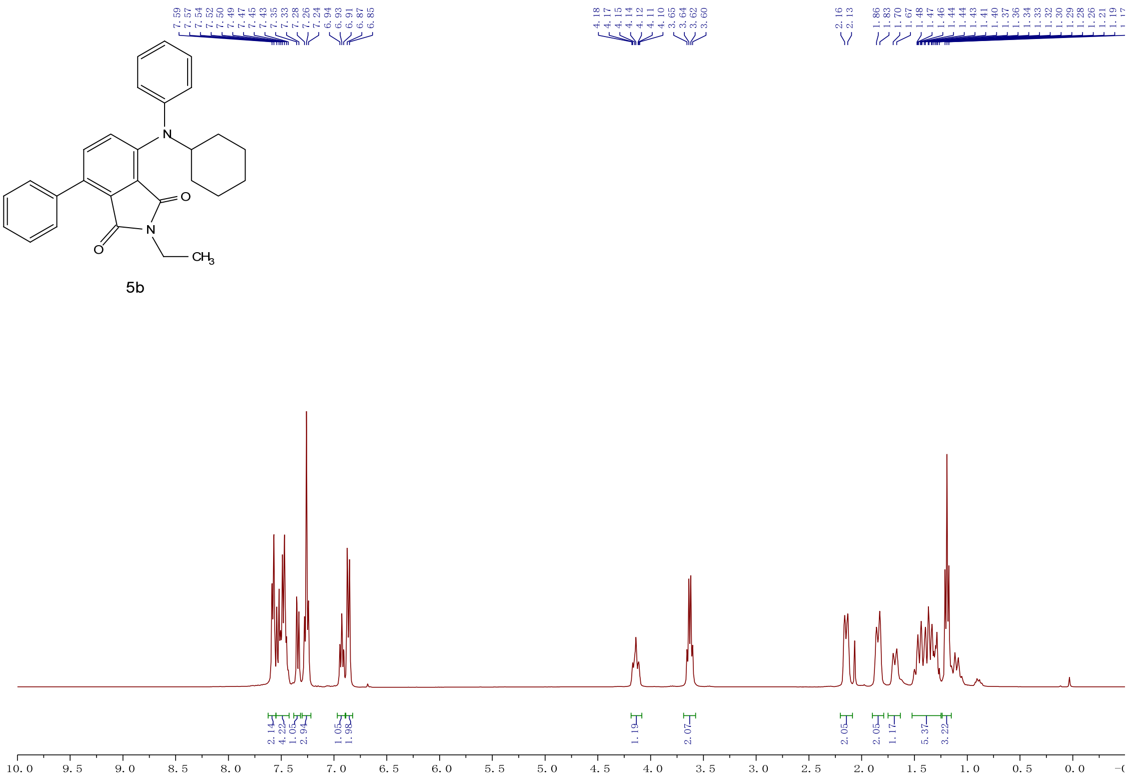

Supplementary Figure 58. ^{1}H NMR spectrum of 5b

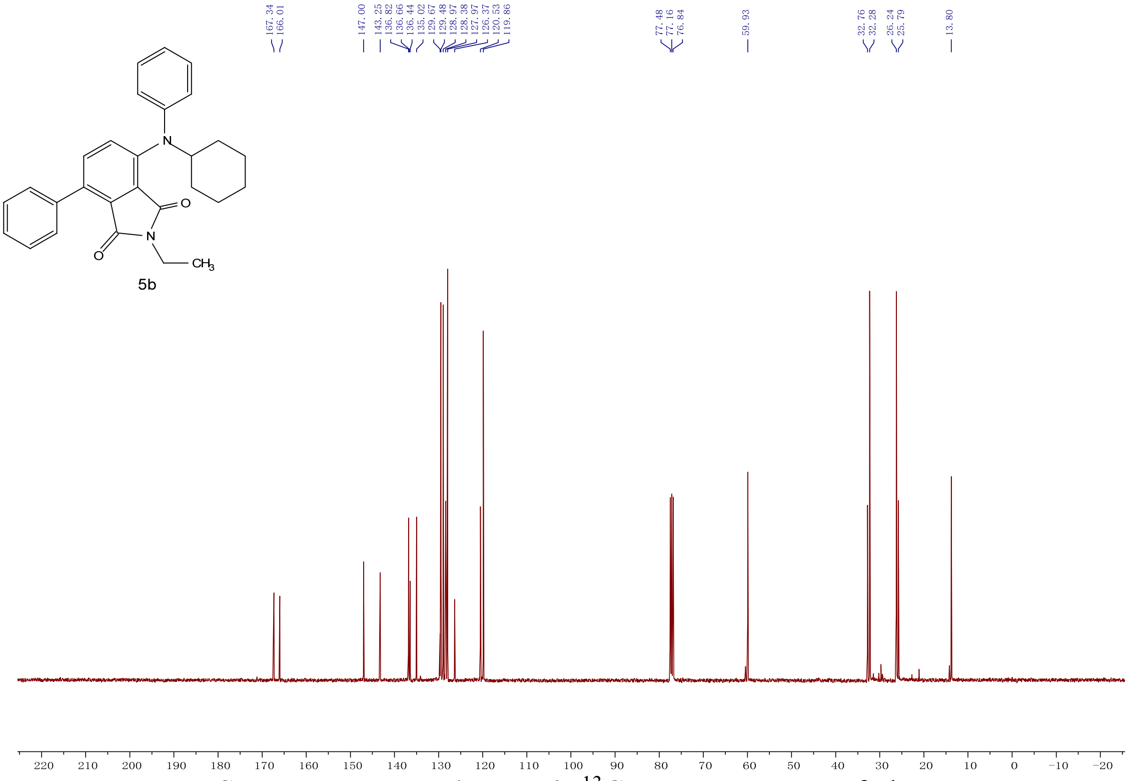

Supplementary Figure 59. ^{13}C NMR spectrum of 5b

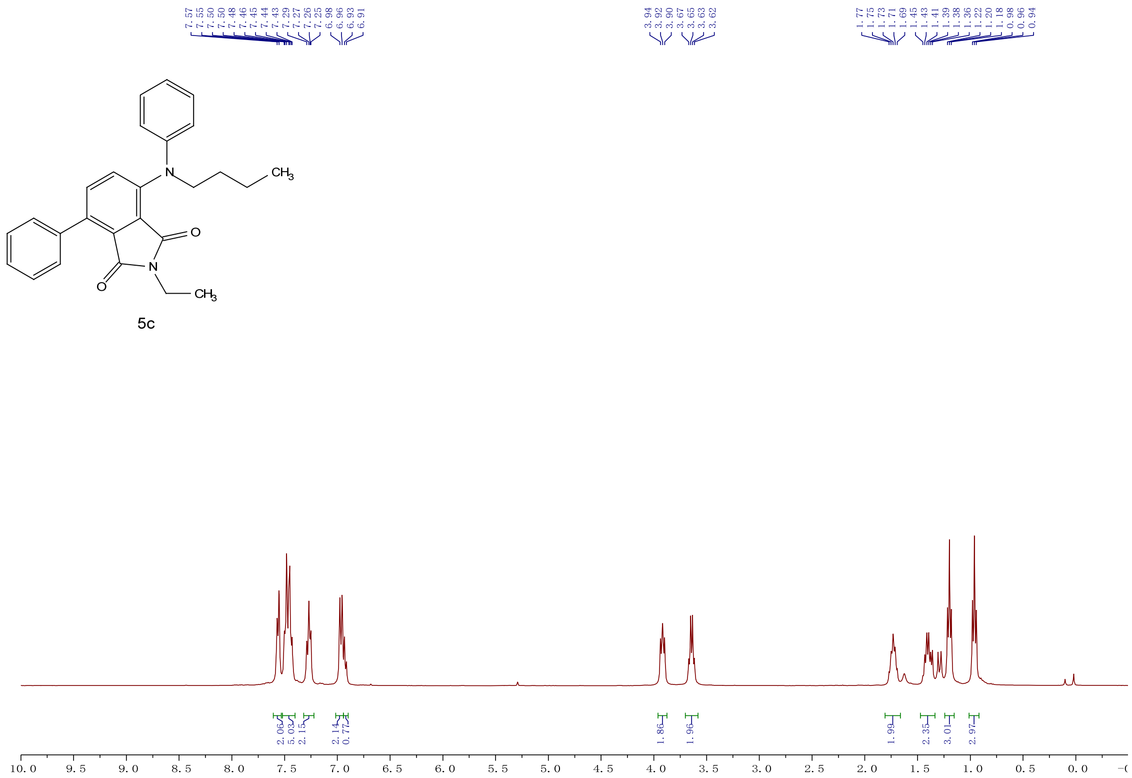

Supplementary Figure 60. ^{1}H NMR spectrum of 5c

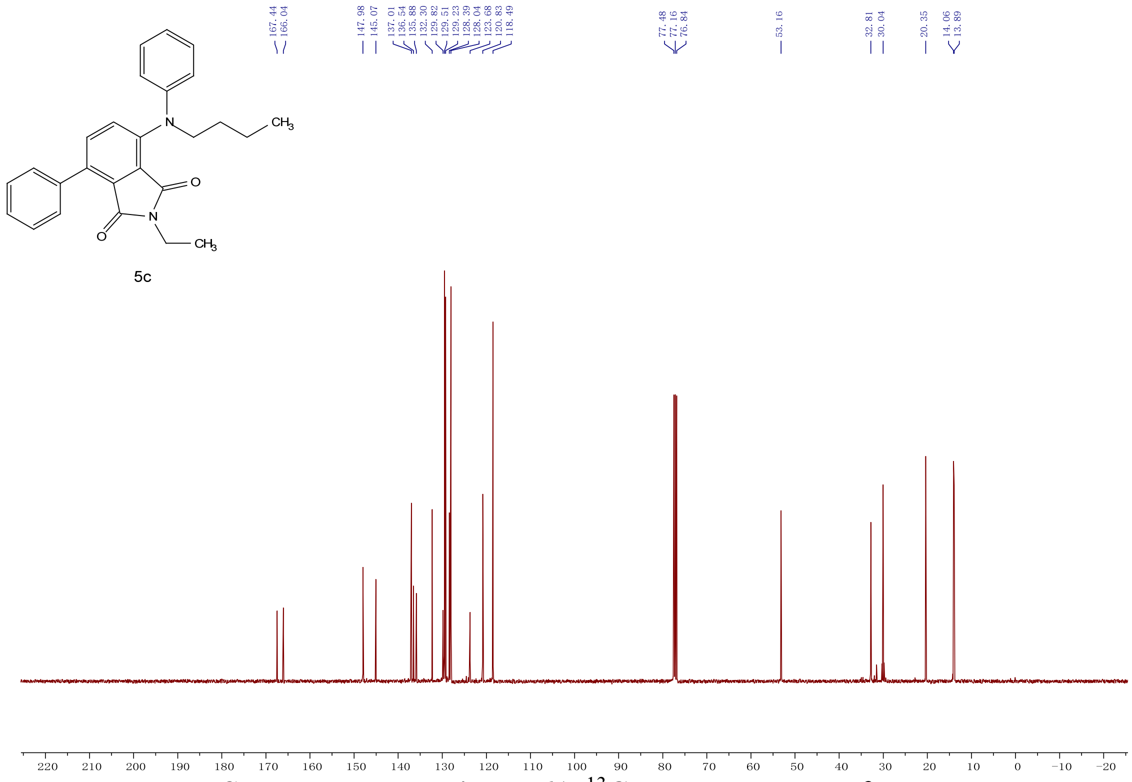

Supplementary Figure 61. ^{13}C NMR spectrum of 5c

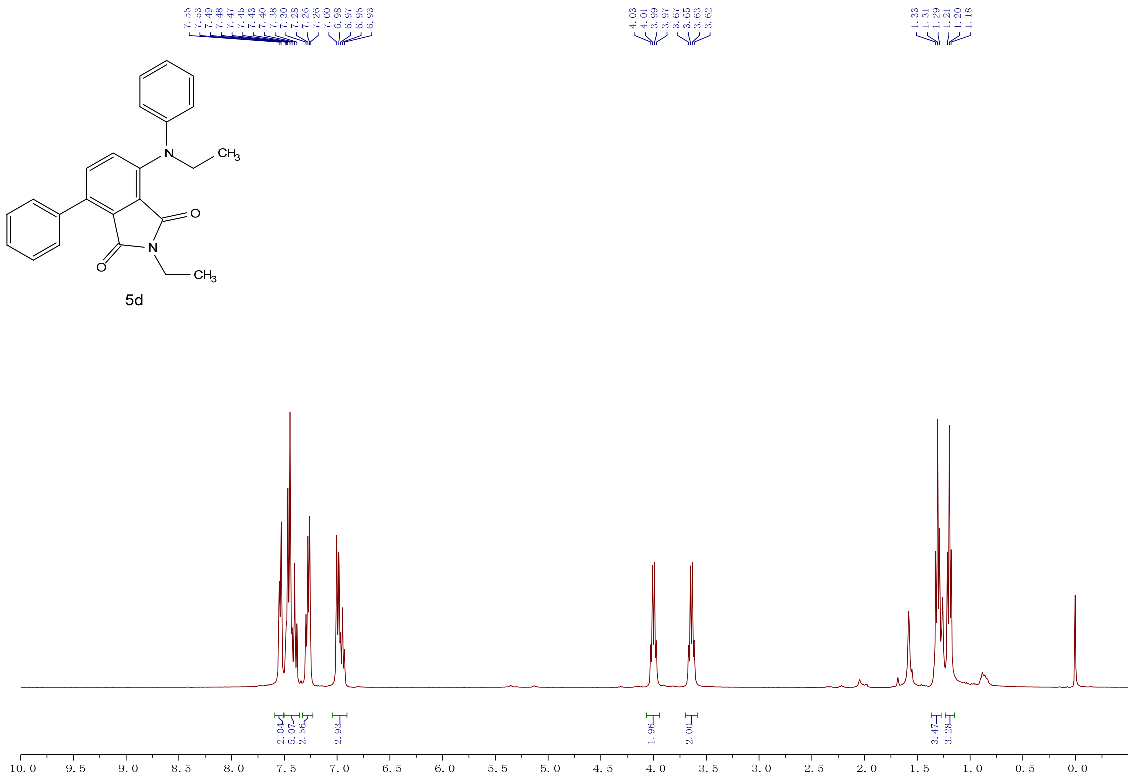

Supplementary Figure 62. ^{1}H NMR spectrum of 5d

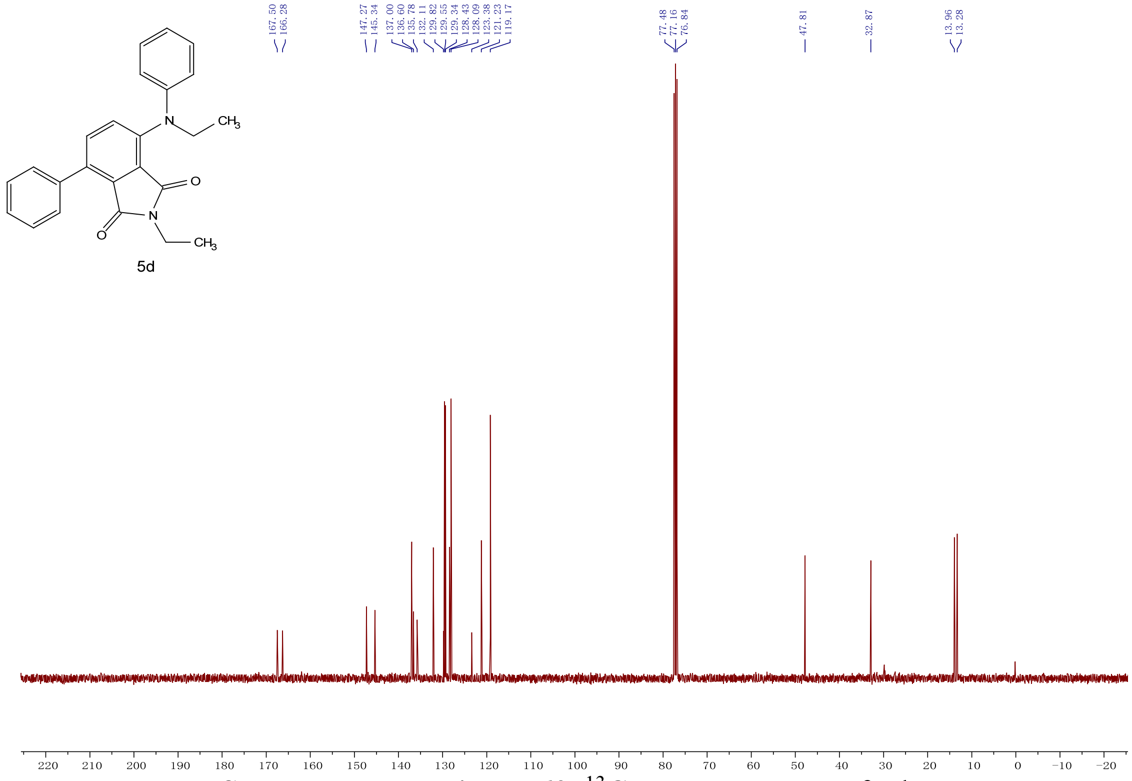

Supplementary Figure 63. ^{13}C NMR spectrum of 5d

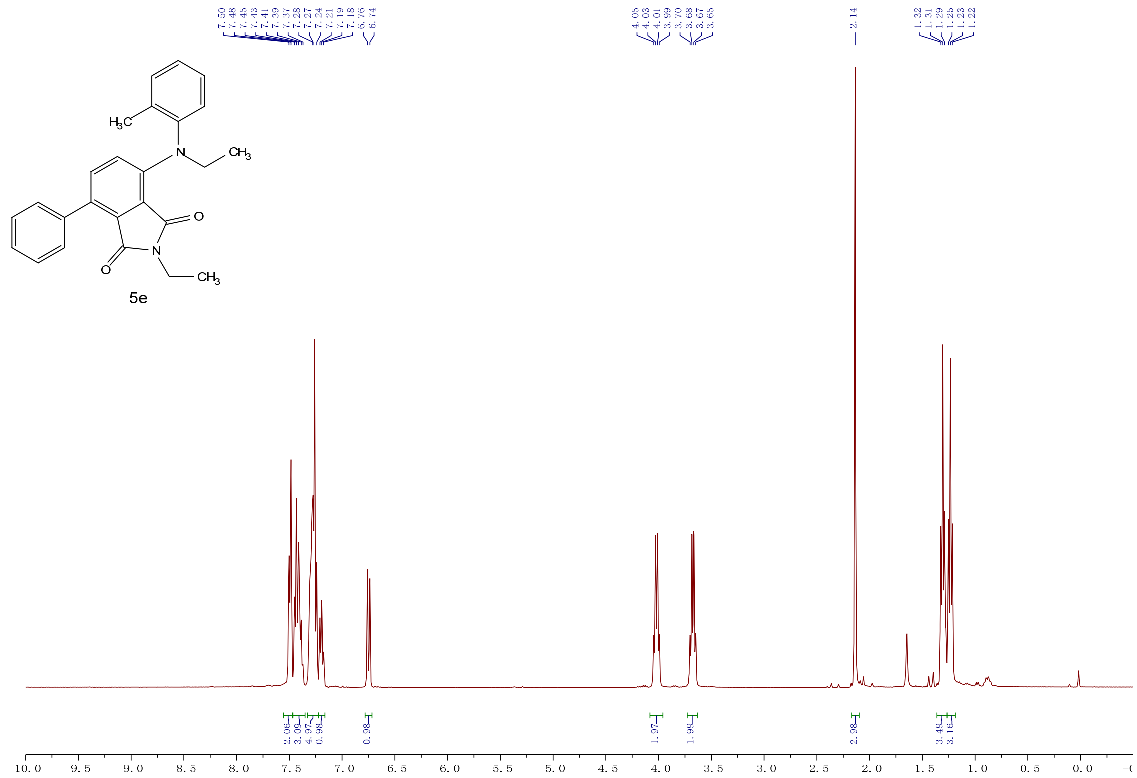

Supplementary Figure 64. ^{1}H NMR spectrum of 5e

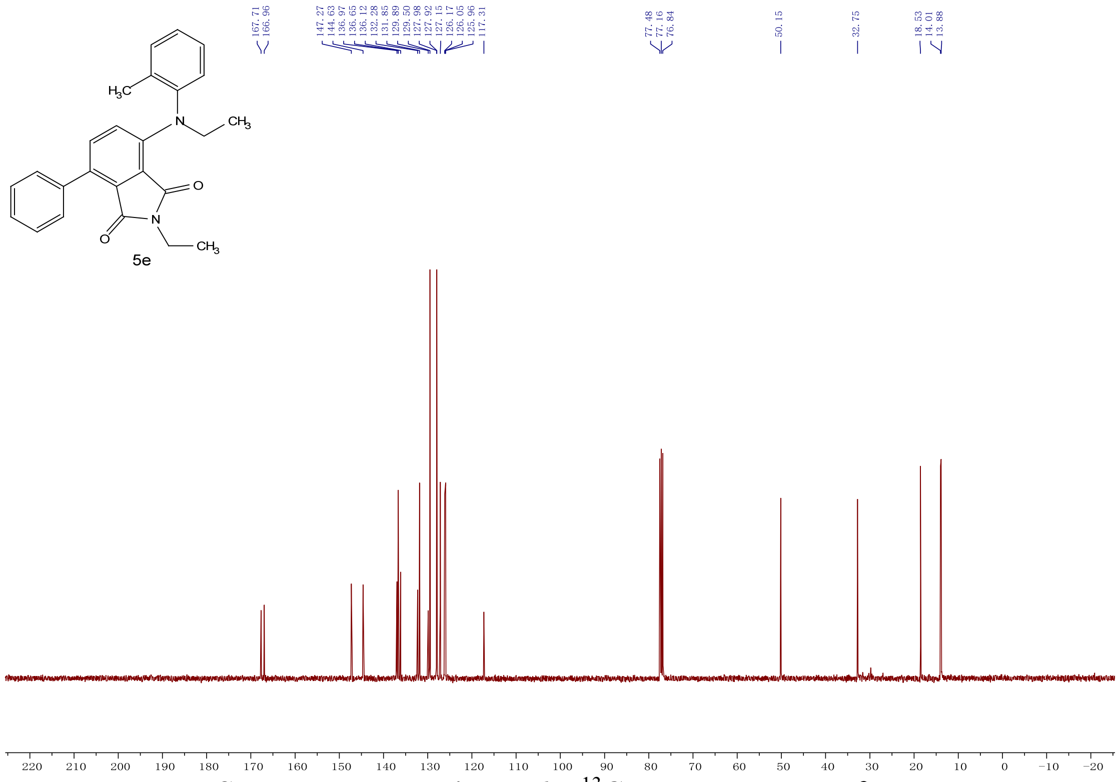

Supplementary Figure 65. ^{13}C NMR spectrum of 5e

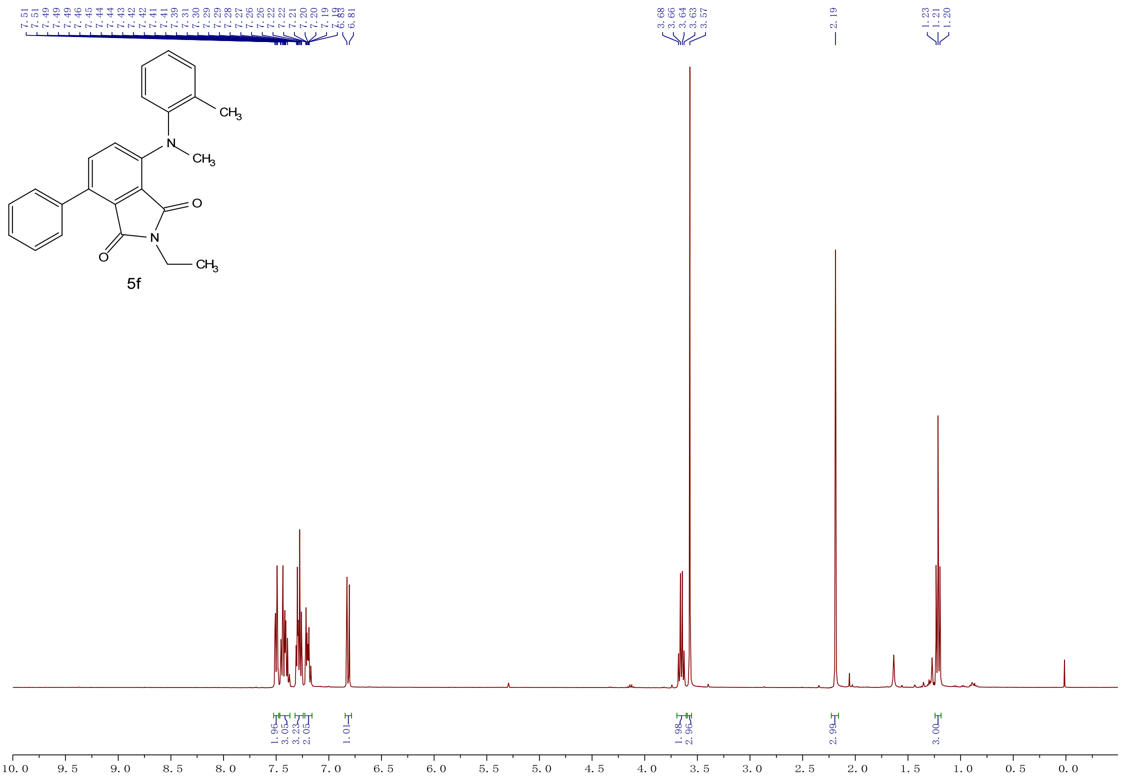

Supplementary Figure 66. ^{1}H NMR spectrum of 5f

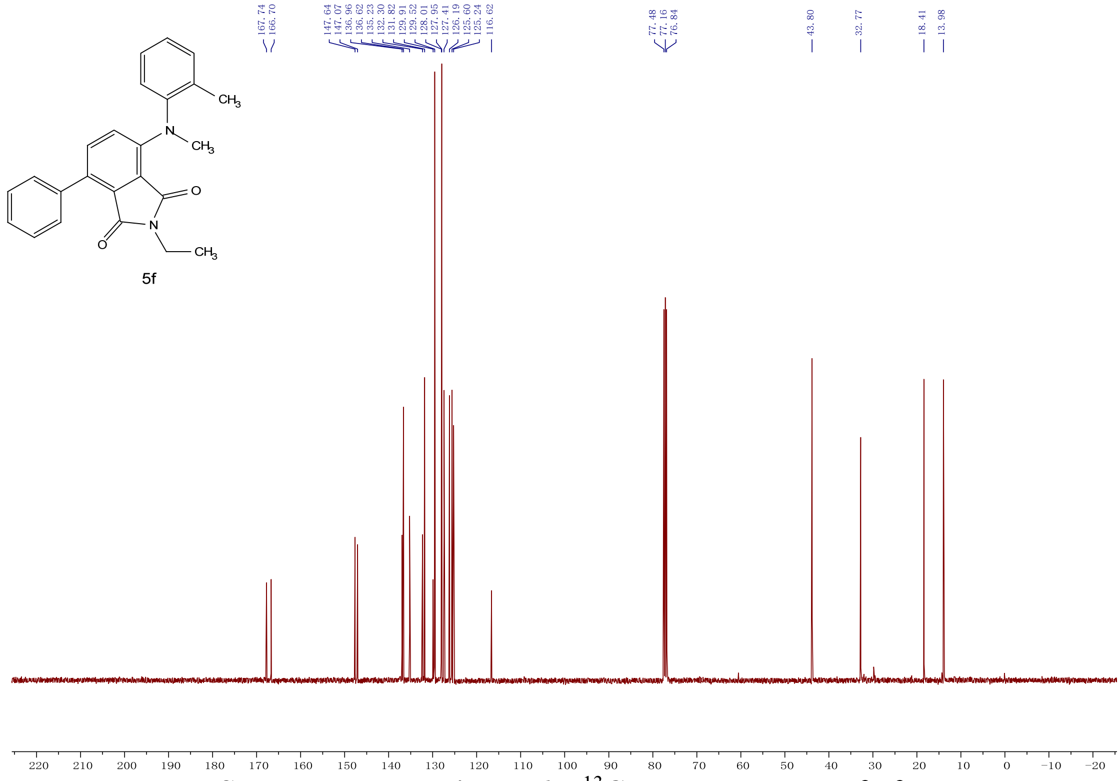

Supplementary Figure 67. ^{13}C NMR spectrum of 5f

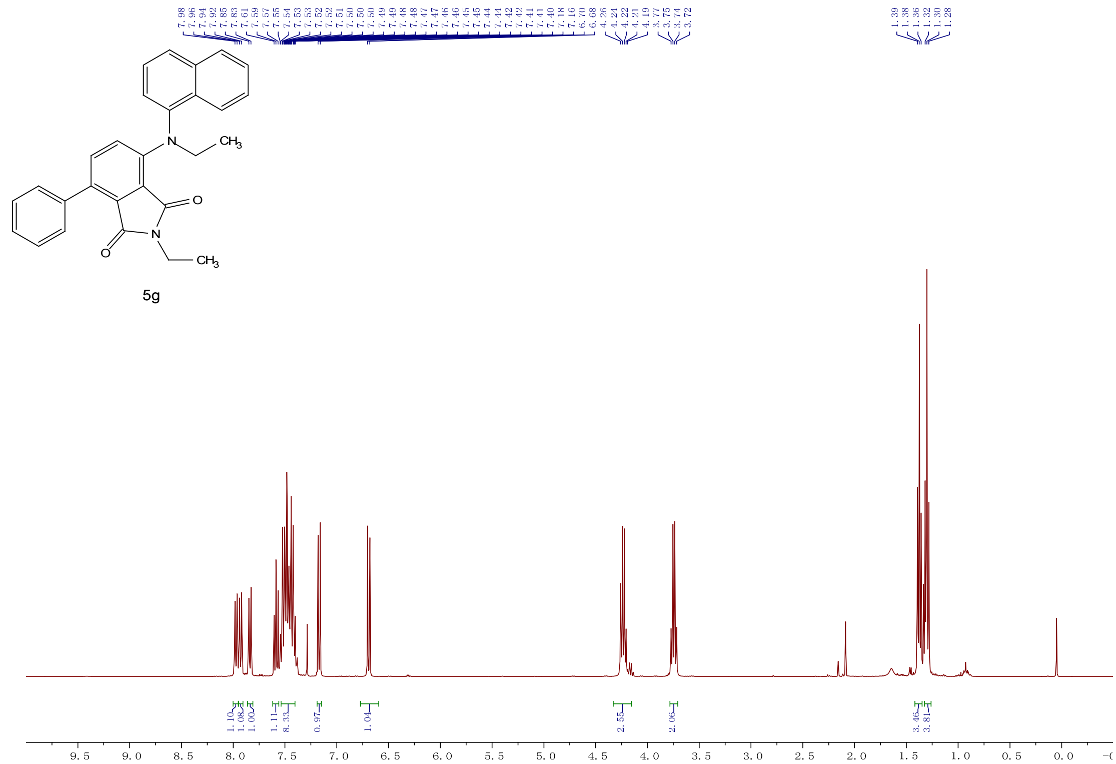

Supplementary Figure 68. ^{1}H NMR spectrum of 5g

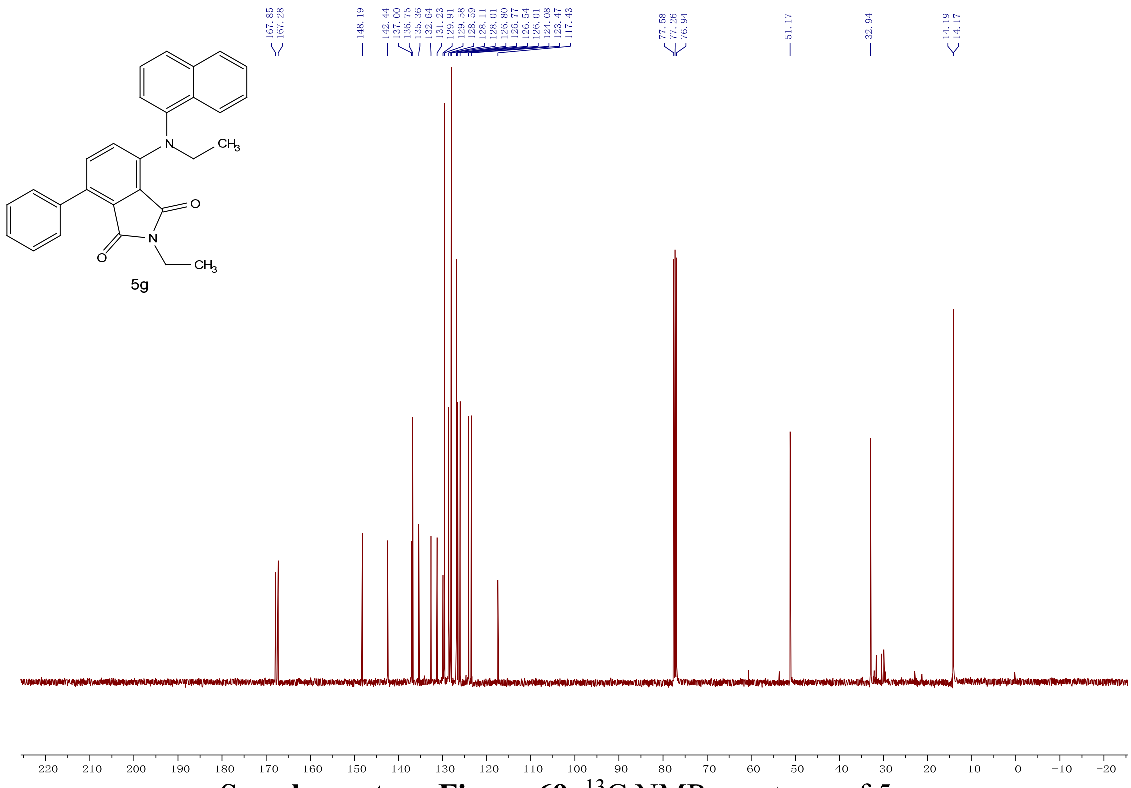

Supplementary Figure 69. ^{13}C NMR spectrum of 5g

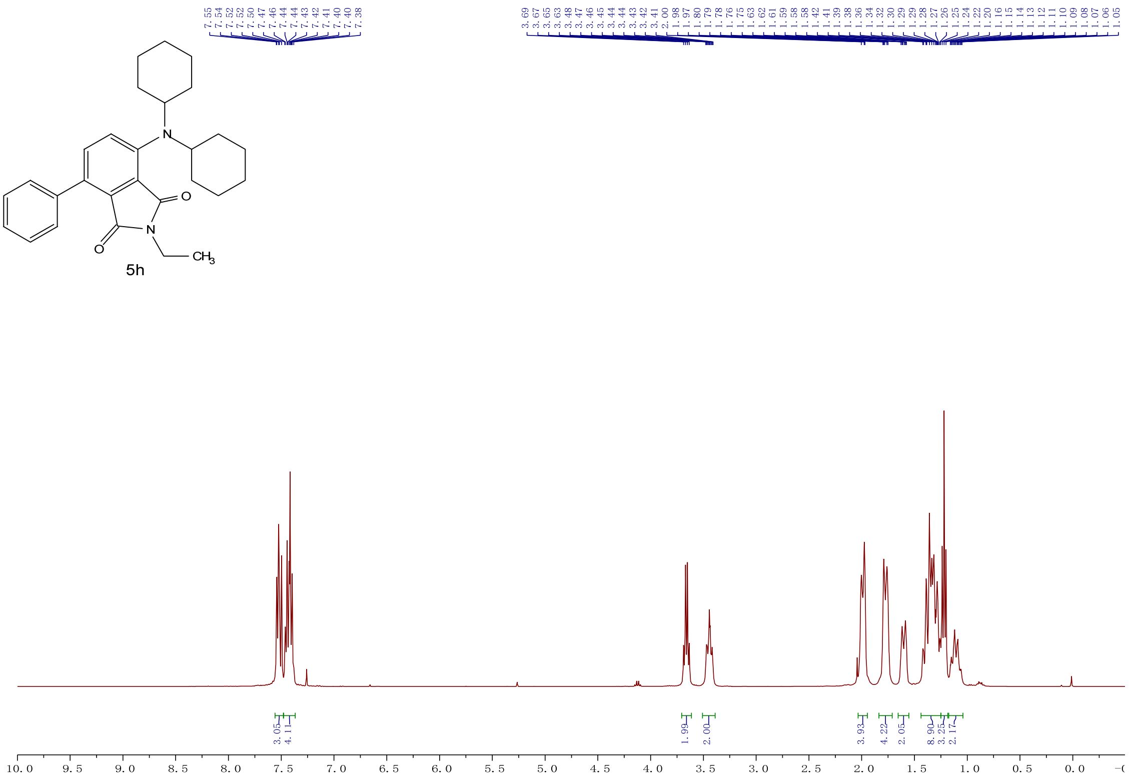

Supplementary Figure 70. ^{1}H NMR spectrum of 5h

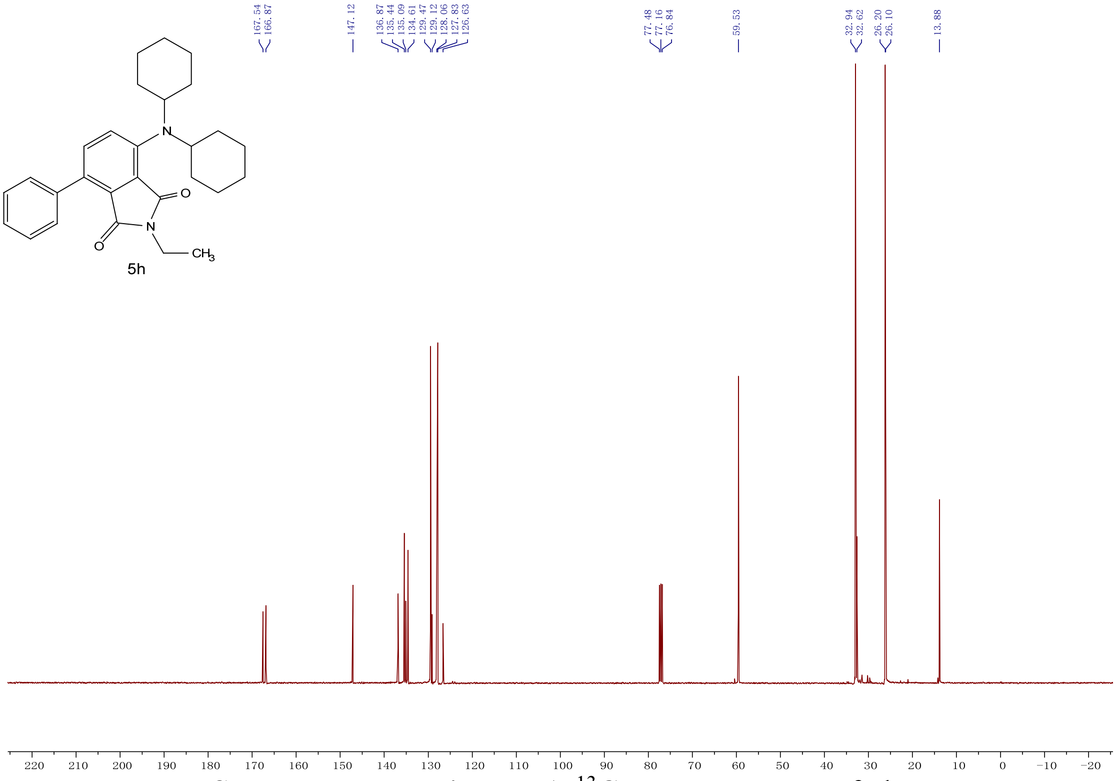

Supplementary Figure 71. ^{13}C NMR spectrum of 5h

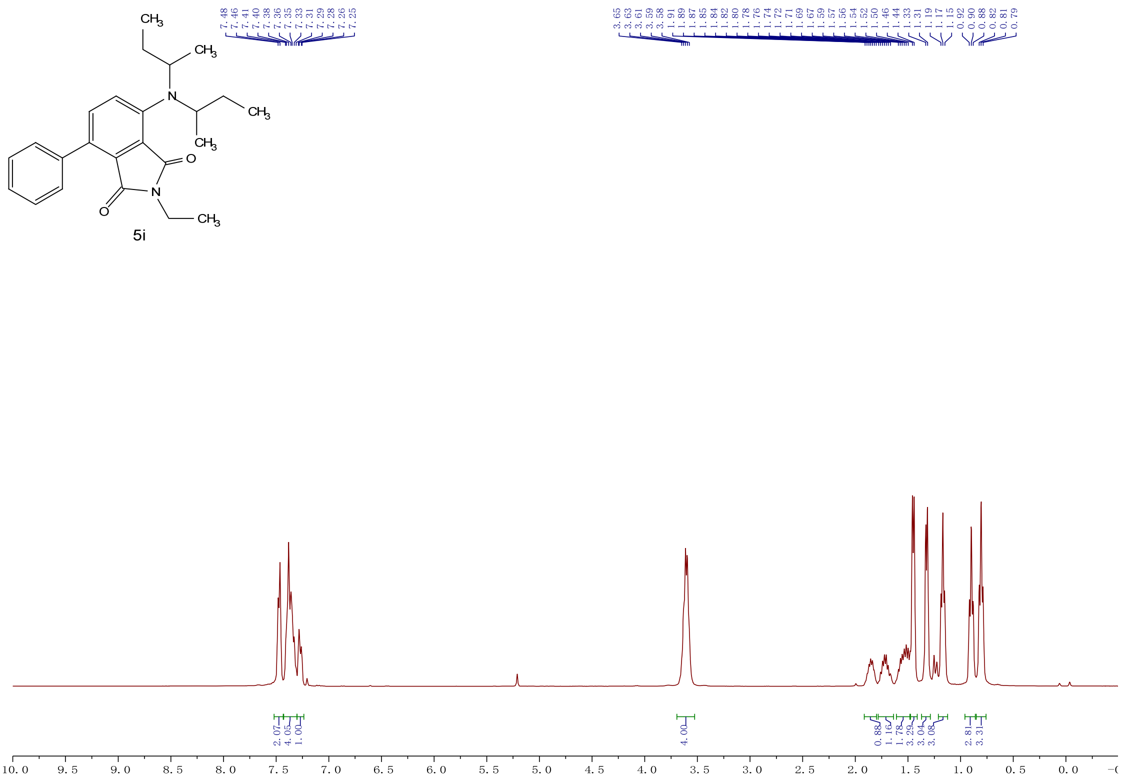

Supplementary Figure 72. ^{1}H NMR spectrum of 5i

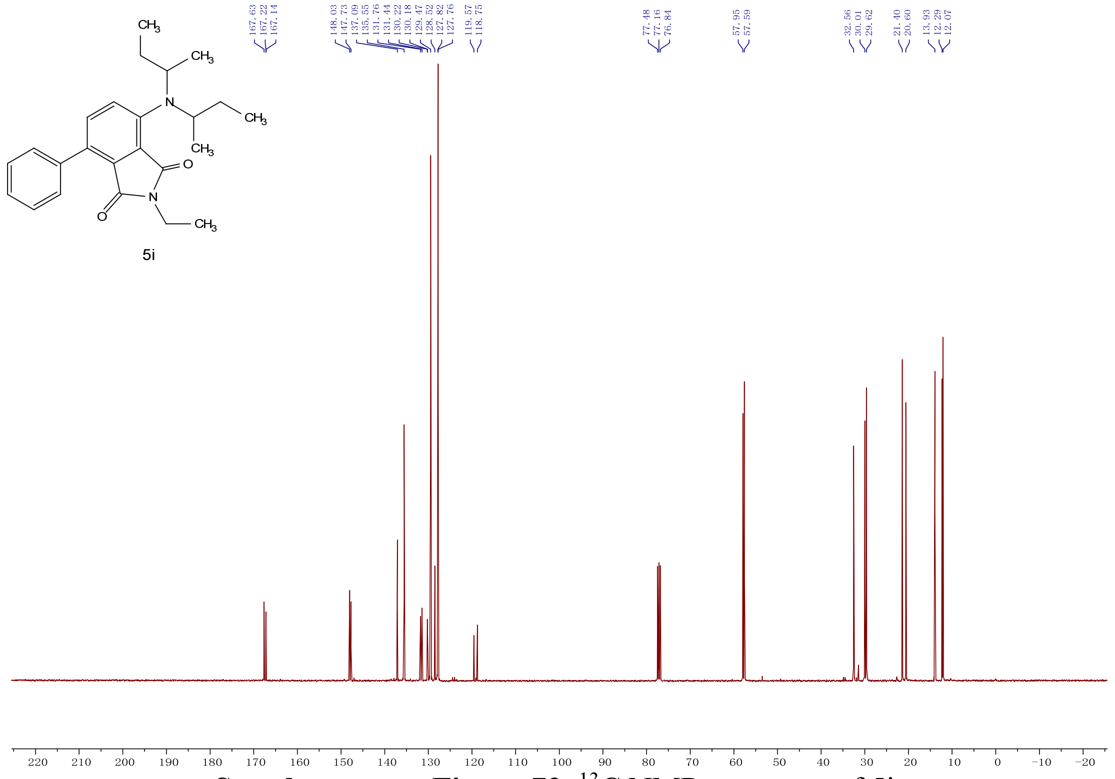

Supplementary Figure 73. ^{13}C NMR spectrum of 5i

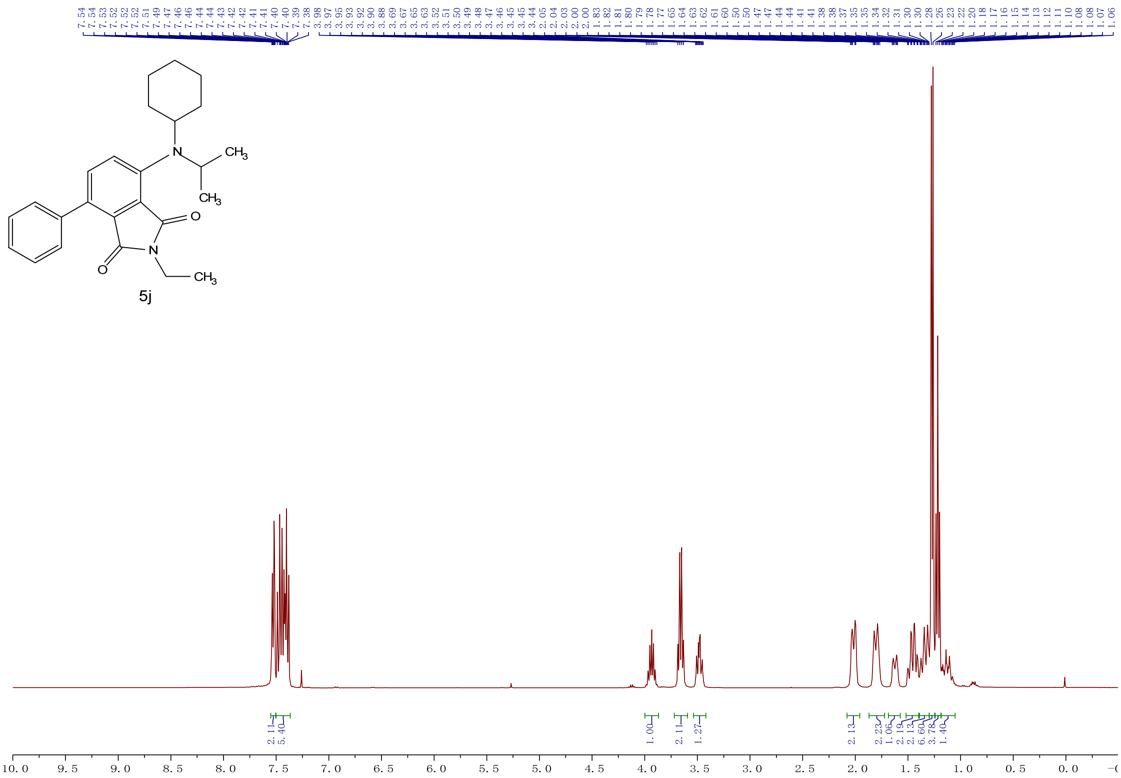

Supplementary Figure 74. ^{1}H NMR spectrum of 5j

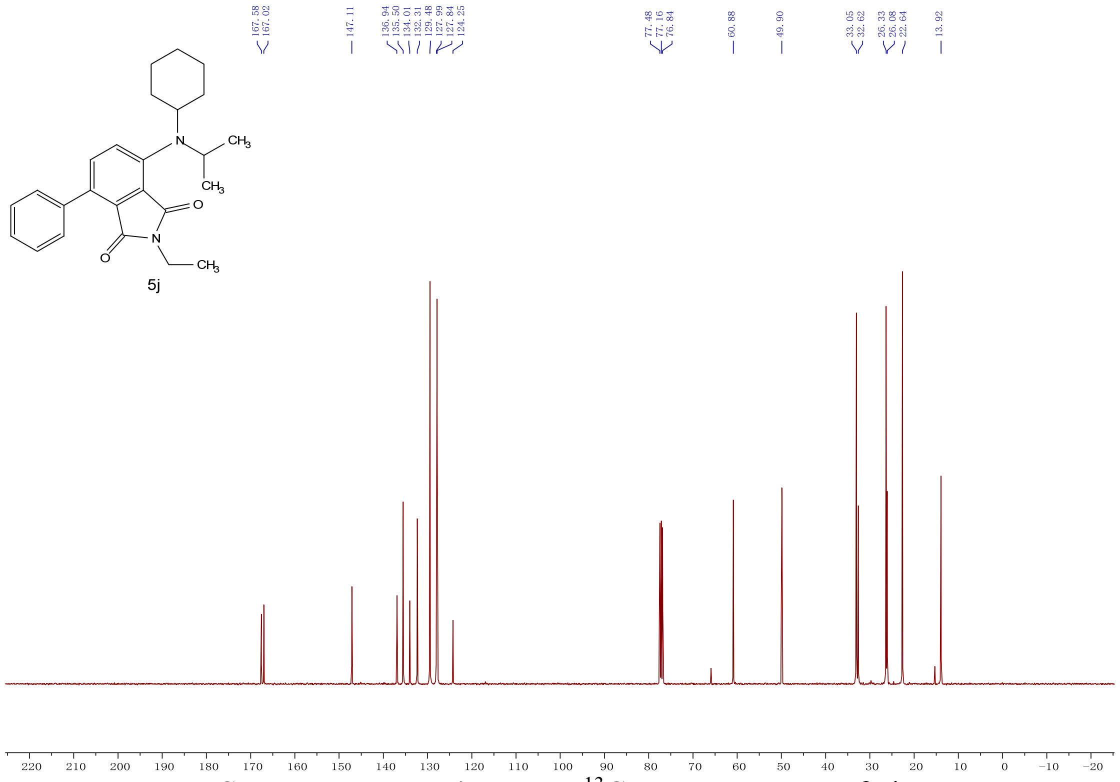

Supplementary Figure 75. ^{13}C NMR spectrum of 5j

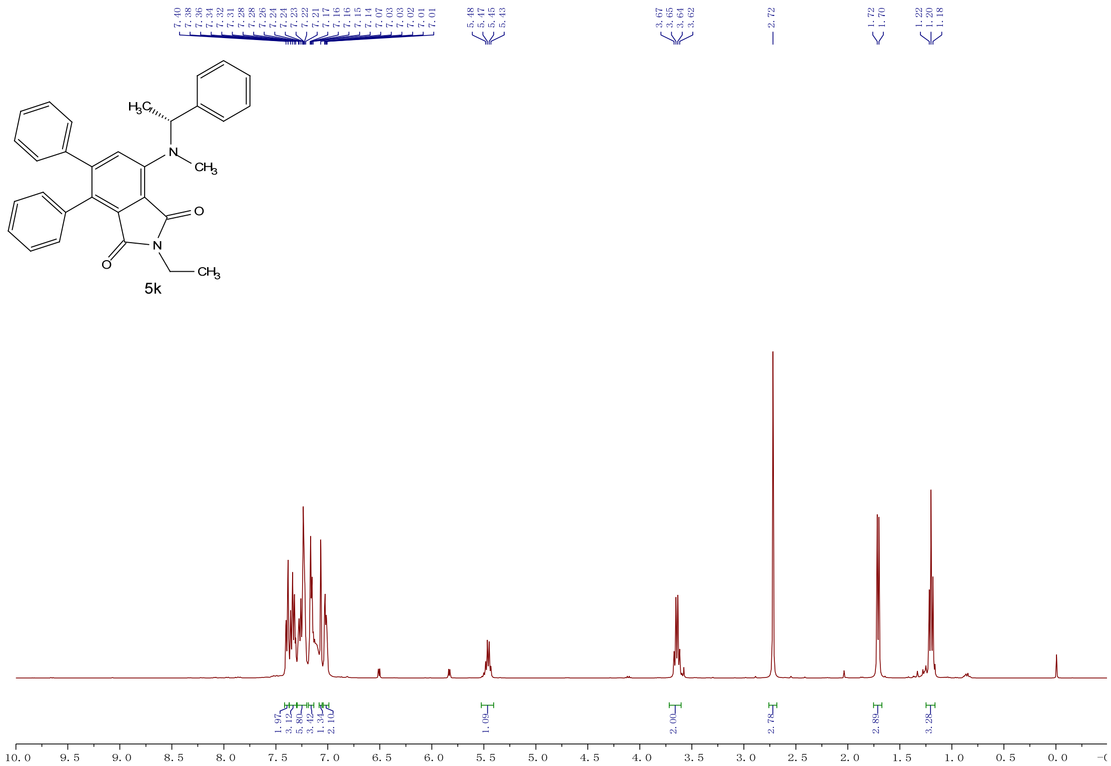

Supplementary Figure 76. ^{1}H NMR spectrum of 5k

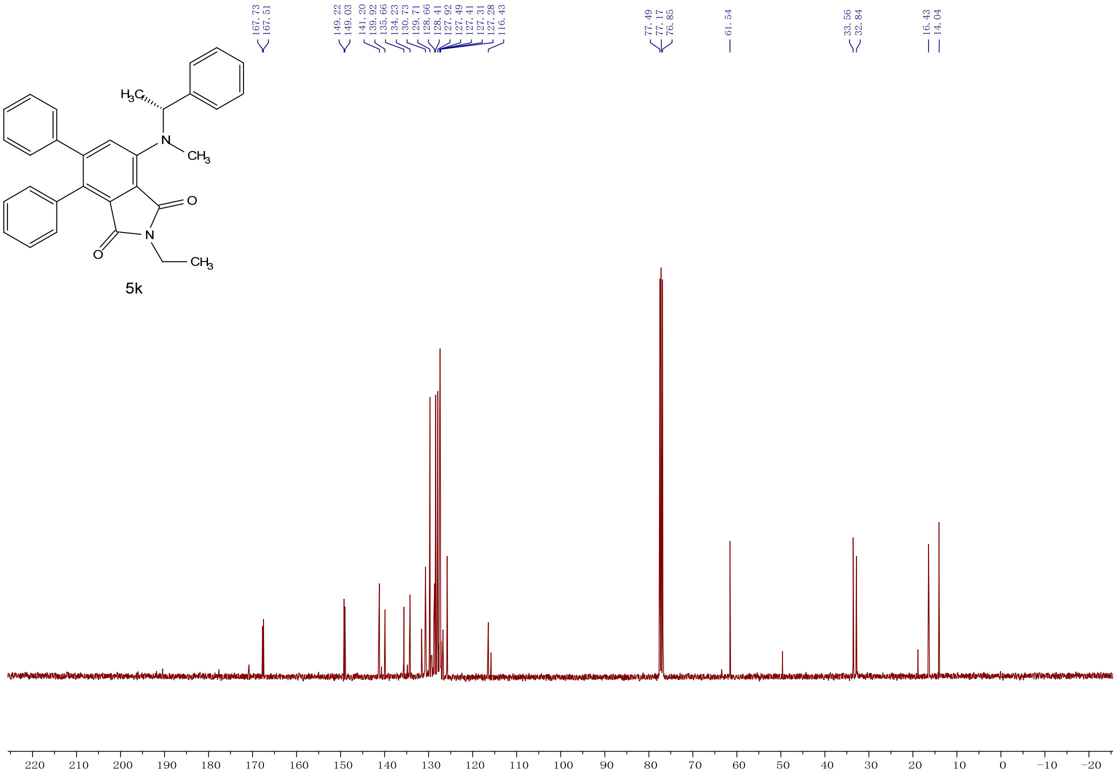

Supplementary Figure 77. ^{13}C NMR spectrum of 5k

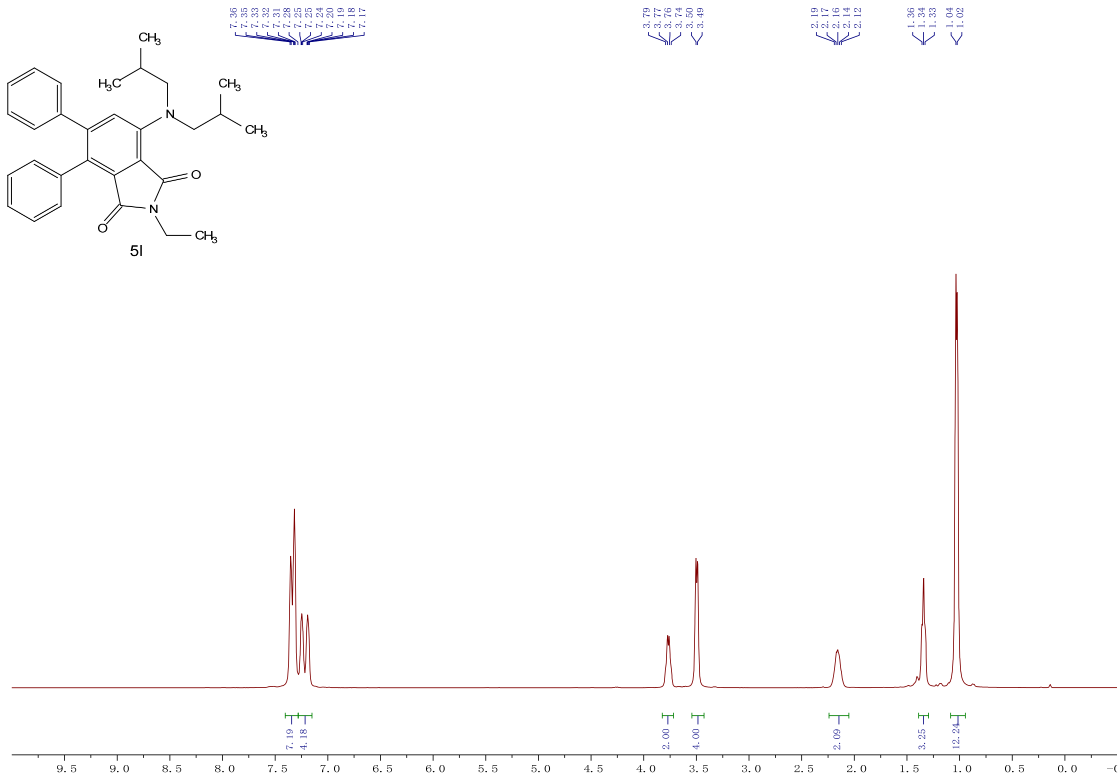

Supplementary Figure 78. ^{1}H NMR spectrum of 5l

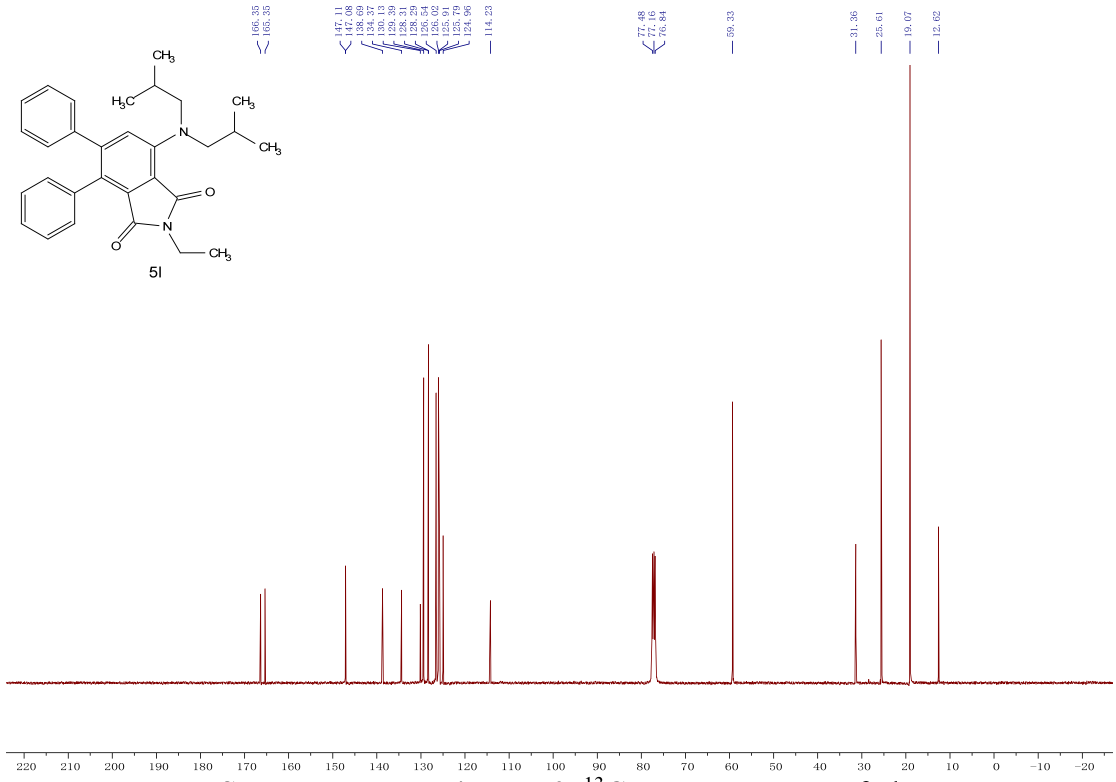

Supplementary Figure 79. ^{13}C NMR spectrum of 5l

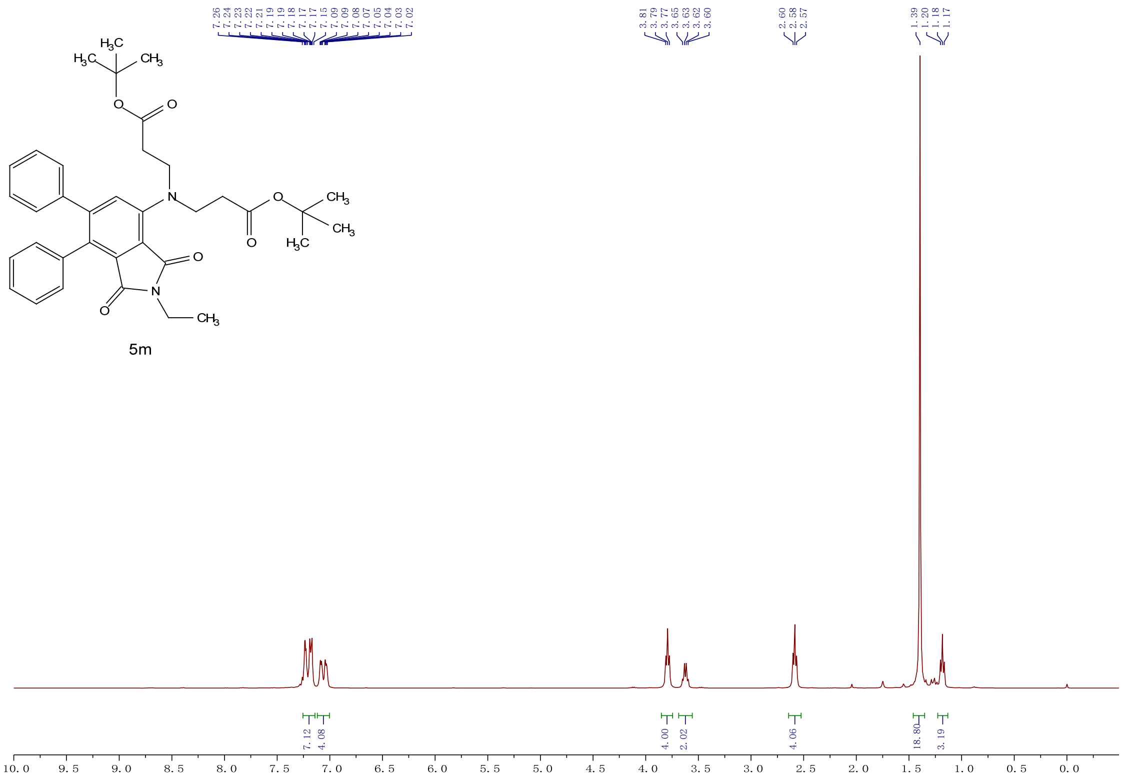

Supplementary Figure 80. ^{1}H NMR spectrum of 5m

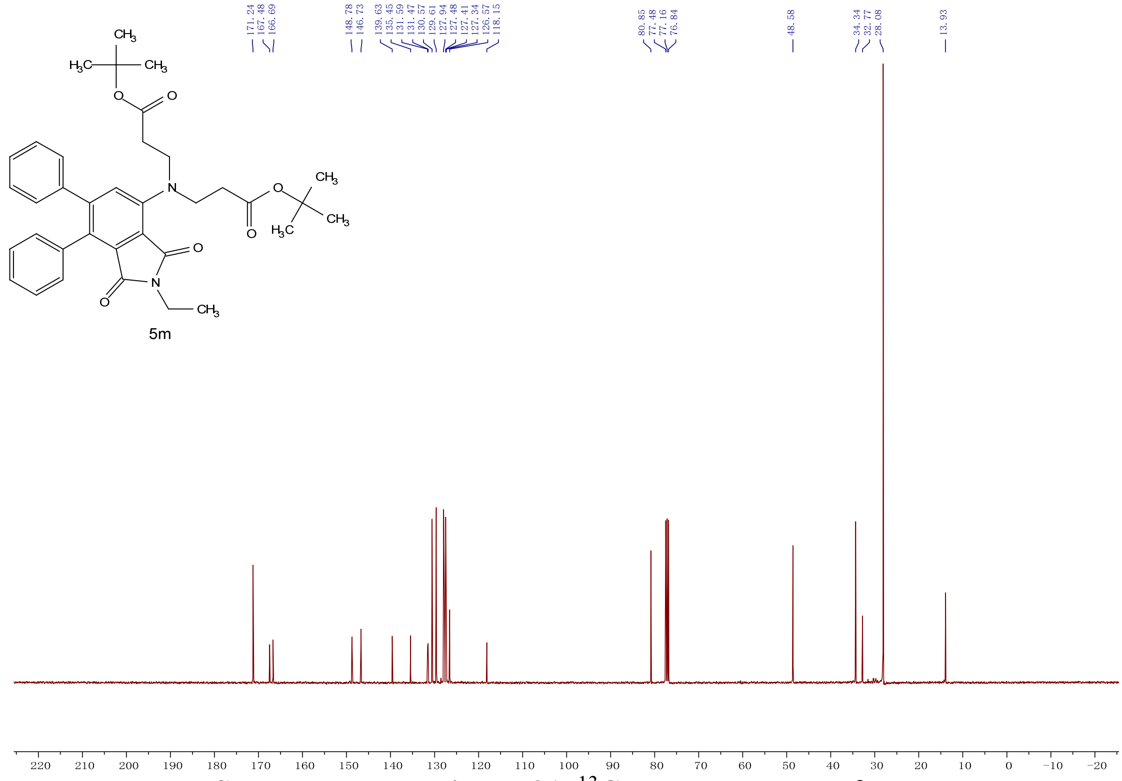

Supplementary Figure 81. ^{13}C NMR spectrum of 5m

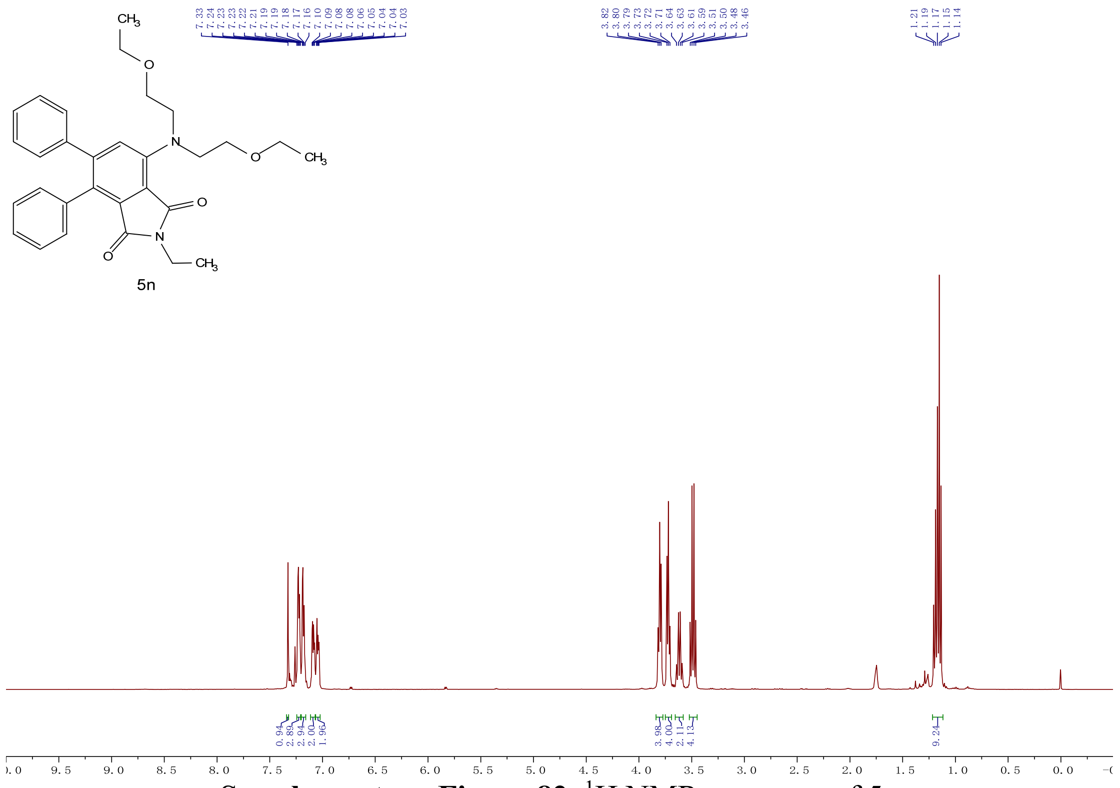

Supplementary Figure 82. ^{1}H NMR spectrum of 5n

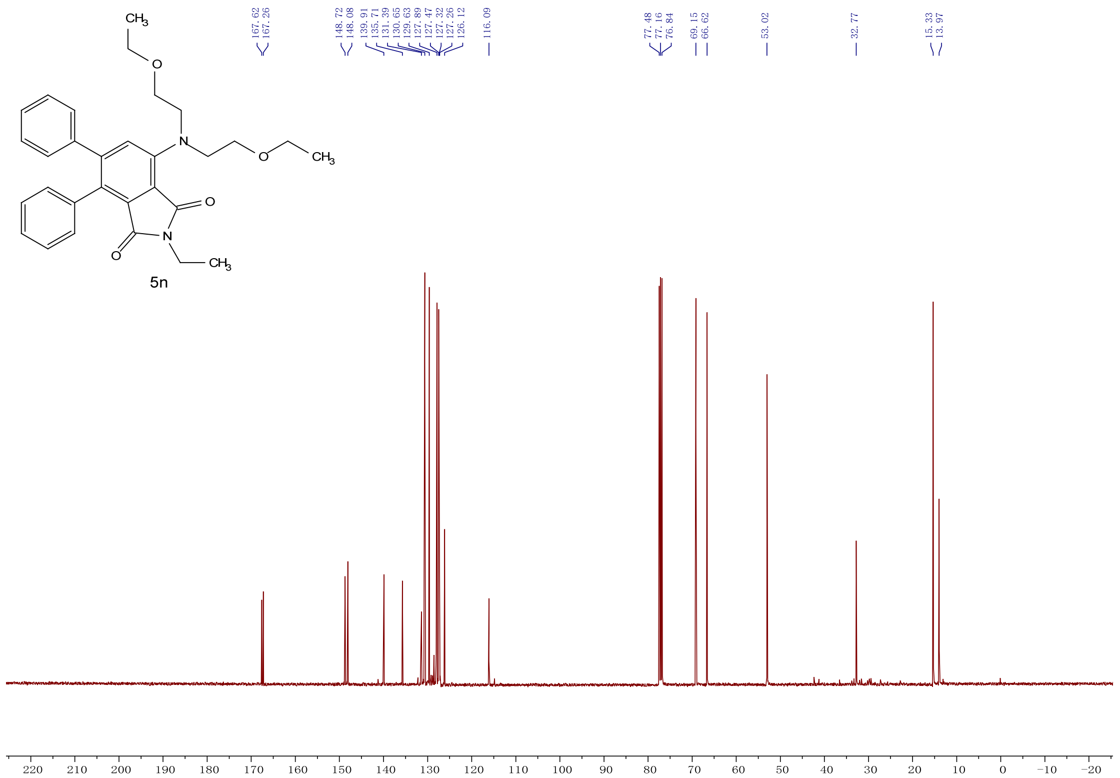

Supplementary Figure 83. ^{13}C NMR spectrum of 5n

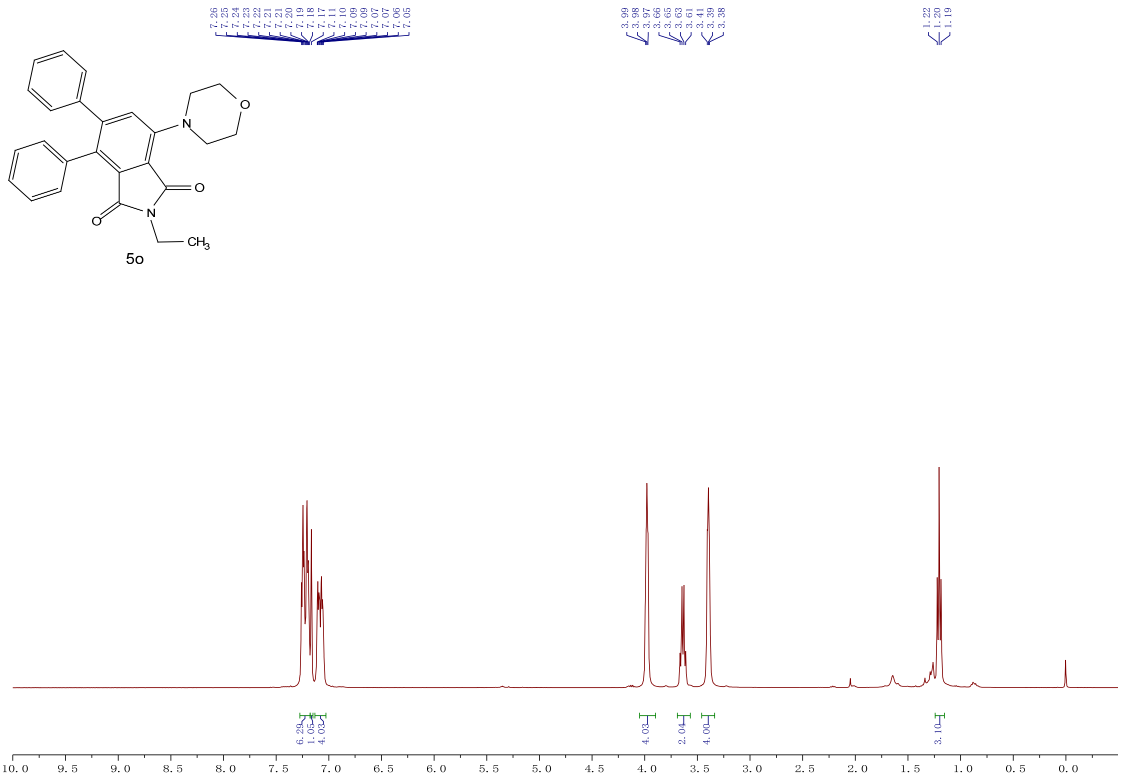

Supplementary Figure 84. ^{1}H NMR spectrum of 5o

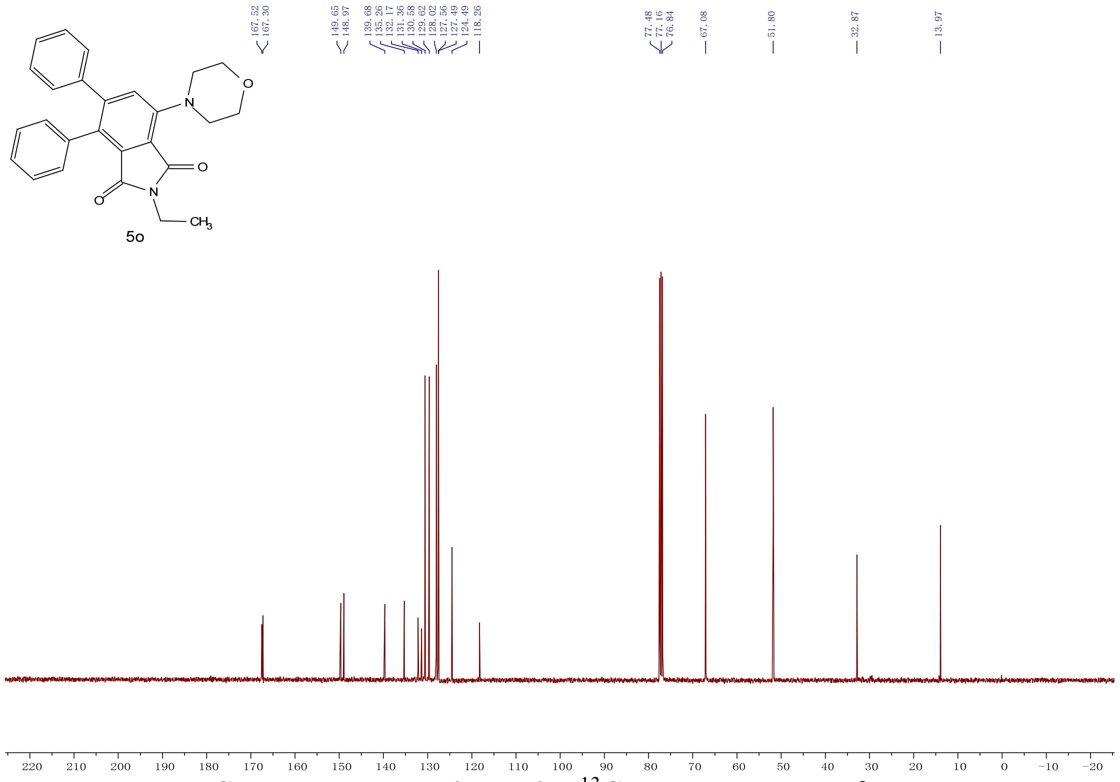

Supplementary Figure 85. ^{13}C NMR spectrum of 5o

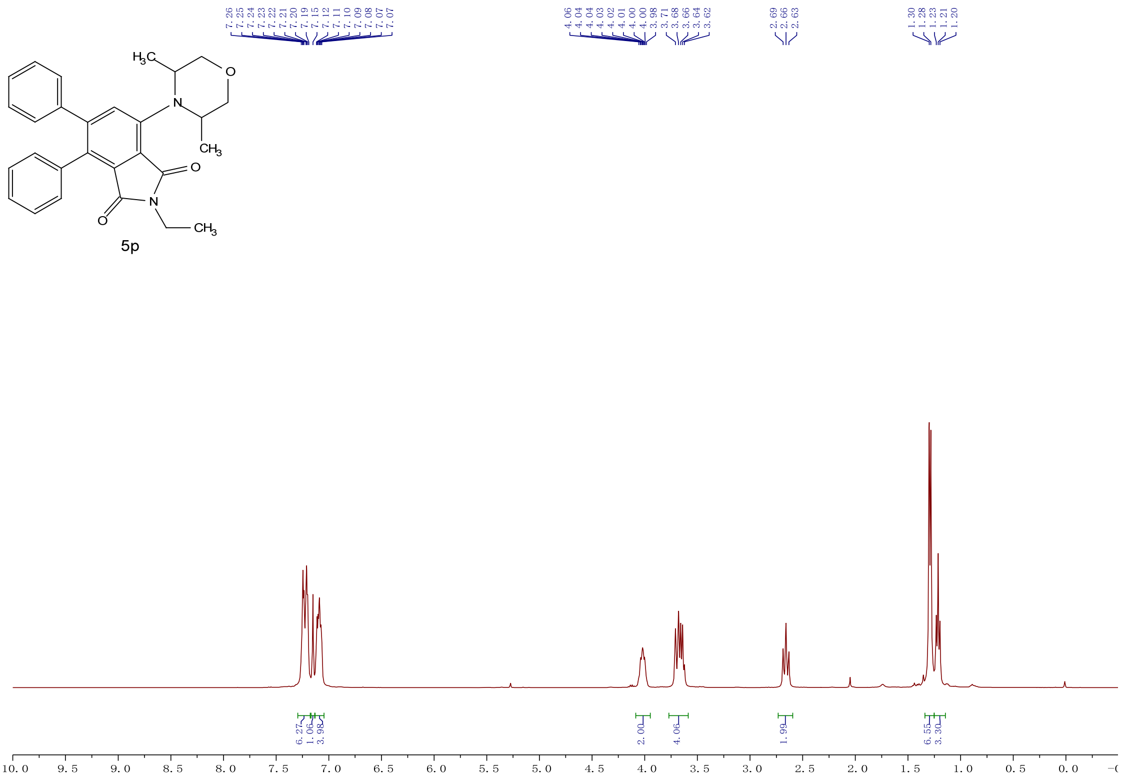

Supplementary Figure 86. ^{1}H NMR spectrum of 5p

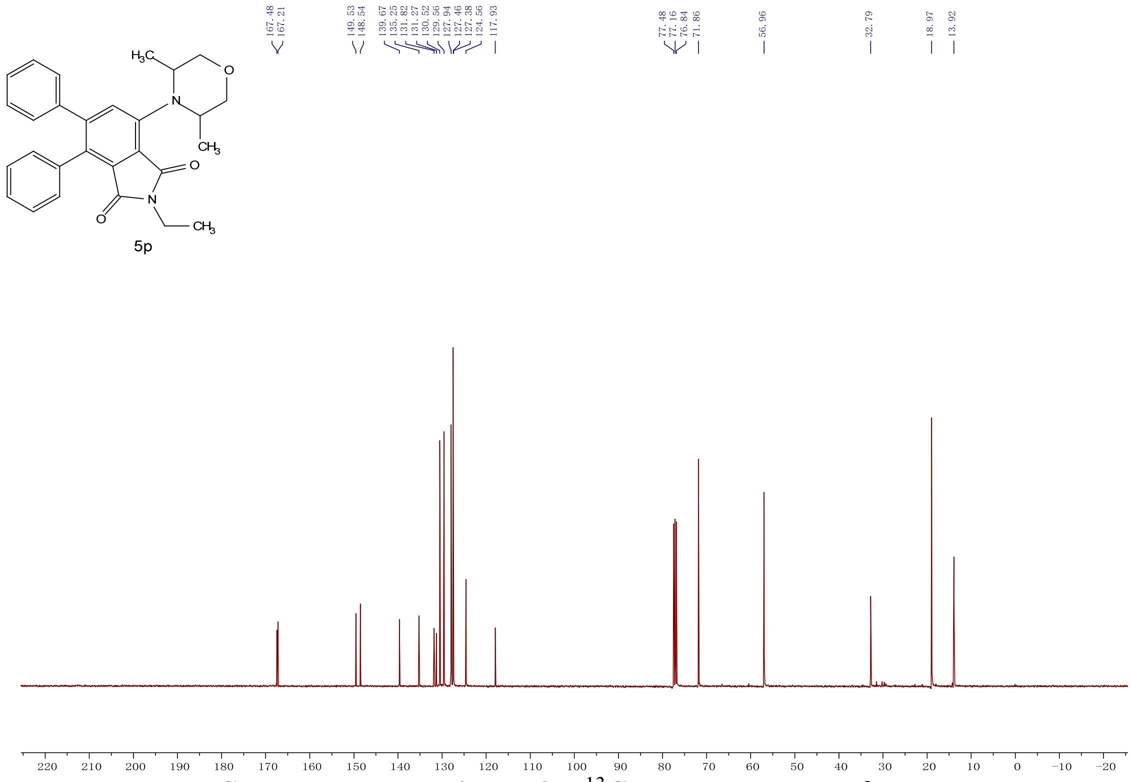

Supplementary Figure 87. ^{13}C NMR spectrum of 5p

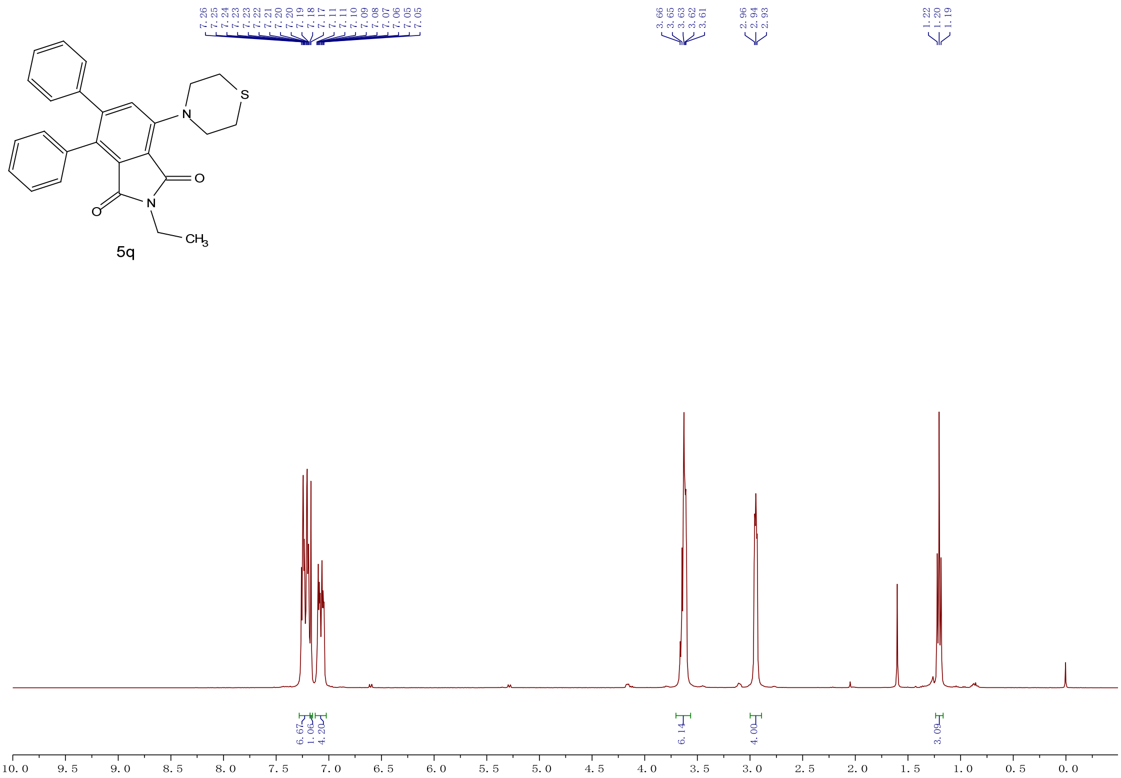

Supplementary Figure 88. ^{1}H NMR spectrum of 5q

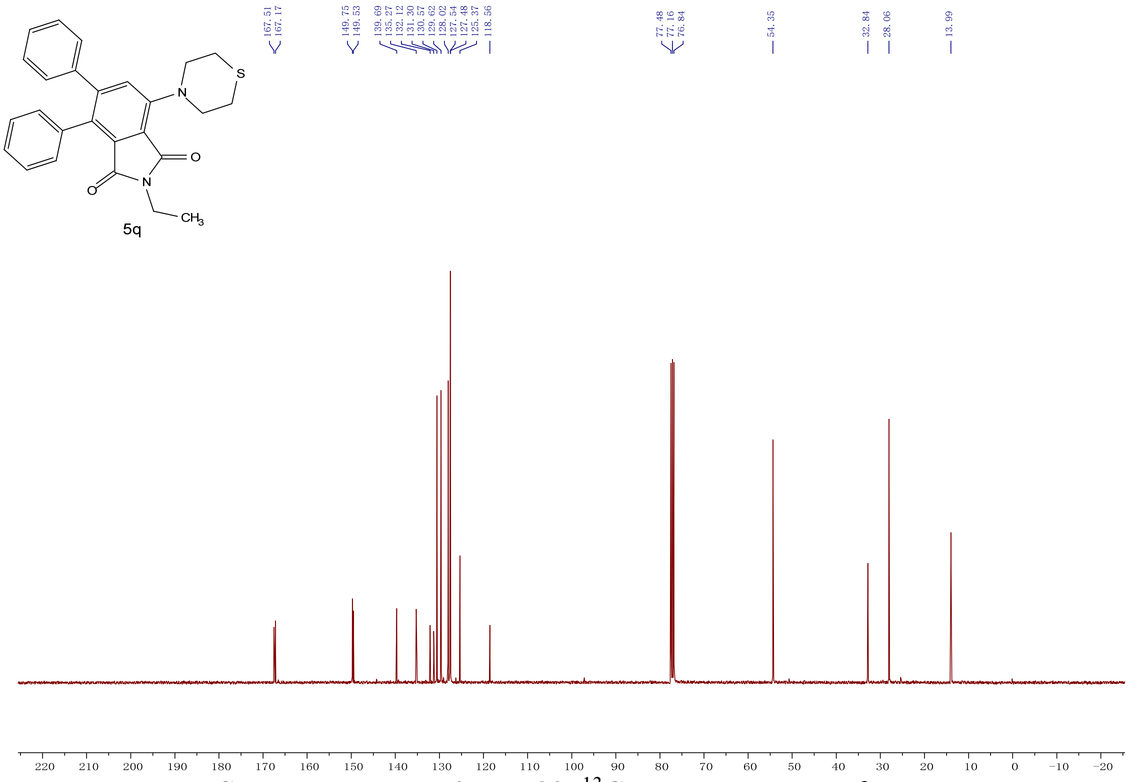

Supplementary Figure 89. ^{13}C NMR spectrum of 5q

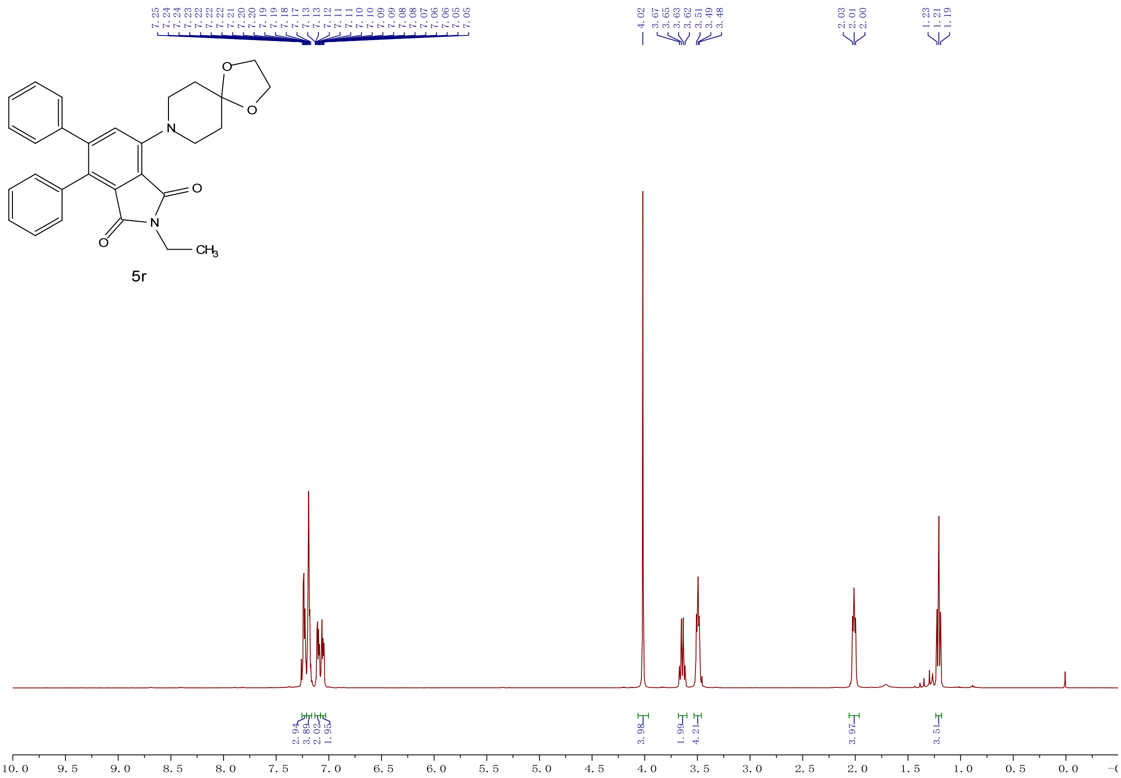

Supplementary Figure 90. ^{1}H NMR spectrum of 5r

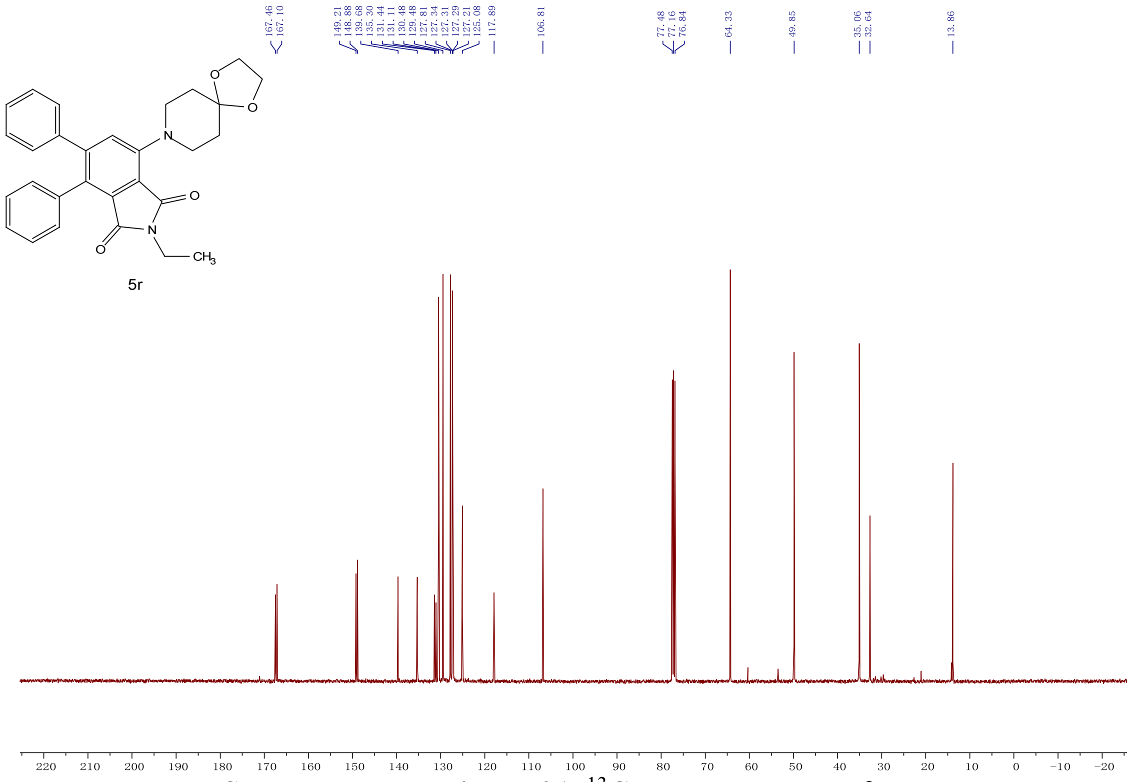

Supplementary Figure 91. ^{13}C NMR spectrum of 5r

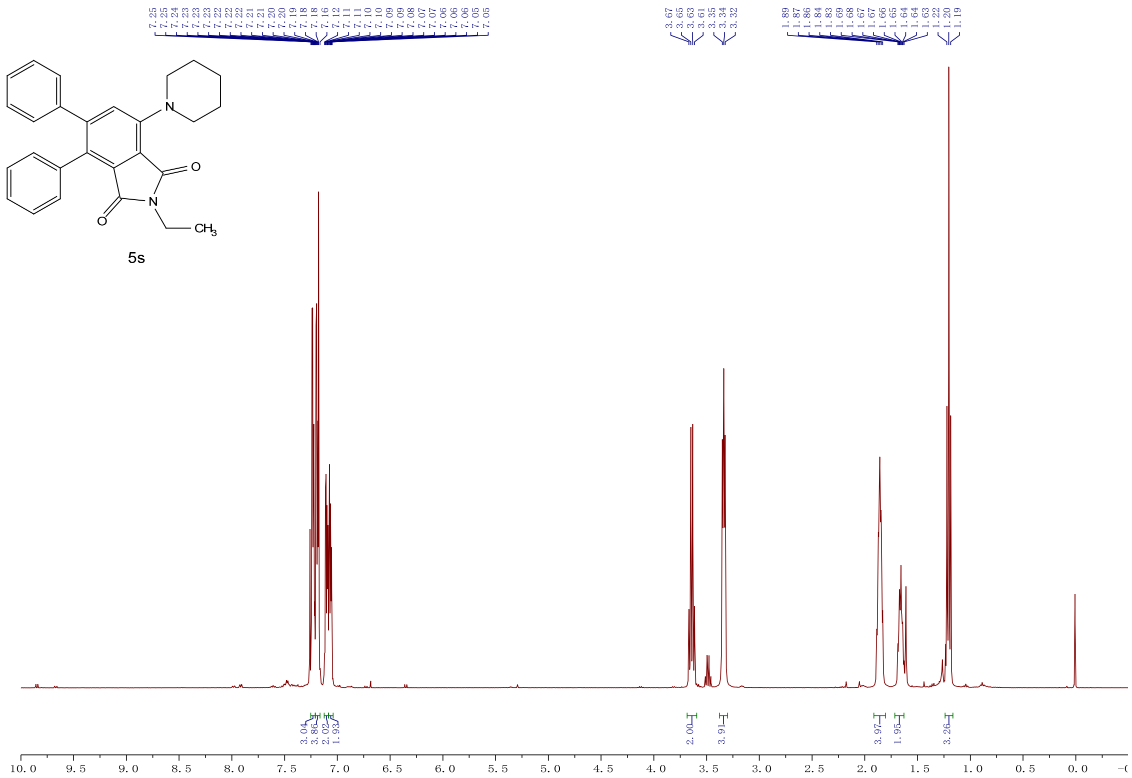

Supplementary Figure 92. ^{1}H NMR spectrum of 5s

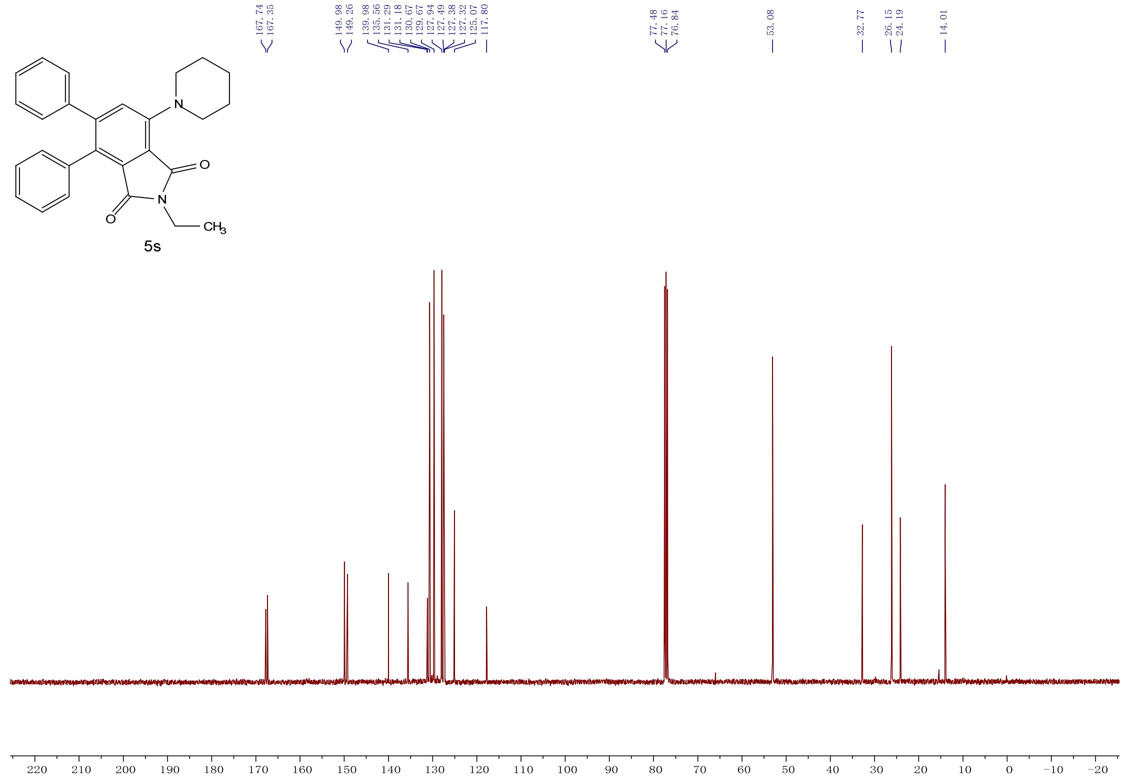

Supplementary Figure 93. ^{13}C NMR spectrum of 5s

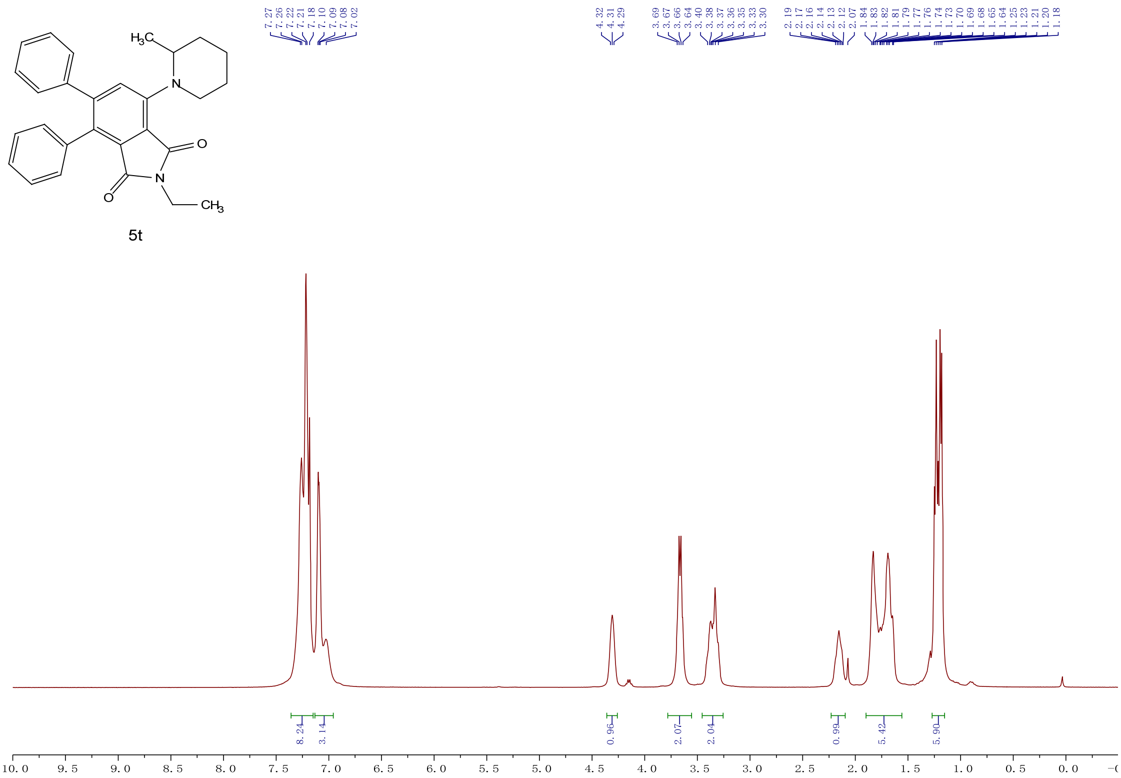

Supplementary Figure 94. ^{1}H NMR spectrum of 5t

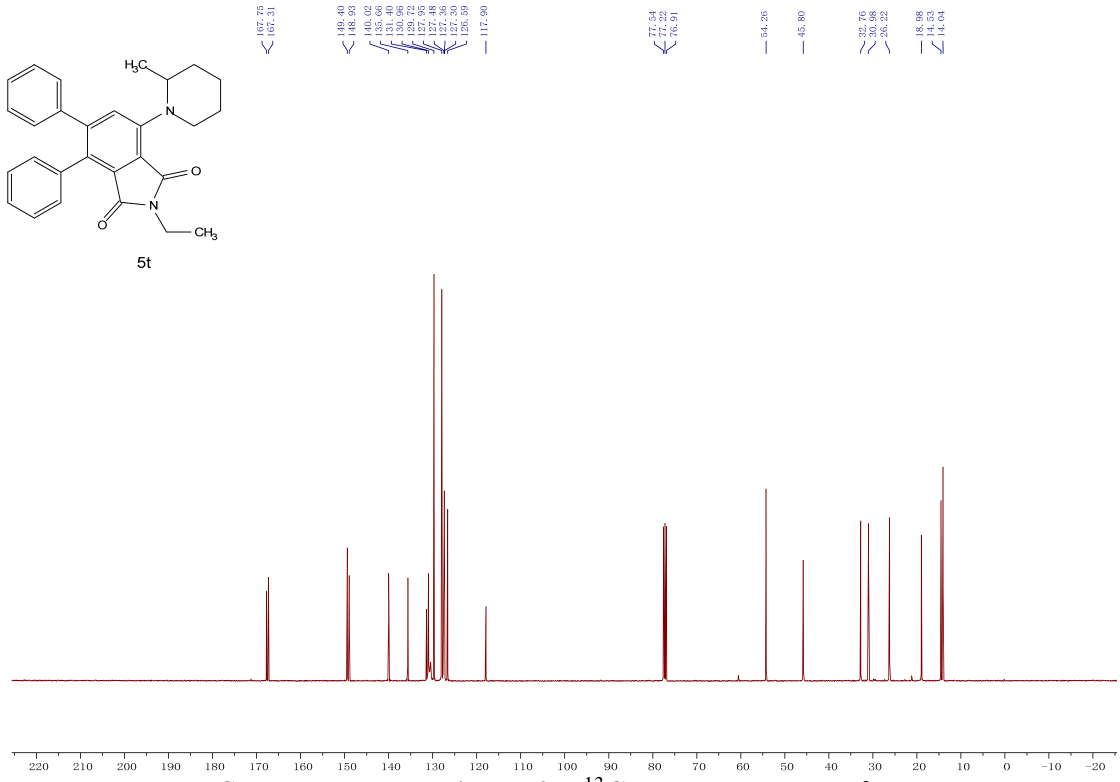

Supplementary Figure 95. ^{13}C NMR spectrum of 5t

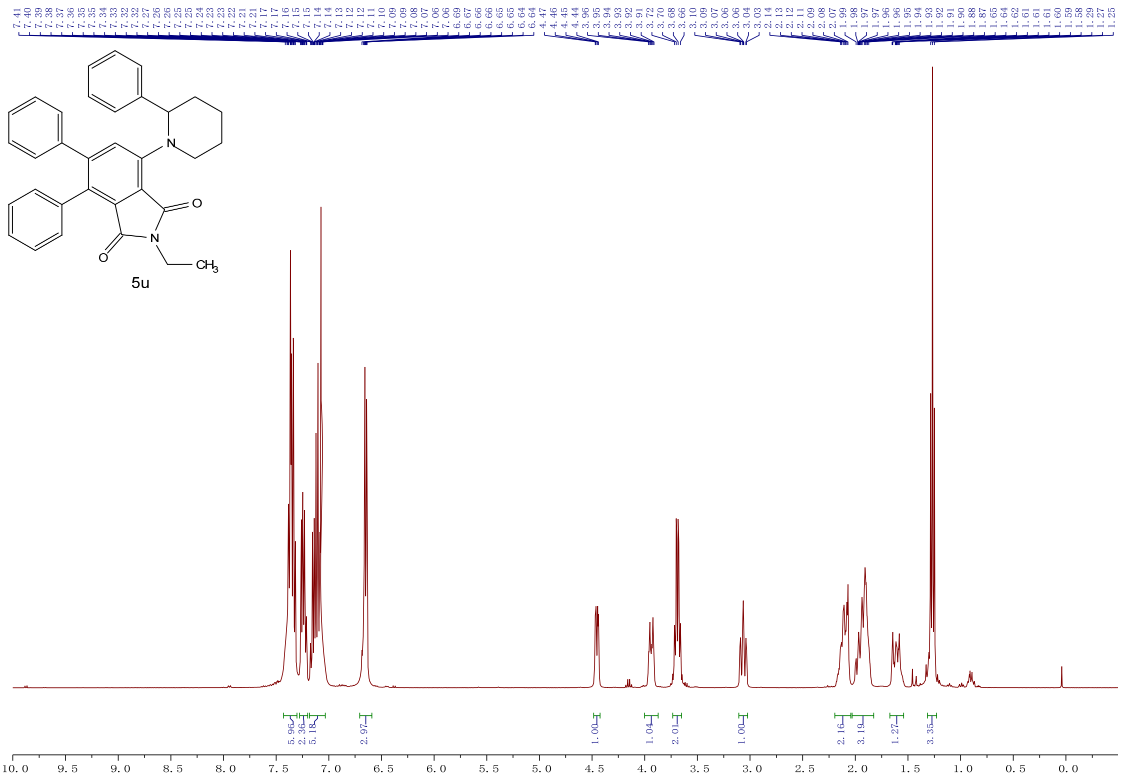

Supplementary Figure 96. ^{1}H NMR spectrum of 5u

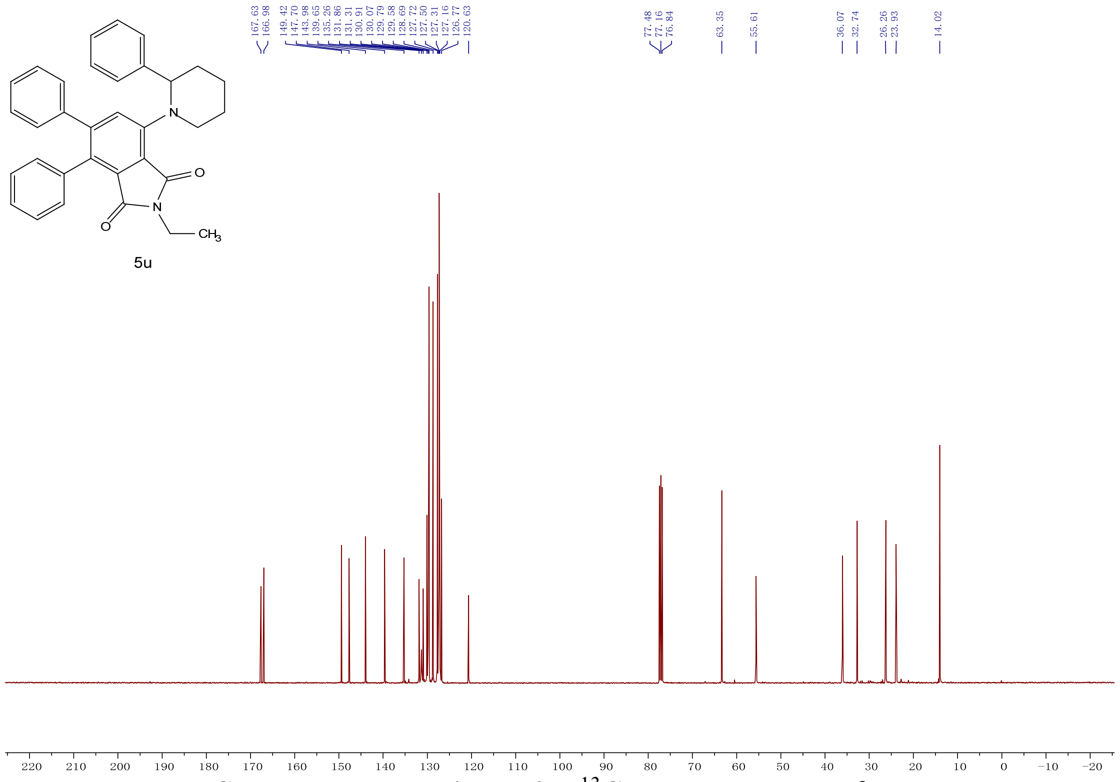

Supplementary Figure 97. ^{13}C NMR spectrum of 5u

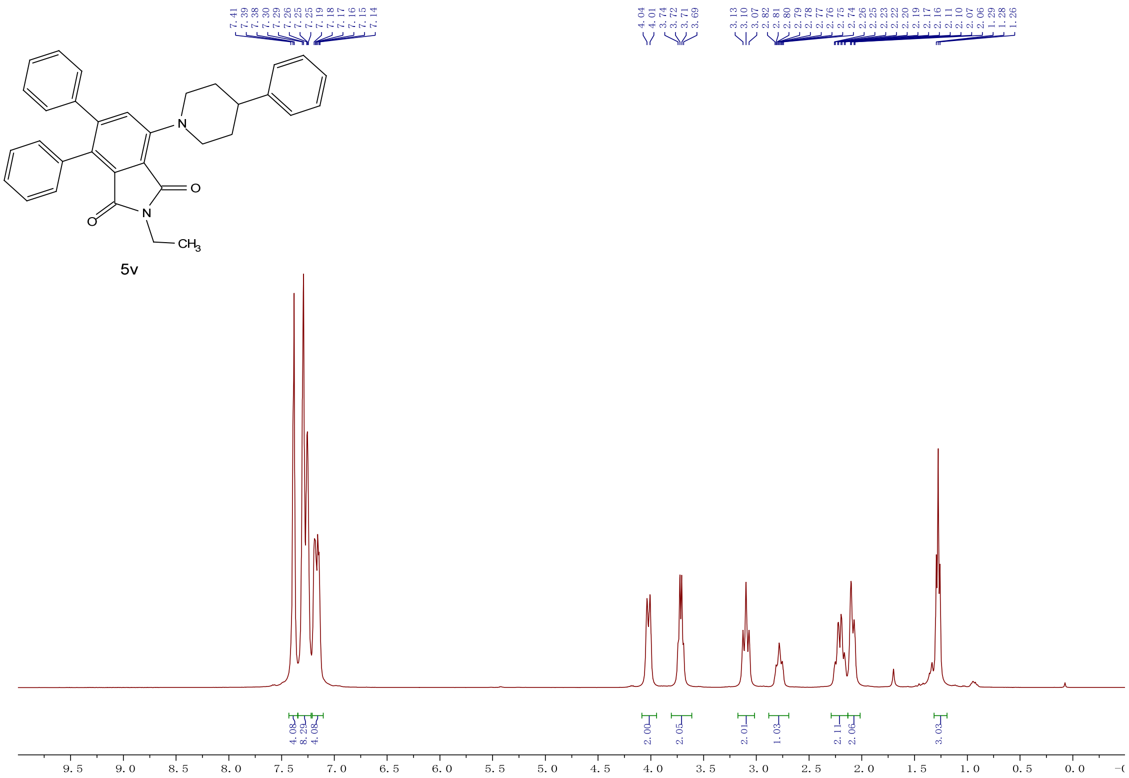

Supplementary Figure 98. ^{1}H NMR spectrum of 5v

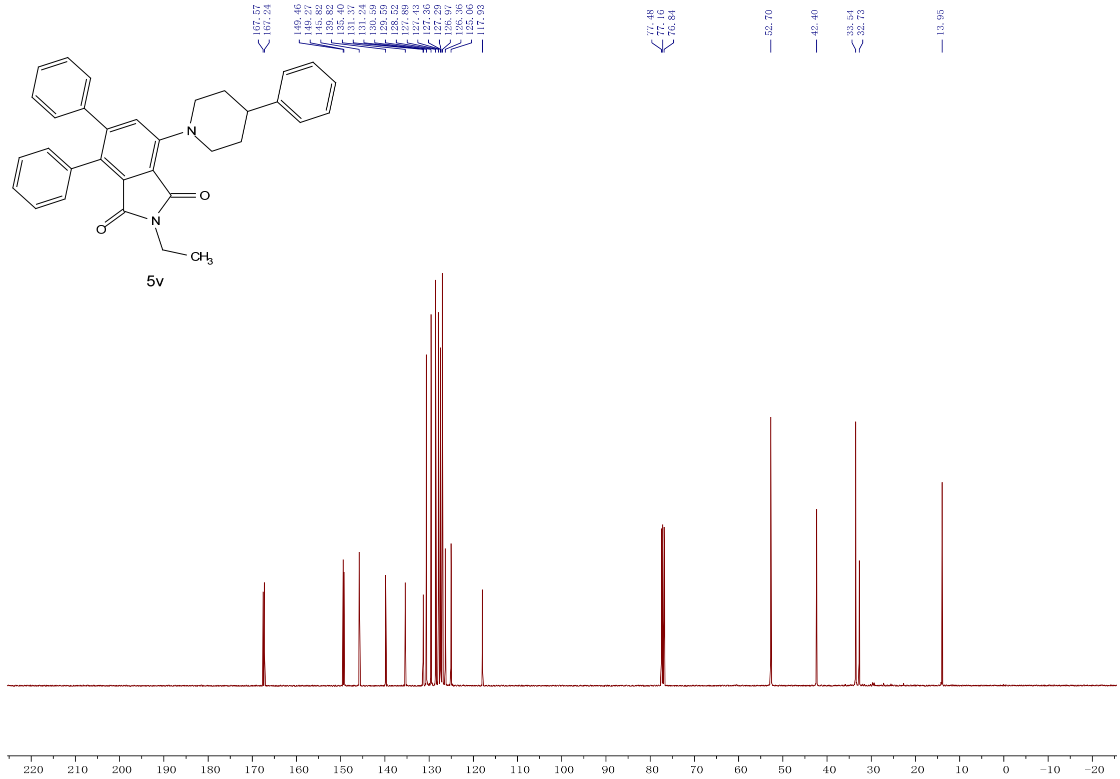

Supplementary Figure 99. ^{13}C NMR spectrum of 5v

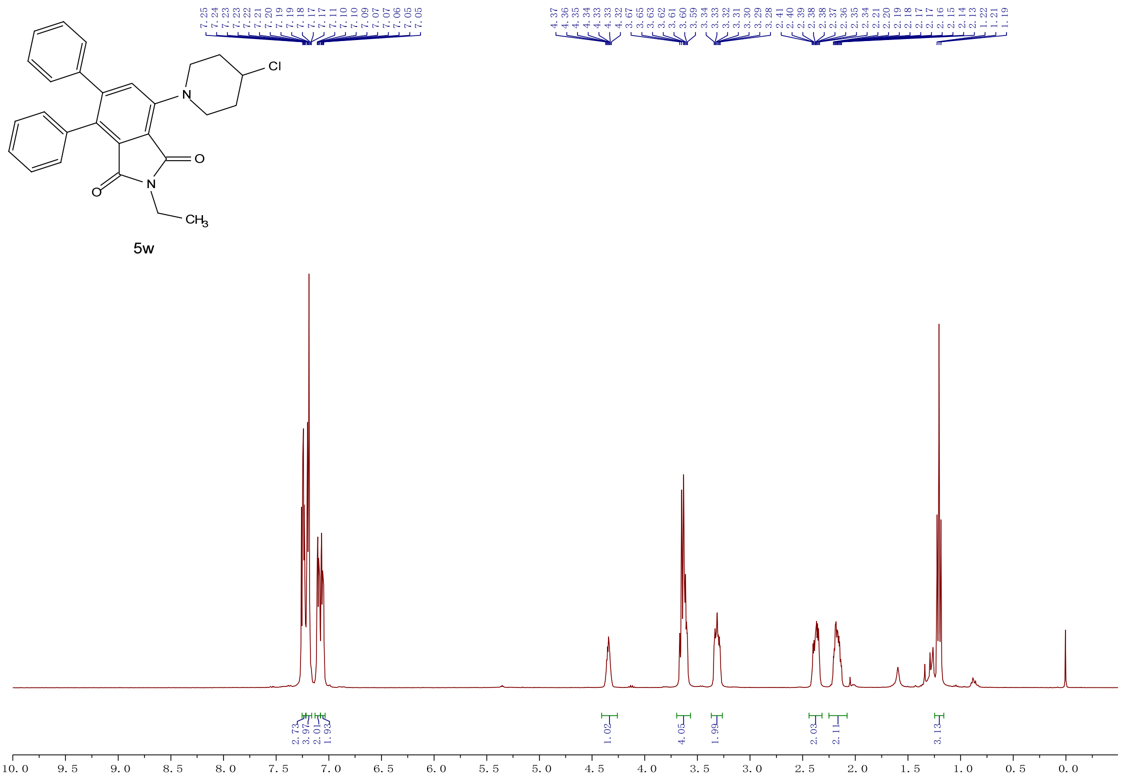

Supplementary Figure 100. ^{1}H NMR spectrum of 5w

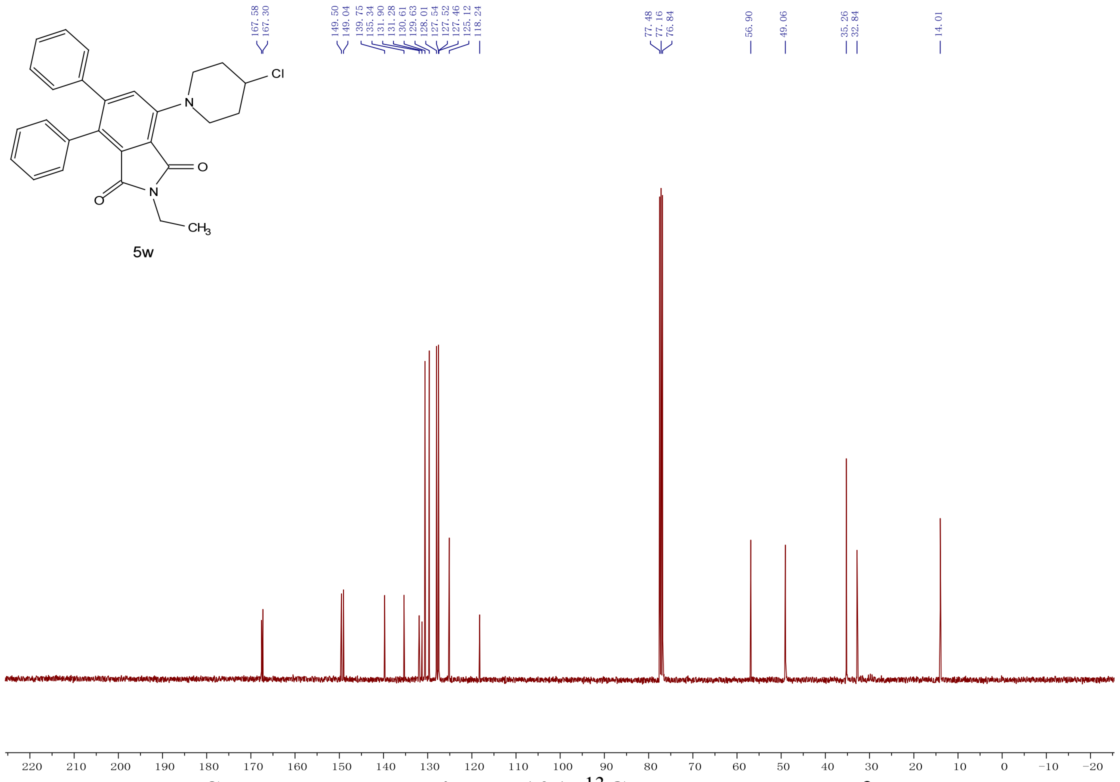

Supplementary Figure 101. ^{13}C NMR spectrum of 5w

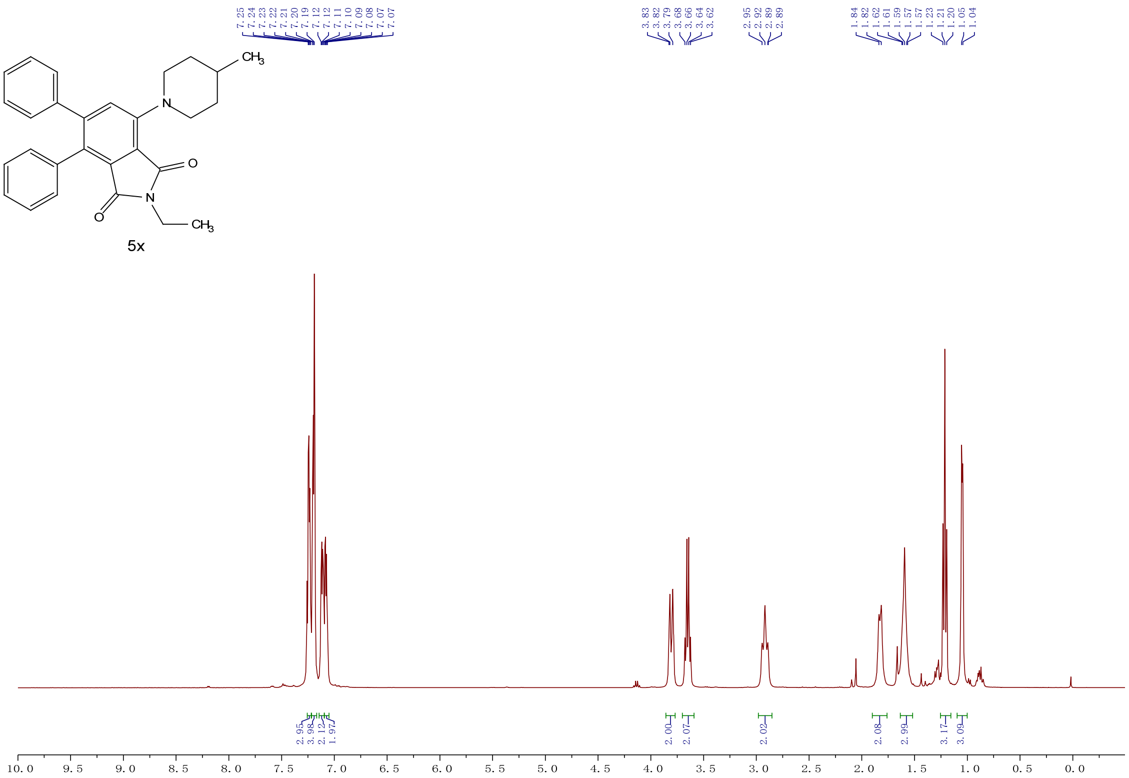

Supplementary Figure 102. ^{1}H NMR spectrum of 5x

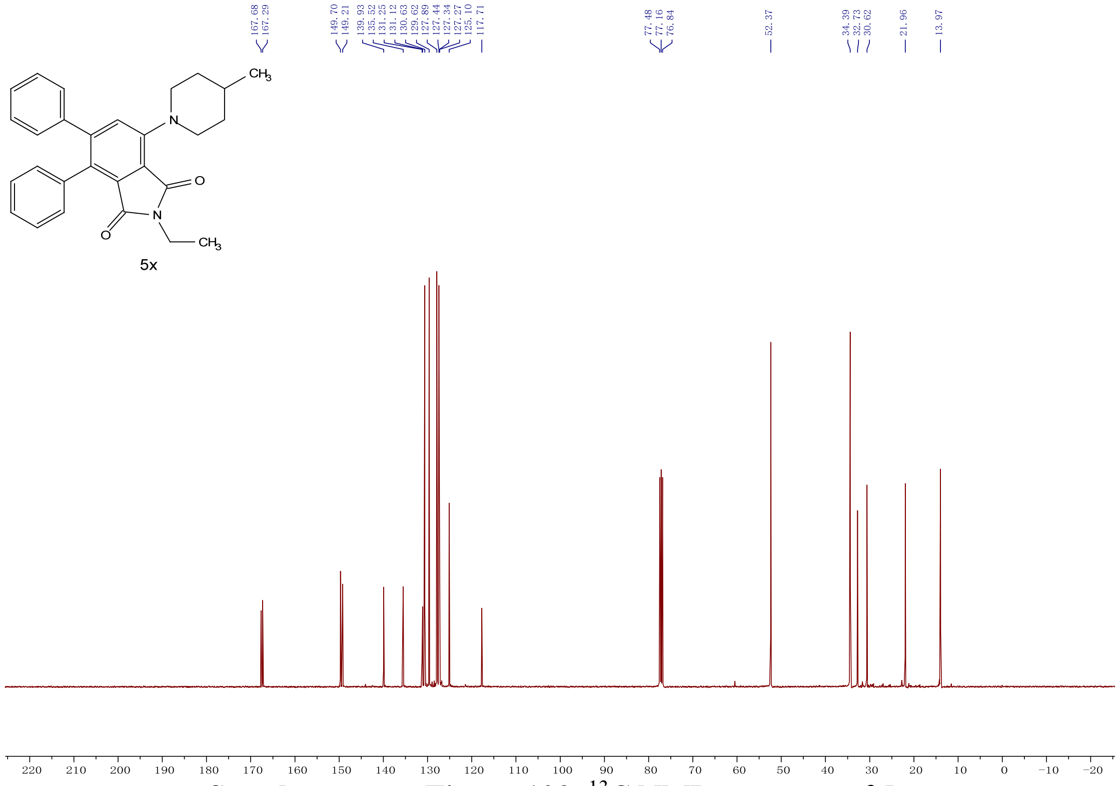

Supplementary Figure 103. ^{13}C NMR spectrum of 5x

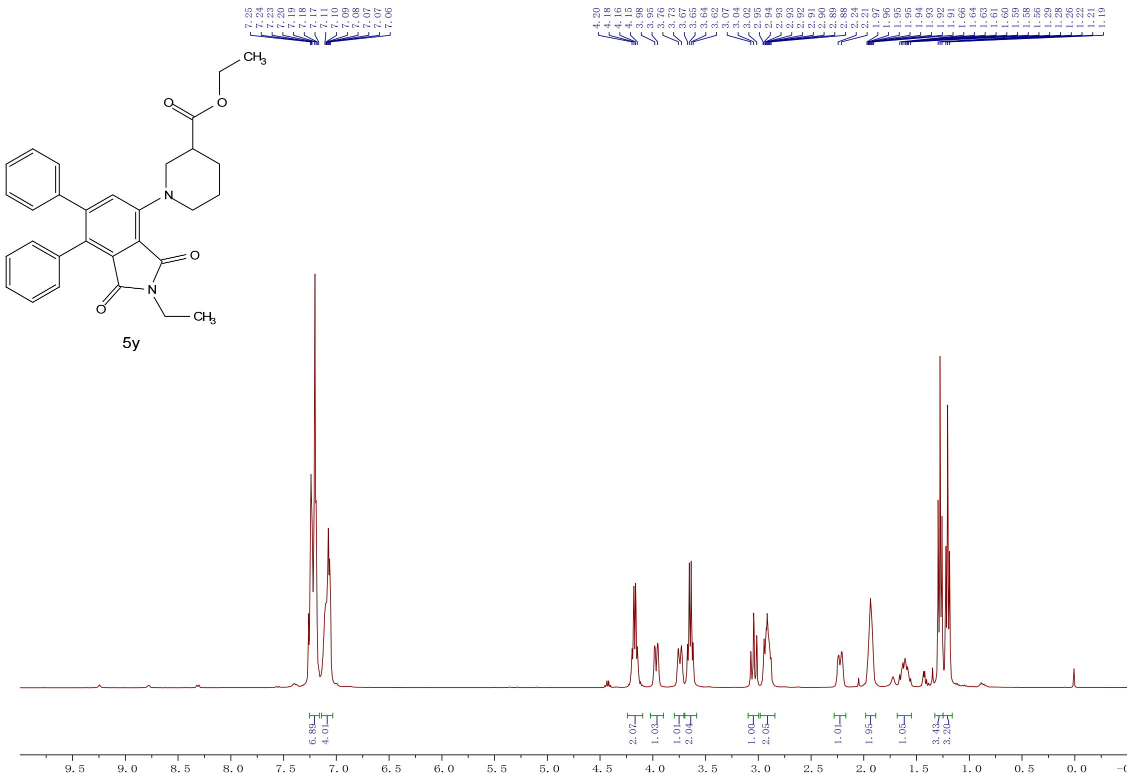

Supplementary Figure 104. ^{1}H NMR spectrum of 5y

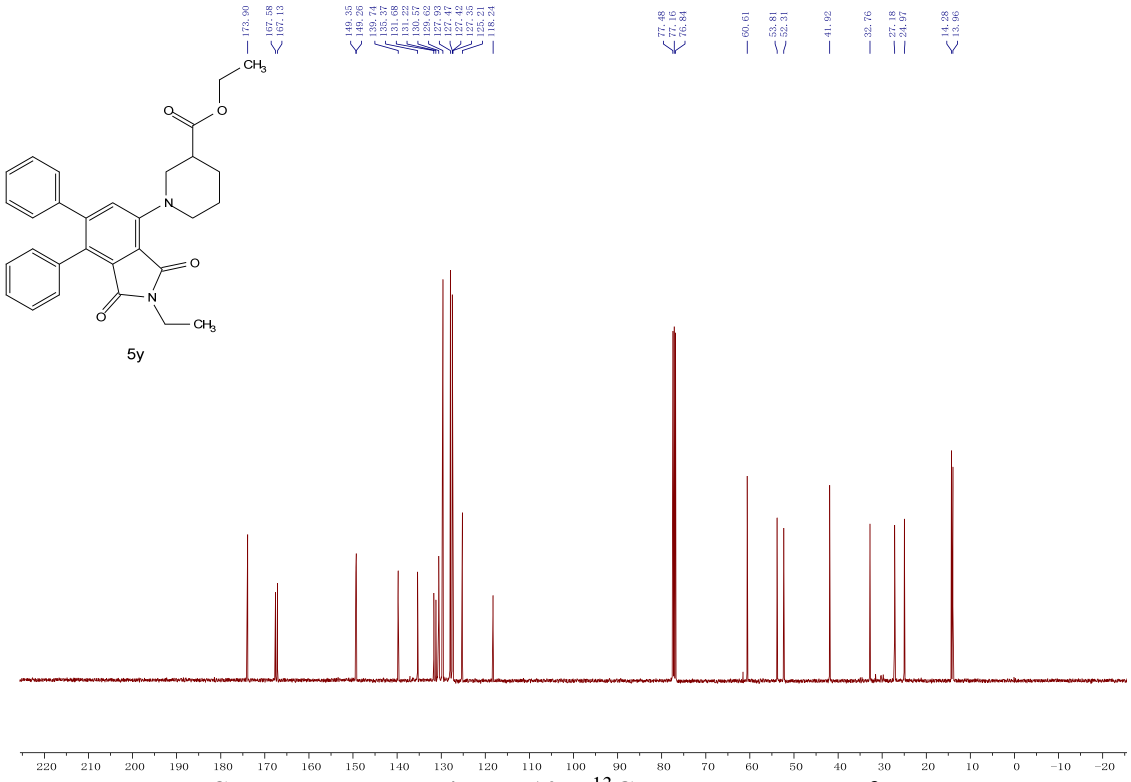

Supplementary Figure 105. ^{13}C NMR spectrum of 5y

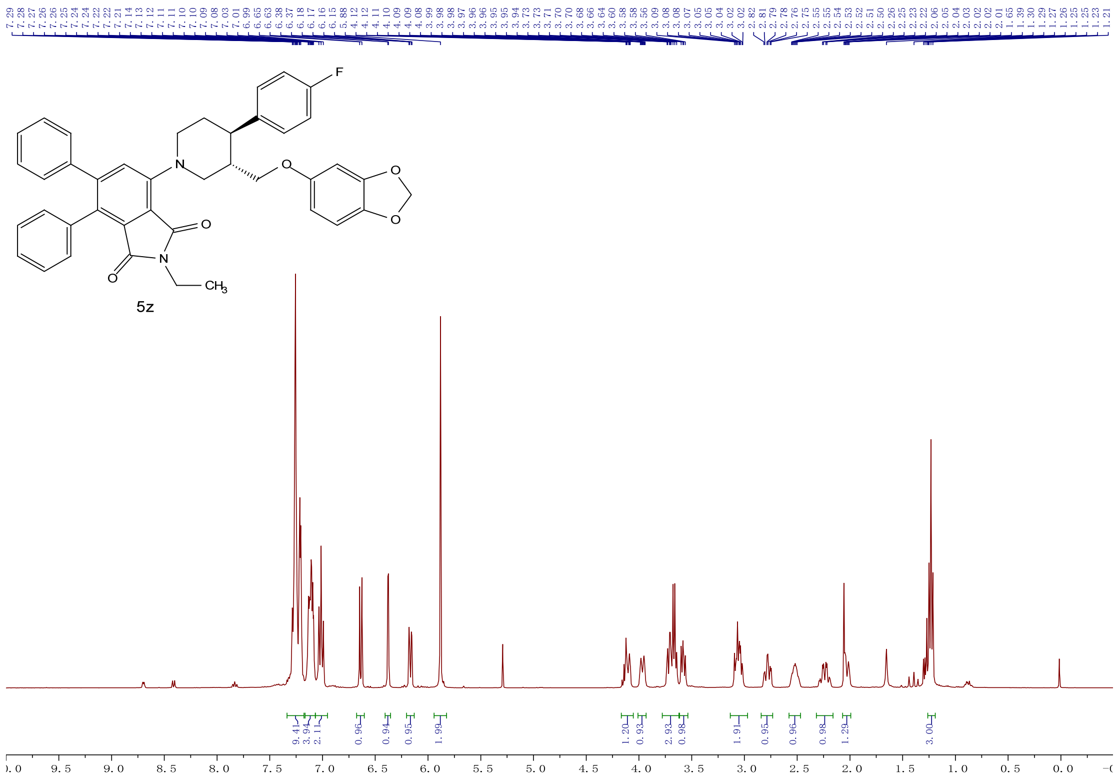

Supplementary Figure 106. ^{1}H NMR spectrum of 5z

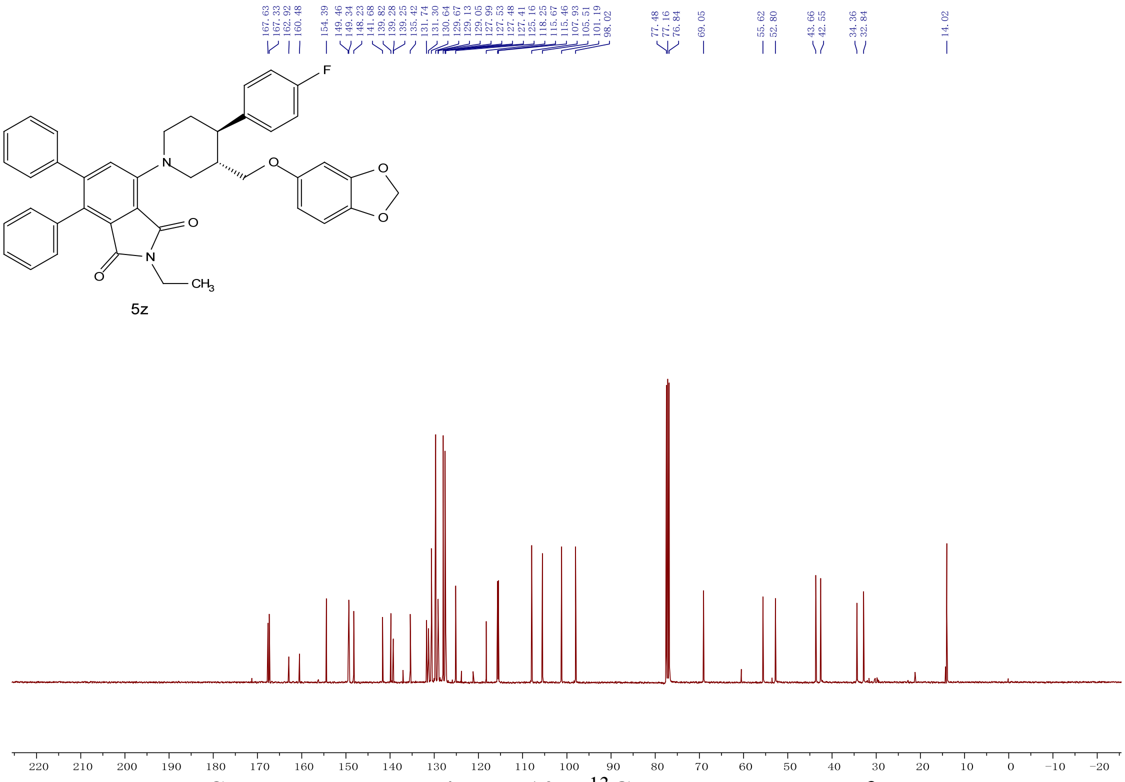

Supplementary Figure 107. ^{13}C NMR spectrum of 5z

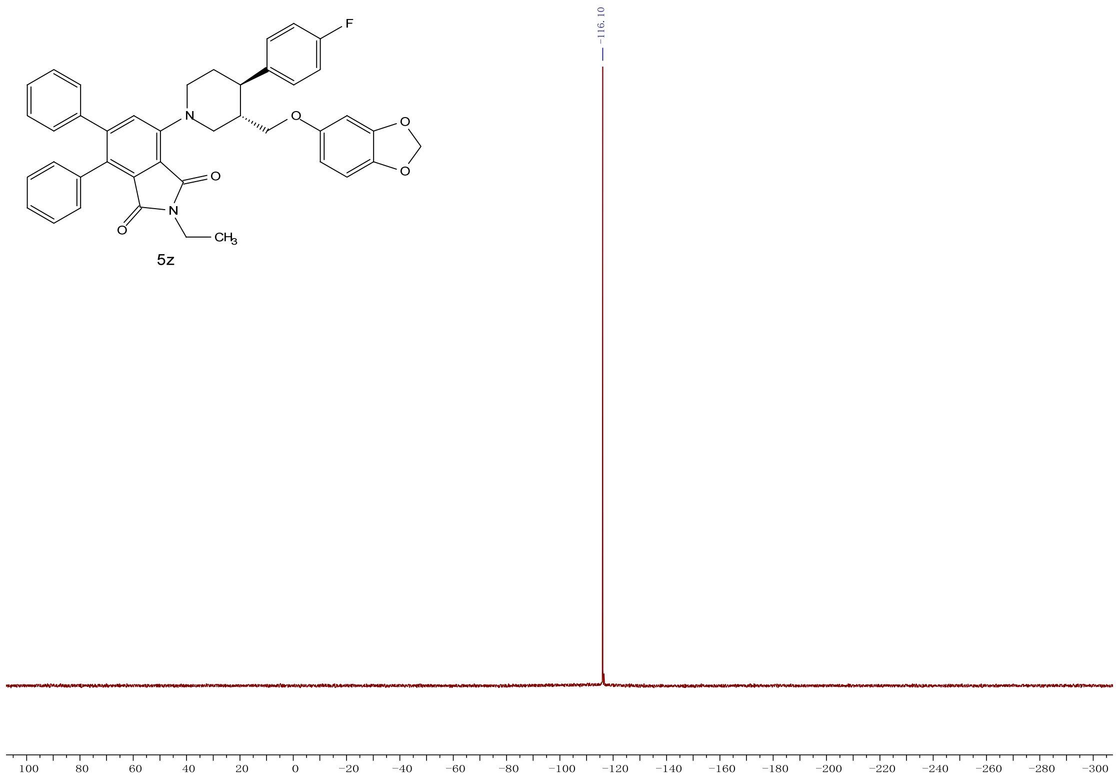

Supplementary Figure 108. ^{19}F NMR spectrum of 5z

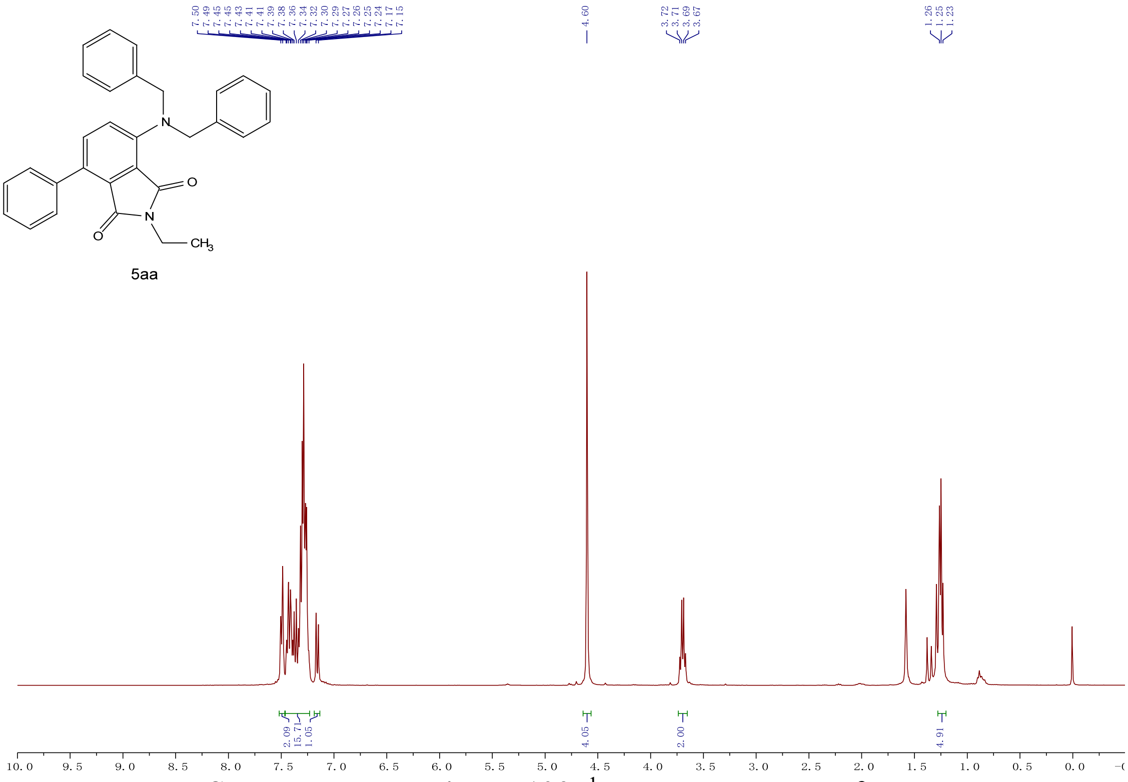

Supplementary Figure 109. ^{1}H NMR spectrum of 5aa

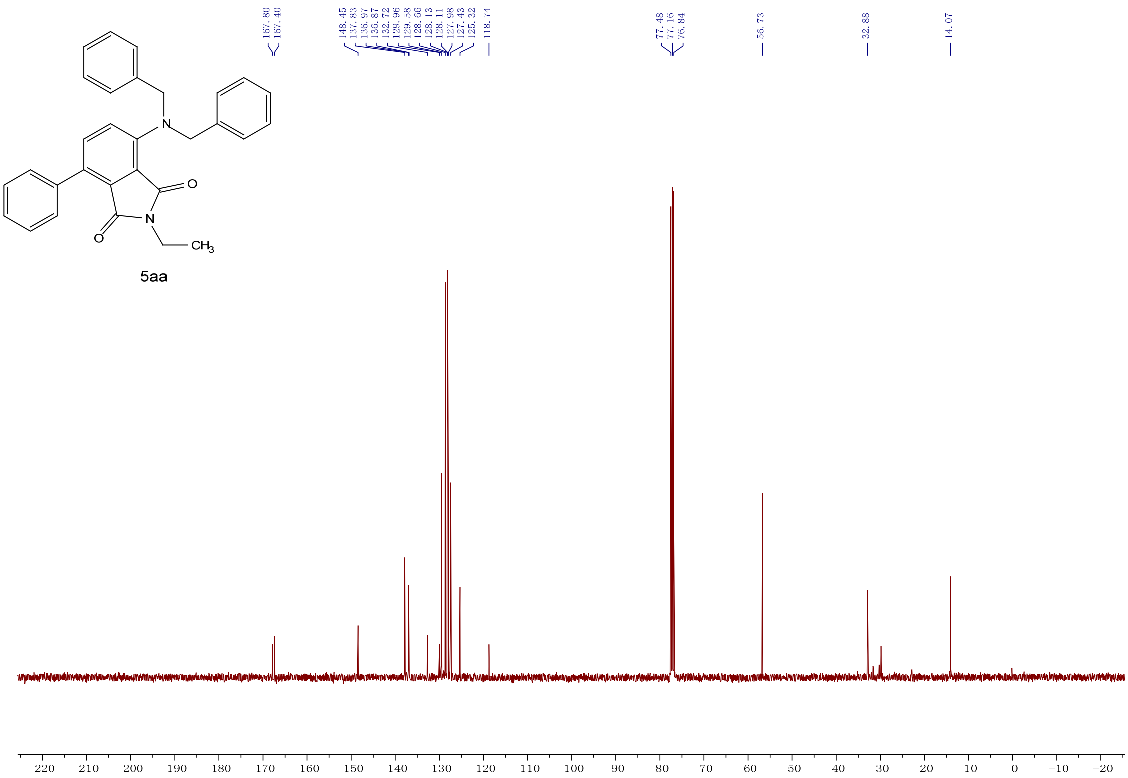

Supplementary Figure 110. ^{13}C NMR spectrum of 5aa

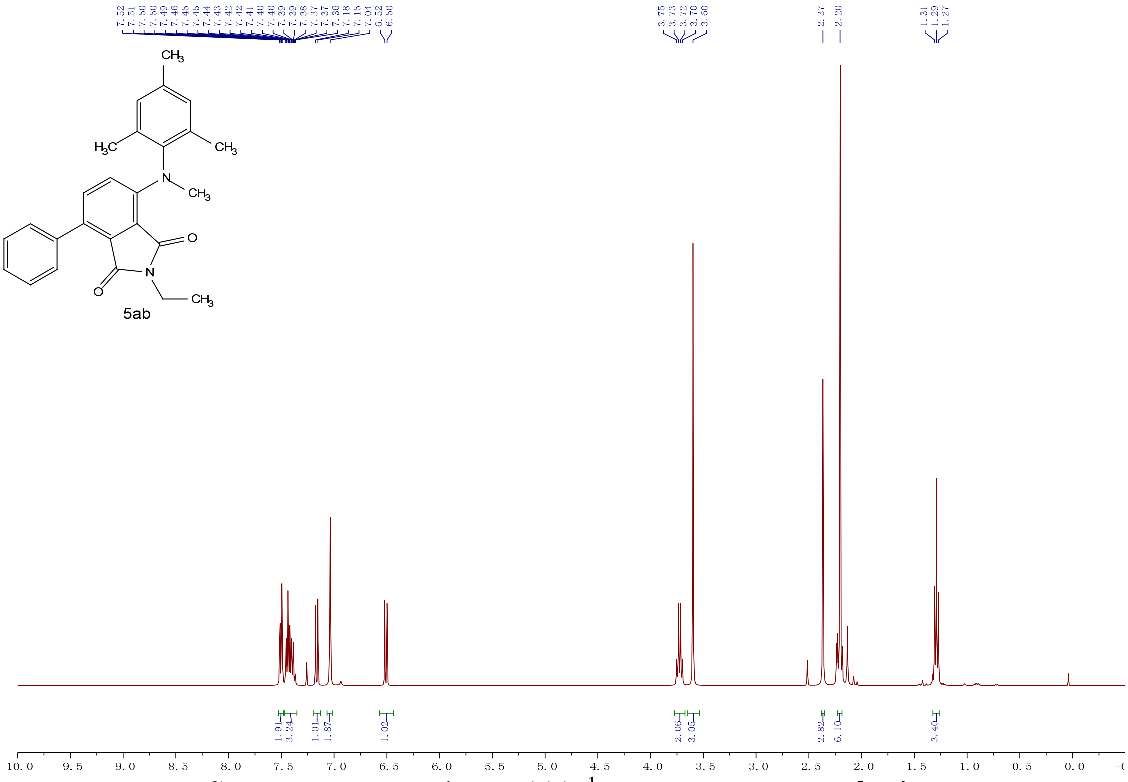

Supplementary Figure 111. ^{1}H NMR spectrum of 5ab

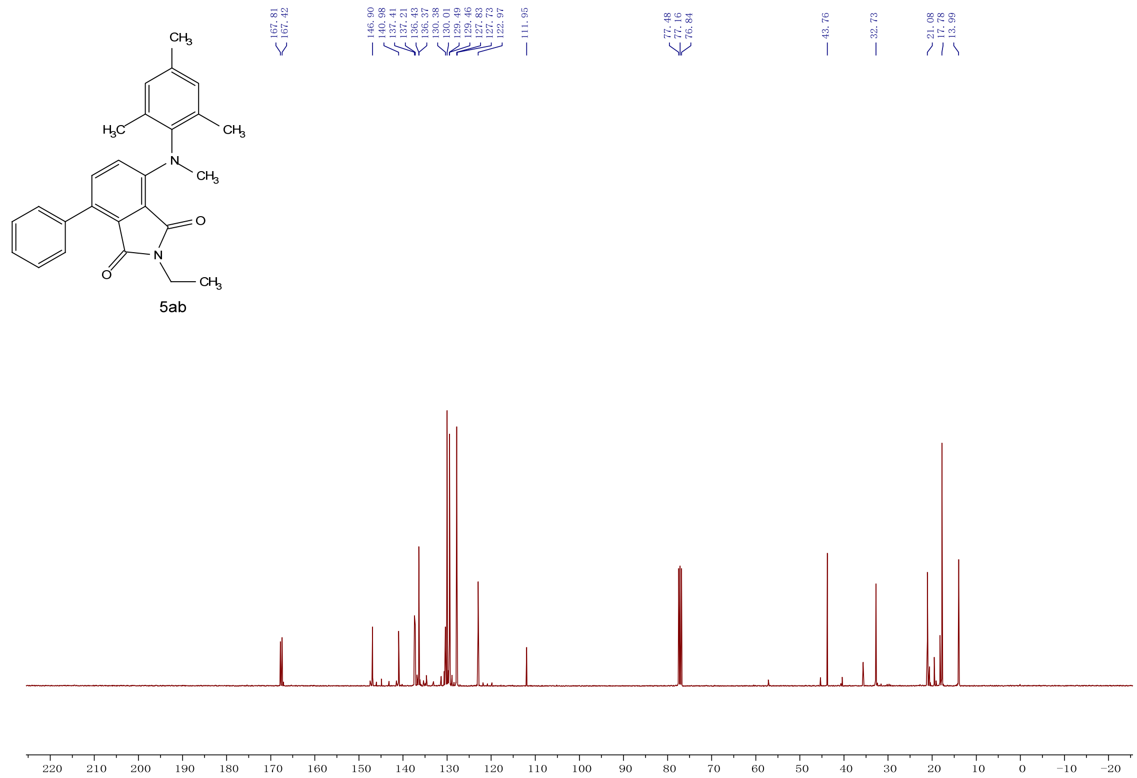

Supplementary Figure 112. ^{13}C NMR spectrum of 5ab

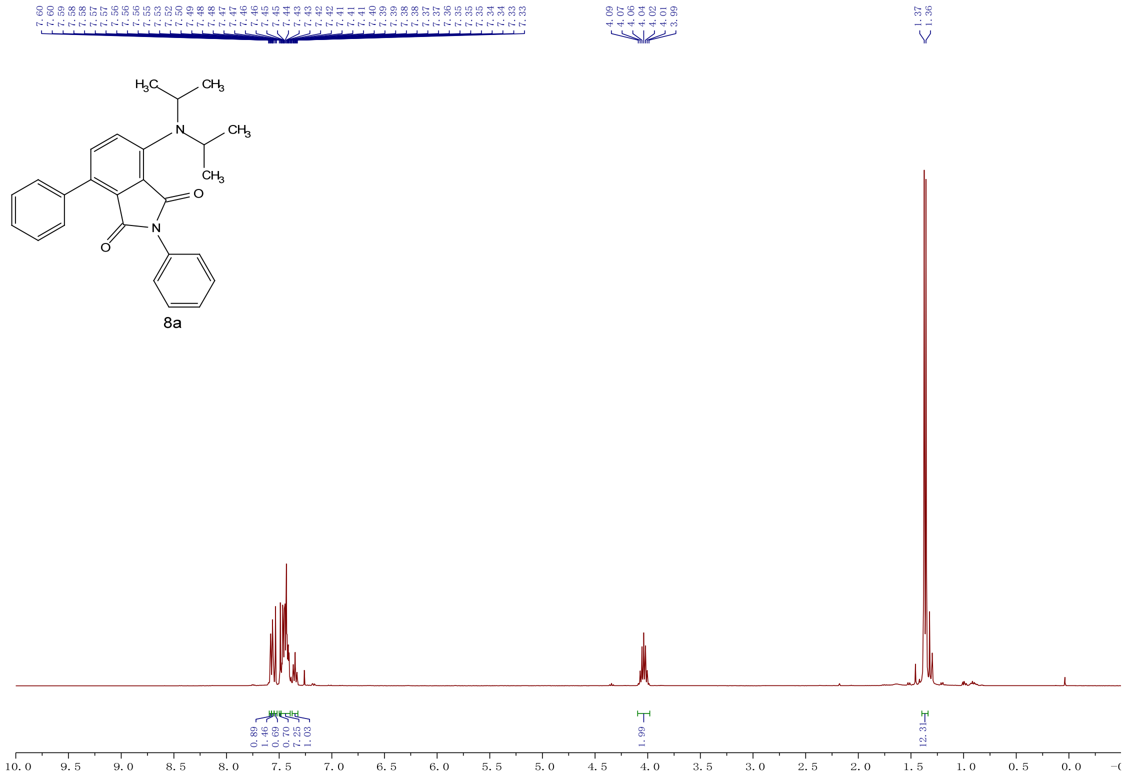

Supplementary Figure 113. ^{1}H NMR spectrum of 8a

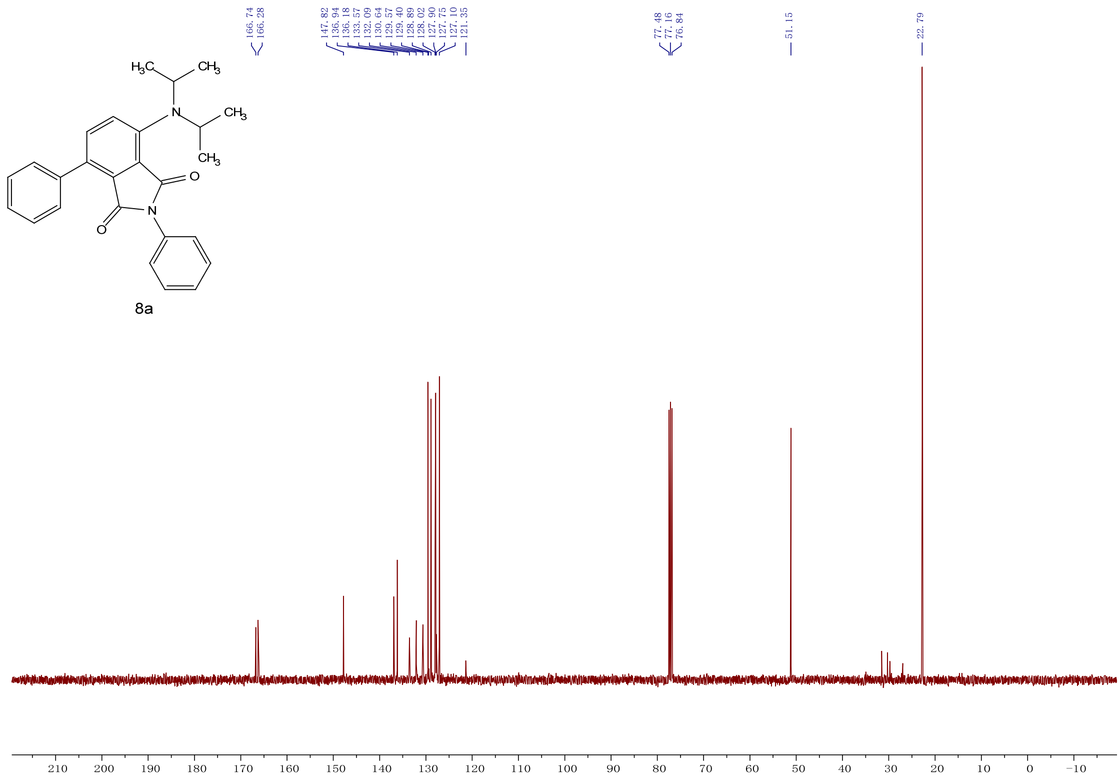

Supplementary Figure 114. ^{13}C NMR spectrum of 8a

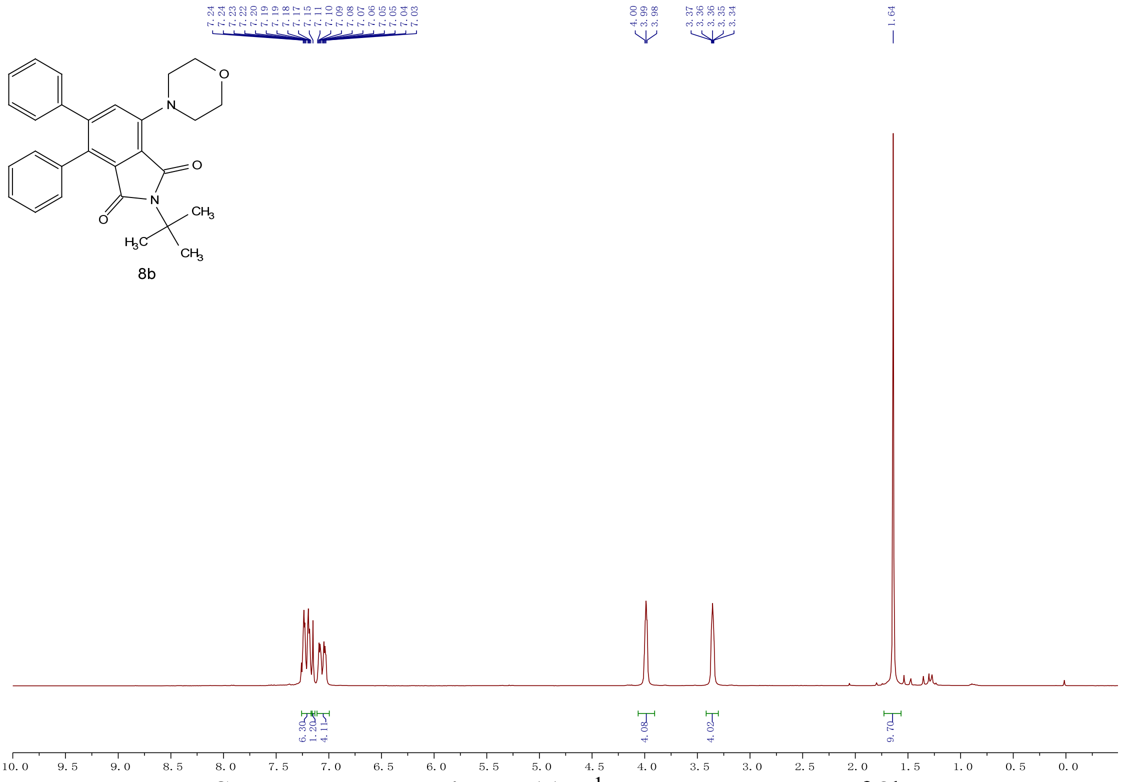

Supplementary Figure 115. ^{1}H NMR spectrum of 8b

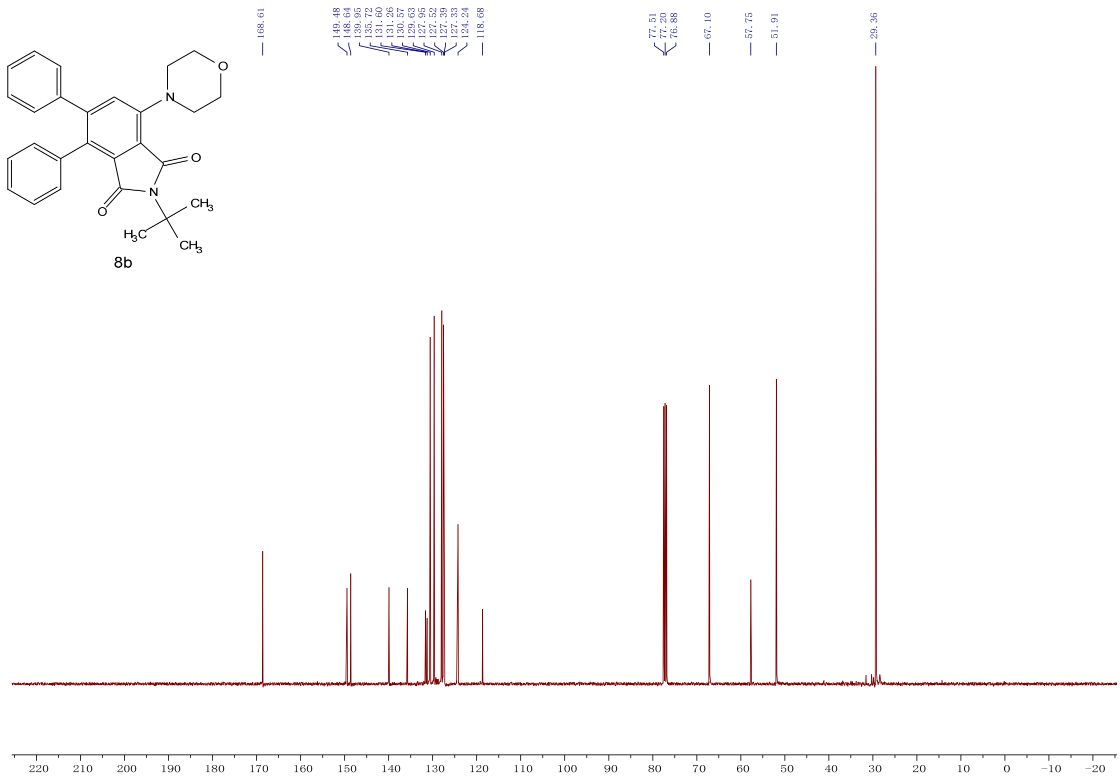

Supplementary Figure 116. ^{13}C NMR spectrum of 8b

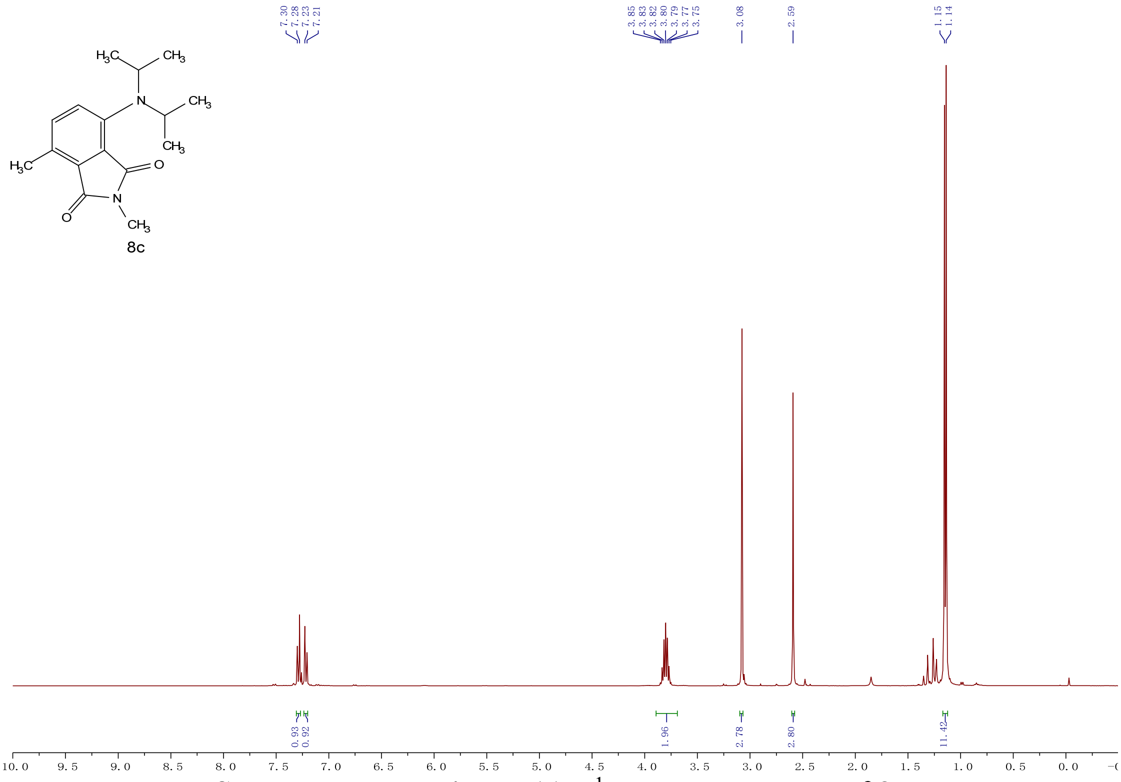

Supplementary Figure 117. ^{1}H NMR spectrum of 8c

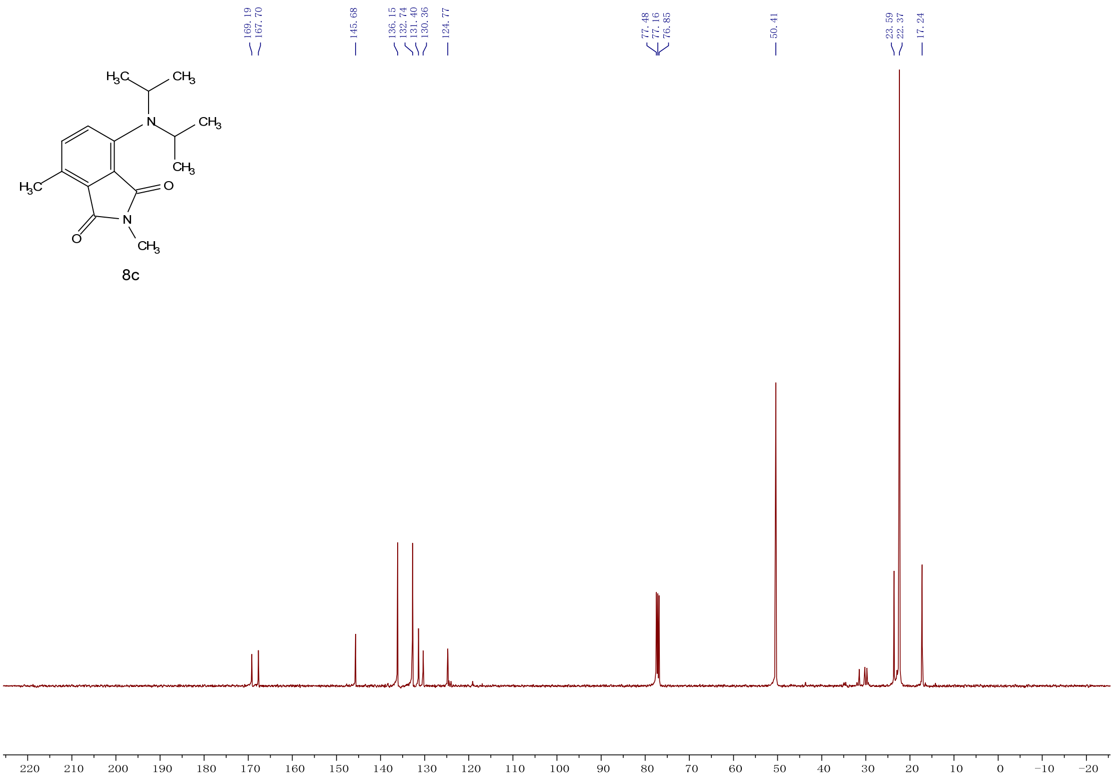

Supplementary Figure 118. ^{13}C NMR spectrum of 8c

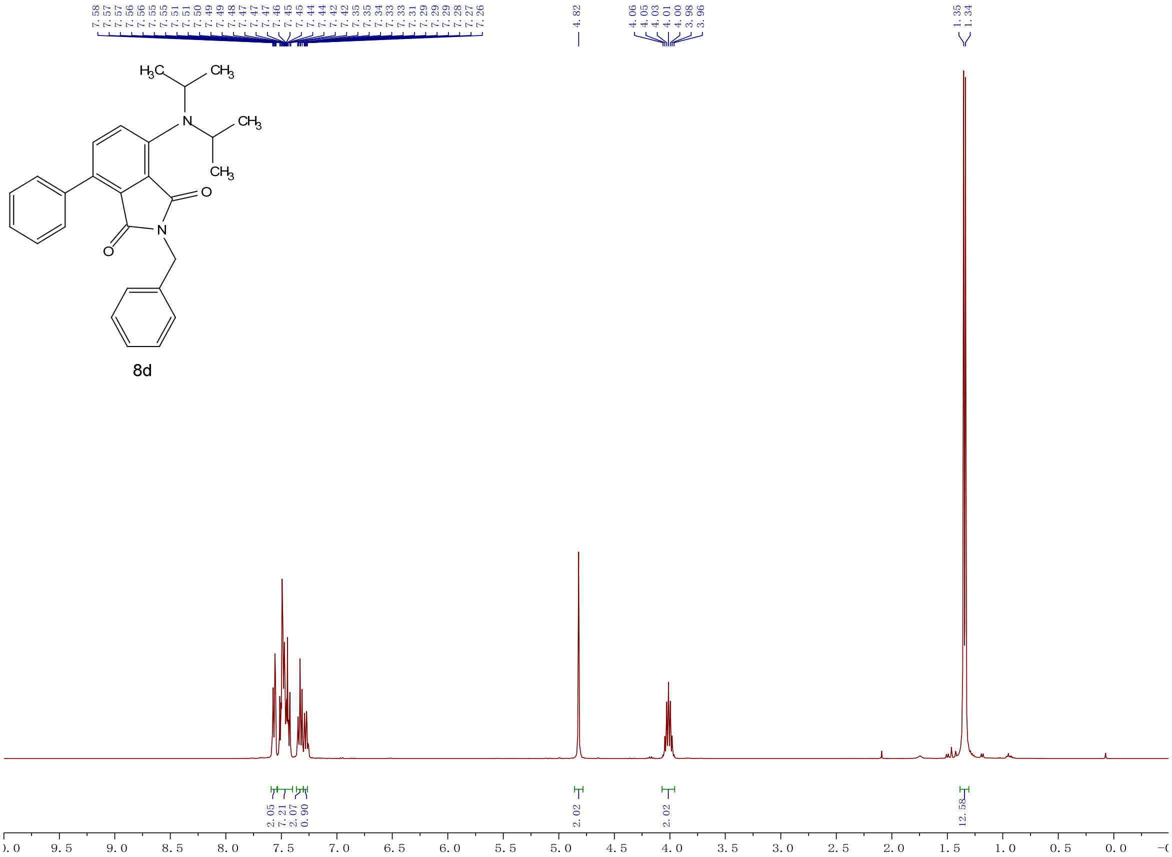

Supplementary Figure 119. ^{1}H NMR spectrum of 8d

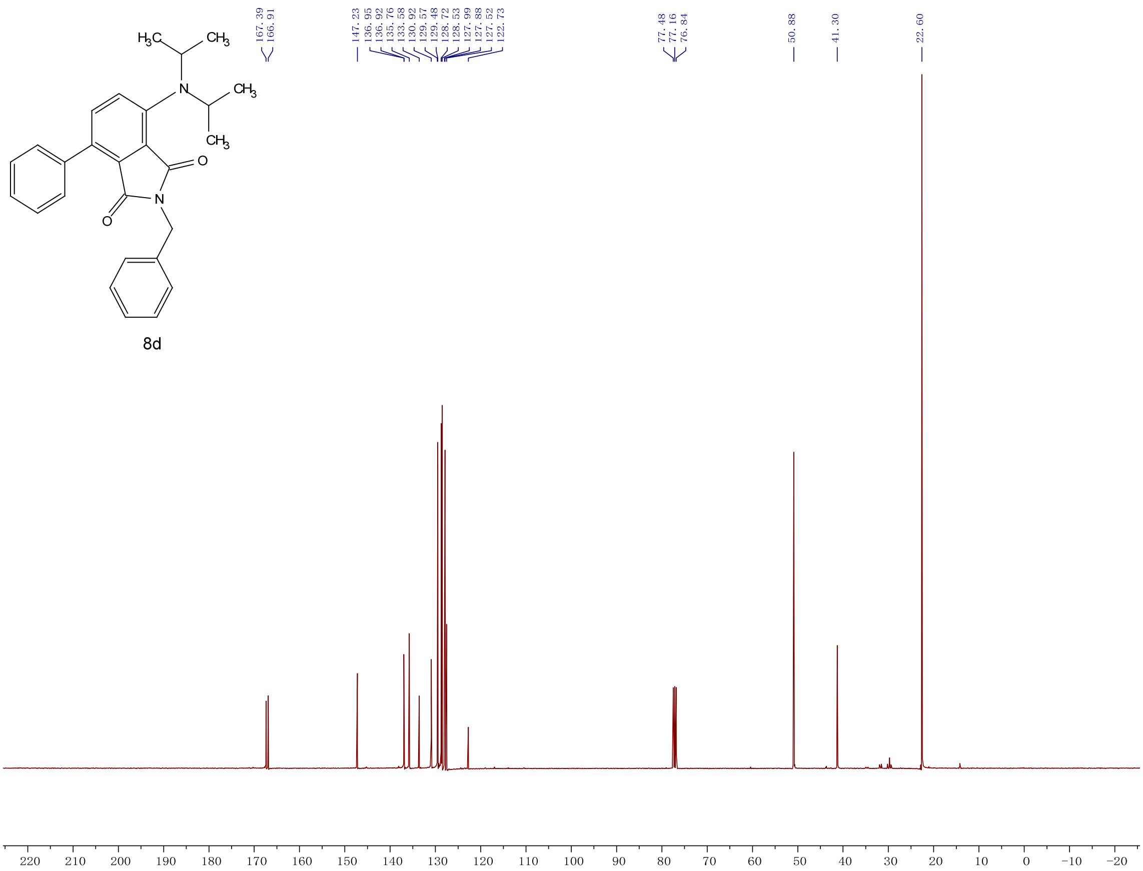

Supplementary Figure 120. ^{13}C NMR spectrum of 8d

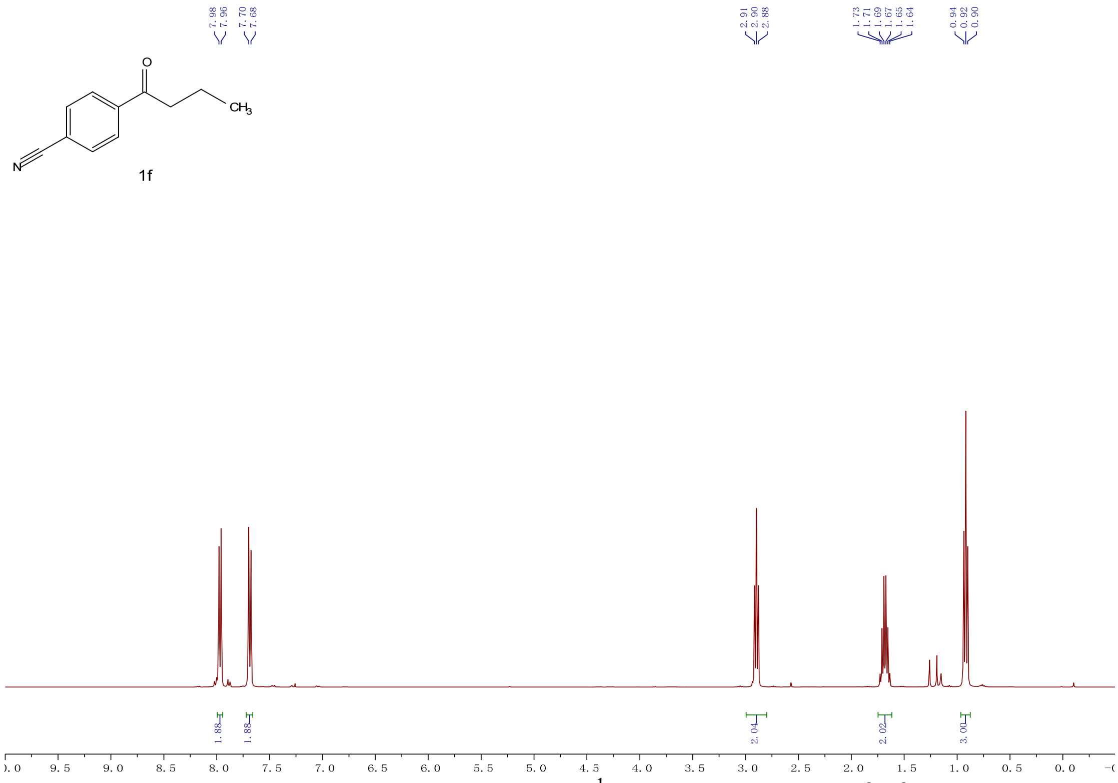

Supplementary Figure 121. ^{1}H NMR spectrum of 1f

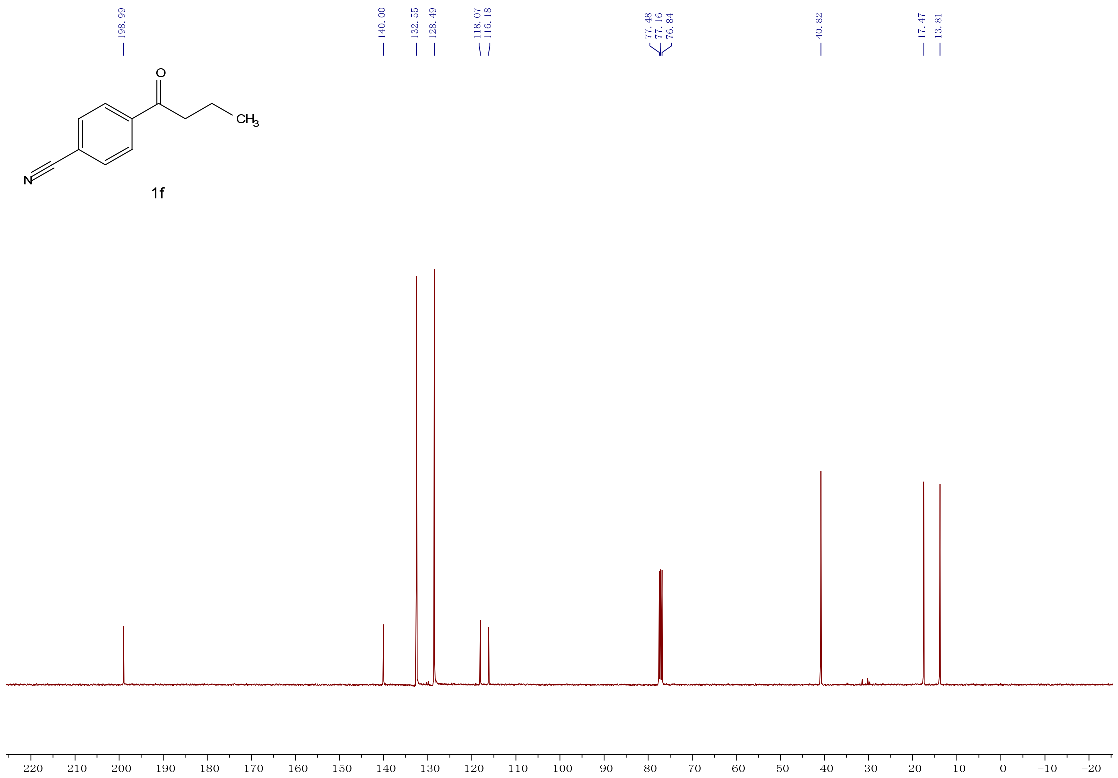

Supplementary Figure 122. ^{13}C NMR spectrum of 1f

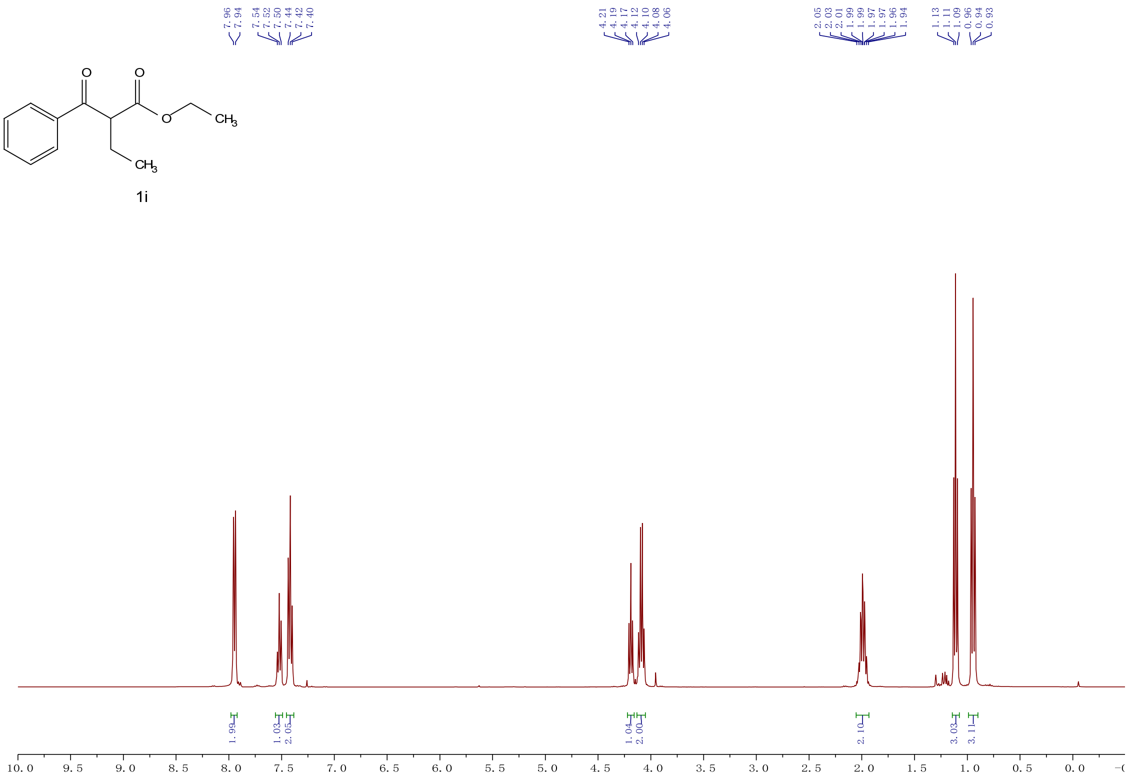

Supplementary Figure 123. ^{1}H NMR spectrum of 1i

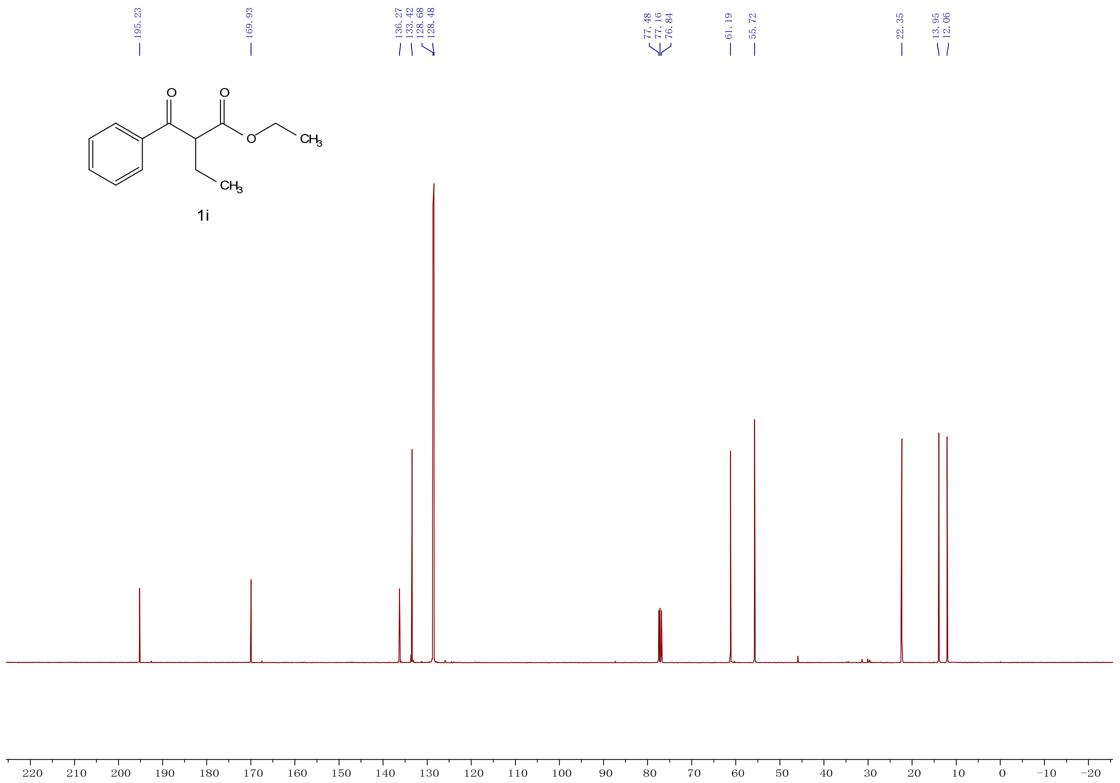

Supplementary Figure 124. ^{13}C NMR spectrum of 1i

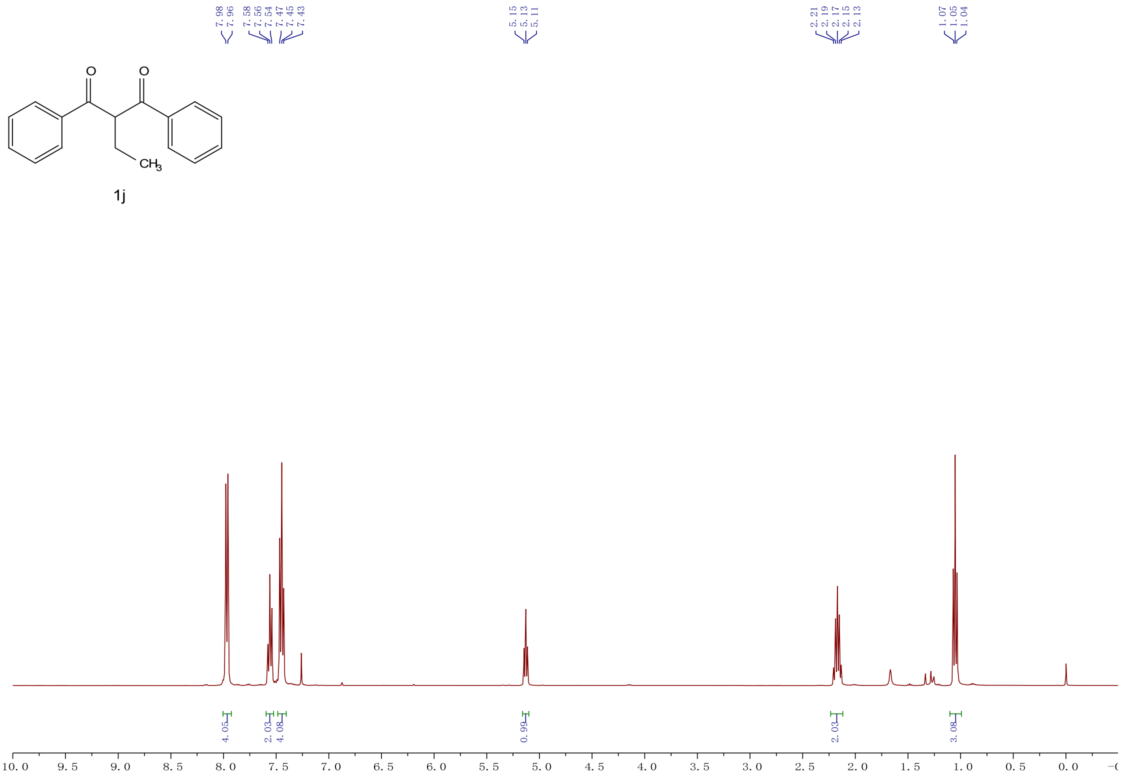

Supplementary Figure 125. ^{1}H NMR spectrum of 1j

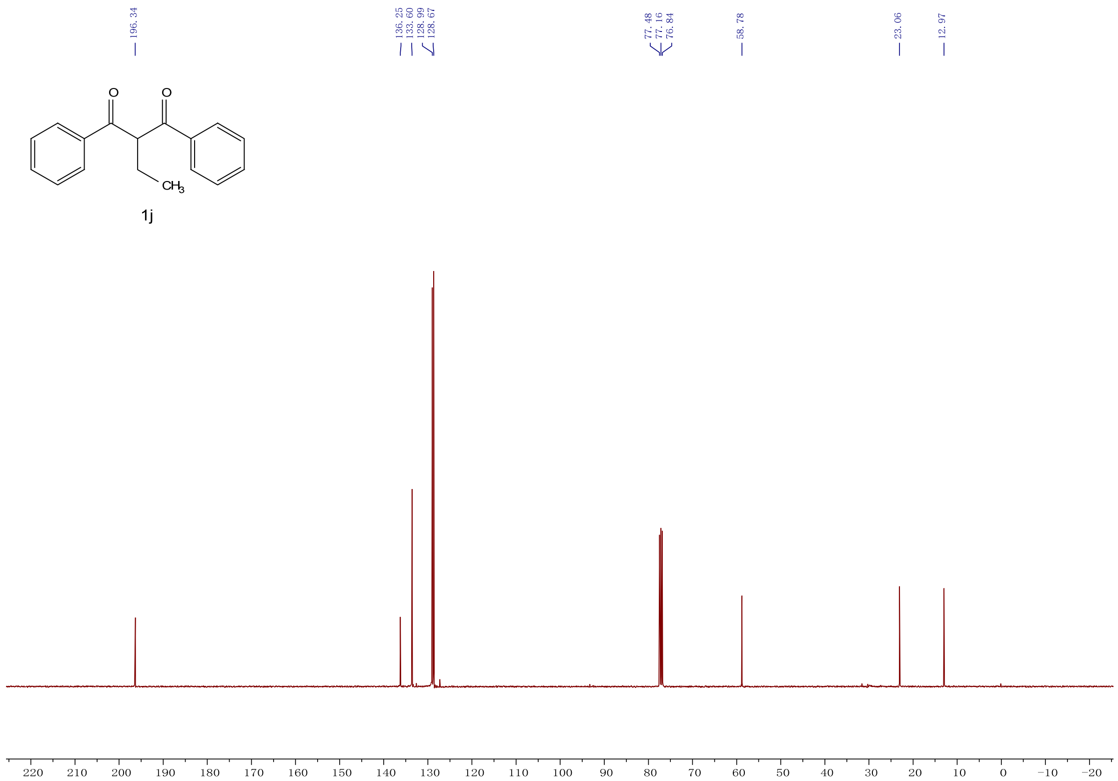

Supplementary Figure 126. ^{13}C NMR spectrum of 1j

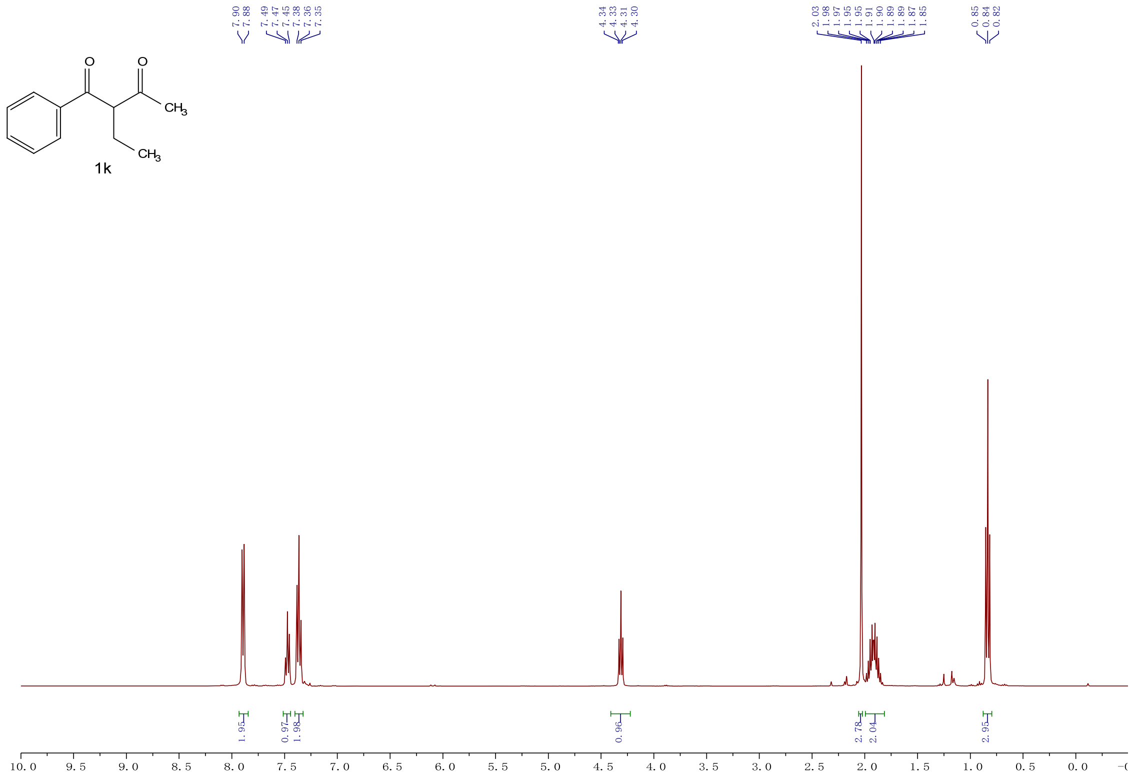

Supplementary Figure 127. ^{1}H NMR spectrum of 1k

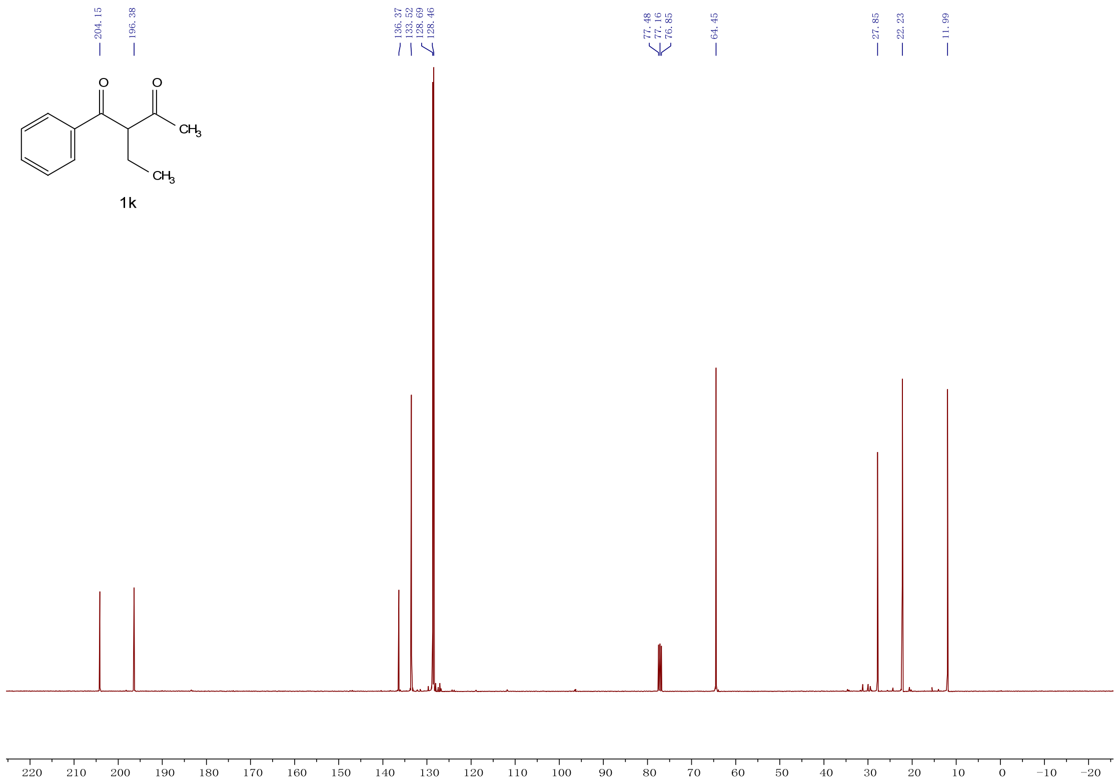

Supplementary Figure 128. ^{13}C NMR spectrum of 1k

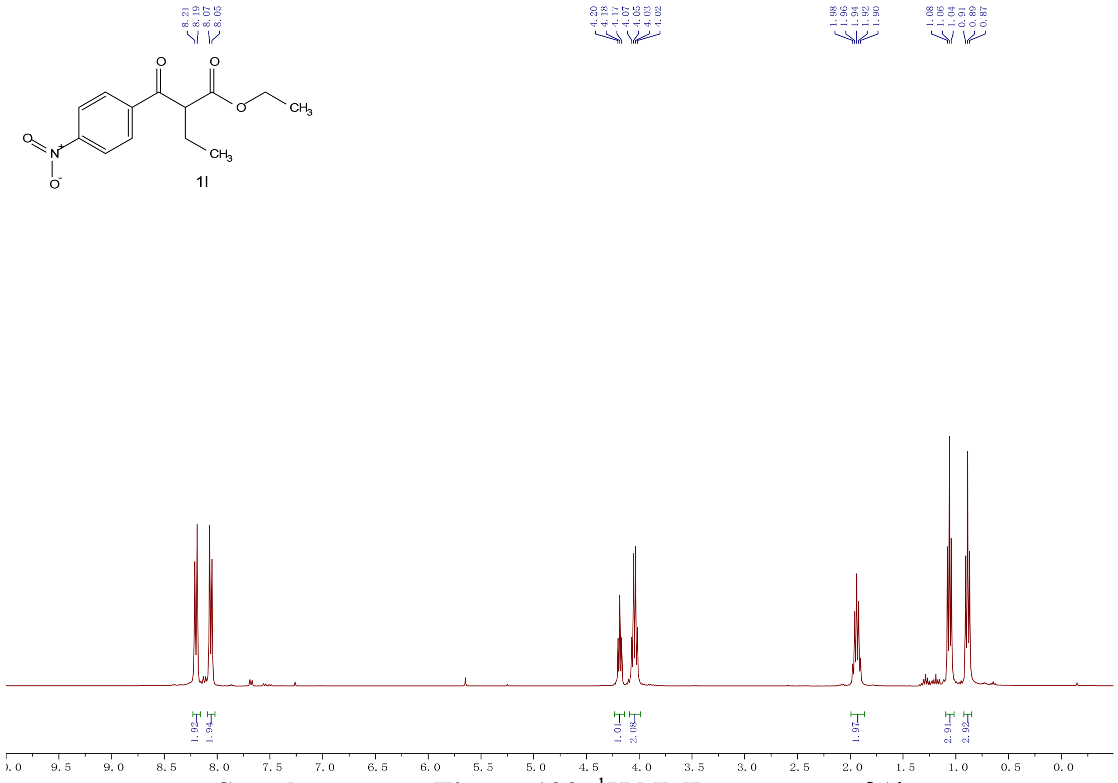

Supplementary Figure 129. ^{1}H NMR spectrum of 1l

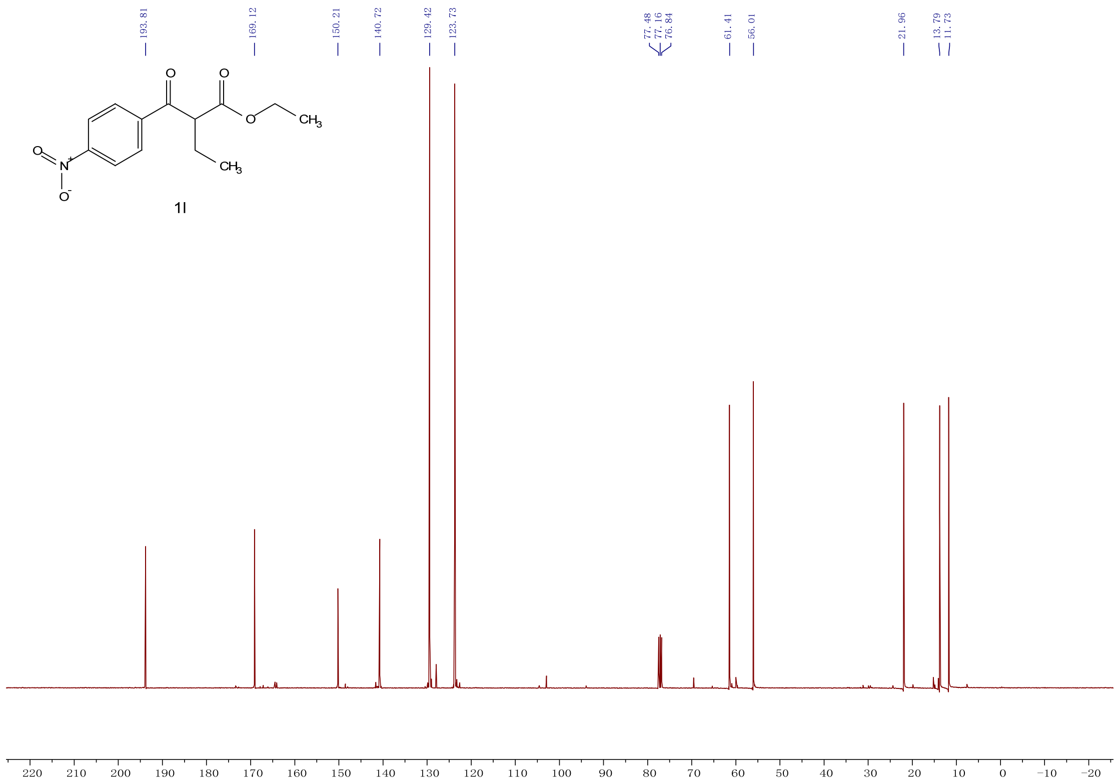

Supplementary Figure 130. ^{13}C NMR spectrum of 1l

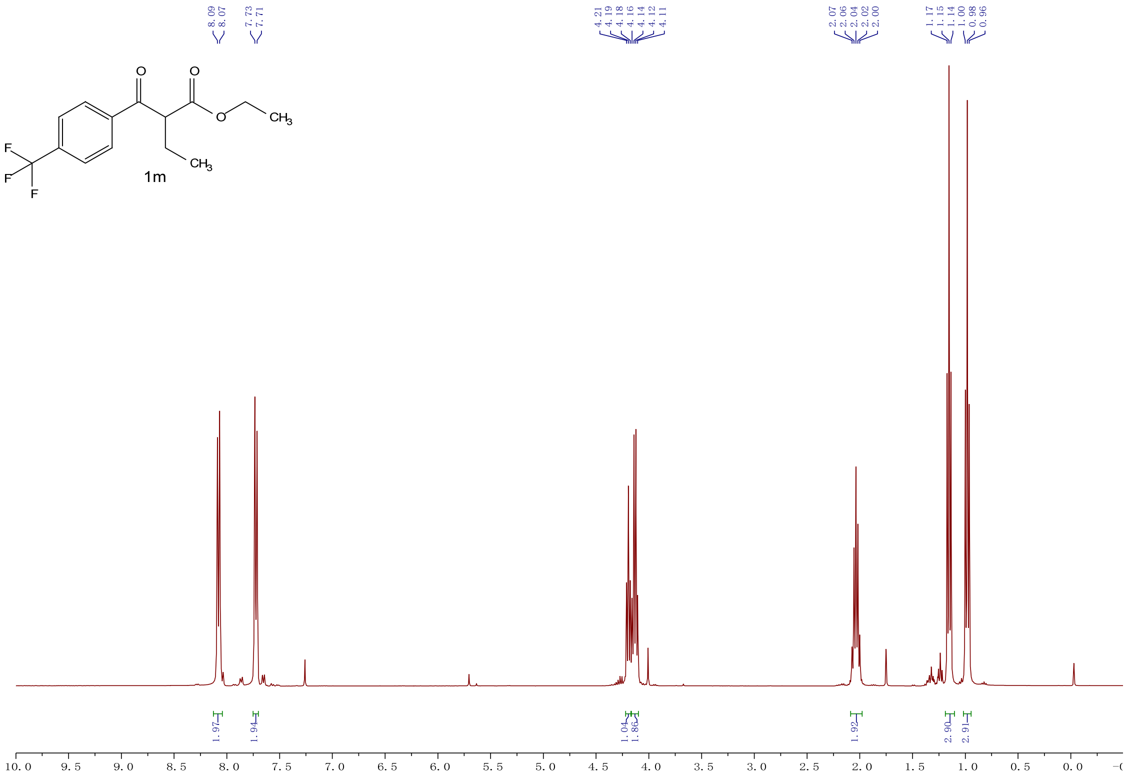

Supplementary Figure 131. ^{1}H NMR spectrum of 1m

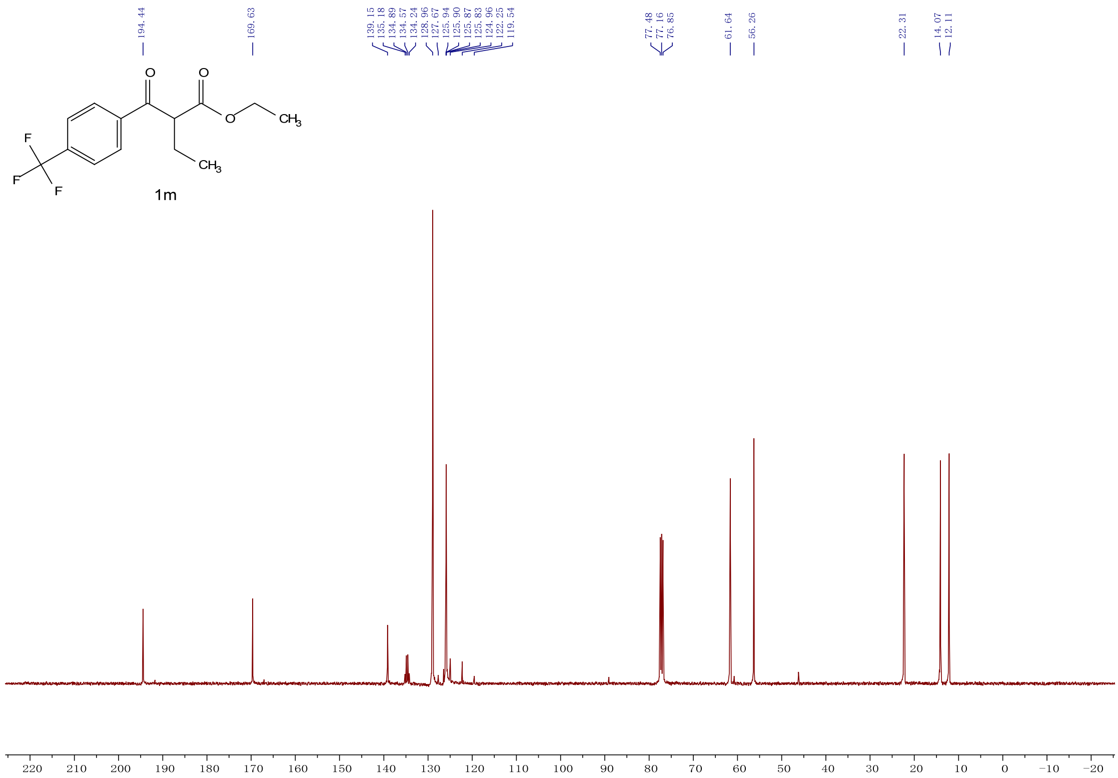

Supplementary Figure 132. ^{13}C NMR spectrum of 1m

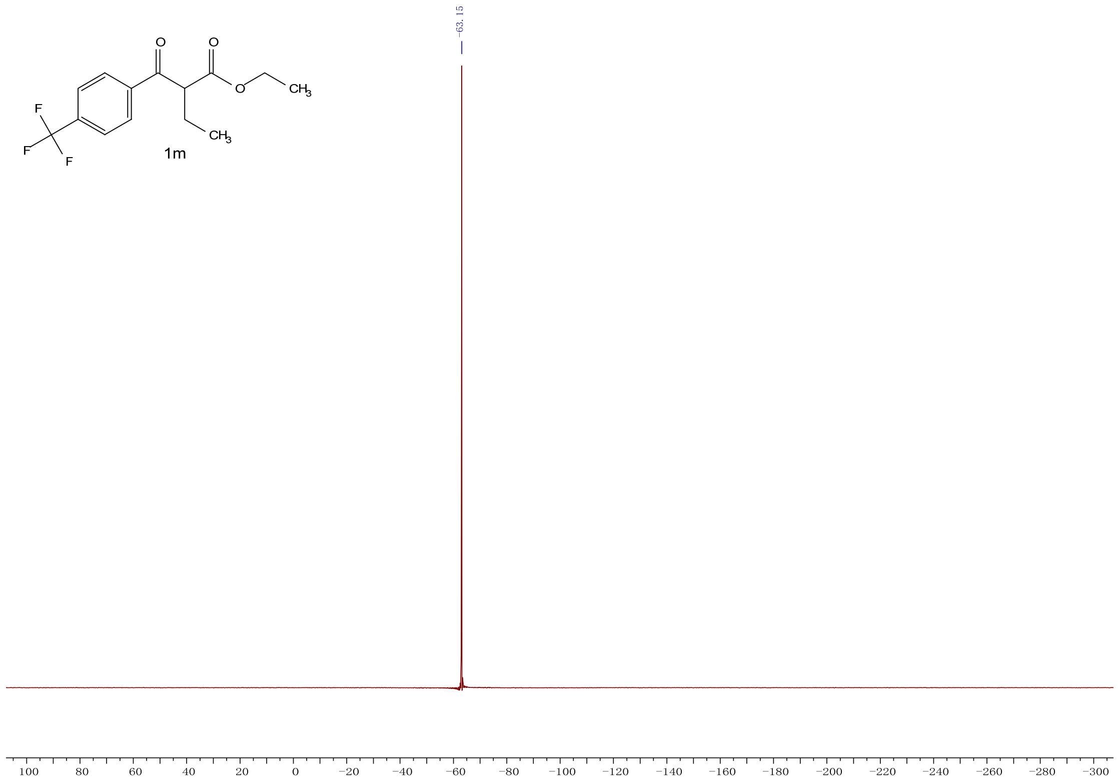

Supplementary Figure 133. ^{19}F NMR spectrum of 1m

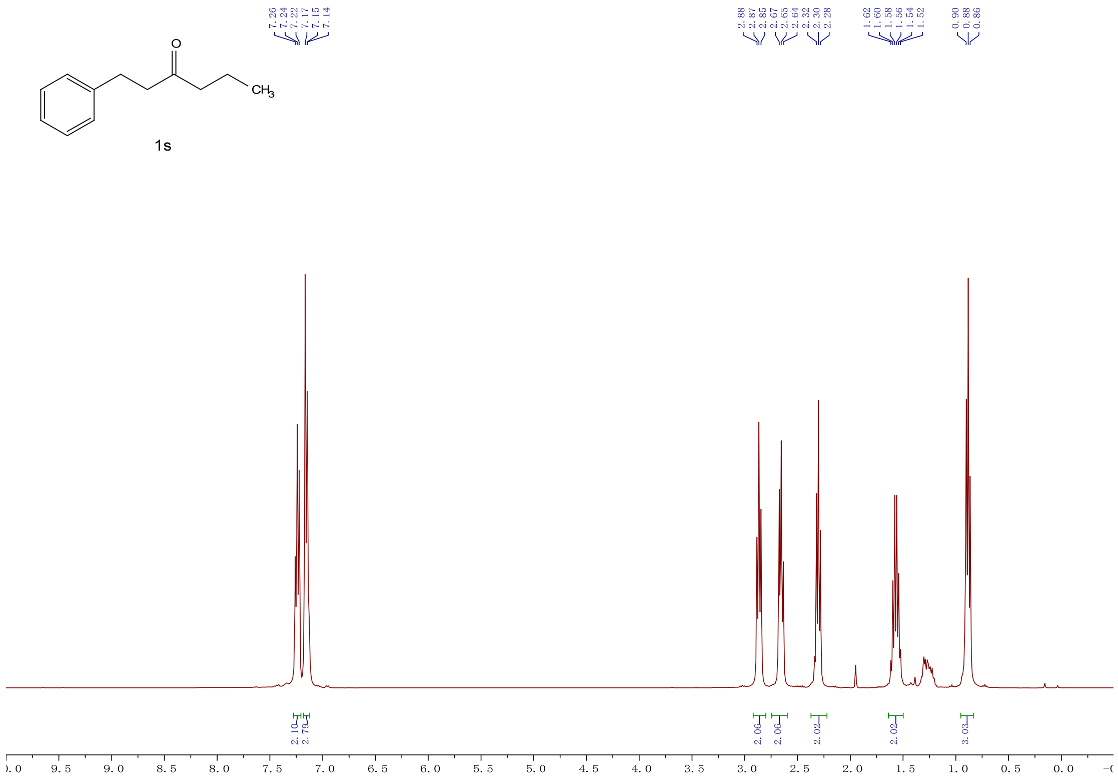

Supplementary Figure 134. ^{1}H NMR spectrum of 1s

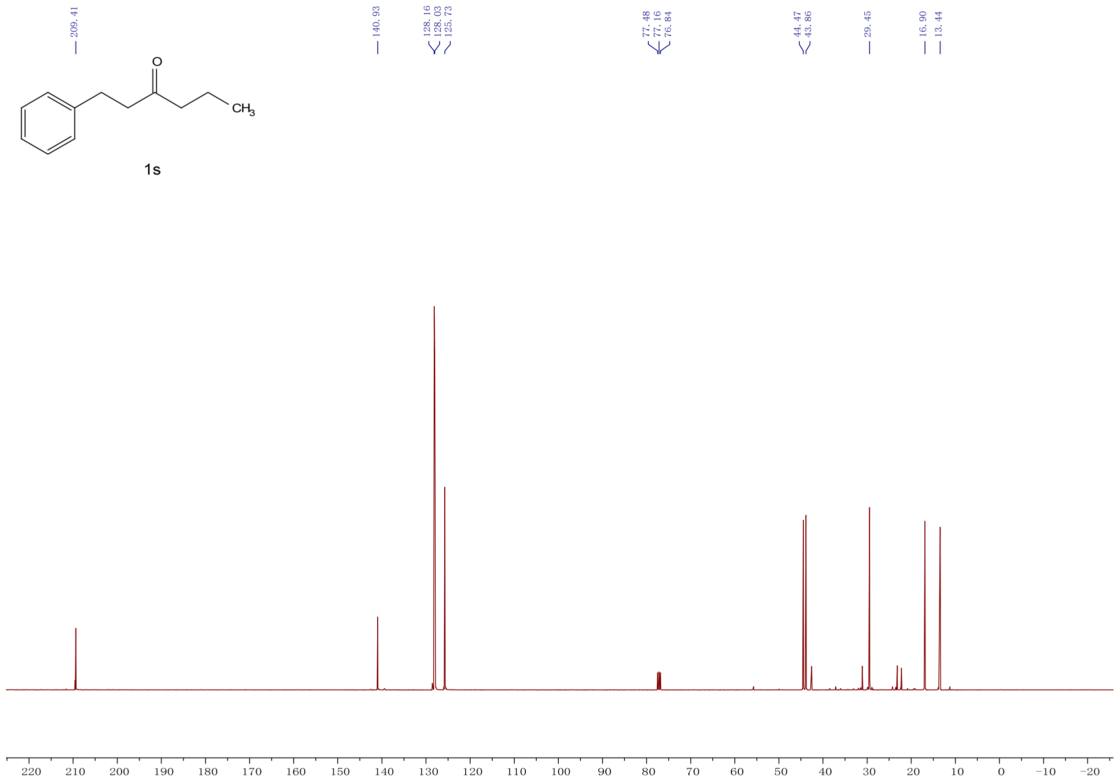

Supplementary Figure 135. ^{13}C NMR spectrum of 1s

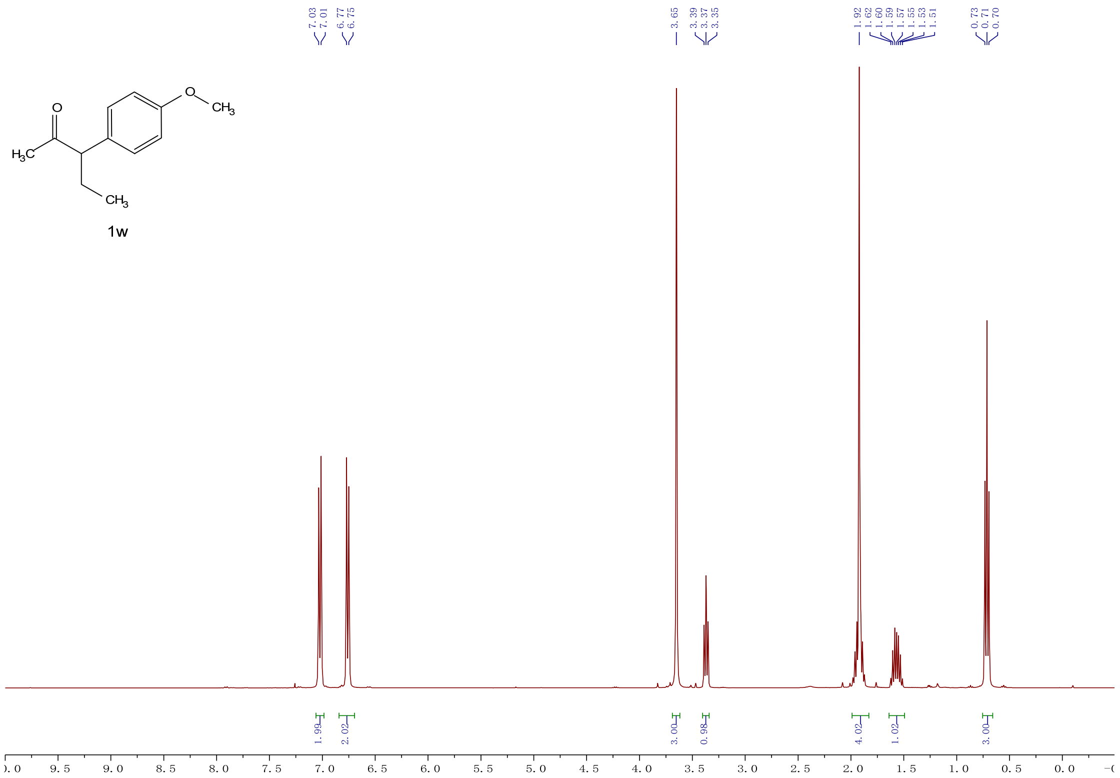

Supplementary Figure 136. ^{1}H NMR spectrum of 1w

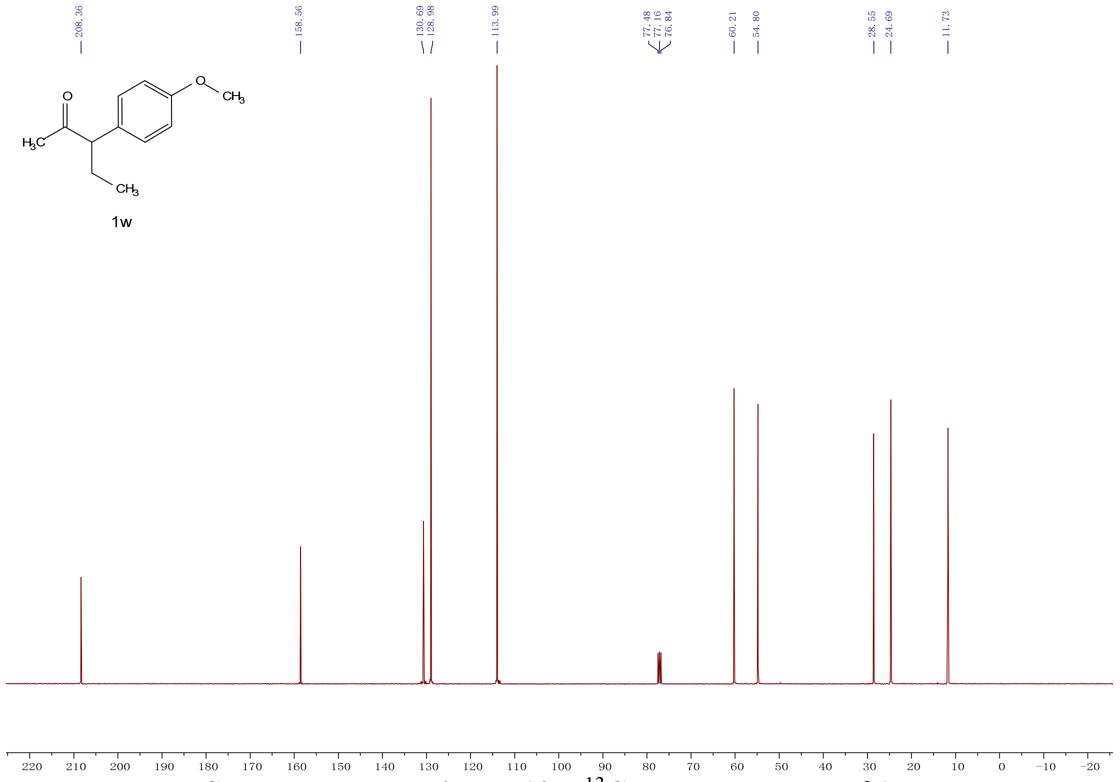

Supplementary Figure 137. ^{13}C NMR spectrum of 1w

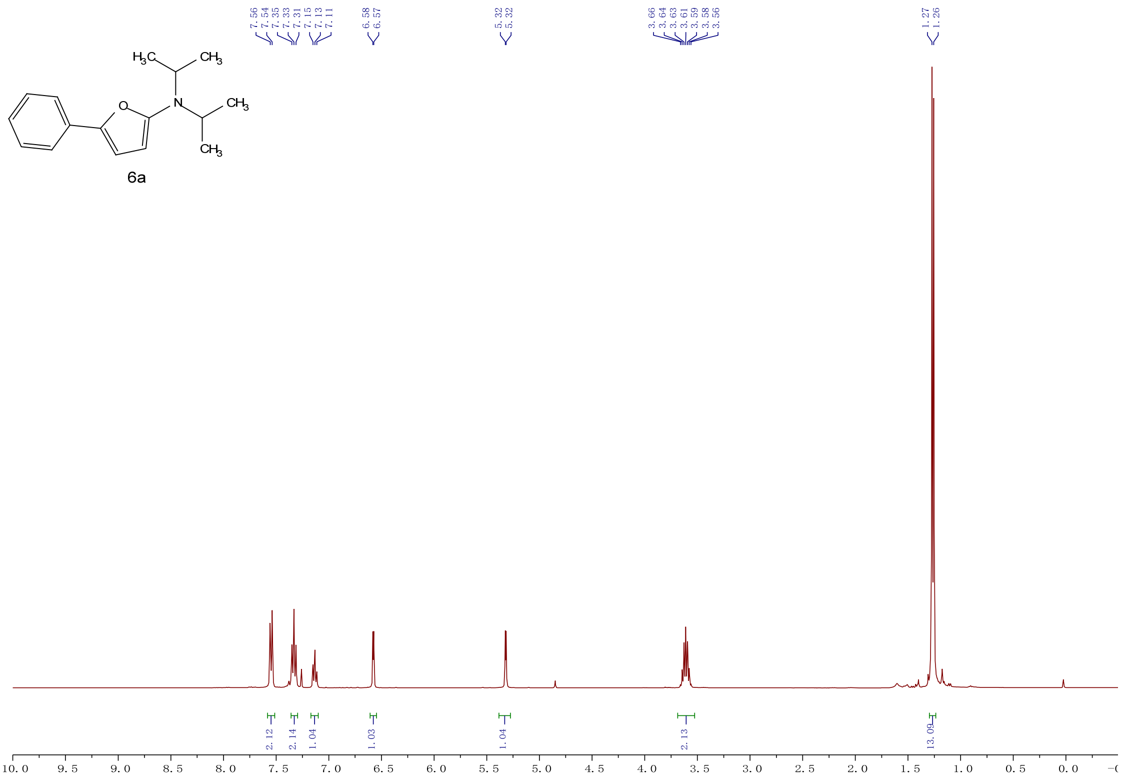

Supplementary Figure 138. ^{1}H NMR spectrum of 6a

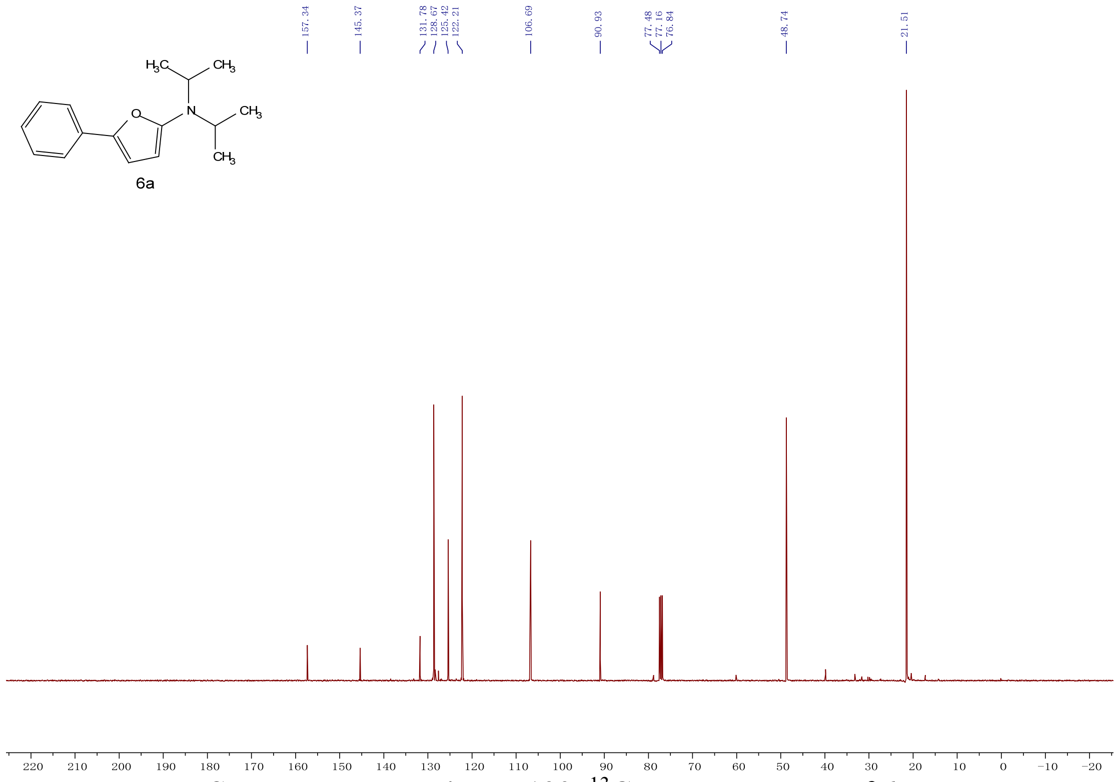

Supplementary Figure 139. ^{13}C NMR spectrum of 6a

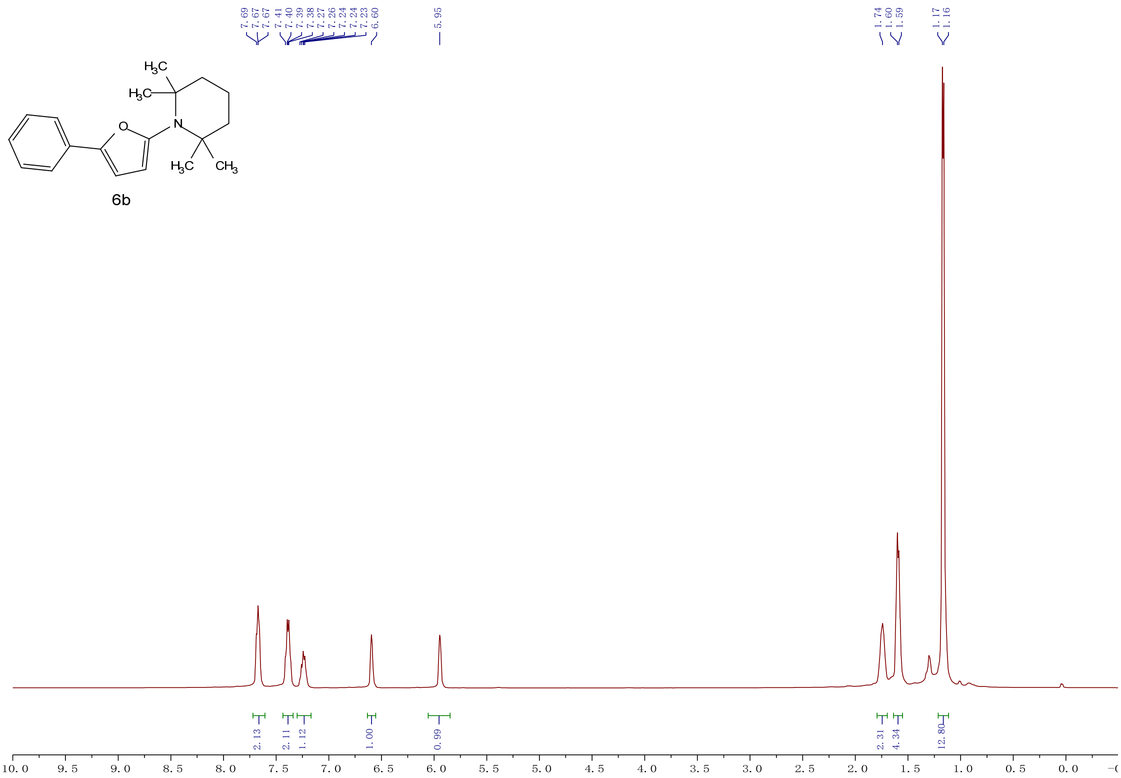

Supplementary Figure 140. ^{1}H NMR spectrum of 6b

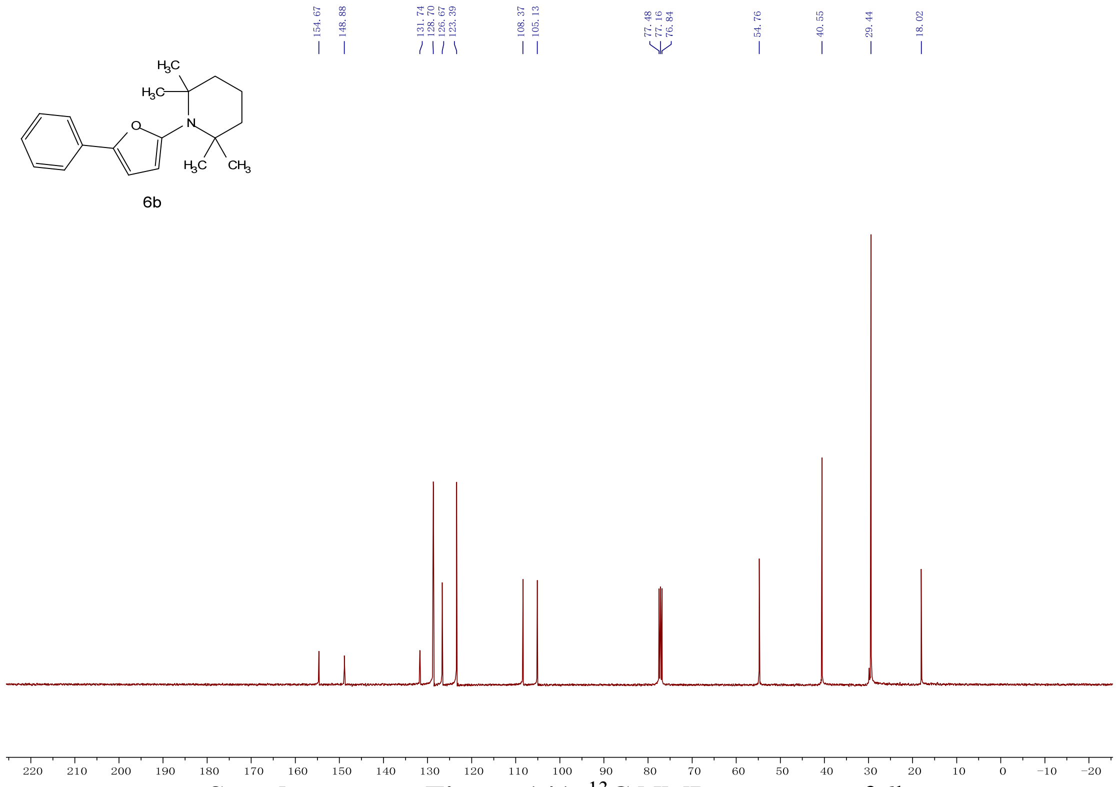

Supplementary Figure 141. ^{13}C NMR spectrum of 6b

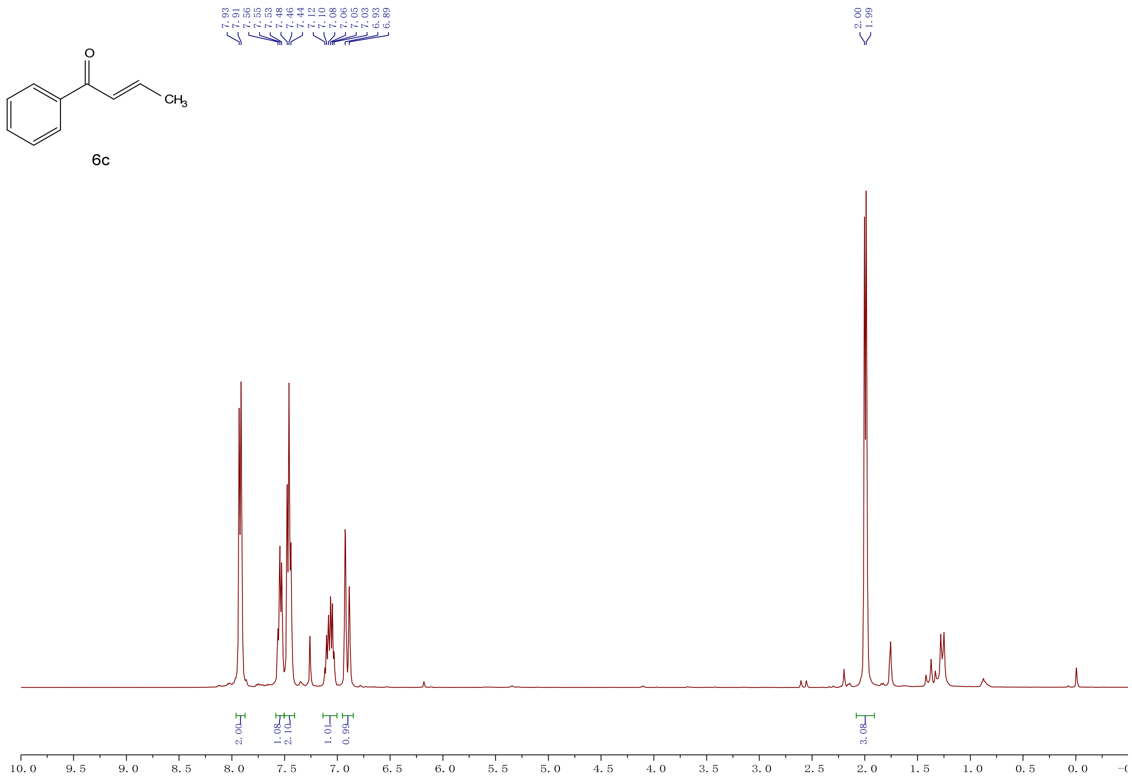

Supplementary Figure 142. ^{1}H NMR spectrum of 6c

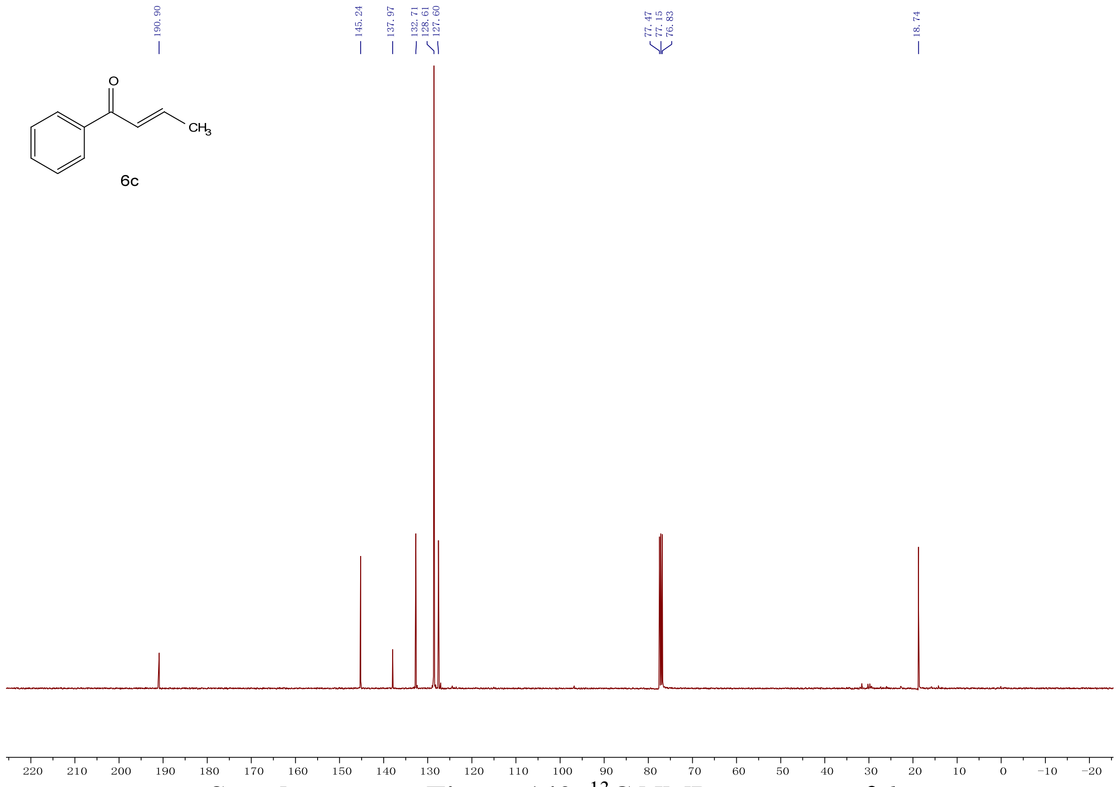

Supplementary Figure 143. ^{13}C NMR spectrum of 6c

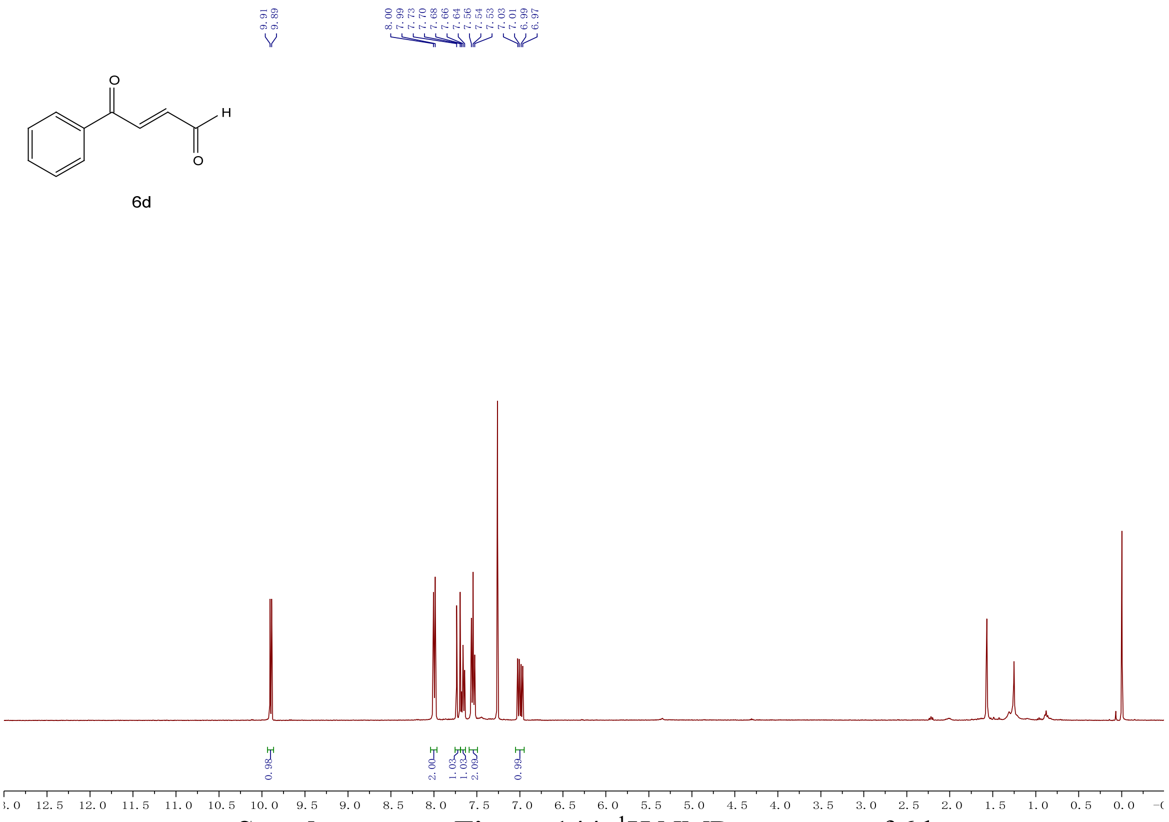

Supplementary Figure 144. ^{1}H NMR spectrum of 6d

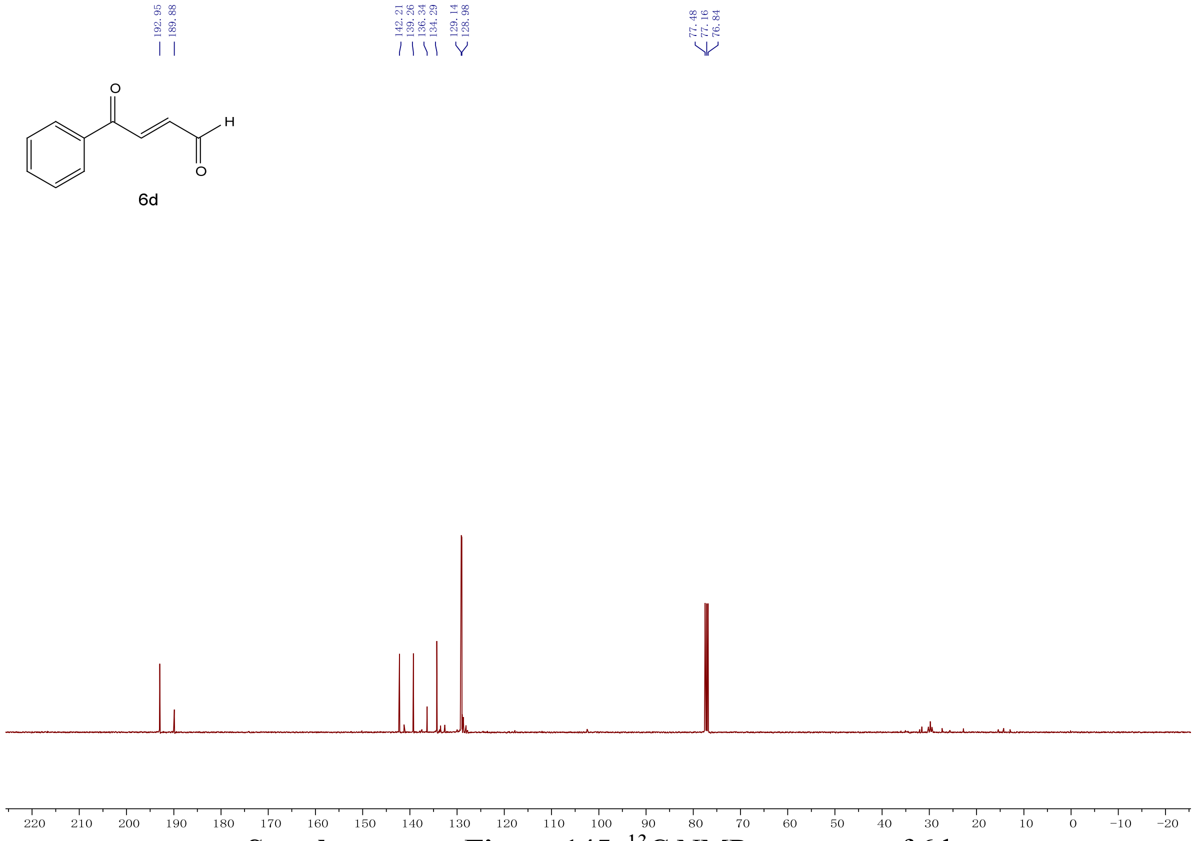

Supplementary Figure 145. ^{13}C NMR spectrum of 6d

Supplementary References

1. He Z, Li H, Li Z. Iodine-mediated synthesis of 3H-indoles via intramolecular cyclization of enamines. *J. Org. Chem.* **75**, 4636-4639 (2010).
2. Wang X, Widenhoefer RA. Palladium(II)- and platinum(II)-catalyzed addition of stabilized carbon nucleophiles to ethylene and propylene. *Chem Commun (Camb)*, 660-661 (2004).
3. Yuan N*, et al.* Probing the evolution of palladium species in Pd@MOF catalysts during the heck coupling reaction: an operando X-ray absorption spectroscopy study. *J Am Chem Soc* **140**, 8206-8217 (2018).
4. Kumar K*, et al.* A convenient synthesis of 4-alkyl-3-benzoylpyrroles from α,β-unsaturated ketones and tosylmethyl isocyanide. *Tetrahedron Lett.* **57**, 2315-2319 (2016).
5. Takemiya A, Hartwig JF. Palladium-catalyzed synthesis of aryl ketones by coupling of aryl bromides with an acyl anion equivalent. *J. Am. Chem. Soc.* **128**, 14800-14801 (2006).
6. Yoon IC, Kim TG, Cho CS. α-alkylation of ketones by trialkylamines under heterogeneous Pd/C catalysis. *Organometallics* **33**, 1890-1892 (2014).
7. Pan G-F, Zhu X-Q, Guo R-L, Gao Y-R, Wang Y-Q. Synthesis of enones and enals via dehydrogenation of saturated ketones and aldehydes. *Adv. Synth. Catal.* **360**, 4774-4783 (2018).
8. He M, Struble JR, Bode JW. Highly Enantioselective azadiene Diels−Alder reactions catalyzed by chiral N-heterocyclic carbenes. *J. Am. Chem. Soc.* **128**, 8418-8420 (2006).
