# Supplementary material for: Copper-catalyzed dehydrogenative γ-C(sp3)-H amination of saturated ketones for synthesis of polysubstituted anilines
Source: Nat Commun. 2019 Aug 15;10:3681. doi: 10.1038/s41467-019-11624-9 (PMC6695438; doi:10.1038/s41467-019-11624-9)
Supplement: Supplementary file 3 — Description of Additional Supplementary Files [file 41467_2019_11624_MOESM3_ESM.pdf]

Description of Additional Supplementary Files

File Name: Supplementary Data 1

Description: Optimization of the reaction conditions
