# Supplementary material for: Copper-catalyzed dehydrogenative γ-C(sp3)-H amination of saturated ketones for synthesis of polysubstituted anilines
Source: Nat Commun. 2019 Aug 15;10:3681. doi: 10.1038/s41467-019-11624-9 (PMC6695438; doi:10.1038/s41467-019-11624-9)
Supplement: Supplementary file 4 — Supplementary Data 1 [file 41467_2019_11624_MOESM4_ESM.pdf]

Supplementary Data 1. Optimization of the reaction conditions[a]

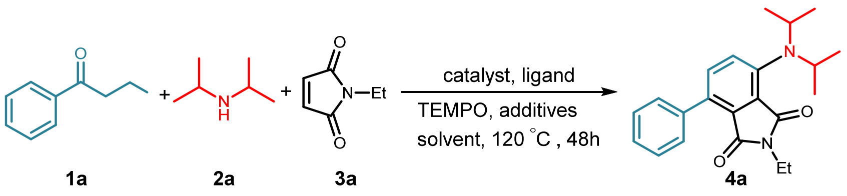

entry	catalyst	ligand	TEMPO	additives	solvent	yield (%)
1	$Cu(OAc)_2$	none	3.0 equiv.	-	$PhCH_3$	67
2	$Cu(OAc)_2$	bpy	3.0 equiv.	-	$PhCH_3$	80
3	$Cu(OAc)_2$	bpy	2.0 equiv.	-	$PhCH_3$	71
4	$Cu(OAc)_2$	bpy	3.0 equiv.	0.2 equiv. *o*-nitro-benzoic acid	$PhCH_3$	85
5	$Cu(OAc)_2$	bpy	3.0 equiv.	0.2 equiv. *o*-nitro-benzoic acid	*o*-dichlorobenzene	67
6	$Cu(OAc)_2$	bpy	3.0 equiv.	0.2 equiv. *o*-nitro-benzoic acid	CH_3CN	63
7	$Cu(OAc)_2$	bpy	3.0 equiv.	0.2 equiv. benzoic acid	$PhCH_3$	53
8	$Cu(OAc)_2$	bpy	3.0 equiv.	0.2 equiv. acetic acid	$PhCH_3$	56
**9**	**$Cu(OAc)_2$**	**bpy**	**3.0 equiv.**	**0.10 equiv. *p*-TsOH[b]**	**$PhCH_3$**	**96**
10	$Cu(OAc)_2$	bpy	3.0 equiv.	0.05 equiv. *p*-TsOH[b]	$PhCH_3$	87
11	$Cu(OAc)_2$	bpy	3.0 equiv.	0.2 equiv. *p*-TsOH[b]	$PhCH_3$	91
12	$Cu(OAc)_2$	bpy	3.0 equiv.	0.10 equiv. *p*-TsOH·H_2O	$PhCH_3$	83
13	-	-	3.0 equiv.	0.08 equiv. *p*-TsOH·H_2O	$PhCH_3$	30
14	$Cu(OAc)_2$	none	3.0 equiv.	0.10 equiv. *p*-TsOH[b]	$PhCH_3$	81
15	$Cu(OAc)_2$	1,10-Phen	3.0 equiv.	0.10 equiv. *p*-TsOH[b]	$PhCH_3$	83
16	$Cu(OAc)_2$	bpy	3.0 equiv.	200 mg 4Å MS	$PhCH_3$	51

17	$Cu(OTf)_2$	bpy	3.0 equiv.	-	$PhCH_3$	67
18	$Cu(OAc)_2$	bpy	3.0 equiv.	0.1 equiv. CsOAc	$PhCH_3$	81
19	$Cu(OAc)_2$	bpy	3.0 equiv.	0.2 equiv. CsOAc	$PhCH_3$	85
20	$Cu(OAc)_2$	bpy	3.0 equiv.	0.2 equiv. Cs_2CO_3	$PhCH_3$	61
21	$Cu(OAc)_2$	bpy	3.0 equiv.	0.2 equiv. Na_2CO_3	$PhCH_3$	53
22	$Cu(OAc)_2$	bpy	3.0 equiv.	0.2 equiv. NaOAc	$PhCH_3$	78
23	$Cu(OAc)_2$	bpy	3.0 equiv.	0.2 equiv. DBU	$PhCH_3$	58
24	$Cu(OAc)_2$	bpy	3.0 equiv.	0.2 equiv. DMAP	$PhCH_3$	69
25	$Cu(OAc)_2$	bpy	3.0 equiv.	0.2 equiv. DABCO	$PhCH_3$	51
26	$Cu(OAc)_2$	bpy	3.0 equiv.	0.2 equiv. CsF	$PhCH_3$	73
27	-	-	3.0 equiv.	0.1 equiv. *p*-TsOH[b]	$PhCH_3$	33
28	-	-	3.0 equiv.	0.2 equiv. *p*-TsOH[b]	$PhCH_3$	21
29	-	-	3.0 equiv.	-	$PhCH_3$	trace

[a]Reaction conditions: **1a** (0.8 mmol, 2.0 equiv.), **2a** (0.4 mmol), **3a** (0.6 mmol, 1.5 equiv.), catalyst (10 mol%), ligand (10 mol%), TEMPO, additives, solvent (1.5 mL), N_2, 120 °C for 48 h. Isolated yield. [b]12 wt.% *p*-TsOH solution in pure acetic acid.
